# Amenable epigenetic traits of dental pulp stem cells underlie high capability of xeno-free episomal reprogramming

**DOI:** 10.1186/s13287-018-0796-2

**Published:** 2018-03-20

**Authors:** Srijaya Thekkeparambil Chandrabose, Sandhya Sriram, Subha Subramanian, Shanshan Cheng, Wee Kiat Ong, Steve Rozen, Noor Hayaty Abu Kasim, Shigeki Sugii

**Affiliations:** 1 Department of Restorative Dentistry, Faculty of Dentistry, University of Malaya, 50603 Kuala Lumpur, Malaysia; 2 Fat Metabolism and Stem Cell Group (FMSCG), Laboratory of Metabolic Medicine (LMM), Singapore Bioimaging Consortium (SBIC), Helios, Biopolis, A*STAR, Singapore, 138667 Singapore; 3 Cancer and Stem Cell Biology Programme, Duke-NUS Medical School, Singapore, 169857 Singapore; 4 School of Pharmacy, University of Reading Malaysia, 79200 Johor, Malaysia; 5 Cardiovascular and Metabolic Disorders Programme, Duke-NUS Medical School, Singapore, 169857 Singapore

**Keywords:** Induced pluripotent stem cells, Dental pulp-derived mesenchymal stem cells, Stem cell therapeutics, Regenerative medicine, Xeno-free, Feeder-free, Episomal vector reprogramming

## Abstract

**Background:**

While a shift towards non-viral and animal component-free methods of generating induced pluripotent stem (iPS) cells is preferred for safer clinical applications, there is still a shortage of reliable cell sources and protocols for efficient reprogramming.

**Methods:**

Here, we show a robust episomal and xeno-free reprogramming strategy for human iPS generation from dental pulp stem cells (DPSCs) which renders good efficiency (0.19%) over a short time frame (13–18 days).

**Results:**

The robustness of DPSCs as starting cells for iPS induction is found due to their exceptional inherent stemness properties, developmental origin from neural crest cells, specification for tissue commitment, and differentiation capability. To investigate the epigenetic basis for the high reprogramming efficiency of DPSCs, we performed genome-wide DNA methylation analysis and found that the epigenetic signature of DPSCs associated with pluripotent, developmental, and ecto-mesenchymal genes is relatively close to that of iPS and embryonic stem (ES) cells. Among these genes, it is found that overexpression of *PAX9* and knockdown of *HERV-FRD* improved the efficiencies of iPS generation.

**Conclusion:**

In conclusion, our study provides underlying epigenetic mechanisms that establish a robust platform for efficient generation of iPS cells from DPSCs, facilitating industrial and clinical use of iPS technology for therapeutic needs.

**Electronic supplementary material:**

The online version of this article (10.1186/s13287-018-0796-2) contains supplementary material, which is available to authorized users.

## Background

The capability of human pluripotent stem cells to differentiate toward multilineage adult tissues provides ample cellular sources for cell therapy. Shinya Yamanaka and his group made the landmark discovery of reprogramming somatic cells by introducing transcription factors chosen from embryonic stem (ES) cells and transforming them into induced pluripotent stem (iPS) cells [1]. This exciting discovery paved the way for replacing the use of ethically and politically controversial ES cells in cell therapies. In vitro modelling of patients’ own cells mimicking human diseases became possible for studying the nature and complexity of genetic diseases and for application in drug discovery [2]. iPS technology can be used for elucidating the complexity of many human ailments, especially regarding genetic, degenerative, injured or age-associated conditions related to neurological systems, hepatic, cardiac, vision, bone disorders, wounding, autoimmune diseases, spinal cord injury, metabolic disorders, and certain types of cancers [3–5].

The initial attempts to induce reprogramming were undertaken with viral-mediated vectors for expressing the transcription factors; however, with time, multiple efforts were made to improve the reprogramming strategies to increase the efficiency of iPS generation and also to adhere to cell therapy practices. Attempts include improving the reprogramming strategies using synthetic mRNAs, synthetic miRNAs, recombinant proteins, temperature-sensitive Sendai virus, and episomal plasmids [6, 7]. Currently, standard iPS culture systems are innately unstable and difficult for application in clinical therapies due to the presence of various xenogenic factors, such as feeder layers, growth factors, and extracellular matrix (ECM) components in culture media, which makes the quality control of cells difficult. Consequently, to improve the clinical utility of iPS cells, attempts were made to use feeder layers, serum, or ECM of human origin for supporting the iPS culture in a physiological niche [8–10]. However, the complication of limited sources of human materials and their laborious maintenance efforts hampered the possibility of testing these cells in human clinical trials. Hence, to facilitate the implementation of iPS cell-based therapies, xeno-free and feeder-free alternative materials and methods minimizing human materials were developed, but this development has been relatively slow due to the lower efficiency of reprogramming in most of the cell types tested [8, 11].

Significant efforts have been made at looking for an ideal starting cell population for reprogramming [12]. It is generally better to have more developmentally immature cells as the starting source as they exhibit inherently higher proliferation, differentiation, and regenerative properties. This reduces the risk of mutations, chromosomal damage, or accumulated epigenetic changes compared to older cells [7]. We chose dental pulp stem cells (DPSCs) as the starting material in our experiments as they are known for their outstanding biological characteristics [13]. DPSCs are immature mesenchymal stem cells (MSCs) placed in the same lineage of cells as the umbilical cord- and Wharton’s Jelly-derived stem cells [14]. DPSCs are a rich source of potent MSCs despite the small amount of tissue samples obtained [15]. They are highly multipotent, have immunomodulatory properties, and can be obtained relatively easily by tooth extraction procedures [16–19]. The isolation procedure of the DPSCs is simple and robust. Remarkably, DPSCs also support reprogramming and induction of pluripotency in a more refined manner, possibly due to their dual mesectodermal and neural crest origins and inherent expression of pluripotent factors including Oct-4, Nanog, c-Myc, Sox2, stage-specific embryonic antigens (SSEA-3, SSEA-4), and tumour recognition antigens (TRA-1-60 and TRA-1-81) [15, 17, 20, 21]. Altogether, these factors provide a compelling reason to use DPSCs for establishing a robust iPS reprogramming protocol.

In this manuscript, we report a clinically applicable method to efficiently derive human iPS cells from DPSCs using episomal vector transduction and culturing in xeno-free media. We compare reprogramming of DPSCs with that of adipose-derived stem cells (ASCs) which were previously shown to be superior to most other cell types in reprogramming efficiency and feeder independence [22]. The amenable propensity for reprogramming is at least explained by the characteristic epigenetic signatures of DPSCs, which show overlap in DNA methylation levels with iPS cells in a number of pluripotent, developmental, and mesenchymal gene loci. Among these, at least two genes, *PAX9* and *HERV-FRD*, appear to affect reprogramming since overexpression of *PAX9* and knockdown of *HERV-FRD* result in improvement in iPS generation efficiencies.

## Methods

### Isolation of primary DPSCs and cell culture

To derive human DPSCs, intact human teeth were collected with informed consent from patients undergoing extraction at the Faculty of Dentistry, University of Malaya, Malaysia. Under sterile conditions, the root surfaces of the teeth were cleaned with povidone-iodine (Sigma-Aldrich, St. Louis, MO, USA) and the pulp was extracted within 2 h post-extraction. Thereafter, the tissues were kept in a 1.5-ml tube in 1× knockout Dulbecco’s modified Eagle’s medium (KO-DMEM; Invitrogen), 10% fetal bovine serum (FBS; Hyclone), 2% penicillin/streptomycin (P/S; Invitrogen), 5% Glutamax (Invitrogen), 100 μg/ml ascorbic acid (Sigma-Aldrich), and 1× insulin**-**transferrin**-**selenium (ITS; Invitrogen) and transported to the laboratory for isolation of the cells. The pulp tissue was minced into small fragments prior to digestion in a solution of 3 mg/ml collagenase type I (Gibco) for 40 min at 37 °C. After neutralisation with 10% FBS, the cells were centrifuged, seeded in a T25 culture flask (BD Biosciences) with culture medium containing KO-DMEM, 10% FBS, 1× P/S, and 1% Glutamax, and incubated in humidified atmosphere of 5% CO_2_ at 37 °C. Non-adherent cells were removed 48 h after initial plating. The medium was replaced every 3 days until the cells reached 80–90% confluency. The DPSCs were further passaged and frozen down in Bambanker (Lymphotec) and stored in liquid nitrogen for future use.

The list of commercial (Lonza and Allcells) and patient-derived dental cells used in this manuscript can be found in Additional file 1: Table S1. DPSCs were grown in vitro in Poietics™ DPSC BulletKit medium (Lonza) according to the manufacturer’s instructions. ASCs were cultured in DMEM containing 15% FBS, non-essential amino acids (NEAA; 1%), basic fibroblast growth factor (bFGF; 5 ng/ml) and P/S as previously described [9, 23, 24]. Upon reaching cell confluency of 80–90%, cells were detached using TrypLE Express and split according to the experimental requirements. Media change for the cells was performed every 2–3 days. For xeno-free culture of DPSC lines, StemPro MSC SFM Xenofree (Lonza) was used from a very early passage (passage 2). DPSC proliferation was slower in xeno-free media when compared to normal media, consistent with earlier reports [25, 26]. All cells were cultured in a humidified incubator at 37 °C in 5% CO_2_.

### Flow cytometric analysis

Flow cytometric analysis for MSC surface markers was performed for both normal and xeno-free conditioned DPSCs. Cultured DPSCs were trypsinized and a total of 2 × 10^5^ cells were suspended in staining buffer (DMEM without phenol red with 2% FBS) as described previously [23]. Cells were then stained directly with fluorochrome-conjugated antibodies and incubated on ice for 30 min. Antibodies used were CD31_FITC, CD105_Alexa Fluor 647-A, CD73_FITC, CD14_Alexa Fluor 647-A, CD90_FITC, and CD45_Alexa Fluor 647-A (all provided by BD Biosciences). After staining, cells were washed and suspended in sorting buffer before analysis by flow cytometry (LSR II, BD Biosciences).

### Culture of human ES and iPS cells

The human ES cell line (H1) was obtained from WiCell Research Institute and cultured on Matrigel (Corning) or MEF feeder cells (GlobalStem) in hES medium as described previously [9]. H1 hES cell line and established iPS cell lines were maintained in either of the following combinations: MEF feeders with hES medium (DMEM/F12 supplemented with 20% knock-out serum replacement (Invitrogen), 1 mM Glutamax, 1 mM NEAA (Invitrogen), P/S, 0.1 mM β-mercaptoethanol (Millipore), and 10 ng/ml b-FGF for feeder-dependent culture; Matrigel-coated plates with hES cell qualified mTeSR1 (Stemcell Technologies) for feeder-free culture; or CELLstart (Life Technologies)-coated plates with NutriStem medium (Stemgent) for xeno-free, feeder-free culture. Fresh medium change was performed every day and cells were passaged 1:3–1:5 using dispase (Stemcell Technologies) or collagenase IV (Stemcell Technologies). Detailed methods for human pluripotent stem cell passaging have been described previously [9]. Human iPS cell lines from ASCs were established as previously described [9, 22].

### Viral-based iPS reprogramming protocol

Yamanaka’s pMX-based retroviral vectors were from Addgene (# 17217, 17218, 17219, and 17220 for human Oct4, Sox2, Klf4, and c-Myc) [1]. To estimate transduction efficiencies, pMX-based green fluorescent protein (GFP) vector (Cell Biolabs) was used [9, 27]. These plasmids were transfected into the 293 T packaging cell line that incorporated gag/pol packaging, and VSV-G envelope plasmids (Addgene # 8449 and 8454) to generate high titre retroviruses as previously described [9]. Forty-eight hours post-transfection, viral supernatant was collected and filtered with 0.45-μm syringe filters, and 1 × 10^6^ cells were seeded in a 10-cm culture dish. The following day, cells were transduced with viral supernatants with equal volume of each factor supplemented with 8 μg/ml polybrene (Sigma-Aldrich) and incubated overnight. The following day, fresh expansion media were added to the cells. The next day, cells were harvested with TrypLE Express, and transferred to a 10-cm dish that had been seeded with either feeder (MEF) or feeder-free (Matrigel) based culture conditions. The following day, medium was changed with hES or mTESR1 cell culture medium. The reprogramming efficiency was calculated as the percentage of iPS colony number per number of GFP-positive cells.

### Episomal-based iPS reprogramming protocol

Episomal plasmids developed by Yamanaka’s lab were obtained from Addgene: pCXLEhOct3/4-shp53-F (Addgene # 27077), pCXLE-hSK (Addgene # 27078), pCXLE-hUL (Addgene # 27080), and pCXLE-EGFP (Addgene # 27082) [28]. Cells (1 × 10^6^) were harvested using TrypLE and the cell pellet was washed once in 1× phosphate-buffered saline (PBS). The cells were then re-suspended in Nucleofector solution supplied in the Nucleofector kit R (Lonza), and 1 μg of each plasmid was added to the cell suspensions for each reaction as recommended by the manufacturer. Next, cell suspensions were transfected with the Program FF-113 on a Nucleofector 2b Device. After 10 min of incubation, the transfected cells were re-suspended in respective MSC culture medium (either DPSCGM or StemPro MSC SFM Xenofree) in 10-cm culture dishes. Early passage MSCs in the growth phase (less than passage 5) were used for all experiments. The reprogramming efficiency was calculated as the percentage of iPS colony number per number of EGFP-positive cells.

For both sets of reprogramming experiments (using viral and episomal vectors) further enrichment was carried out using small molecules to improve the reprogramming process and improve iPS generation quality [6]. Starting from the next day of transfection, daily media change of respective MSC culture medium was performed which was supplemented with 0.5 mM sodium butyrate (Sigma-Aldrich). On day 7 post-transfection, 1 × 10^5^ viable cells were seeded over MEF feeders for feeder-based iPS derivation; 2 × 10^5^ viable cells were seeded for feeder-free iPS derivation into one well of a six-well plate, which was either pre-coated with Matrigel or CELLstart (for the xeno-free condition). The following day, MSC medium was changed to hES medium (feeder layer) or mTeSR1 (feeder-free) or NutriStem (xeno-free), supplemented with 0.5 mM sodium butyrate. At 12 days post-transfection, supplementation of sodium butyrate was stopped, and conditioned further with SMC4 cocktail (consisting of small molecules of PD0325901, CHIR99021, Thiazovivin and SB431542 (FOCUS Biomolecules)) in hES medium/mTeSR1/NutriStem. This media supplement was continued until initial colony formation began, usually between 13 and 17 days post-transfection. When iPS colony formation was observed, the SMC4 addition was stopped and only hES/mTeSR1/NutriStem medium was used to culture and further passage the colonies.

### Over-expression and knock-down and of PAX9 and HERV-FRD

For the over-expression study, DPSCs (L1) were nucleofected simultaneously using episomal reprogramming factors as mentioned above along with the PCMV6-AC-GFP vector expressing human PAX9 (OriGene cat. # PS100019) for over-expression of PAX9. As the control, the empty vector pCMV6-AN-GFP was used. For the knock-down study, DPSCs (L1) were initially nucleofected using episomal reprogramming factors as mentioned above, and then incubated for a period of 10 min. This was followed by the addition of small-interfering (si)RNA against human ERVFRD-1 (ON-TARGET plus; GE Dharmcon) for transient silencing of HERV-FRD during reprogramming. The siRNA control was the vehicle alone. Both groups of nucleofected cells were then used for the downstream procedure as discussed previously in our methods.

### Immunofluorescence live cell staining

Reprogrammed cells were immune-stained with fluorescent live cell stain TRA-1-60 (R&D Systems, GloLIVE NL557), TRA-1-81 (MACS Miltenyi Biotec), and alkaline phosphatase (Life Technologies) as per the manufacturers’ instructions. After incubating with the live staining antibodies, cells were washed three times with PBS and images were immediately captured using a Nikon microscope TS100 and ImageXpress Micro High Content Image System.

### Gene expression analysis

Total RNA was isolated from cells in TRIzol reagent (Invitrogen), and purified using the RNA easy kit Column (Qiagen) as per the manufacturer’s instruction. Complementary DNA (cDNA) was synthesised from purified RNA using the RevertAid H minus first strand cDNA synthesis kit (Fermentas). Quantitative polymerase chain reaction (qPCR) was subsequently carried out using SYBR Green PCR Master Mix on a StepOnePlus Real-Time PCR System (Applied Biosystems). Primer sequences used in the current study are listed in Table 1. cDNA from the H1 hES cell line was used as a control for the hiPS lines. Samples were run in triplicates and their relative mRNA expression was calculated using the ΔΔct method and normalised to *GAPDH*.

**Table 1:** Primer sequences used for quantitative polymerase chain reaction analysis

Gene	Forward 5’ to 3’	Reverse 5’ to 3’
*GAPDH*	CAAGGTCATCCATGACAACTTTG	GGCCATCCACAGTCTTCTGG
*ACTA*	CTCGGAGATCATCACGTTTG	CCTTGGAAATCTCGAAGTGC
*LIN28*	GAAGCGCAGATCAAAAGGAG	GCTGATGCTCTGGCAGAAGT
*OCT4*	GCAAAACCCGGAGGAGTC	CCACATCGGCCTGTGTATATC
SOX2	TTGCTGCCTCTTTAAGACTAGGA	CTGGGGCTCAAACTTCTCTC
*NANOG*	CCAACATCCTGAACCTCAGC	GCTATTCTTCGGCCAGTTG
*DPPA2*	TGGTGTCAACAACTCGGTTTG	CTCGAACATCGCTGTAATCTGG
*TGFb1*	GCAGCACGTGGAGCTGTA	CAGCCGGTTGCTGAGGTA
*PDGFRA*	AGGTGGTTGACCTTCAATGG	TTTGATTTCTTCCAGCATTGTG
*FN1*	CTGGCCGAAAATACATTGTAAA	CCACAGTCGGGTCAGGAG
hERVFRD	TGAGGAGGGCAATCCATTTCAT	TGCATGGTCGTTAAGGCTT
*hPAX9*	GTTATGTTGCTGGACATGGGT	GAAGCCGTGACAGAATGACTAC

### Karyotyping

Actively dividing human ES and iPS cells were cultured in 25-cm^2^ culture flasks. Upon reaching 70–80% confluency, cell cultures were transported at room temperature to the Cytogenetics Laboratory. Karyotyping analysis was performed by the Cytogenetics Laboratory from the Department of Pathology and Laboratory Medicine, at KK Women’s and Children’s Hospital, Singapore.

### In vitro differentiation analysis

For spontaneous in vitro differentiation, DPSC-derived iPS (DiPS) cells were grown to confluency and passaged by dispase. Cells were resuspended in hES medium (without bFGF) and transferred to low-attachment six-well plates (Greiner Bio One). Medium change was made every 3 days. Embryoid bodies (EBs) were formed [29]; day 8–10 EBs were transferred to a 12-well plate precoated with 0.1% gelatin and cultured for a further 12 days. Subsequently, the attached EBs were allowed to undergo spontaneous differentiation. These differentiated cells were later stained with three germ layer immunocytochemistry antibodies (Life Technologies) as per the manufacturer’s instructions.

### In vivo differentiation analysis

For in vivo pluripotency determination, a teratoma formation assay was performed. DiPS cells were grown to confluency and passaged by dispase. Approximately 2–5 × 10^6^ cells (one confluent well of a six-well plate) was used per animal. Cell pellets were resuspended in a mixture of 50% Matrigel and 50% mTeSR1, and 150 μl of the iPS cell suspension was injected into the subcutaneous area above the thigh (quadriceps) muscle of 6- to 8-week-old NOD-SCID mice under isoflurane anaesthesia with isoflurane. After 8–10 weeks, when a teratoma developed, animals were sacrificed by carbon dioxide asphyxiation, and the teratomas were excised and fixed in 4% paraformaldehyde, prepared for paraffin sections, and stained for haematoxylin and eosin (H&E). Histological processing and H&E staining were performed by the Advanced Molecular Pathology Laboratory from the Institute of Molecular and Cell Biology, Singapore*.*

### DNA methylation analysis

ASC-derived iPS (AiPS) and DiPS cells were used. Genomic DNA from AiPS/DiPS and from their corresponding mesenchymal starting cell populations was extracted from approximately 1 × 10^6^ cells using a QIAamp DNA Mini Kit (Qiagen) as per the manufacturer’s instructions. Approximately 1 μg genomic DNA of each sample was bisulphite converted and subsequently processed for profiling with the Illumina Infinium DNA methylation platform (HumanMethylation450 BeadChip) at the Genome Biology Facility at Duke-NUS Medical School, Singapore. The methylation data were subject to quality control and processed in R using the Minfi package (version: 1.18.2) [30], removing probes with a detection *p* value greater than 0.05 in more than 25% of all samples, probes which contain common single nucleotide polymorphisms (SNPs), and probes on the sex chromosomes. Differential methylation analysis based on M values was performed using two-way analysis of variance (ANOVA), controlling for matched sample sources and correcting for multiple testing using the Benjamini-Hochberg method. In addition, ingenuity pathway analysis (IPA) was also performed to analyse the set of genes that undergo significant epigenetic changes while transforming from the somatic cell state into the iPS cell state.

### Neural progenitor cell generation

Neural progenitor cells (NPCs) were generated from DiPS cells and H1 cells using STEMdiff™ Neural Induction Medium (Stemcell Technologies) using an EB-based protocol as per the manufacturer’s instructions.

### Statistical analysis

Data are expressed as mean ± standard deviation (SD), unless otherwise stated. All experimental assays were performed in triplicates. For differential methylation analysis, M values were determined using two-way ANOVA and corrected for multiple testing using the Benjamini-Hochberg method.

## Results

### Reprogramming of DPSCs using viral and episomal methods

The conventional standard reprogramming protocol (using Yamanka’s viral plasmids of *OCT4*, *SOX2*, *KLF4*, and *C-MYC*) was performed initially to ascertain the reprogramming threshold of DPSCs that could be achieved in our laboratory. This four-factor retroviral transduction method yielded approximately 0.02% (feeder-free) to 0.05% (feeder) efficiency in terms of iPS colonies, and approximately 80–90 colonies in one well of a six-well culture plate when counted at post-transduction days (d)20–30. The number of transduced cells was assessed by a GFP-encoding retroviral vector, as shown in Additional file 1: Figure S1a, which was estimated to be 85–90% based on cell count. Several DPSC-derived iPS (DiPS) cell lines generated using the retrovirus method were established, but only one line (L1_viral) was used as a standard for further downstream characterisation.

In parallel, experiments were conducted using episomal vectors which contained the transcription factors *OCT4*, *SOX2*, *KLF4*, *L-MYC*, *LIN28*, and sh*TP53* (knock-down of p53) [28]. These constructs are non-viral and non-integrative, and oncogenic *C-MYC* was replaced with non-oncogenic *L-MYC*, assuring more safety while maintaining reprogramming efficiency. The episomal reprogramming method resulted in a lesser transfection efficiency of about 20–30% only, as shown in Additional file 1: Figure S1b, c, based on GFP-positive cells. The reprogramming efficiency in DPSCs using episomal vectors was 0.05% in the feeder-free condition. Other human cell types, such as ASCs, were reported to inherently enable feeder-free reprogramming. Surprisingly, the reprogramming efficiency of DPSCs under feeder-free conditions was comparatively higher than that of ASC-derived iPS colonies, which was approximately 0.008% [22, 31].

### Improvement of iPS colony formation time and efficiency using SMC4 cocktail

To improve the reprogramming efficiency of DPSCs further, we supplemented the reprogramming media with small molecules (“SMC4 cocktail”) during feeder and feeder-free reprogramming using both viral transduction (control) and episomal transfection. SMC4 consists of inhibitors for transforming growth factor (TGF)β, MEK, GSK3, and ROCK [32]. Colony formation on feeder layers was observed in those supplemented with the SMC4 cocktail as early as d13, while colony formation was observed in the control (without SMC4) group only at d18–22 (Additional file 1: Figure S2a). Under feeder-free conditions, SMC4 addition accelerated colony formation as early as d18 when compared with d22 in the control group (Additional file 1: Figure S2a). Overall, SMC4 addition improved the reprogramming efficiency and colony formation in cells that were transfected with episomal vectors using the nucleofection method in feeder-free conditions. At d22–24, the colonies were manually picked and passaged. Additional file 1: Figure S3 shows the morphological changes in DPSCs during reprogramming at different days post-reprogramming using episomal vectors on feeder layers. SMC4 addition strikingly resulted in not only early emergence of colonies, but also in a more compact iPS colony appearance and increased number of colonies, indicative of higher pluripotency and quality of the iPS colonies, as shown in Additional file 1: Figure S2b and Table S2. Hence, episomal-based DiPS generation was significantly improved with SMC4 addition, especially in the feeder-free condition, and thus was chosen for further studies. This method also allowed us to pursue reprogramming using animal source-free (xeno-free) generation of iPS colonies, which would be more ideal for future clinical applications.

### Xeno-free and feeder-free episomal method for generating DiPS cell lines

**Fig. 1:**
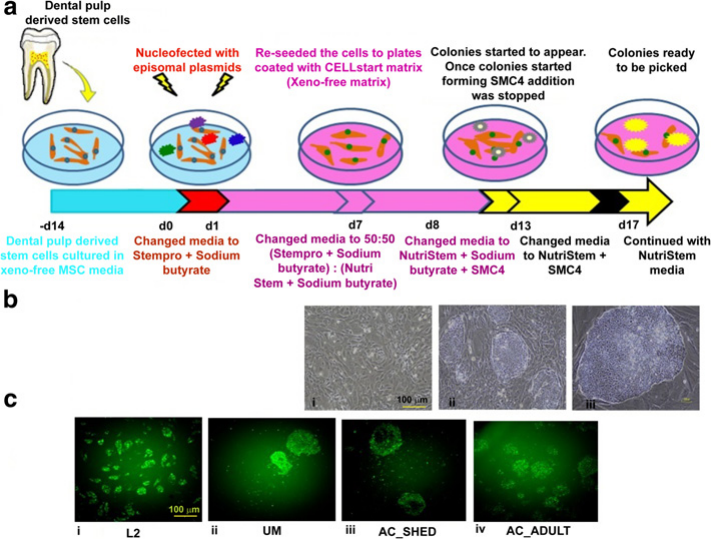
Generation of clinically compatible DPSC-derived iPS cells using virus-free, xeno-free, and feeder-free approaches. **a** Schematic time course of DiPS generation under xeno-free conditions and using small molecules. **b** Morphology of L1 DPSCs post-nucleofection under xeno-free and feeder-free conditions at post-transduction days (d)7 (i), d13 (ii), and d17 (iii). **c** DiPS colonies expressing TRA-1-81 marker which were picked and expanded for downstream characterisation. MSC mesenchymal stem cell

Since DPSCs undergo efficient reprogramming by nucleofection with episomal vectors in the feeder-free condition, we attempted to establish the xeno-free reprogramming protocol to generate clinical-grade iPS colonies. As depicted in the schematic time course in Fig. 1a, DPSCs (*n* = 5) were cultured in xeno-free MSC media and evaluated for their MSC characteristics by flow cytometry. As shown in Additional file 1: Figure S4a, b, DPSCs grown in xeno-free media exhibited standard MSC characteristics in terms of morphology and cell surface expression of the MSC markers (CD105^+^, CD73^+^, CD90^+^, CD31^−^, CD14^−^, and CD45^−^).

The next day following transfection of the DPSCs grown in xeno-free conditions with episomal vectors, the cells were supplemented with sodium butyrate (0.5 mM). At d7, the media was changed to NutriStem (xeno-free reprogramming/iPS media). Visible cell morphological changes were observed from d9 to d10 from fibroblast-like cells to more circular structures, later forming a cluster and displaying the first emergence of iPS colonies (Fig. 1a). At d13, smaller colonies were formed, and at d17 the colonies grew bigger, as shown in Fig. 1a, b. Compared to the feeder-free reprogramming method (Additional file 1: Figure S2a), this was significantly much earlier, demonstrating the potency of the xeno-free protocol. At d18, TRA-1-81-positive DiPS colonies (Fig. 1c) were counted and the reprogramming efficiency was determined further by dividing by the number of GFP-positive cells, estimated through flow cytometry (Additional file 1: Figure S1c). Different DPSC lines from young and adult humans were tested, with reprogramming efficiencies ranging from 0.04% to 0.26% (mean = 0.16%, median = 0.19%; Table 2). Several clones from each of all the DPSC lines were established and used for downstream analysis. All established DiPS lines displayed standard human ES-like morphology with a high nucleus to cytoplasm ratio, more visible nucleoli, and with firmly packed colonies (Fig. 1b). Collectively, our method establishes more convenient, robust, and improved xeno-free and integration-free reprogramming protocol for DPSCs.

**Table 2:** Reprogramming efficiencies of DPSCs cultured under xeno-free and feeder-free conditions

DPSC sources	Transfection/transduction efficiency (GFP^+^ cells) (%)	No. of TRA-1-81-positive colonies^a^	Reprogramming efficiency^b^ (%)
L1	33.4	92.6 ± 5.1	0.13
L2	36.5	147 ± 12.03	0.20
AC_SHED	40.3	158 ± 15.5	0.19
AC_ADULT	22.2	115.6 ± 11.3	0.26
UM	19.1	18.66 ± 8.9	0.04

*DPSC* dental pulp stem cell, *GFP* green fluorescent protein

^a^*n* = 3, mean ± SE

^b^Reprogramming efficiency (%) = number of iPS colonies/number of transduced or transfected cells

### Characterisation of DiPS lines

**Fig. 2:**
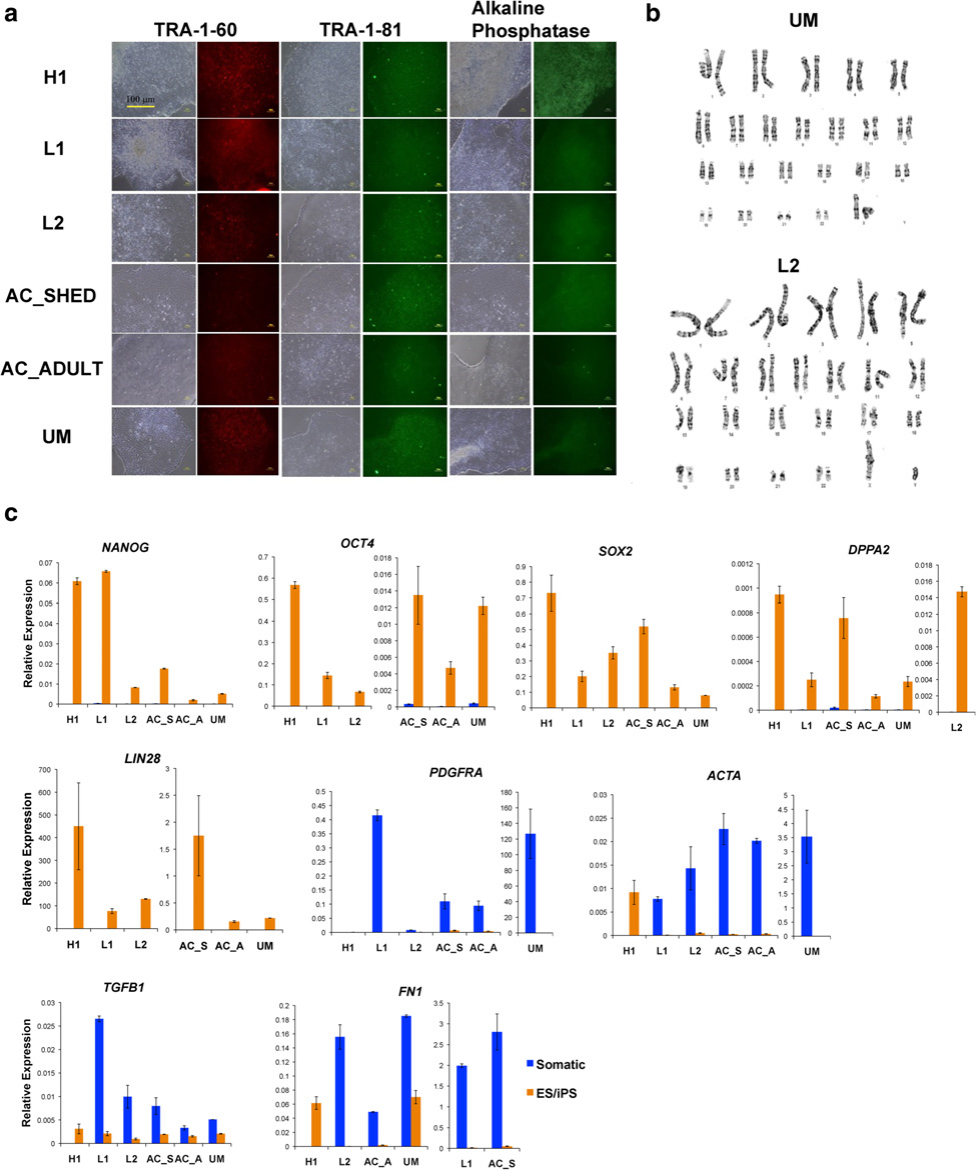
Pluripotent characterisation of clinically compatible DPSC-derived iPS cells using virus-free, xeno-free, and feeder-free approaches. **a** Immunofluorescence results indicating that H1 hES and five DiPS cell lines express pluripotency markers TRA-1-60, TRA-1-81, and alkaline phosphatase. **b** Karyotyping results of two representative DiPS lines. **c** Reverse transcription qPCR analysis results showing the expression levels of pluripotency-related genes and mesenchymal genes of the H1 hES and DiPS cells relative to their isogeneic somatic cells. Note that H1 does not have ‘somatic’ data

To test the pluripotency of the xeno-free DiPS lines, live cell immuno-staining was performed using standard pluripotent markers such as TRA-1-60, TRA-1-81, and alkaline phosphatase. The results indicated that the DiPS cells showed expression of these markers comparable to the H1 hES line which were also maintained in xeno-free media (Fig. 2a). We also performed cytogenetic analysis by karyotyping, and results revealed normal karyotypes in DiPS lines (Fig. 2b and Additional file 1: Figure S5a).

Next, reverse-transcription qPCR analysis was performed to show the mRNA expression of endogenous pluripotency genes such as *NANOG*, *OCT4*, *SOX2, DPPA2*, and *LIN28*, and endogenous somatic genes such as *ACTA*, *PDGFRA*, *TGFB1*, and *FN1* in all five DiPS cell lines in comparison to their isogenic parental line and H1. As expected, pluripotent genes were significantly up-regulated and somatic genes were significantly down-regulated in all DiPS lines (Fig. 2c) when compared with their somatic cells of origin, further establishing their pluripotency. Some variations in gene expressions across cell lines were observed, consistent with earlier reports that donor-specific heterogeneities in the genetic and non-genetic background can introduce such variability in iPS cells [33, 34].

**Fig. 3:**
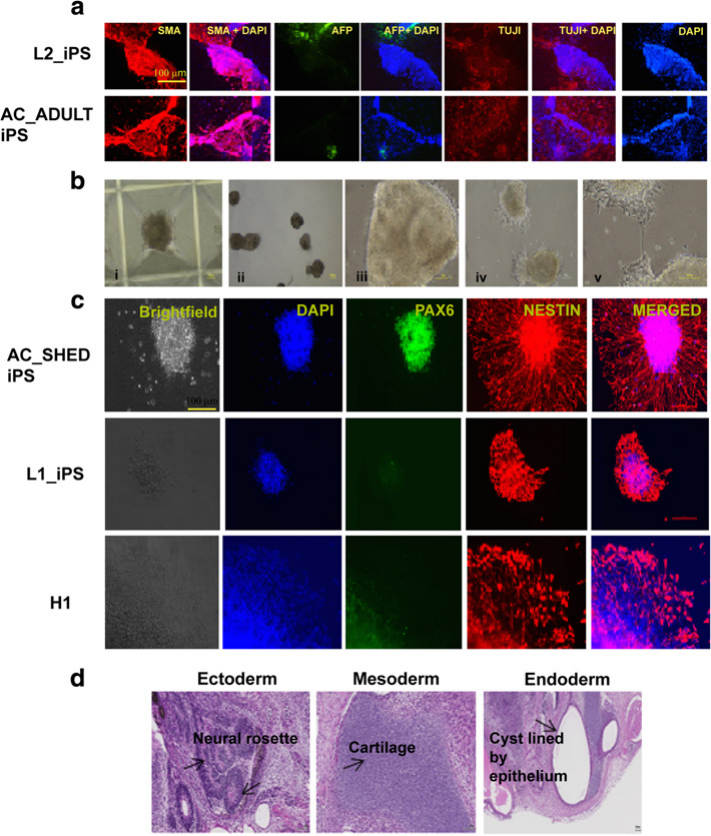
Xeno-free DiPS cells can be spontaneously differentiated into all three germ layers under in vitro and in vivo conditions. **a** Three germ layer immunostaining markers of EBs, ectoderm (beta-III tubulin (TUJ1)), mesoderm (smooth muscle actin (SMA)), and endoderm (alpha-fetoprotein (AFP)). **b** DiPS-derived EBs differentiated to neural rosettes and further morphological changes of adherent neurospheres with cell extensions in xeno-free media. (i) EBs generated from AC_SHED iPS cells in AggreWell™800 plates. (ii) On day 5, EBs were harvested and re-plated on matrix (poly-l-ornithine/laminin)-coated culture plates. (iii) Neural rosette formation occurs from day 9–11. (iv) Neural rosettes formed were selected and re-plated on matrix (poly-l-ornithine/laminin)-coated culture plates on day 12. (v) Attached neural rosette-containing clusters start forming neural progenitor cell outgrowths. **c** Expression of neural markers Nestin and PAX6 by immunofluorescence for neural progenitor cells differentiated from xeno-free grown DiPS and ES lines. **d** H&E staining of teratoma derived from AC_SHED iPS cells. The teratoma contains structures of all three germ layers (as shown by the arrows)

For in vitro differentiation assays, embryoid bodies (EBs) were generated from DiPS cells (Additional file 1: Figure S5c) and, at day 7 onwards, the EBs were allowed to differentiate spontaneously. The authenticity of differentiation was assessed by three germ layer immunostaining markers of EBs: ectodermal (beta-III tubulin (TUJ1)), mesodermal (smooth muscle actin (SMA)), and endodermal (alpha-fetoprotein (AFP)) markers, as shown in Fig. 3a.

Concurrently, in vivo differentiation assay was performed by examining teratoma formation in DiPS cell lines that were injected into the subcutaneous area above the thigh (quadriceps) muscle of immune-compromised SCID mice. We confirmed the formation of teratoma and identified the presence of structures from all three germ layers, observed by H&E staining of the teratoma section (Fig. 3d and Additional file 1: Figure S5b).

### Differentiation into neural progenitor cells

DPSCs have a neuro-ectoderm origin and are expected to retain epigenetic memory of neuronal origins. The inherent potential of DiPS cells to differentiate towards the neuronal lineage was demonstrated through formation of neural spheres and later towards neuronal progenitor cells as shown in Fig. 3b. We used the H1 (ES) cell line as a control along with L1 and AC_SHED iPS lines to examine the effect of neuronal differentiation potentials under xeno-free culture conditions. We attempted neural progenitor formation using the DiPS lines (L1 and AC_SHED) in comparison to H1 cells, which were also maintained under the xeno-free culture condition. The neural progenitor cells were immunostained with the neural markers PAX6 and Nestin. As shown in Fig. 3c, the DiPS lines generated using xeno-free methods exhibited positive staining for PAX6 and Nestin.

### Genome-wide DNA methylation profiling of DiPS in comparison to AiPS cells

To delineate epigenetic changes during reprogramming of DPSCs, DiPS as well as AiPS cells derived from the episomal-based reprogramming method were subjected to genome-wide DNA methylation analysis in comparison to their somatic origins and hES cells.

**Fig. 4:**
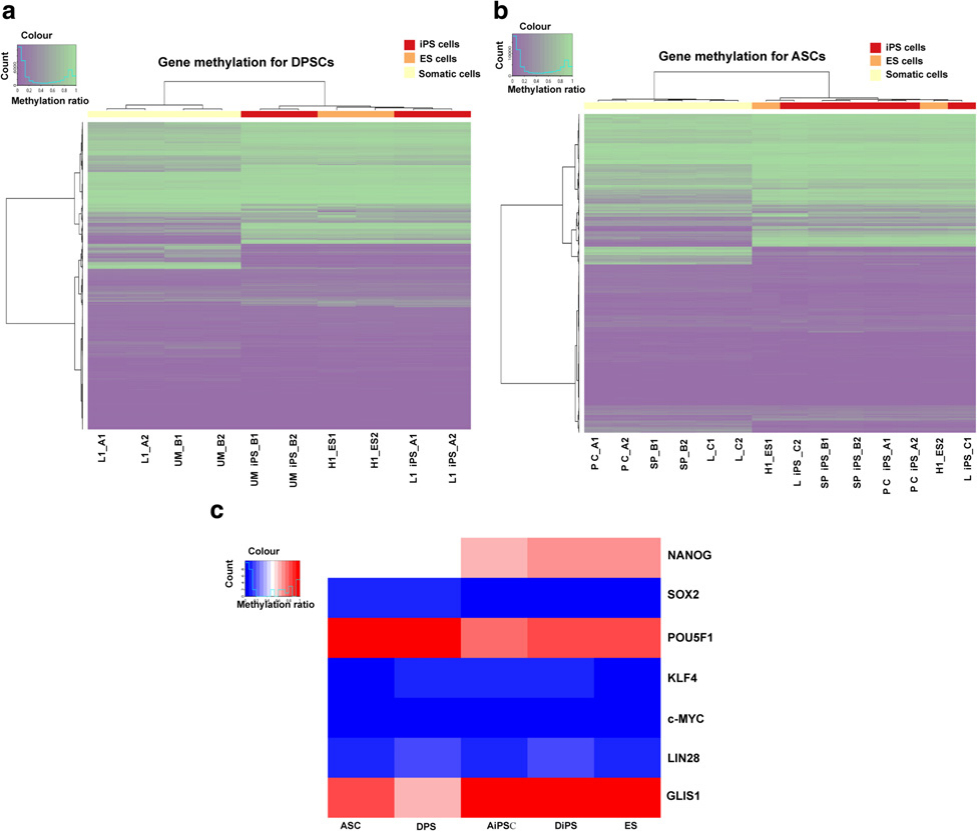
DNA methylation profiling of DiPS and AiPS cell lines in comparison to their parental cells. **a** Heatmap of the methylation ratio (beta-value) of global probe sets for genes in the dental-derived somatic parental (L1, UM), DiPS (L1 iPS, UM iPS), and H1 ES cells. Duplicates for each cell line. **b** Heatmap of the methylation ratio (beta-value) of global probe sets for genes in the adipose-derived somatic parental (PC, SP, L), AiPS (PC iPS, SP iPS, L iPS), and H1 hES cells. Duplicates for each cell line. **c** Heatmap representing classic pluripotent genes in parental DPSCs and ASCs with respect to their resultant iPS lines and H1 hES cells. The methylation ratio is averaged across all probes for each gene and across all samples in the same group. AiPS adipose-derived stem cell-derived induced pluripotent stem, ASC adipose-derived stem cell, DiPS dental pulp stem cell-derived induced pluripotent stem, DPSC dental pulp stem cell, ES embryonic stem, iPS induced pluripotent stem

To compare DNA methylation levels between parental somatic (DPSCs and ASCs) and iPS cells, with ES (H1 line) as a reference, unsupervised hierarchical clustering of the methylation ratio (beta-values) was performed to identify grouping of samples (Fig. 4a, b). The ES and iPS (DiPS and AiPS) cells appeared indistinguishable from each other and formed a tight cluster, well separated from the other two clusters of the parental somatic cells, DPSCs and ASCs (Fig. 4a, b). This result shows that the DNA methylomes of iPS lines closely resemble those of ES cells and yet are very distinct from their parental DPSC/ASC lines. DNA methylation patterns of classical pluripotent genes (*SOX2*, *OCT4* (also known as *POU5F1*), *KLF4*, *C-MYC*, *LIN28*, *GLIS1*), however, were relatively unchanged in the whole cell samples, except for *NANOG* (Fig. 4c).

**Fig. 5:**
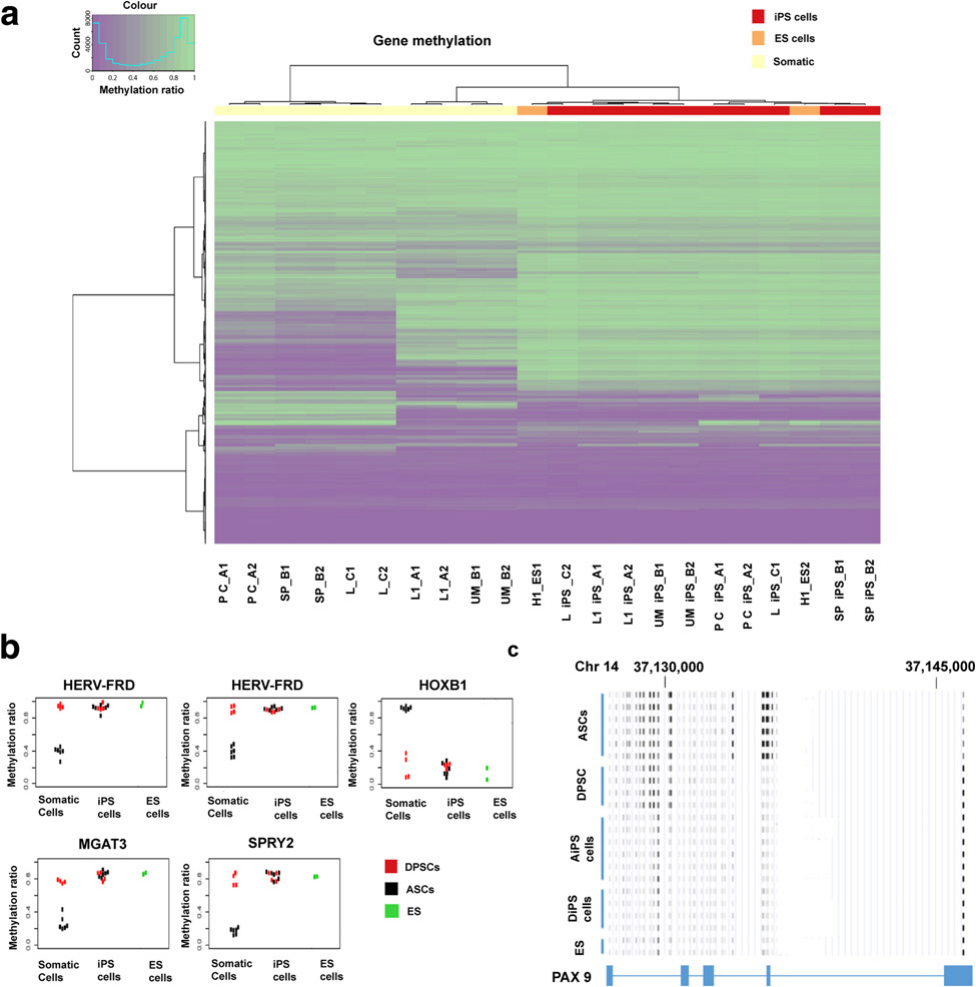
Proximity of DNA methylation markers in DPSCs to hES and iPS cells. **a** Heatmap of the methylation ratio of probes for selected genes involved in pluripotency, development, endoderm, mesoderm, and ectomesenchyme in the dental- and adipose-derived somatic parental cell lines, DiPS, AiPS, and ES cells. Duplicates for each cell line. **b** Methylation levels of five selected developmentally associated genes for dental- and adipose-derived somatic cells with respect to iPS cells (DiPS and AiPS) and H1 hES. The probe sets for each gene analysed are: HER-FRD = cg25106036; HER-FRD = cg214999175; HOXB1 = cg24948406; MGAT3 = cg00101350; SPRY2 = cg00185066. See Additional file 1 (Table S3) for original data. **c** Gene model of *PAX9* showing methylation levels (black being completely methylated and white completely unmethylated) at different probe sets. DPSCs show a highly similar epigenetic profile to ES and iPS cells at exons 4 and 5, but not ASCs. See Additional file 1: Table S4 for original probe sets data. AiPS adipose-derived stem cell-derived induced pluripotent stem, ASC adipose-derived stem cell, DiPS dental pulp stem cell-derived induced pluripotent stem, DPSC dental pulp stem cell, ES embryonic stem, iPS induced pluripotent stem

Interestingly, DPSCs showed DNA methylation profiles closer to pluripotent cell (ES and iPS) populations (Fig. 5a), compared with ASCs, in genes involved in development, pluripotency, endoderm, mesoderm, and ectomesenchyme. This explains the high efficiency of reprogramming of DPSCs when compared with ASCs under the same culture conditions. Further delineation of DNA methylation profiles led to identification of certain pluripotent and development-associated genes (*HERV-FRD*, *MGAT3*, *SPRY2*, *HOXB1*, and *PAX9*) epitomizing the reprogramming dynamic of DPSCs (Fig. 5b, c, Additional file 1: Table S3 and Table S4). These five selected pluripotent-associated genes for DPSCs contained regions of methylation levels on a par with iPS cells (DiPS and AiPS) and H1 hES cells. In particular, the gene locus of *PAX9* in DPSCs shows a highly similar epigenetic profile to the iPS/ES cells, especially at exons 4 and 5, which was not the case for ASCs. Other differentially methylated genes of interest (> 80 genes) in DPSCs versus ASCs in comparison with their respective iPS and H1 ES cell lines are listed in Additional file 1: Table S3. Additionally, results from IPA analysis demonstrates the set of genes that undergo significant epigenetic changes when transforming from the somatic cell state (DPSCs/ASCs) into the iPS (DiPS/AiPS) cell state as depicted in Additional file 1: Table S5. Notable epigenetic changes were undertaken in ASCs in comparison with DPSCs after reprogramming into iPS cells, unravelling the significance of the starting cell population for iPS generation. We found that among the top enriched networks of the genes in IPA, analysis includes “cellular development, cellular growth and proliferation, connective tissue development and function,” and “gene expression, cell morphology, cellular assembly, and organisation.” Altogether our results indicate that DPSCs exhibit epigenetic signatures of many developmental genes that are closer to those of pluripotent stem cells, at least partially accounting for their favourable reprogramming capability.

### Overexpression of PAX9 and knockdown HERV-FRD improve reprogramming

**Fig. 6:**
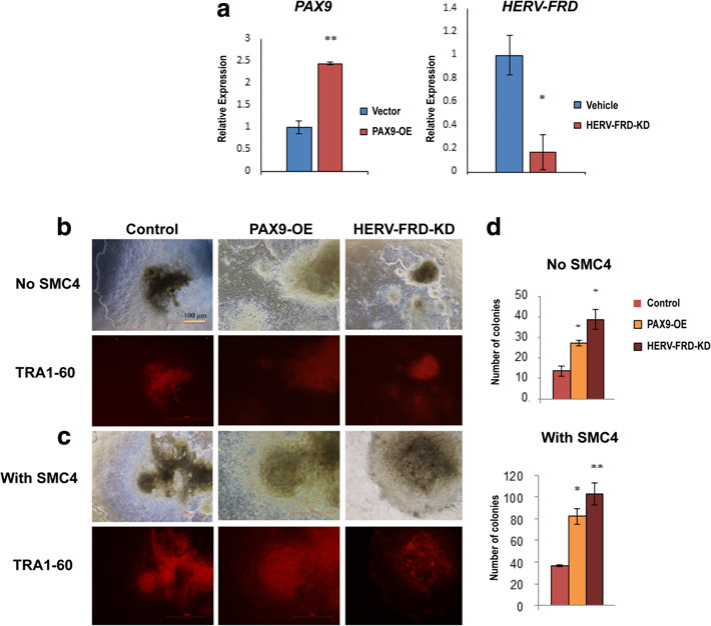
Effects of *PAX9* and *HERV-FRD* modulations in iPS reprogramming. **a** Reverse transcription qPCR analysis results showing the over-expression (OE) levels of *PAX9* and knock-down (KD) level of *HERV-FRD* genes in the DPSC (L1) line. **b** Immunofluorescence results of TRA-1-60 expression for iPS clones generated without SMC4 cocktail in control, PAX9 over-expressing, and HERV-FRD knock-down DPSC (L1) lines. **c** Immunofluorescence results of TRA-1-60 expression for iPS clones generated with SMC4 cocktail in control, PAX9 over-expressing, and HERV-FRD knock-down DPSC (L1) lines. **d** Number of iPS colonies counted from control, PAX9 over-expressing, and HERV-FRD knock-down DPSC (L1) lines reprogrammed with and without SMC4 cocktail

Among the differentially methylated genes, we picked *PAX9* and *HERV-FRD* to further investigate their roles in reprogramming. As shown in Fig. 5c, *PAX9* contains various regions in DPSCs that are hypomethylated compared with ASCs and that are similar to those in iPS/ES cells. In contrast, *HERV-FRD* includes at least two hypermethylated sites in DPSCs that are similar to those in iPS/ES cells, which is hypomethylated in ASCs (Fig. 5b). We transiently over-expressed PAX9 and knocked-down HERV-FRD in DPSCs, and examined their effects on reprogramming. As expected, after inducing the over-expression (OE) of PAX9 and knock-down (KD) of HERV-FRD, the transcript level of *PAX9* was up-regulated while that of *HERV-FRD* was down-regulated (Fig. 6a). Furthermore, the iPS colonies that were generated from these PAX9 OE and HERV-FRD KD cell lines and their reprogramming efficiency was assessed in the presence and absence of SMC4 (Fig. 6b, c). Both PAX9 OE and HERV-FRD KD cell lines exhibited increased iPS colony number compared with mock transfected control cells (Fig. 6d).

### Presence of DPSC subpopulation that exhibits more pluripotent and self-renewal supporting capacities

Our epigenetic study indicates that DPSCs already have higher pluripotent potential than ASCs, even without reprogramming factors. During DPSC culture we noted the occurrence of a peculiar scanty clonal structure within the normal fibroblast-like DPSCs. Due to its resemblance to a compact iPS/ES colony-like structure, we performed standard pluripotent cell live staining using TRA-1-60, SSEA-3, and Connexin-43 on normal DPSC culture. Connexin-43, a gap junction protein, is also significantly involved in embryonic development [35]. Its involvement in the course of reprogramming is also reported through regulation of E-cadherin, which later mediates mesenchymal-to-epithelial transition (MET) [35, 36]. To our surprise, these DPSC-derived satellite-like structures sporadically displayed pluripotent surface marker expressions for TRA-1-60, SSEA-3, and Connexin-43, which may account for the higher propensity of DPSCs for iPS generation (Additional file 1: Figure S6a).

In addition, it was previously demonstrated that feeder-free reprogramming is possible with ASCs since ASCs themselves possess an ability to support self-renewal of other pluripotent stem cells [9]. We thus evaluated the suitability of DPSCs as a feeder layer in comparison with ASCs and MEF to support pluripotency of H1 hES cells, and found that DPSCs also have a comparable capacity as feeders (Additional file 1: Figure S6b). In our study, both DPSC- and ASC-derived feeder layers supported the H1 cell maintenance. However, a certain degree of variation in expression of pluripotent genes was observed. H1 cells supported by ASCs exhibited higher SSEA4 expression than those supported by DPSC feeder layers. On the other hand, DPSCs were slightly superior in terms of supporting TRA-1-81 and TRA-1-60 expression in H1 cells compared with MEF and ASC feeder layers (Additional file 1: Figure S6b). Therefore, even though all three feeder layers seem to be more or less supportive of H1 cell self-renewal, DPSCs may maintain expression of authentic pluripotent markers such as TRA-1-81 and TRA-1-60 better than ASCs and even MEF. Our observation indicates the potential of DPSCs as human-derived cell feeders as well as the cell source for iPS reprogramming, which further justifies their suitability for clinical use.

## Discussion

Although iPS cells can be routinely generated for many purposes, there is still no consent on the best reprogramming method for clinical applications as this may be highly affected by pre-disposed cellular factors including their maturity, complexity, and origin. This variance in reprogramming efficiencies is apparent when different cell populations from different tissues are used [6, 12]. In our findings, human DPSCs can be easily reprogrammed into iPS cells with the conventional viral approach. Importantly, the same efficacy was consistently obtained with the episomal mode of reprogramming for DPSCs, which has the advantage of clinical safety by avoiding potential viral gene integration into the cells. In a clinical perspective, standard operation procedures using iPS cells would highly favour those that involve manageable tissue sources, with minimal patient discomfort during harvesting, ideally from low-cost biological waste, short-time frame, and minimal processing cost and complications. DPSCs are advantageous in these aspects. The presence of certain cellular signalling factors may play a significant role in the favourable fate determinant of DPSCs. Families of such factors may include Wnt, Notch, and BMP, which are known to regulate the fate of cells by mediating the molecular cross-talk [37]. Notably, the TGF-β/activin pathway was reported to maintain the pluripotent state, whereas the p53 pathway acts as a barrier for cell reprogramming [38]. Interestingly, the positive reprogramming process of DPSCs can be at least partially explained by its reduced level of p53 molecules accompanied by abundant expression of all the members of TGF-β signalling molecules [21]. In addition, we found that at least some populations of DPSCs have high intrinsic expression for pluripotent markers such as SSEA3, Tra-1-60, and Connexin-43, which suggests a lower barrier to undergoing reprogramming into pluripotency.

With the conventional method it takes approximately 30 days to derive human iPS cells with a reprogramming success rate of approximately 0.01% using fibroblasts, as reported earlier [1]. The reprogramming rate has been progressively improved using different cell lines and implementing various modes of vector systems. For instance, the rate was improved to 0.2% or higher using ASCs [22, 31]. Later, and with the advent of more clinically acceptable methods such as integration-free episomal plasmids, human iPS generation efficiency was further improved by 0.5% as reported in human gingival tissue [39]. However, the reprogramming procedure may still take as long as for fibroblasts, and may not be robustly reproducible under clinically compatible conditions. In this paper, we made efforts to further improve the clinically applicable reprogramming protocol by successfully generating virus-free DiPS cells using episomal plasmids in a more refined manner using a small molecule cocktail of four inhibitors (SMC4 cocktail) and under the xeno-free culture condition. This protocol was relatively quick (less than 18 days in colony formation), handy, reproducible, and capable of inducing feeder-free pluripotency of primary DPSCs from five different donors. All the five DiPS lines generated in our laboratory had standard pluripotent characteristics equivalent to ES cells, which include the expression of pluripotent surface markers using immunostaining and reverse-transcription qPCR, spontaneous in vitro differentiation, and in vivo differentiation potential through teratoma formation and karyotyping analysis. Unexpectedly the reprogramming efficiency of DPSCs using episomal vectors under feeder-free and xeno-free conditions were higher (0.19%) than the conventional viral methods using feeder-free layers (0.05%), thereby generating higher numbers of iPS clones. This is possibly because of the stochastic mode of reprogramming as previously suggested by Yamanaka [40], where cells could be reprogrammed in the right direction only with the right amount and balance of different reprogramming factor expressions. This subtle balance may be influenced by the mode of transfection affecting expression of the reprogramming transgenes [41].

In addition, DPSCs were found to exhibit a higher feeder-free reprogramming capability. The feeder independence of human cell sources was previously noted for ASCs because this cell type can support self-renewal of other pluripotent stem cells when used as a feeder layer [22]. We showed that DPSCs exhibit comparable or even higher feeder-free reprogramming efficiency and support pluripotency of human ES cells when DPSCs themselves were used as feeders. This indicates the potential clinical use of DPSCs as an alternative human-derived feeder source. Furthermore, we optimised the episomal-based xeno-free protocol for DiPS generation and showed that DiPS cells can be derived in less than 14 days. This was achieved by adopting a cocktail complex of SMC4 consisting of SB431542 (TGFβi), PD0325901 (MEKi), CHIR99021 (GSK3i), and Thiazovivin (ROCKi), which was shown to improve reprogramming efficiency as described by Valamehr et al. [42]. Combination of these four small molecules resulted in high cell viability, survival, high clonality (colony compactness), and enrichment of homogeneous iPS clones from reprogrammed DPSCs. Thus, we believe that the use of SMC4 is highly suitable for industrial and clinical applications.

Although DPSCs exhibit a higher capability to become iPS cells, very little has been studied regarding their epigenetic basis. In our studies, we investigated epigenetic mechanisms of the favourable reprogramming propensity of DPSCs. Our global DNA methylation analysis indicates that DPSCs, which express considerable levels of pluripotency-associated factors even without reprogramming, also exhibit various methylation marks predisposed for pluripotency compared with ASCs. The candidate genes *HERV-FRD*, *SPRY2*, and *MGAT3* were highly methylated and *PAX9* and *HOXB1* relatively unmethylated in DPSCs, levels similar to ES and iPS cells as shown in Fig. 5. For instance, the paired box 9 (*PAX9*) homeobox gene is well known for its critical role during development in orchestrating growth factors, developmental cytokines, adhesion molecules, and its complex interactions in embryonic- as well as neural crest-derived tissues [43, 44]. *HERV-W*, also known as Syncytin-1 and an isoform of *HERV-FR*D, was reported to be a specific marker of the human trophoblast [45]. A very recent report confirmed the expression of Syncytin-1 to be mainly associated with trophoblast cells of the blastocyst particularly in cells underlying the inner cell mass [46]. Interestingly, Syncytin-1 was previously identified in membrane proteins involved directly in trophoblastic cell adhesion molecules along with Connexin-43 and ZO-1 [47, 48]. This may be in accordance with our experimental observation of Connexin-43 expression during DPSC culture. In contrast, the role of HERV-FRD/Syncytin-2 is enigmatic, but one piece of evidence suggests that it is also expressed in human trophoblasts and plays an important role in trophoblast cell fusion [49]. Based on the DNA methylation results, we selected *PAX9* and *HERV-FRD* genes due to the presence of multiple differentially methylated regions that are equivalent to iPS/ES cells and examined their effects. Accordingly, PAX9 was over-expressed (PAX9-OE) and HERV-FRD was knocked-down (HERV-FRD-KD) in DPSCs for evaluation of the reprogramming propensity. Consistent with expectation, increases in generation of iPS colonies were observed in both cell lines, indicating that *PAX9* serves as an enhancing factor and *HERV-FRD* as a suppressing factor for iPS reprogramming. An important question remaining to be answered is how the developmental *PAX9* and *HERV-FRD* genes regulate the reprogramming process, and this warrants additional study. Although the prime determinants and effects of epigenetic changes during induced pluripotency are not fully understood, it is clearly perceived that dynamic changes in epigenetic signatures from the somatic state into the pluripotent state are needed to acquire pluripotency [50]. Our results indicate that DPSCs are advantageous since the cells already possess certain degrees of epigenetic marker similarity to the iPS state, especially regarding developmental genes presumed to play important roles in regulating pluripotency.

The use of DPSCs as a source for iPS cells may have additional advantages for cell replacement therapy or modelling disease physiology because of their epigenetic inclination toward certain cell lineage. For example, DPSCs are in the neural crest origin and thus can be more inclined towards differentiation to the neural cell types [51]. We hypothesise that epigenetic memory of the neural crest origin may remain and successfully differentiate DiPS cells, but not AiPS cells, to neural progenitor-like cells in vitro. Future work is necessary to develop further directed differentiation protocols to derive DiPS cells into various functional cell types. In addition, while the xeno-free reprogramming protocol was established in this paper, development of a suitable xeno-free isolation and initial culture of DPSCs may still be necessary for optimal clinical applications. Collectively, the epigenetic advantage of DPSCs as the starting cell material for generating fast, efficient, and high-quality iPS cells and their differentiated cells can be fully utilised in regenerative medicine if further advances in clinically compatible protocols are made.

## Conclusion

To develop safe and efficient iPS generation methods towards clinical applications, we established non-integrative episomal-based reprogramming of DPSCs in the feeder-free and xeno-free culture condition. Importantly, this xeno-free episomal method can more efficiently reprogramme DPSCs compared with the conventional viral method. Further refinement of this procedure by including the SMC4 cocktail resulted in both a shorter time (< 14 days) and higher quality of the reprogramming process. DPSCs may be more pre-disposed to feeder/xeno-free reprogramming because they express relatively high levels of pluripotent markers and can serve themselves as a feeder layer for human pluripotent stem cells. In addition, DPSCs exhibit epigenetic DNA methylation marks of certain development-associated genes in relative proximity to those of human ES and iPS cells. Among these genes, it was found that PAX9 would enhance and HERV-FRD/Syncytin-2 repress reprogramming into iPS cells. These combined properties of DPSCs may make them amenable for iPS cell research and subsequent clinical applications. Further work is necessary for more in-depth understanding of the molecular regulatory mechanisms of DPSC reprogramming and the development of DiPS-based novel therapies in the regenerative arena.

## Additional file

Additional file 1: **Table S1.** List of commercial and patient-derived dental stem cell samples used. **Table S2.** DiPS colony counts of dental pulp stem cell samples used for reprogramming under different culture conditions. **Figure S1.** Transduction efficiencies for reprogramming DPSCs. **Figure S2.** Generation of iPS cells from DPSCs in the presence of enhancer compounds. **Figure S3.** Time course during episomal-based reprogramming of DPSCs. **Figure S4.** Characterisation of DPSCs cultured in xeno-free media. **Figure S5.** Cytogenetics and in vivo characterisation of DiPS lines generated under xeno-free culture conditions. **Table S3.** List of differentially methylated regions of selected genes in DPSCs versus ASCs with respect to iPS (DiPS and AiPS) and H1 hES cell lines. **Table S4.** DNA methylation raw data analysed with multiple probe sets of *PAX9* gene that exhibit significant differences between DPSCs and ASCs. **Table S5.** Top networks by ingenuity pathway analysis (IPA) for differentially methylated genes in ASCs versus AiPS cells that do not exhibit such differences in DPSCs versus DiPS cells. **Figure S6.** Pluripotent and self-renewal supporting characteristics of DPSCs. (ZIP 8876 kb)

## Abbreviations

AiPS: Adipose-derived stem cell-derived induced pluripotent stem
ANOVA: Analysis of variance
ASC: Adipose-derived stem cell
bFGF: Basic fibroblast growth factor
cDNA: Complementary DNA
d: Days post-transduction
DiPS: Dental pulp stem cell-derived induced pluripotent stem
DPSC: Dental pulp stem cell
EB: Embryoid body
ECM: Extracellular matrix
ES: Embryonic stem
FBS: Fetal bovine serum
GFP: Green fluorescent protein
H&E: Haematoxylin and eosin
IPA: Ingenuity pathway analysis
iPS: Induced pluripotent stem
KD: Knock-down
KO-DMEM: Knockout Dulbecco’s modified Eagle’s medium
MET: Mesenchymal-to-epithelial transition
MSC: Mesenchymal stem cell
NEAA: Non-essential amino acids
NPC: Neural progenitor cell
OE: Over-expression
P/S: Penicillin/streptomycin
PBS: Phosphate-buffered saline
qPCT: Quantitative polymerase chain reaction
TGF: Transforming growth factor

## References

[1] Takahashi K, Tanabe K, Ohnuki M, Narita M, Ichisaka T, Tomoda K, Yamanaka S (2007). Induction of pluripotent stem cells from adult human fibroblasts by defined factors. Cell..

[2] Shi Y, Inoue H, Wu JC, Yamanaka S (2017). Induced pluripotent stem cell technology: a decade of progress. Nat Rev Drug Discov..

[3] Okano H, Nakamura M, Yoshida K, Okada Y, Tsuji O, Nori S, Ikeda E, Yamanaka S, Miura K (2013). Steps toward safe cell therapy using induced pluripotent stem cells. Circ Res..

[4] Srijaya TC, Pradeep PJ, Zain RB, Musa S, Abu Kasim NH, Govindasamy V (2012). The promise of human induced pluripotent stem cells in dental research. Stem Cells Int..

[5] Trounson A, DeWitt ND (2016). Pluripotent stem cells progressing to the clinic. Nat Rev Mol Cell Biol..

[6] Goh PA, Caxaria S, Casper C, Rosales C, Warner TT, Coffey PJ, Nathwani AC (2013). A systematic evaluation of integration free reprogramming methods for deriving clinically relevant patient specific induced pluripotent stem (iPS) cells. PLoS One..

[7] Silva M, Daheron L, Hurley H, Bure K, Barker R, Carr AJ, Williams D, Kim HW, French A, Coffey PJ (2015). Generating iPSCs: translating cell reprogramming science into scalable and robust biomanufacturing strategies. Cell Stem Cell..

[8] Rodriguez-Piza I, Richaud-Patin Y, Vassena R, Gonzalez F, Barrero MJ, Veiga A, Raya A, Izpisua Belmonte JC (2010). Reprogramming of human fibroblasts to induced pluripotent stem cells under xeno-free conditions. Stem Cells..

[9] Sugii S, Kida Y, Berggren WT, Evans RM (2011). Feeder-independent iPS cell derivation from human and mouse adipose stem cells. Nature Protoc..

[10] Wang J, Hao J, Bai D, Gu Q, Han W, Wang L, Tan Y, Li X, Xue K, Han P (2015). Generation of clinical-grade human induced pluripotent stem cells in xeno-free conditions. Stem Cell Res Ther..

[11] MacArthur CC, Fontes A, Ravinder N, Kuninger D, Kaur J, Bailey M, Taliana A, Vemuri M, Lieu P (2012). Generation of human-induced pluripotent stem cells by a nonintegrating RNA Sendai virus vector in feeder-free or xeno-free conditions. Stem Cells Int..

[12] Gonzalez F, Boue S, Izpisua Belmonte JC (2011). Methods for making induced pluripotent stem cells: reprogramming a la carte. Nat Rev Genet..

[13] Srijaya TC, Sriram S, Sugii S, NHA K, Pham PV (2016). Stem cells in dentistry: potential applications and perspectives in clinical research. Bone and cartilage regeneration.

[14] Kashyap R (2015). SHED—basic structure for stem cell research. J Clin Diagn Res..

[15] Huang GT, Gronthos S, Shi S (2009). Mesenchymal stem cells derived from dental tissues vs. those from other sources: their biology and role in regenerative medicine. J Dent Res..

[16] Jiang L, Peng WW, Li LF, Yang Y, Zhu YQ (2012). Proliferation and multilineage potential of CXCR4-positive human dental pulp cells in vitro. J Endod..

[17] Karamzadeh R, Eslaminejad MB, Andrades JA (2013). Dental-related stem cells and their potential in regenerative medicine in regenerative medicine. INTECH, ISBN tissue engineering and regenerative medicine.

[18] Leprince JG, Zeitlin BD, Tolar M, Peters OA (2012). Interactions between immune system and mesenchymal stem cells in dental pulp and periapical tissues. Int Endod J..

[19] Liu J, Yu F, Sun Y, Jiang B, Zhang W, Yang J, Xu GT, Liang A, Liu S (2015). Concise reviews: characteristics and potential applications of human dental tissue-derived mesenchymal stem cells. Stem Cells..

[20] Tamaoki N, Takahashi K, Tanaka T, Ichisaka T, Aoki H, Takeda-Kawaguchi T, Iida K, Kunisada T, Shibata T, Yamanaka S, Tezuka K (2010). Dental pulp cells for induced pluripotent stem cell banking. J Dent Res..

[21] Yildirim S (2013). Dental pulp stem cells.

[22] Sugii S, Kida Y, Kawamura T, Suzuki J, Vassena R, Yin YQ, Lutz MK, Berggren WT, Izpisua Belmonte JC, Evans RM (2010). Human and mouse adipose-derived cells support feeder-independent induction of pluripotent stem cells. Proc Natl Acad Sci U S A..

[23] Ong WK, Tan CS, Chan KL, Goesantoso GG, Chan XH, Chan E, Yin J, Yeo CR, Khoo CM, So JB (2014). Identification of specific cell-surface markers of adipose-derived stem cells from subcutaneous and visceral fat depots. Stem Cell Reports..

[24] Takeda K, Sriram S, Chan XH, Ong WK, Yeo CR, Tan B, Lee SA, Kong KV, Hoon S, Jiang H (2016). Retinoic acid mediates visceral-specific adipogenic defects of human adipose-derived stem cells. Diabetes..

[25] Khanna-Jain R, Mannerstrom B, Vuorinen A, Sandor GK, Suuronen R, Miettinen S (2012). Osteogenic differentiation of human dental pulp stem cells on beta-tricalcium phosphate/poly (l-lactic acid/caprolactone) three-dimensional scaffolds. J Tissue Eng..

[26] Suchanek J, Suchankova Kleplova T, Rehacek V, Browne KZ, Soukup T (2016). Proliferative capacity and phenotypical alteration of multipotent ecto-mesenchymal stem cells from human exfoliated deciduous teeth cultured in xenogeneic and allogeneic media. Folia Biol (Praha).

[27] Takahashi K, Okita K, Nakagawa M, Yamanaka S (2007). Induction of pluripotent stem cells from fibroblast cultures. Nat Protoc..

[28] Okita K, Matsumura Y, Sato Y, Okada A, Morizane A, Okamoto S, Hong H, Nakagawa M, Tanabe K, Tezuka K (2011). A more efficient method to generate integration-free human iPS cells. Nat Methods..

[29] Kurosawa H (2007). Methods for inducing embryoid body formation: in vitro differentiation system of embryonic stem cells. J Biosci Bioeng..

[30] Aryee MJ, Jaffe AE, Corrada-Bravo H, Ladd-Acosta C, Feinberg AP, Hansen KD, Irizarry RA (2014). Minfi: a flexible and comprehensive bioconductor package for the analysis of infinium DNA methylation microarrays. Bioinformatics..

[31] Sun N, Panetta NJ, Gupta DM, Wilson KD, Lee A, Jia F, Hu S, Cherry AM, Robbins RC, Longaker MT, Wu JC (2009). Feeder-free derivation of induced pluripotent stem cells from adult human adipose stem cells. Proc Natl Acad Sci U S A..

[32] Lin T, Ambasudhan R, Yuan X, Li W, Hilcove S, Abujarour R, Lin X, Hahm HS, Hao E, Hayek A, Ding S (2009). A chemical platform for improved induction of human iPSCs. Nat Methods..

[33] Carcamo-Orive I, Hoffman GE, Cundiff P, Beckmann ND, D'Souza SL, Knowles JW, Patel A, Papatsenko D, Abbasi F, Reaven GM (2017). Analysis of transcriptional variability in a large human iPSC library reveals genetic and non-genetic determinants of heterogeneity. Cell Stem Cell..

[34] Rouhani F, Kumasaka N, de Brito MC, Bradley A, Vallier L, Gaffney D (2014). Genetic background drives transcriptional variation in human induced pluripotent stem cells. PLoS Genet..

[35] Ke Q, Li L, Cai B, Liu C, Yang Y, Gao Y, Huang W, Yuan X, Wang T, Zhang Q (2013). Connexin 43 is involved in the generation of human-induced pluripotent stem cells. Hum Mol Genet..

[36] Li R, Liang J, Ni S, Zhou T, Qing X, Li H, He W, Chen J, Li F, Zhuang Q (2010). A mesenchymal-to-epithelial transition initiates and is required for the nuclear reprogramming of mouse fibroblasts. Cell Stem Cell..

[37] Mitsiadis TA, Graf D (2009). Cell fate determination during tooth development and regeneration. Birth Defects Res C Embryo Today..

[38] Feng B, Ng JH, Heng JC, Ng HH (2009). Molecules that promote or enhance reprogramming of somatic cells to induced pluripotent stem cells. Cell Stem Cell..

[39] Umezaki Y, Hashimoto Y, Nishishita N, Kawamata S, Baba S (2015). Human gingival integration-free iPSCs: a source for MSC-like cells. Int J Mol Sci..

[40] Yamanaka S (2009). Elite and stochastic models for induced pluripotent stem cell generation. Nature..

[41] Huang GTJ (2010). Induced pluripotent stem cells—a new foundation in medicine. J Exp Clin Med..

[42] Valamehr B, Abujarour R, Robinson M, Le T, Robbins D, Shoemaker D, Flynn P (2012). A novel platform to enable the high-throughput derivation and characterization of feeder-free human iPSCs. Sci Rep..

[43] de Almeida CV, de Andrade SC, Saito CPB, Ramenzoni LL, Line SRP (2010). Transcriptional analysis of the human PAX9 promoter. J Appl Oral Sci..

[44] Monsoro-Burq AH (2015). PAX transcription factors in neural crest development. Semin Cell Dev Biol..

[45] Malassine A, Handschuh K, Tsatsaris V, Gerbaud P, Cheynet V, Oriol G, Mallet F, Evain-Brion D (2005). Expression of HERV-W Env glycoprotein (syncytin) in the extravillous trophoblast of first trimester human placenta. Placenta..

[46] Soygur B, Moore H (2016). Expression of Syncytin 1 (HERV-W), in the preimplantation human blastocyst, embryonic stem cells and trophoblast cells derived in vitro. Hum Reprod..

[47] Frendo JL, Olivier D, Cheynet V, Blond JL, Bouton O, Vidaud M, Rabreau M, Evain-Brion D, Mallet F (2003). Direct involvement of HERV-W Env glycoprotein in human trophoblast cell fusion and differentiation. Mol Cell Biol..

[48] Malassine A, Pidoux G, Gerbaud P, Frendo JL, Evain-Brion D (2011). Human trophoblast in trisomy 21: a model for cell-cell fusion dynamic investigation. Adv Exp Med Biol..

[49] Vargas A, Moreau J, Landry S, LeBellego F, Toufaily C, Rassart E, Lafond J, Barbeau B (2009). Syncytin-2 plays an important role in the fusion of human trophoblast cells. J Mol Biol..

[50] Papp B, Plath K (2013). Epigenetics of reprogramming to induced pluripotency. Cell..

[51] Ibarretxe G, Crende O, Aurrekoetxea M, Garcia-Murga V, Etxaniz J, Unda F (2012). Neural crest stem cells from dental tissues: a new hope for dental and neural regeneration. Stem Cells Int..

