# Supplementary material for: Amenable epigenetic traits of dental pulp stem cells underlie high capability of xeno-free episomal reprogramming
Source: Stem Cell Res Ther. 2018 Mar 20;9:68. doi: 10.1186/s13287-018-0796-2 (PMC5859503; doi:10.1186/s13287-018-0796-2)
Supplement: Supplementary file 1 — Table S1. List of commercial and patient-derived dental stem cell samples used. Table S2. DiPS colony counts of dental pulp stem cell samples used for reprogramming under different culture conditions. Figure S1. Transduction efficiencies for reprogramming DPSCs. Figure S2. Generation of iPS cells from DPSCs in the presence of enhancer compounds. Figure S3. Time course during episomal-based reprogramming of DPSCs. Figure S4. Characterisation of DPSCs cultured in xeno-free media. Figure S5. Cytogenetics and in vivo characterisation of DiPS lines generated under xeno-free culture conditions. Table S3. List of differentially methylated regions of selected genes in DPSCs versus ASCs with respect to iPS (DiPS and AiPS) and H1 hES cell lines. Table S4. DNA methylation raw data analysed with multiple probe sets of PAX9 gene that exhibit significant differences between DPSCs and ASCs. Table S5. Top networks by ingenuity pathway analysis (IPA) for differentially methylated genes in ASCs versus AiPS cells that do not exhibit such differences in DPSCs versus DiPS cells. Figure S6. Pluripotent and self-renewal supporting characteristics of DPSCs. (ZIP 8876 kb) [file 13287_2018_796_MOESM1_ESM.zip › DiPS_Manuscript Suppl Info Final 190118.docx]

**Additional file 1**

**Table S1**

The list of commercial and patient-derived dental stem cells samples used**.**

**Table S2**

DiPS colony counts of dental pulp stem cell samples used for reprogramming under different culture conditions

**Figure S1**

**Transduction efficiencies for reprogramming DPSCs.**

1. Transduction and transfection efficiencies of DPSCs under retroviral conditions.

(i) GFP retroviral transduction of DPSC lines under feeder layer conditions.

(ii) GFP retroviral transduction of DPSC lines under feeder-free conditions.

1. Somatic cell morphology of DPSCs cultured in xeno-free media conditions and their transfection efficiencies using epiosmal based reprogramming.
2. Flow cytometry profiles for GFP positive cells of 5 DPSC lines used for epiosmal based reprogramming under xeno-free conditions. The control group was not transfected with episomal vectors, but underwent the same flow cytometry procedure.

**Figure S2**

**Generation of iPS cells from DPSCs in the presence of enhancer compounds.**

1. Colony formation efficiency of retroviruses (Viral) and episomal (NC) based reprogramming of L1 DPSCs with and without addition of small molceule cockatil (SMC4) under feeder (MEF) and feeder-free (MTG) conditions. Cells were photographed under phase contrast microscope in different time points post transduction. The left panel set shows the L1 DPSC cells in SMC4-free culture conditions. Right panel set shows L1 DPSC repgroammed cells cultured in SMC4-supplemented conditions.
2. Colony compactness of L1 DPSCs reprogrammed under the SMC4-supplemented conditions.

**Figure S3**

**Time course during episomal based reprogramming of DPSCs.**

1. Fibroblastic nature of DPSCs at d8 post transduction (typically seen from d0 till ~d10) under the episomal based reprogramming condition under feeder layers.
2. GFP expression showing 20-30% tranfection effieciency using the episomal based reprogramming approach.
3. First wave of morphological changes appearing for transduced DPSCs on d13.
4. Emerging compact colonies are seen in reprogrammed DPSCs by d22.
5. A developing DiPS colony by d25.
6. A culture expanded DiPS colony by d27.

**Figure S4**

**Characterization of DPSCs cultured in xeno-free media**

**(a)** Somatic cell morphology of three different DPSC cell lines cultured in normal expansion media and xeno-free media.

**(b)** Flow cytometry analysis of MSC marker expression showing the MSC potential of L1 DPSCs cultured for two weeks under the xeno-free condition compared with that under the normal condition.

**Figure S5**

**Cytogenetics and *in vivo* charcterization of DiPS lines generated under xeno-free culture conditions.**

1. Karyotyping results of hES cell line (H1) and DiPS lines (H1, L1_viral & AC_ADULT).
2. H&E staining of teratoma derived from all 5 DiPS lines. The teratoma contains tissues of all three germ layers.
3. Morphology of EBs at day 8 derived from one representative DiPS line (AC_ADULT iPS) and hES H1.

**Table S3**

List of differentially methylated regions of seleceted genes in DPSCs versus ASCs with respect to iPS (DiPS & AiPS) and H1 hES cell lines. These genes include *ADPRHL1, AGR3, AMBRA1, ANGPTL5, ARL4C, ARSJ, PLAU, C1QTNF3, C5orf38, C6orf105, CARD14, CASP1, CIDEC, CLU, COX15, ENSA, FERD3L, FHL1, FLJ32063, FMO2, FXYD7, FYB, GART, GDPD3, HAS2, HMGA1, HOXD3, HSPB6, IL1RL1, INTU, IPO11, ISLR, KAT2B, KCNA5, KIFC3, KLHL25, LEPROTL1, LHCGR, MAP3K14, ISLR2, MFAP5, MYO3B, NIN, NUP62, NUTF2, OGDH, PFN4, PI16, RAP2A, RBM47, RBMS3, RECK, REEP3, ROBO4, SDC4, SH2D2A, SIT1, SLC10A6, SLC26A8, SLC2A5, SLC41A2, SLC44A1, STON1, TAF5L, TCP11, TCTEX1D4, THNSL1, TMED3, TMEM182, TNFSF4, TNNT3, TNXB, TTC32, USP31, VEPH1, WISP2, ZC3H6,* and *ZNF503*.

**Table S4**

DNA methylation raw data analyzed with multiple probe sets of *PAX9* gene that exhibit significant differences between DPSCs and ASCs. Note that the methylation levels of these probes in DPSCs are closer to those in ES and iPS cells.

**Table S5**

Top networks by Ingenuity pathway analysis (IPA) for differentially methylated genes in ASCs versus AiPS cells that do not exhibit such differences in DPSCs versus DiPS cells.

**Figure S6**

**Pluripotent and self-renewal supporting characteristics of DPSCs.**

1. Immunofluorescence results of pluripotent protein marker expression in small clonal-like structure within UM DPSCs cultured under normal culture conditions.
2. Flow cytometric results of pluripotent surface markers for H1 ES cell lines when cultured on UM DPSCs, ASCs and MEF as feedeer layers.

**Table S1**

| **DPSC samples used for DiPS generation** | **Sources**  **Commercial/**  **Patient derived)** | **Age** | **Sex** | **Passage number** |
| --- | --- | --- | --- | --- |
| L1 | Lonza | NA | Female | P4 |
| L2 | Lonza | 18yrs | Male | P4 |
| AC_SHED | All Cells | NA | NA | P4 |
| AC_ADULT | All Cells | NA | NA | P4 |
| UM | Patient derived | 12yrs | Female | P6 |

1. *L1 = Denoting the sample 1 of commercial source “Lonza”.*
2. *L2 = Denoting the sample 2 of commercial source “Lonza”.*
3. *AC_SHED = Denoting the sample 1 of commercial source “All Cells” which was derived from human exfoliated deciduous teeth (younger patients).*
4. *AC_ADULT = Denoting the sample 2 of commercial source “All Cells” which was derived from adult teeth. We used “ADULT” term for easier reference.*
5. *UM = Patient sample derived from University of Malaya.*
6. *NA = Information Not Available*

**Table S2**

| **Sl No:** | **DPSC samples** | **No. of iPS colonies*^1^*** |
| --- | --- | --- |
| 1 | Viral_MEF | 19.6+1.1 |
| 2 | Viral_MEF_SMC4 | 25+5 |
| 3 | Viral_MTG | 3+2.6 |
| 4 | Viral_MTG_SMC4 | 8.6+2 |
| 5 | NC_MEF | 0.3+0.5 |
| 6 | NC_MEF_SMC4 | 2.3+2.5 |
| 7 | NC_MTG | 1.6+1.1 |
| 8 | NC_MTG_SMC4 | 16.3+4.9 |

1. *n=3 (3 wells of 6 well plate), mean±SE*
2. Viral_MEF *= Viral based reprogramming under MEF feeder layer without SMC4*
3. *Viral_MEF_SMC4 = Viral based reprogramming under MEF feeder layer with SMC4*
4. *Viral_MTG = Viral based reprogramming under Matrigel without SMC4*
5. *Viral_MTG_SMC4 = Viral based reprogramming under Matrigel with SMC4*
6. *NC_MEF = Nucelofected based episomal reprogramming under MEF feeder layer without SMC4*
7. *NC_MEF_SMC4 = Nucelofected based episomal reprogramming under MEF feeder layer with SMC4*
8. *NC_MTG = Nucelofected based episomal reprogramming under Matrigel without SMC4*
9. *NC_MTG_SMC4 = Nucelofected based episomal reprogramming under Matrigel with SMC4*

**Figure S1**


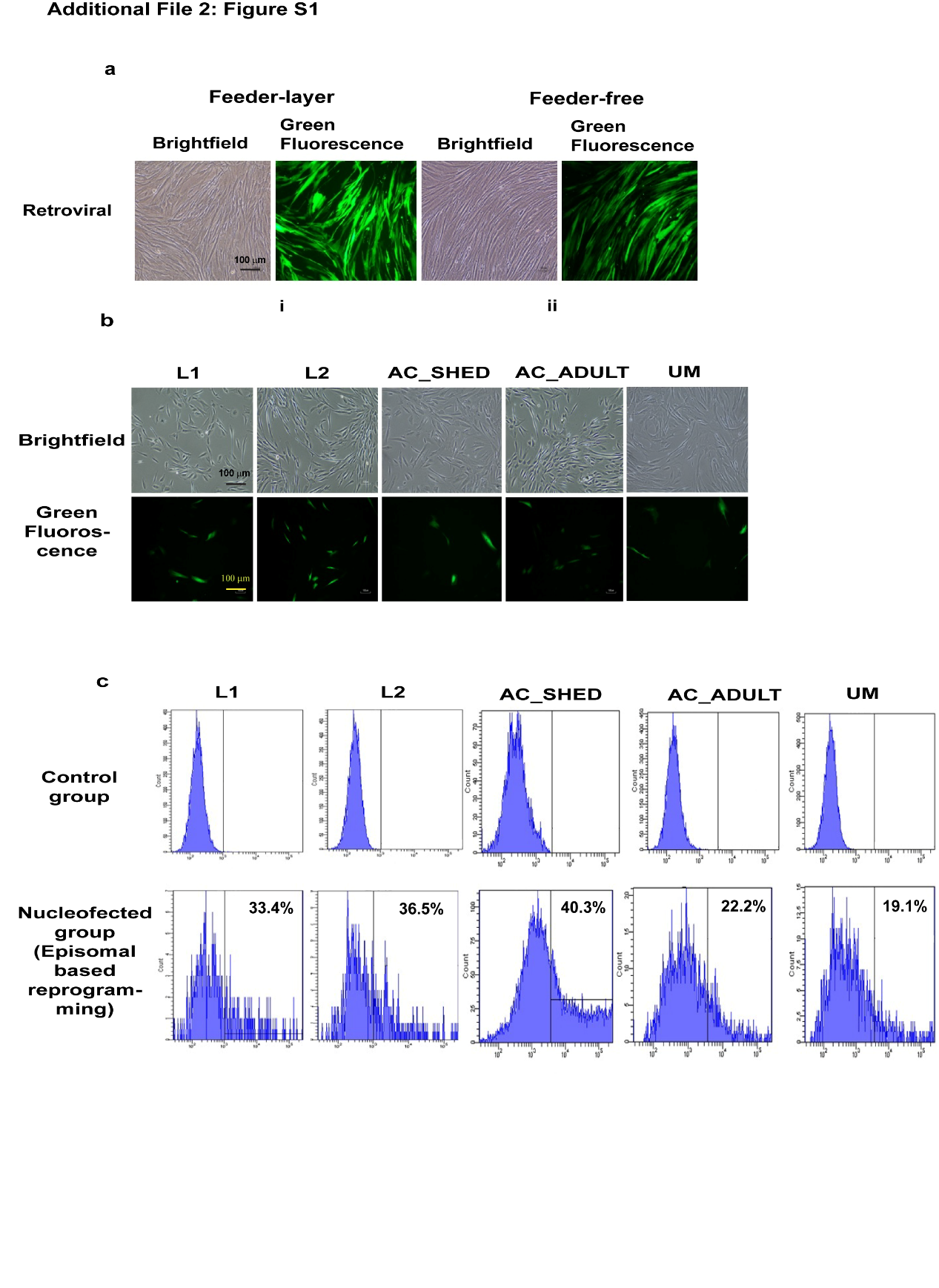


**Figure S2**


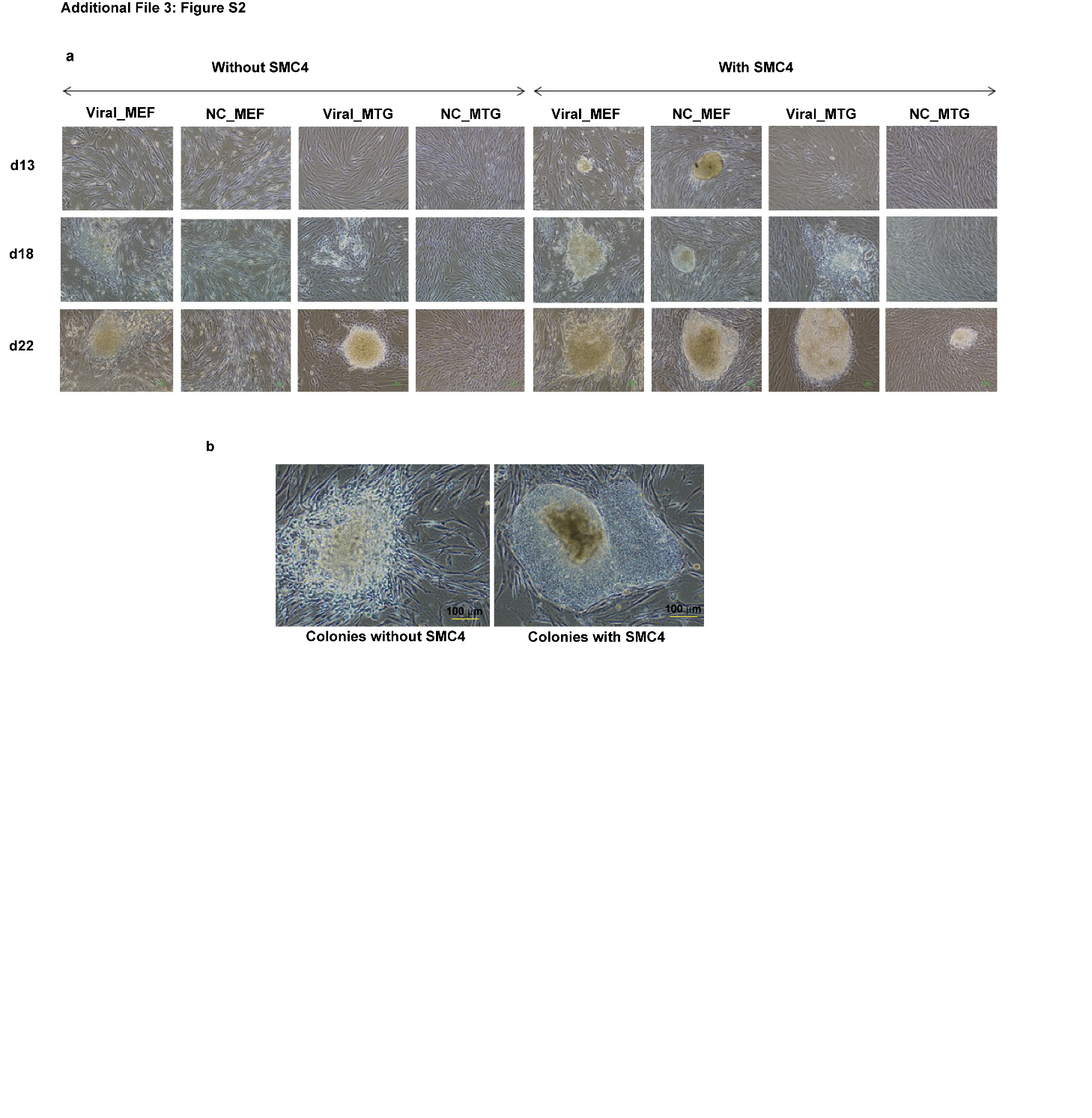
**Figure S3**


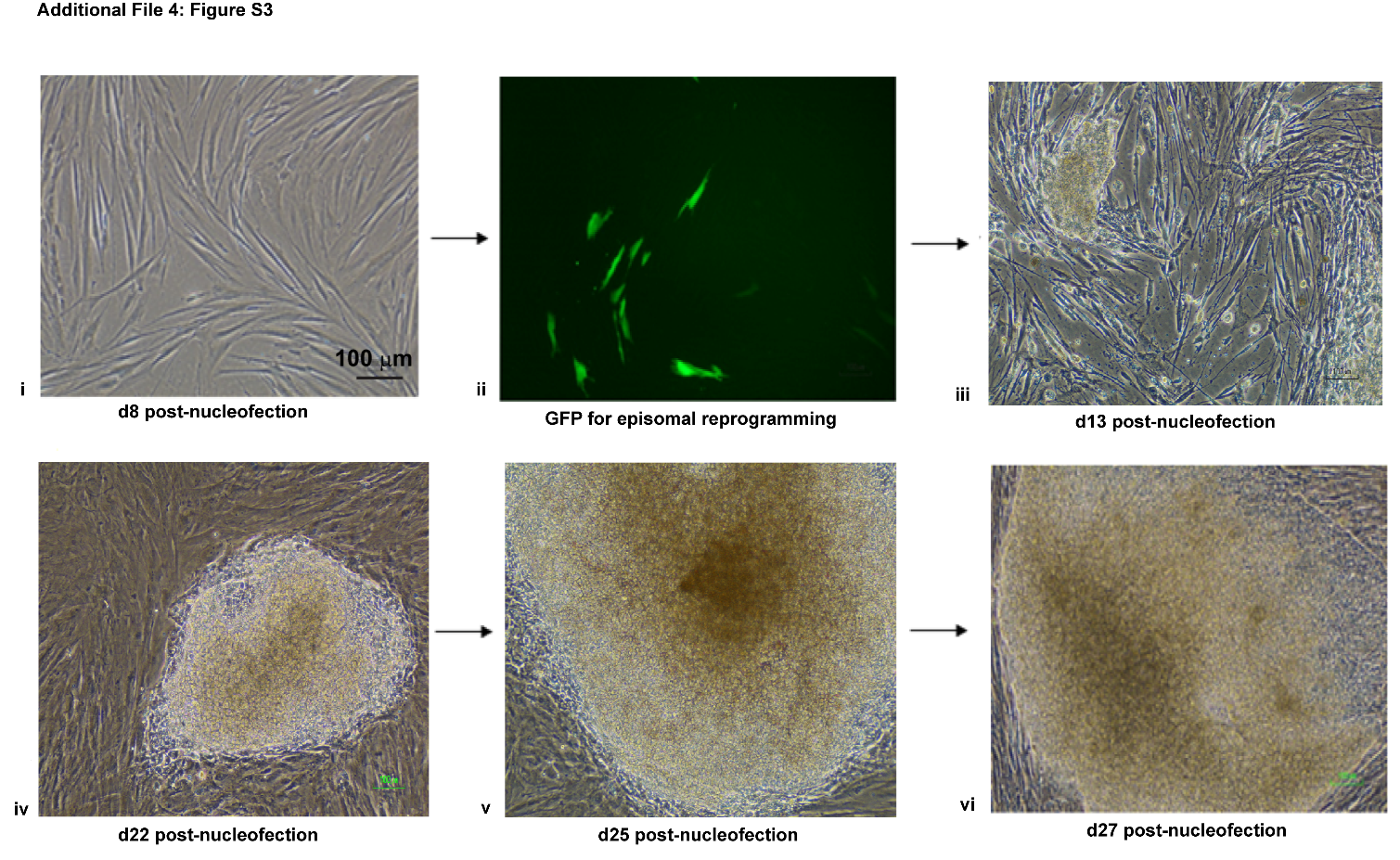


**Figure S4**


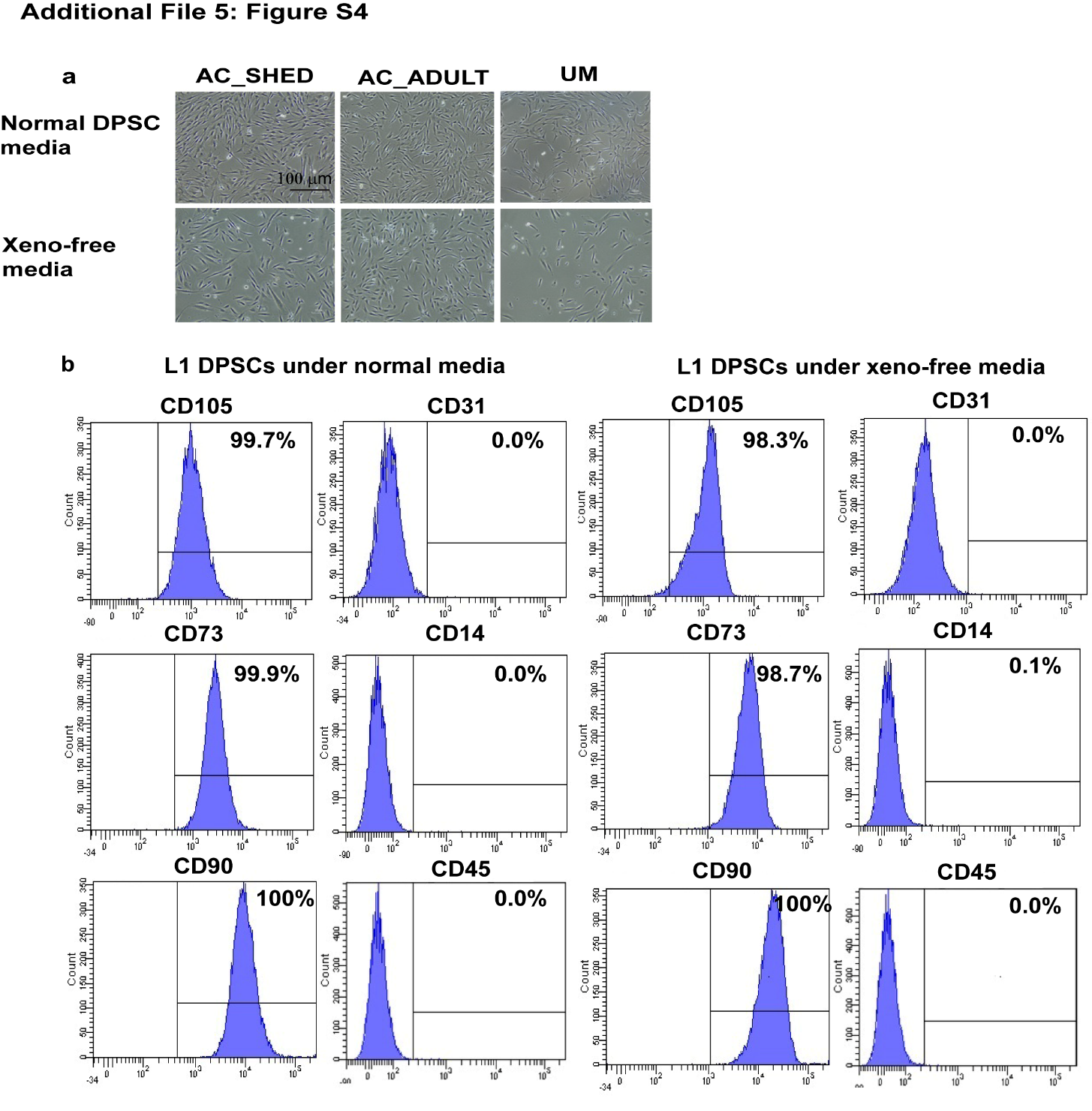


**Figure S5**


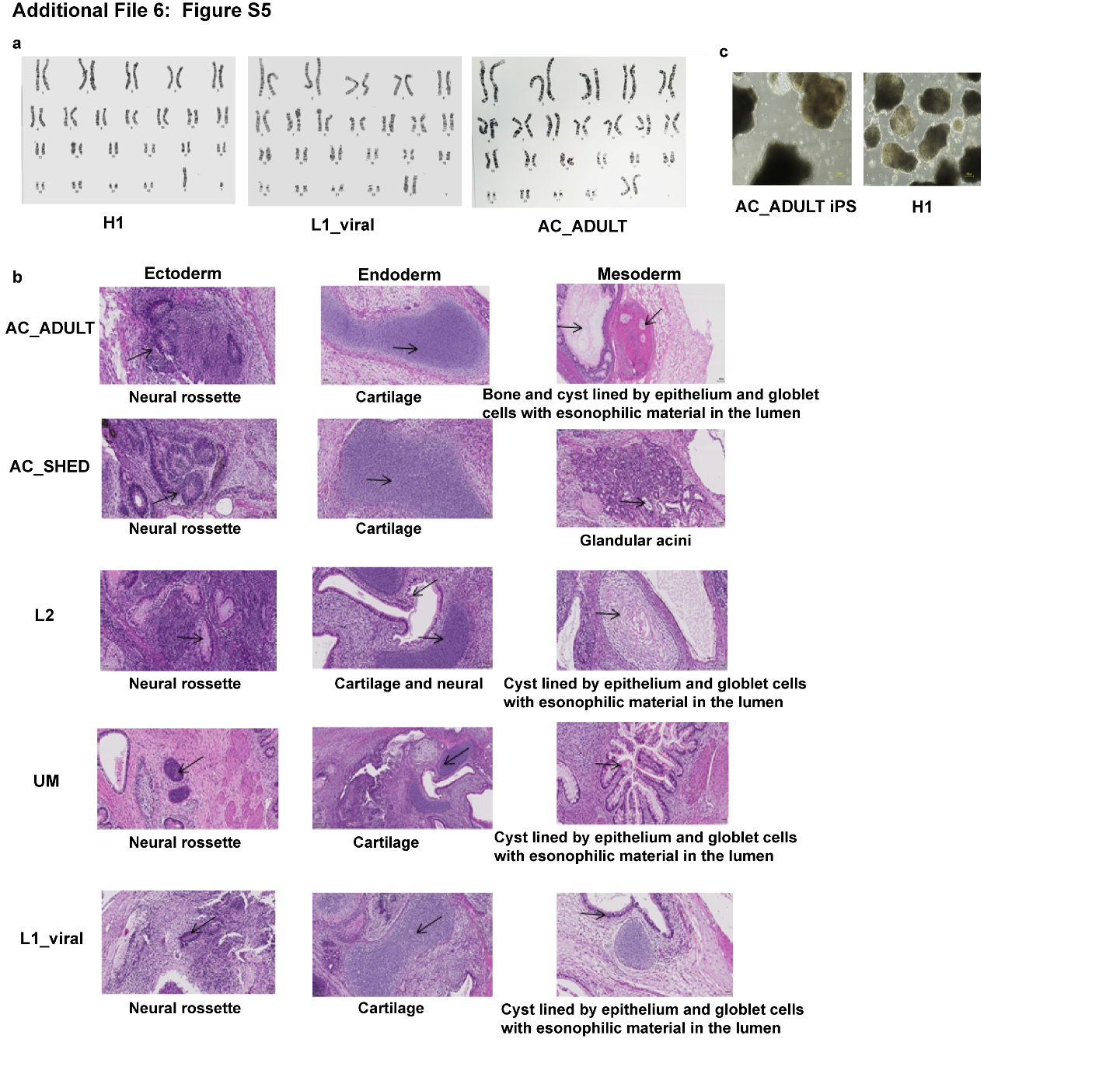


**Figure S6**


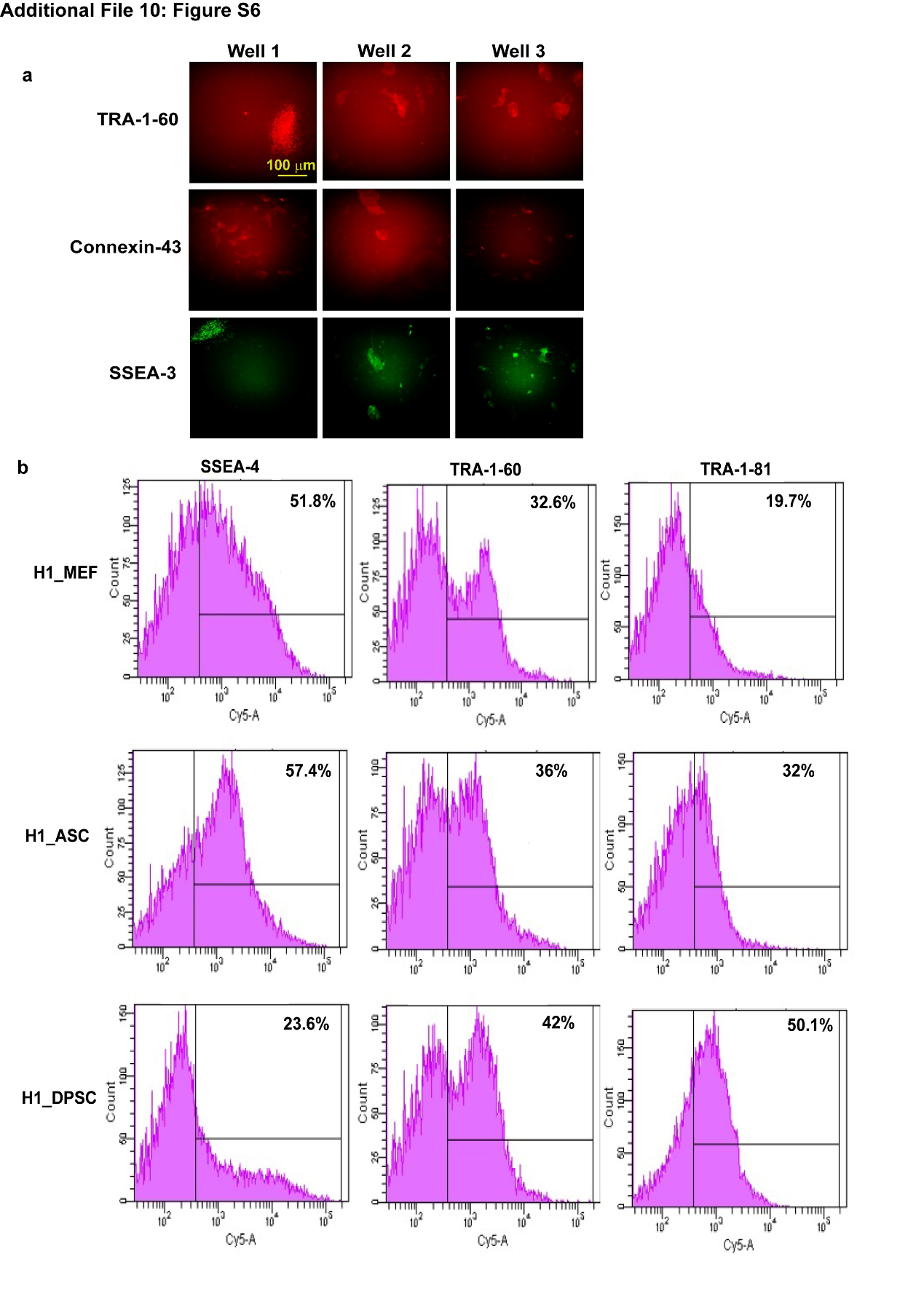


**Table S5**

**Top networks by Ingenuity Pathway analysis (IPA) for differentially methylated genes in ASCs versus AiPS cells that do not exhibit such differences in DPSCs versus DiPS cells.**

| **Top enriched networks:** | | |
| --- | --- | --- |
| **Molecular and Cellular Functions** | **P-value** | **#Molecules** |
| Cellular Movement | 1.74E-02 - 1.58E-04 | 75 |
| Cellular Assembly and Organization | 1.74E-02 - 2.81E-04 | 30 |
| Cell-To-Cell Signaling and Interaction | 1.84E-02 - 4.78E-04 | 74 |
| **Top Networks** | | **Score** |
| Developmental Disorder, Hereditary Disorder, Metabolic Disease | | 44 |
| Cellular Development, Cellular Growth and Proliferation, Connective Tissue Development and Function | | 42 |
| Gene Expression, Cell Morphology, Cellular Assembly and Organization | | 39 |

**Table S3 (from next page)**

**List of differentially methylated regions of seleceted genes in DPSCs versus ASCs with respect to iPS (DiPS & AiPS) and H1 hES cell lines.**

**Table S4 (after Table S3)**

DNA methylation raw data analyzed with multiple probe sets of *PAX9* gene that exhibit significant differences between DPSCs and ASCs.
