# Supplementary material for: Amenable epigenetic traits of dental pulp stem cells underlie high capability of xeno-free episomal reprogramming
Source: Stem Cell Res Ther. 2018 Mar 20;9:68. doi: 10.1186/s13287-018-0796-2 (PMC5859503; doi:10.1186/s13287-018-0796-2)
Supplement: Supplementary file 1 — Table S1. List of commercial and patient-derived dental stem cell samples used. Table S2. DiPS colony counts of dental pulp stem cell samples used for reprogramming under different culture conditions. Figure S1. Transduction efficiencies for reprogramming DPSCs. Figure S2. Generation of iPS cells from DPSCs in the presence of enhancer compounds. Figure S3. Time course during episomal-based reprogramming of DPSCs. Figure S4. Characterisation of DPSCs cultured in xeno-free media. Figure S5. Cytogenetics and in vivo characterisation of DiPS lines generated under xeno-free culture conditions. Table S3. List of differentially methylated regions of selected genes in DPSCs versus ASCs with respect to iPS (DiPS and AiPS) and H1 hES cell lines. Table S4. DNA methylation raw data analysed with multiple probe sets of PAX9 gene that exhibit significant differences between DPSCs and ASCs. Table S5. Top networks by ingenuity pathway analysis (IPA) for differentially methylated genes in ASCs versus AiPS cells that do not exhibit such differences in DPSCs versus DiPS cells. Figure S6. Pluripotent and self-renewal supporting characteristics of DPSCs. (ZIP 8876 kb) [file 13287_2018_796_MOESM1_ESM.zip › Table S3.pdf]

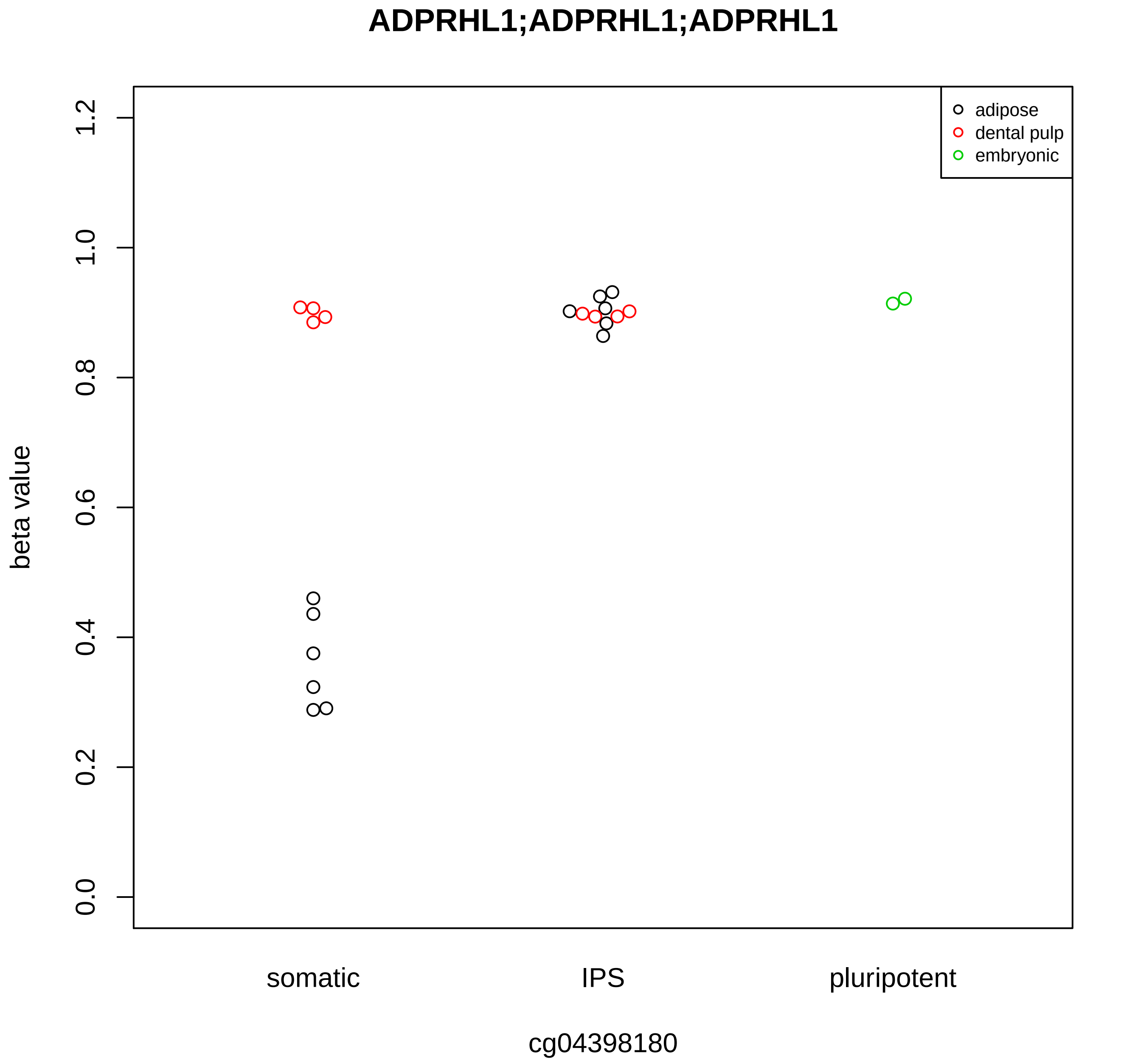
ADPRHL1;ADPRHL1;ADPRHL1
beta value
0.0
0.2
0.4
0.6
0.8
1.0
1.2
adipose
dental pulp
embryonic
somatic
IPS
pluripotent
cg04398180

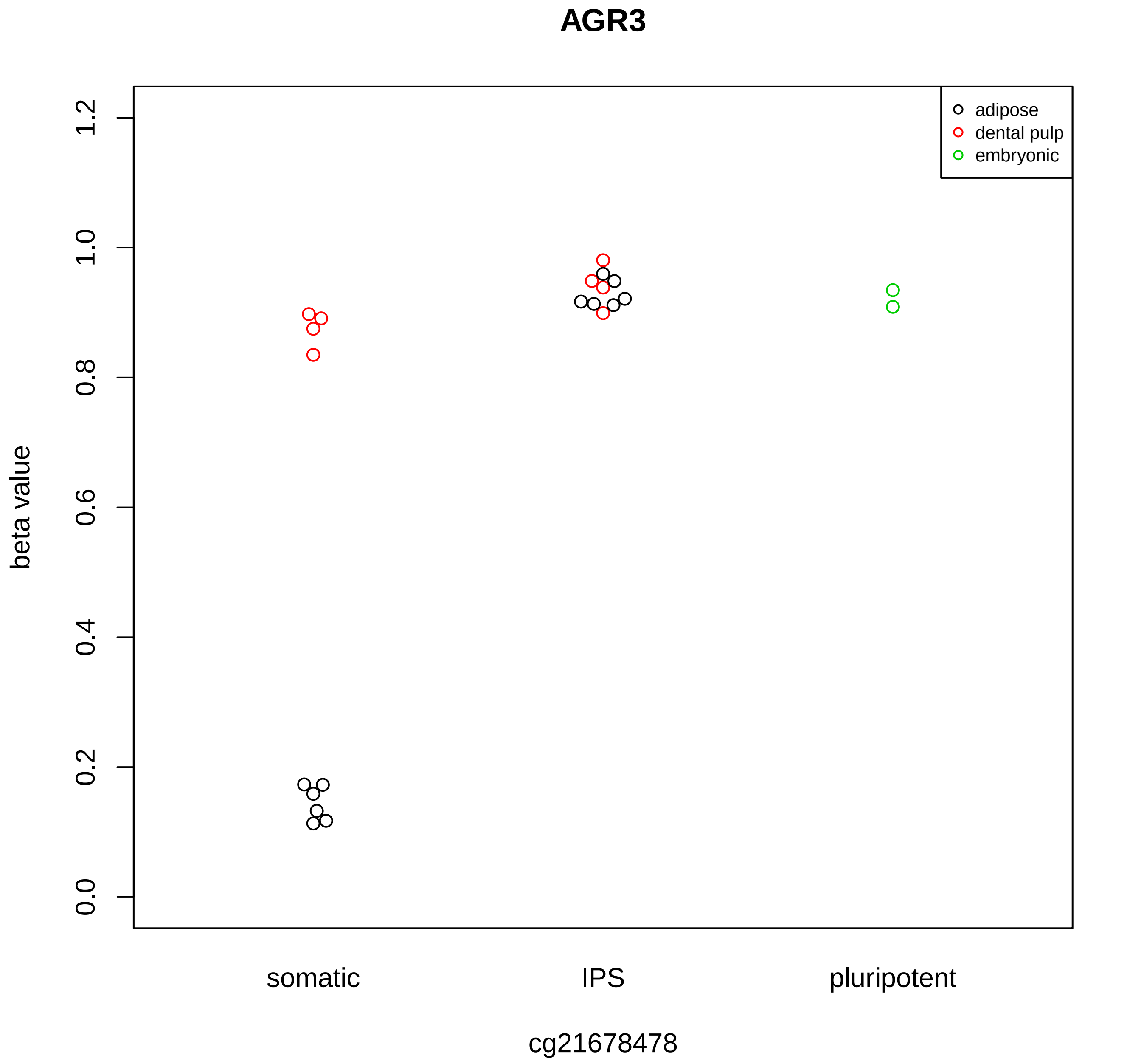
AGR3
beta value
0.0
0.2
0.4
0.6
0.8
1.0
1.2
adipose
dental pulp
embryonic
somatic
IPS
pluripotent
cg21678478

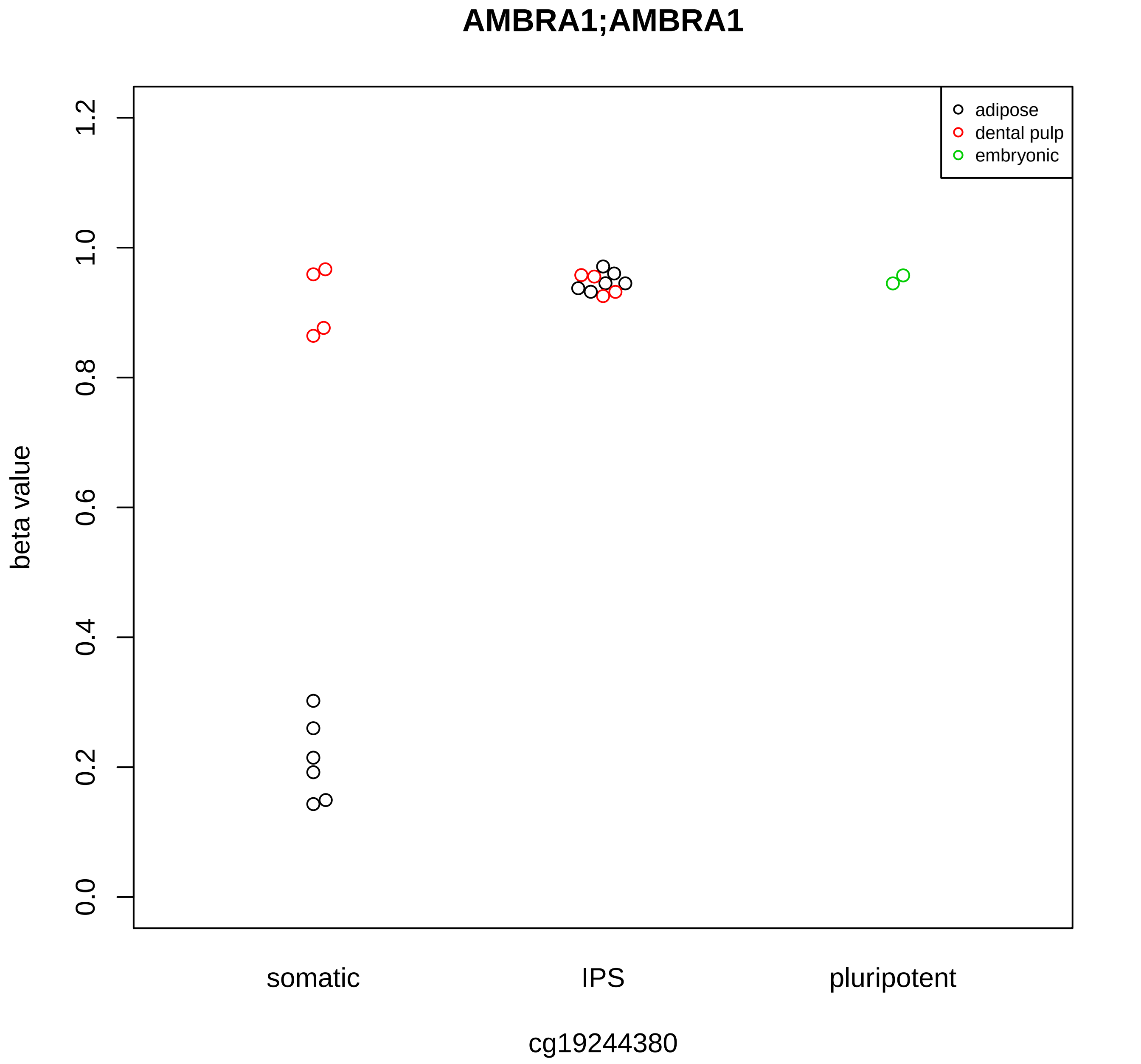

AMBRA1;AMBRA1
adipose
dental pulp
embryonic
beta value
0.0
0.2
0.4
0.6
0.8
1.0
1.2
somatic
IPS
pluripotent
cg19244380

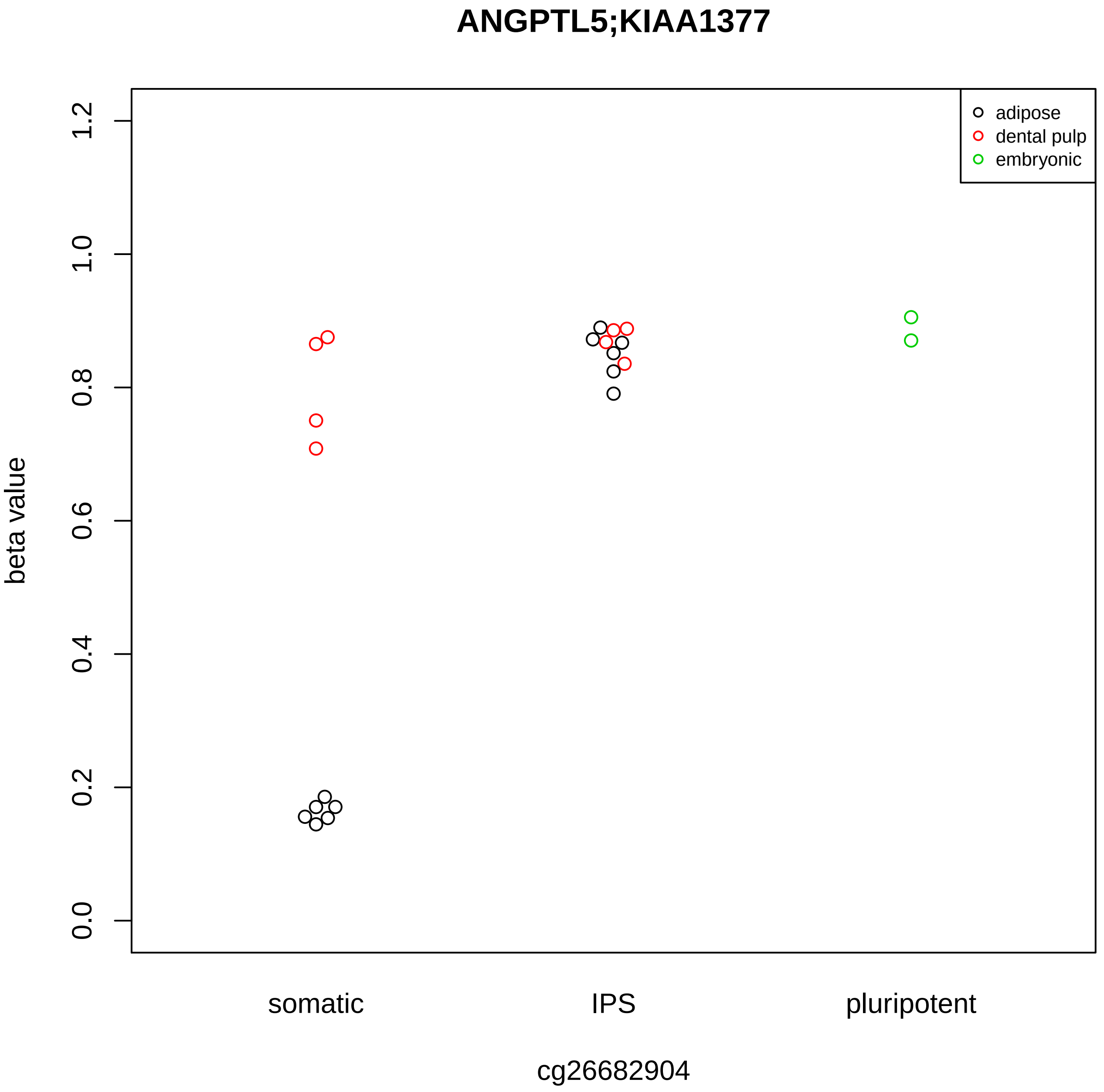
ANGPTL5;KIAA1377
adipose
dental pulp
embryonic
beta value
0.0
0.2
0.4
0.6
0.8
1.0
1.2
somatic
IPS
pluripotent
cg26682904

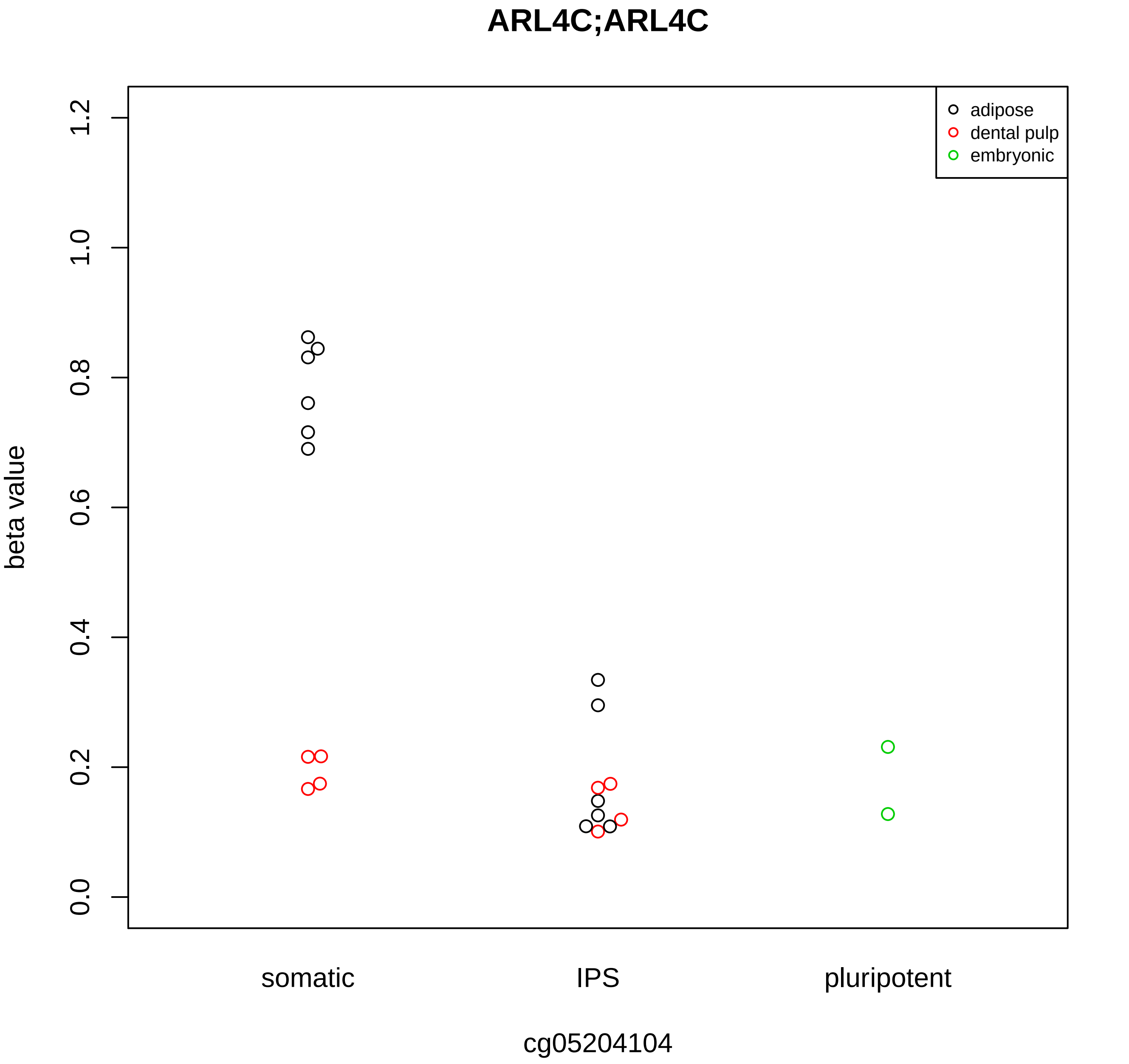
ARL4C;ARL4C
beta value
0.0
0.2
0.4
0.6
0.8
1.0
1.2
adipose
dental pulp
embryonic
somatic
IPS
pluripotent
cg05204104

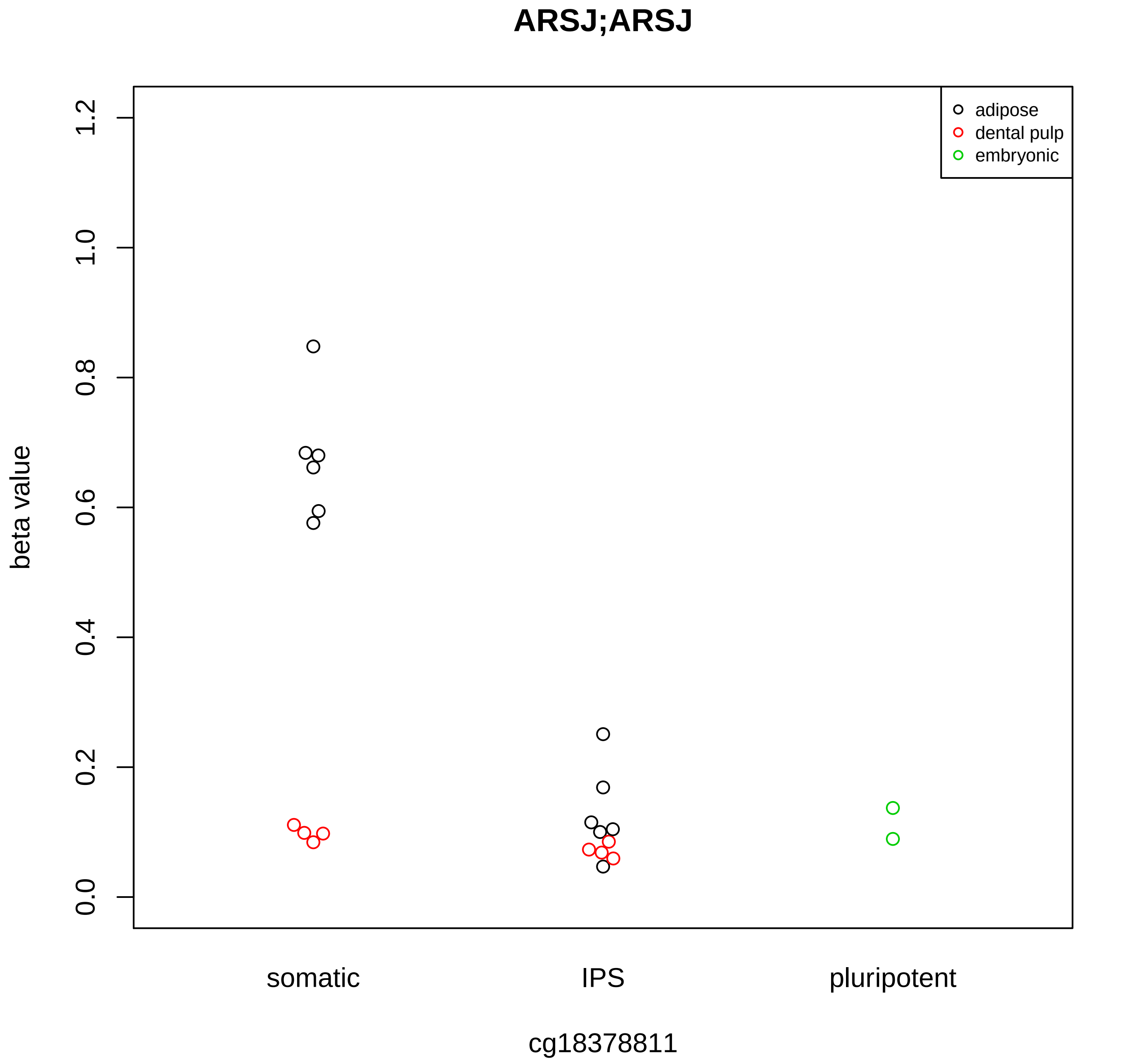

ARSJ;ARSJ
beta value
0.0
0.2
0.4
0.6
0.8
1.0
1.2
adipose
dental pulp
embryonic
somatic
IPS
pluripotent
cg18378811

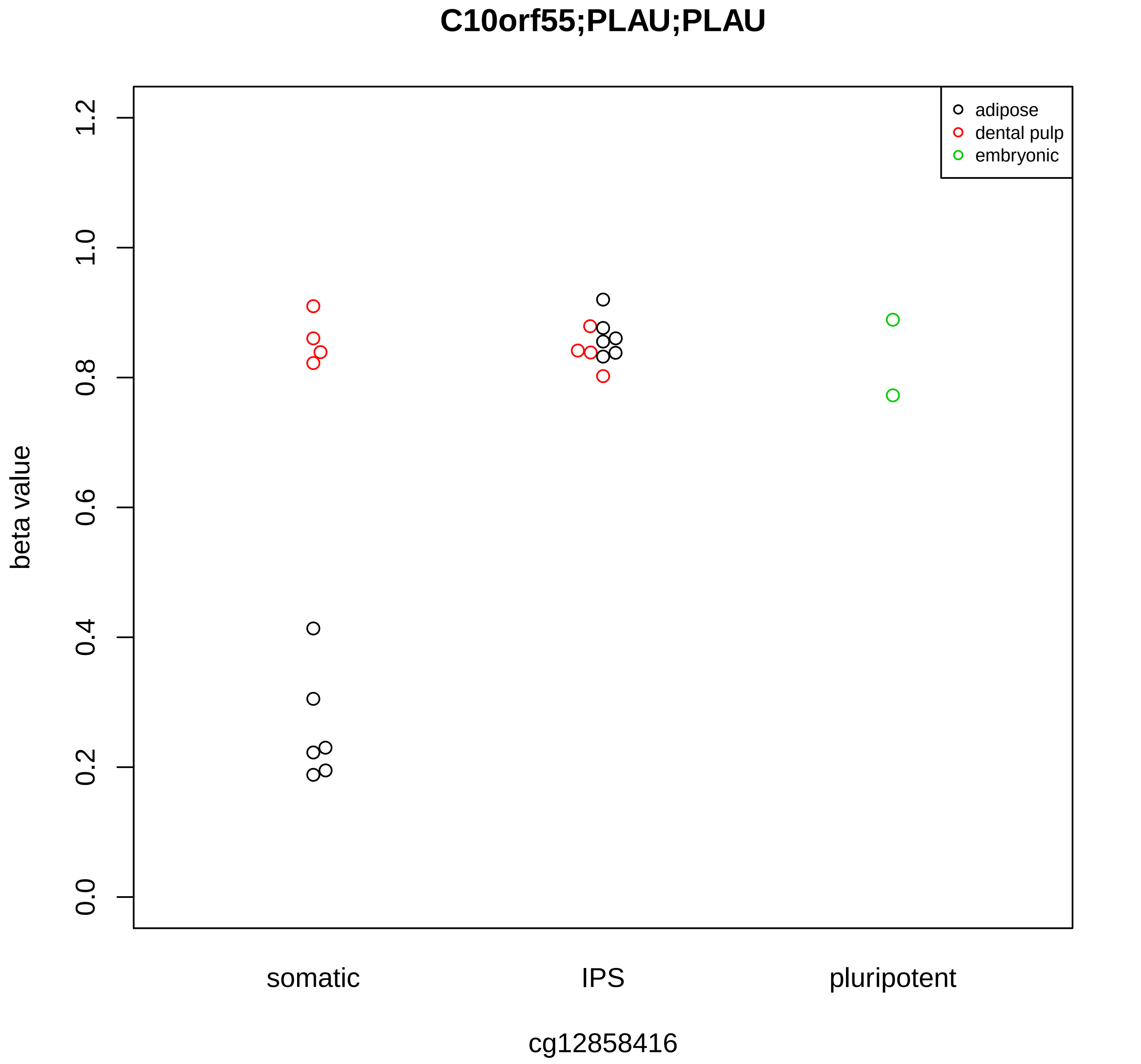

C10orf55;PLAU;PLAU
adipose
dental pulp
embryonic
beta value
0.0
0.2
0.4
0.6
0.8
1.0
1.2
somatic
IPS
pluripotent
cg12858416

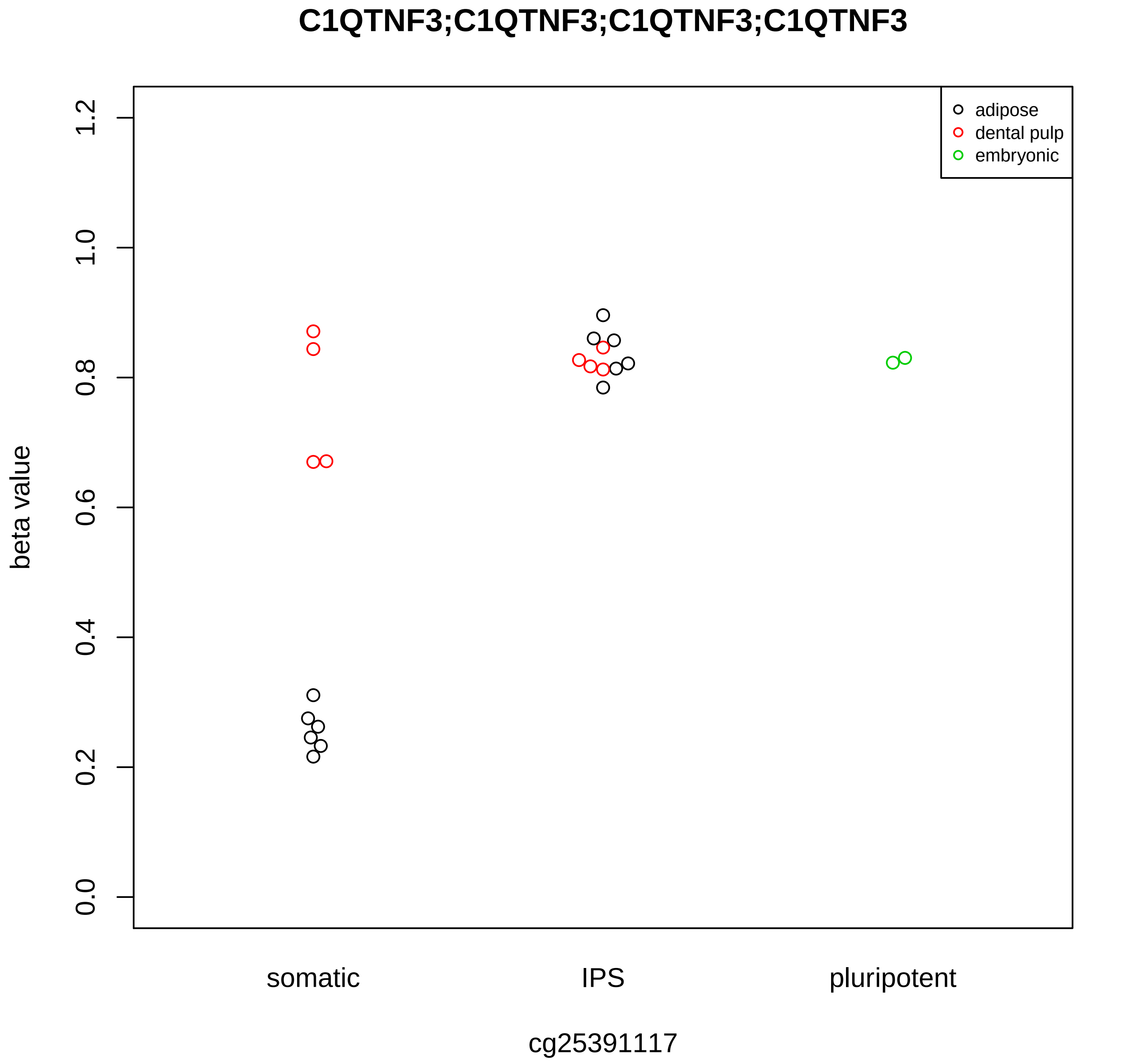
C1QTNF3;C1QTNF3;C1QTNF3;C1QTNF3
beta value
0.0
0.2
0.4
0.6
0.8
1.0
1.2
adipose
dental pulp
embryonic
somatic
IPS
pluripotent
cg25391117

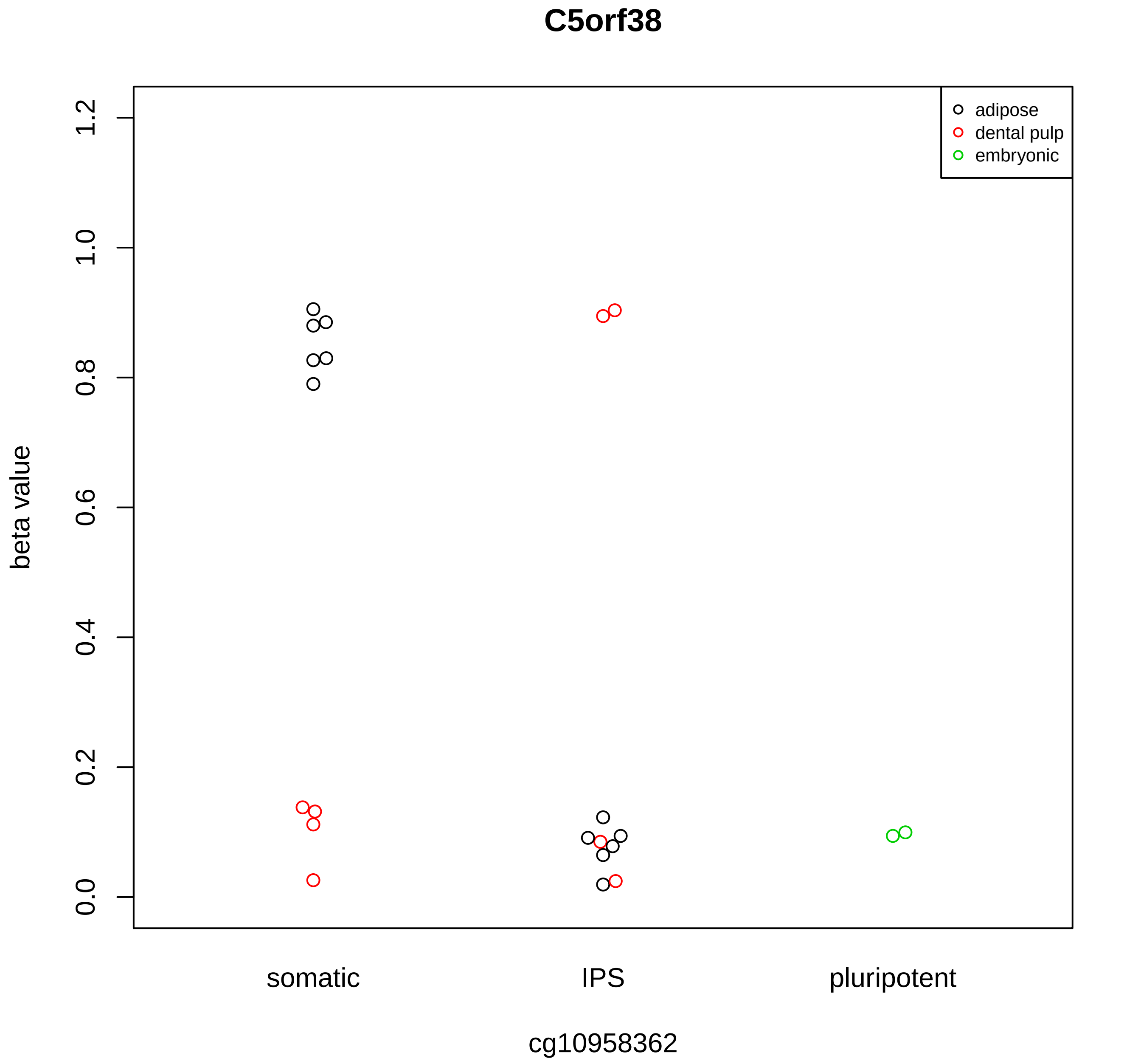
C5orf38
adipose
dental pulp
embryonic
beta value
0.0
0.2
0.4
0.6
0.8
1.0
1.2
somatic
IPS
pluripotent
cg10958362

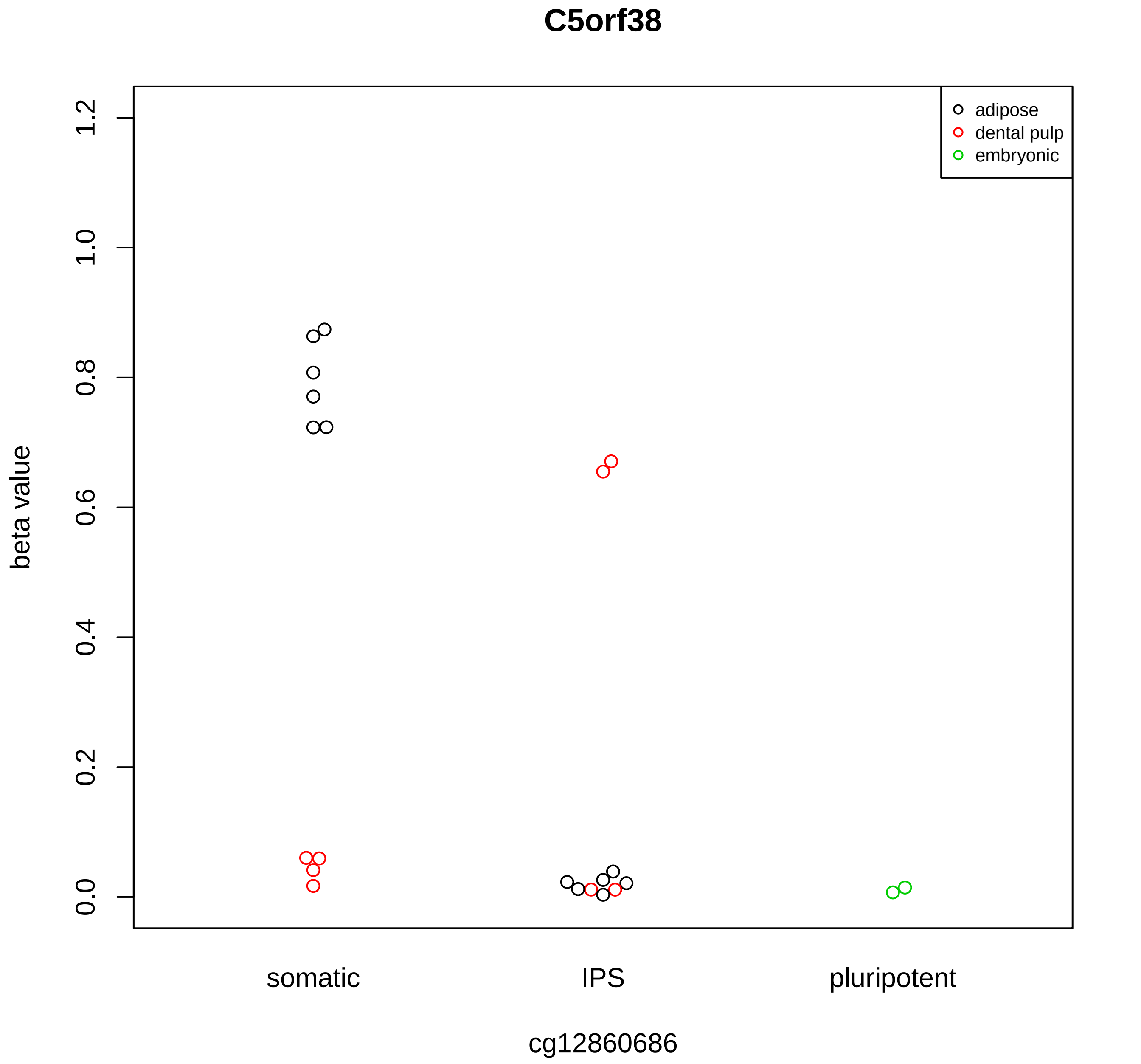
C5orf38
beta value
1.2
1.0
0.8
0.6
0.4
0.2
0.0
adipose
dental pulp
embryonic
somatic
IPS
pluripotent
cg12860686

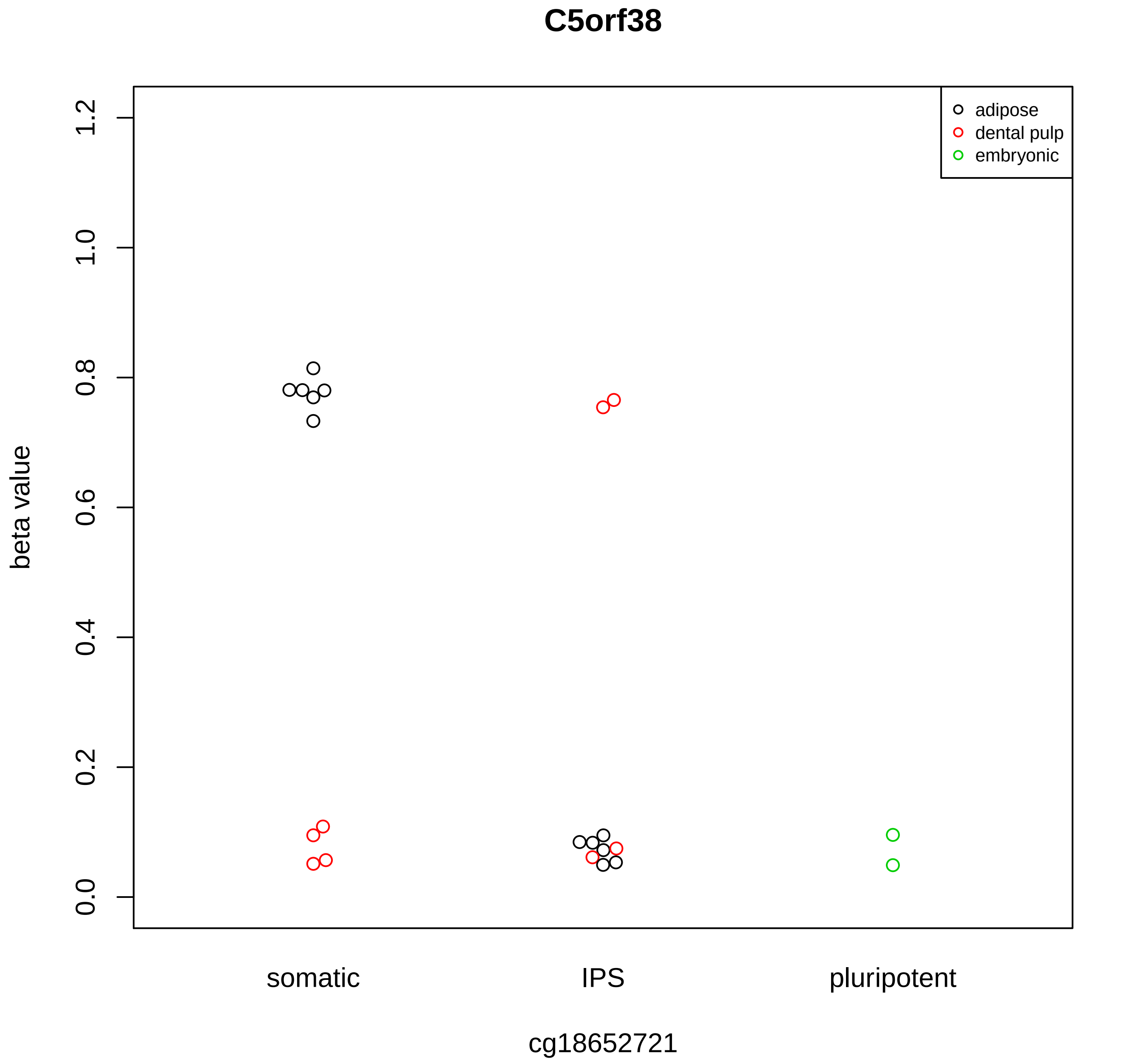
C5orf38
beta value
0.0
0.2
0.4
0.6
0.8
1.0
1.2
adipose
dental pulp
embryonic
somatic
IPS
pluripotent
cg18652721

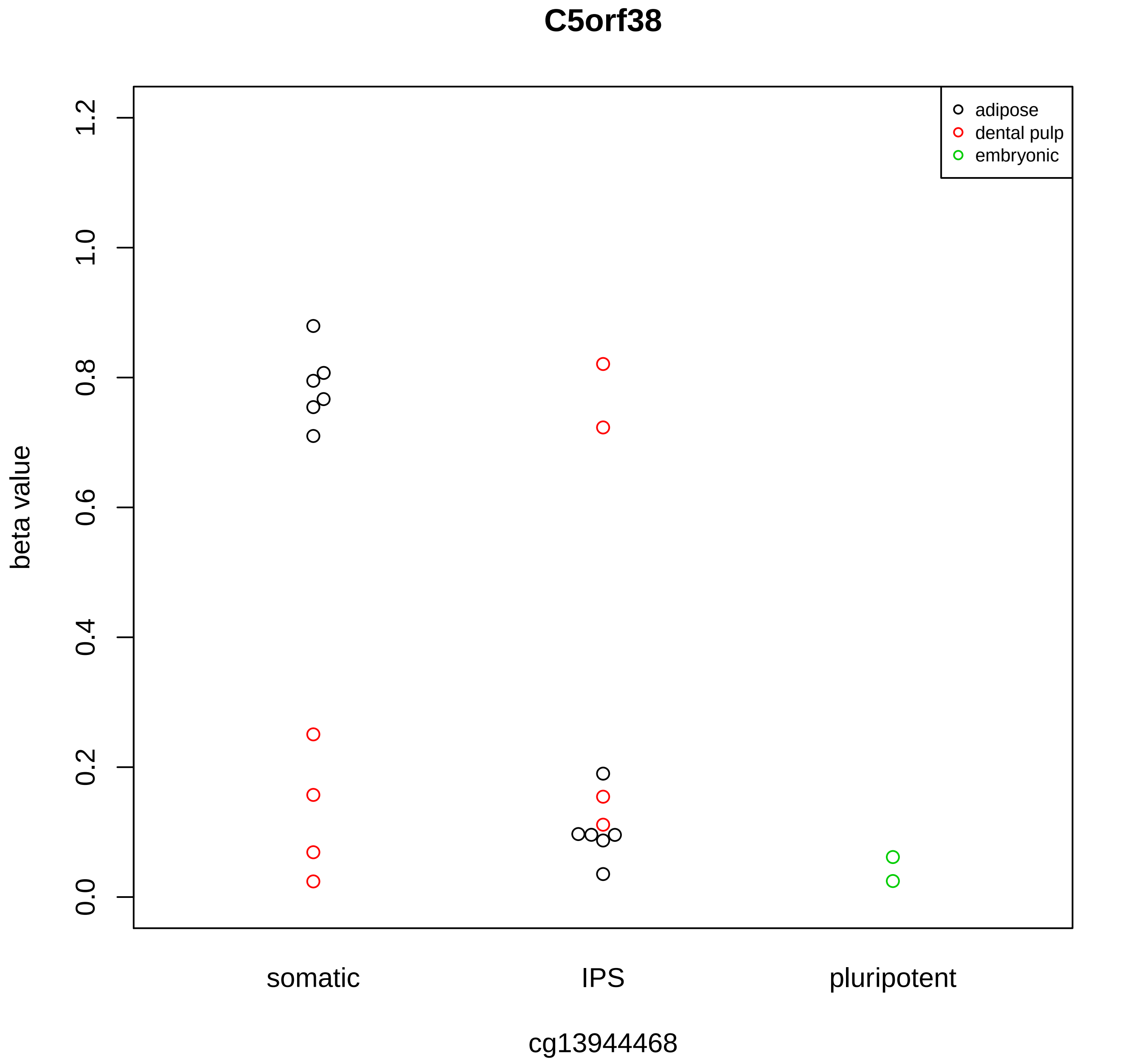

C5orf38
adipose
dental pulp
embryonic
beta value
0.0
0.2
0.4
0.6
0.8
1.0
1.2
somatic
IPS
pluripotent
cg13944468

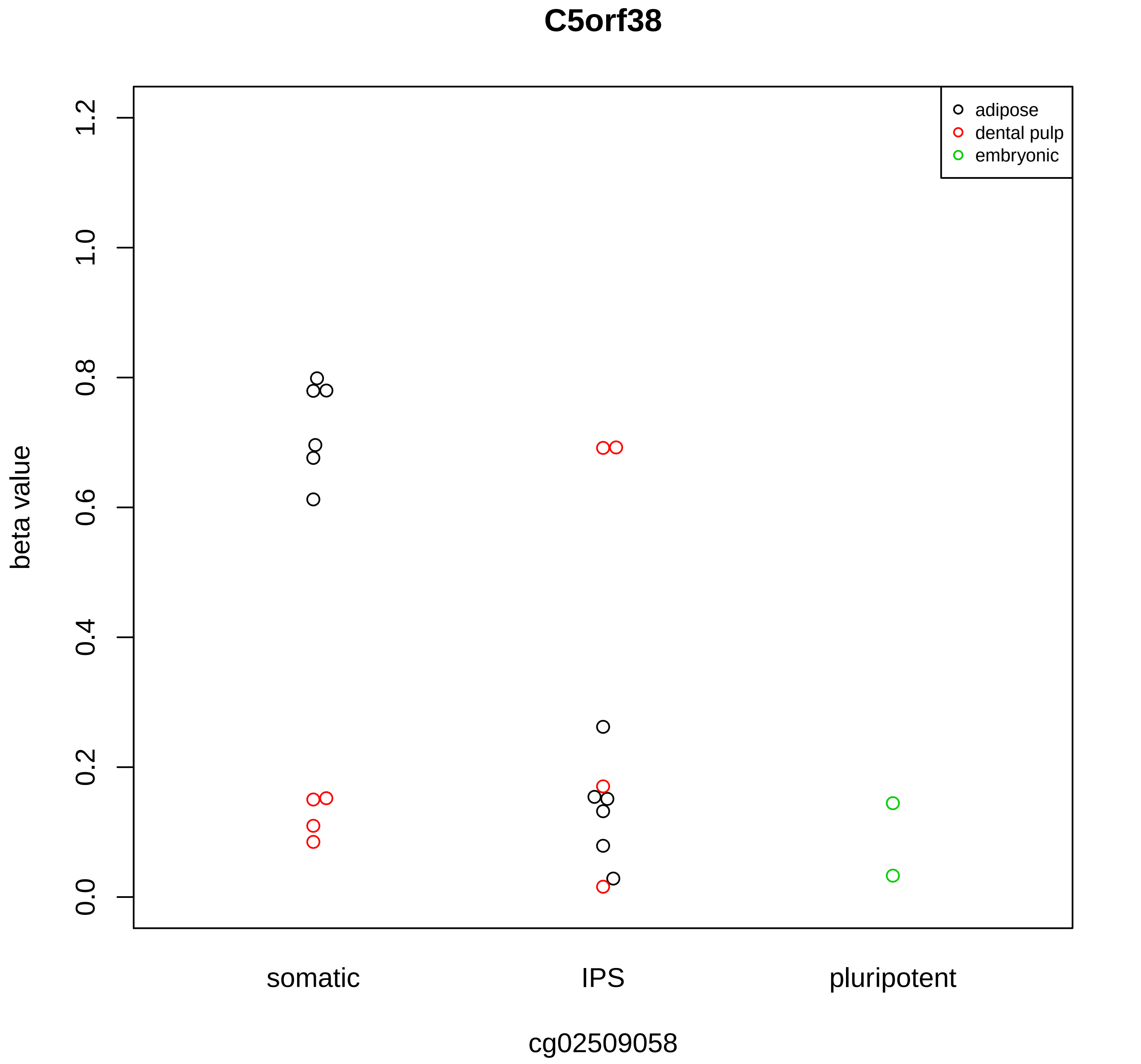

C5orf38
adipose
dental pulp
embryonic
beta value
0.0
0.2
0.4
0.6
0.8
1.0
1.2
somatic
IPS
pluripotent
cg02509058

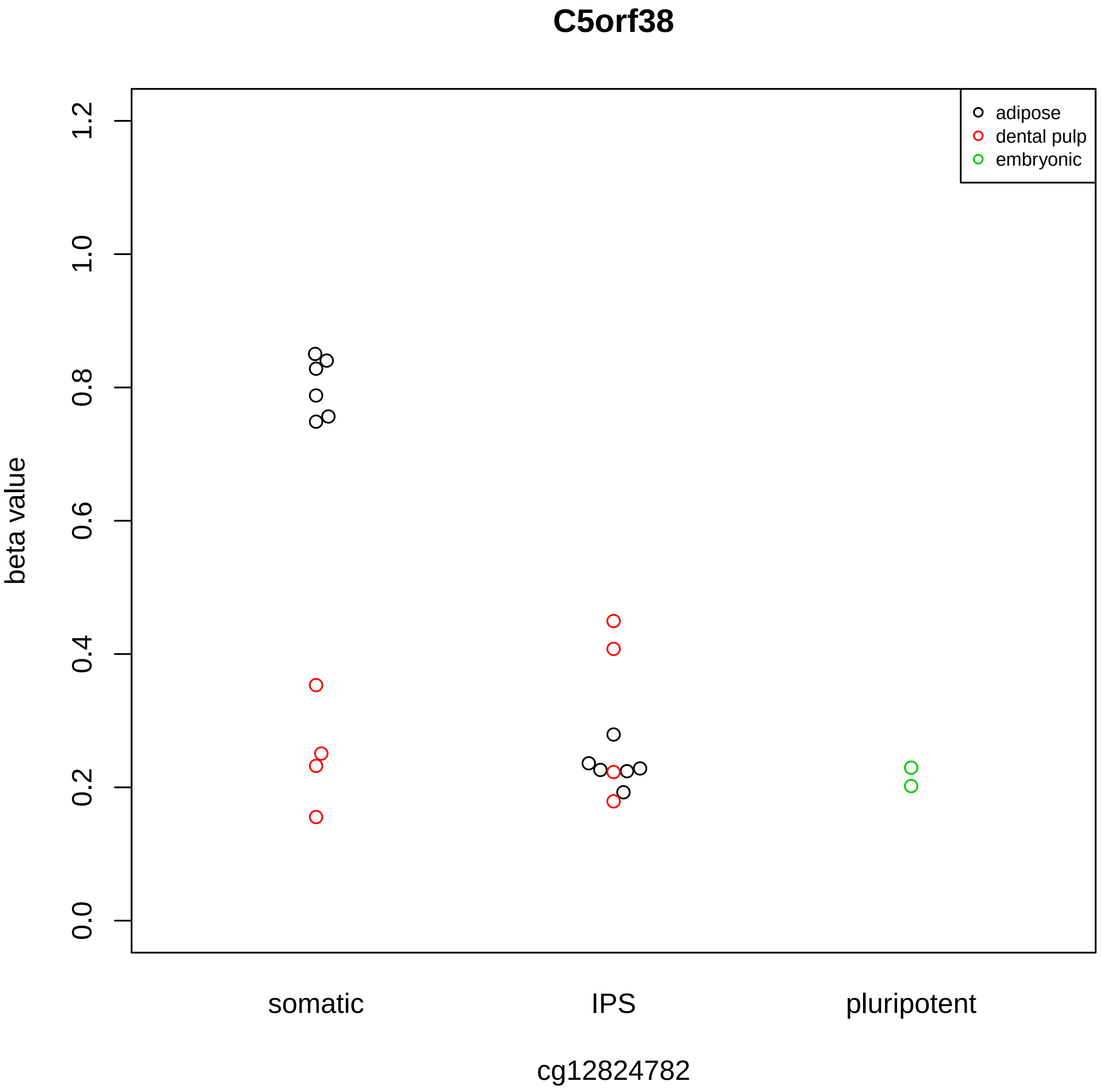
C5orf38
beta value
0.0
0.2
0.4
0.6
0.8
1.0
1.2
adipose
dental pulp
embryonic
somatic
IPS
pluripotent
cg12824782

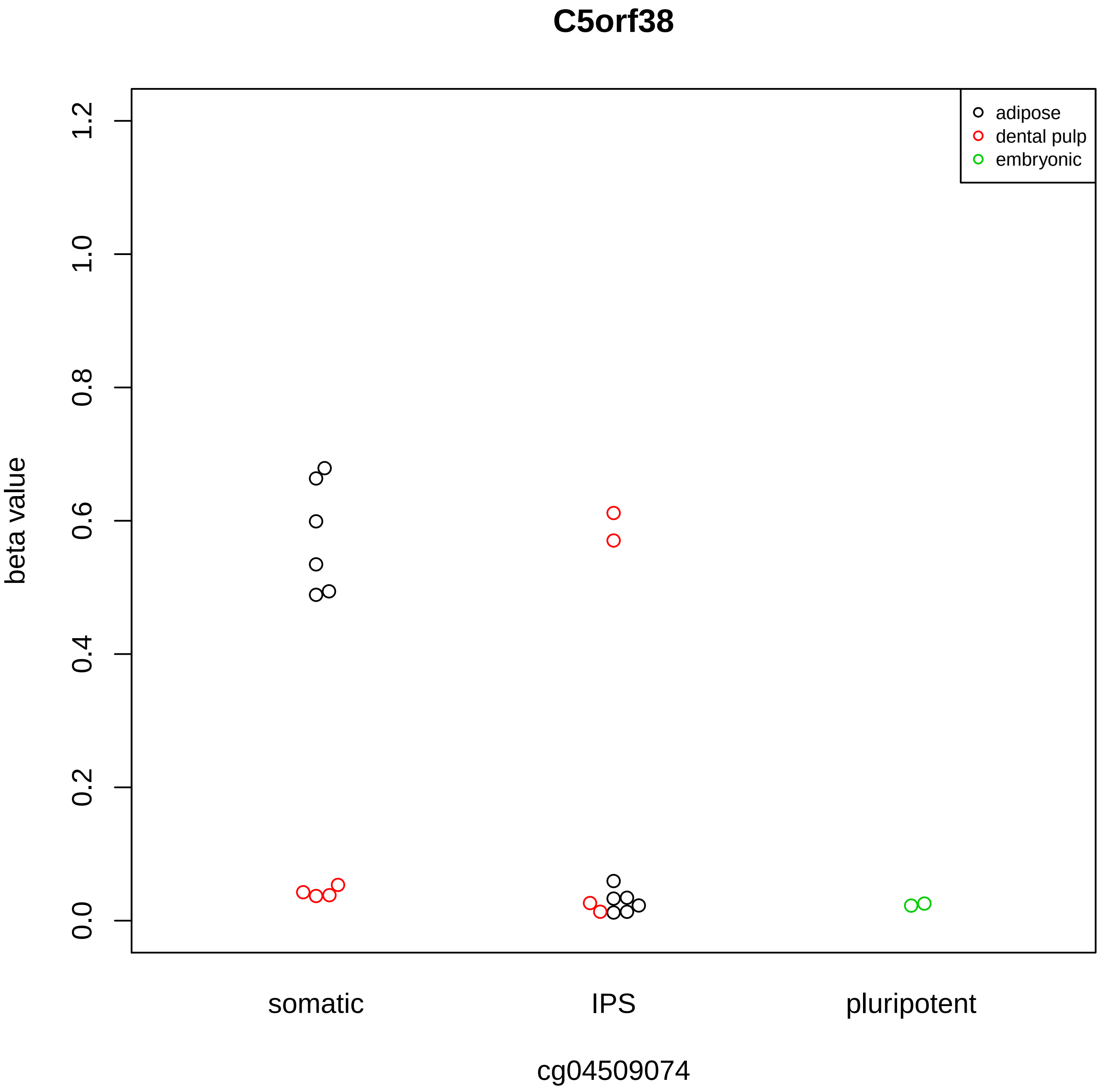
C5orf38
beta value
0.0
0.2
0.4
0.6
0.8
1.0
1.2
adipose
dental pulp
embryonic
somatic
IPS
pluripotent
cg04509074

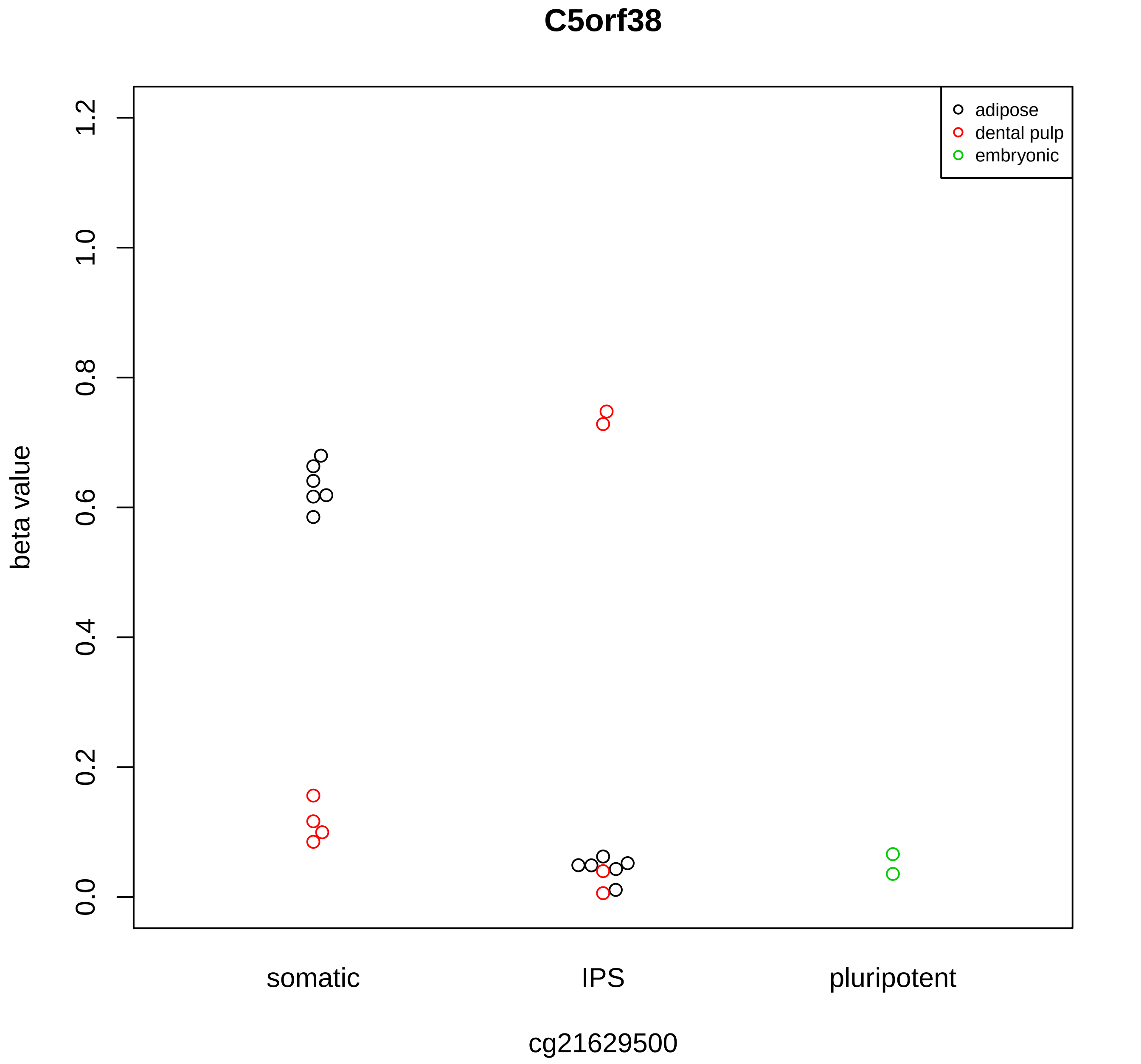
C5orf38
beta value
0.0
0.2
0.4
0.6
0.8
1.0
1.2
adipose
dental pulp
embryonic
somatic
IPS
pluripotent
cg21629500

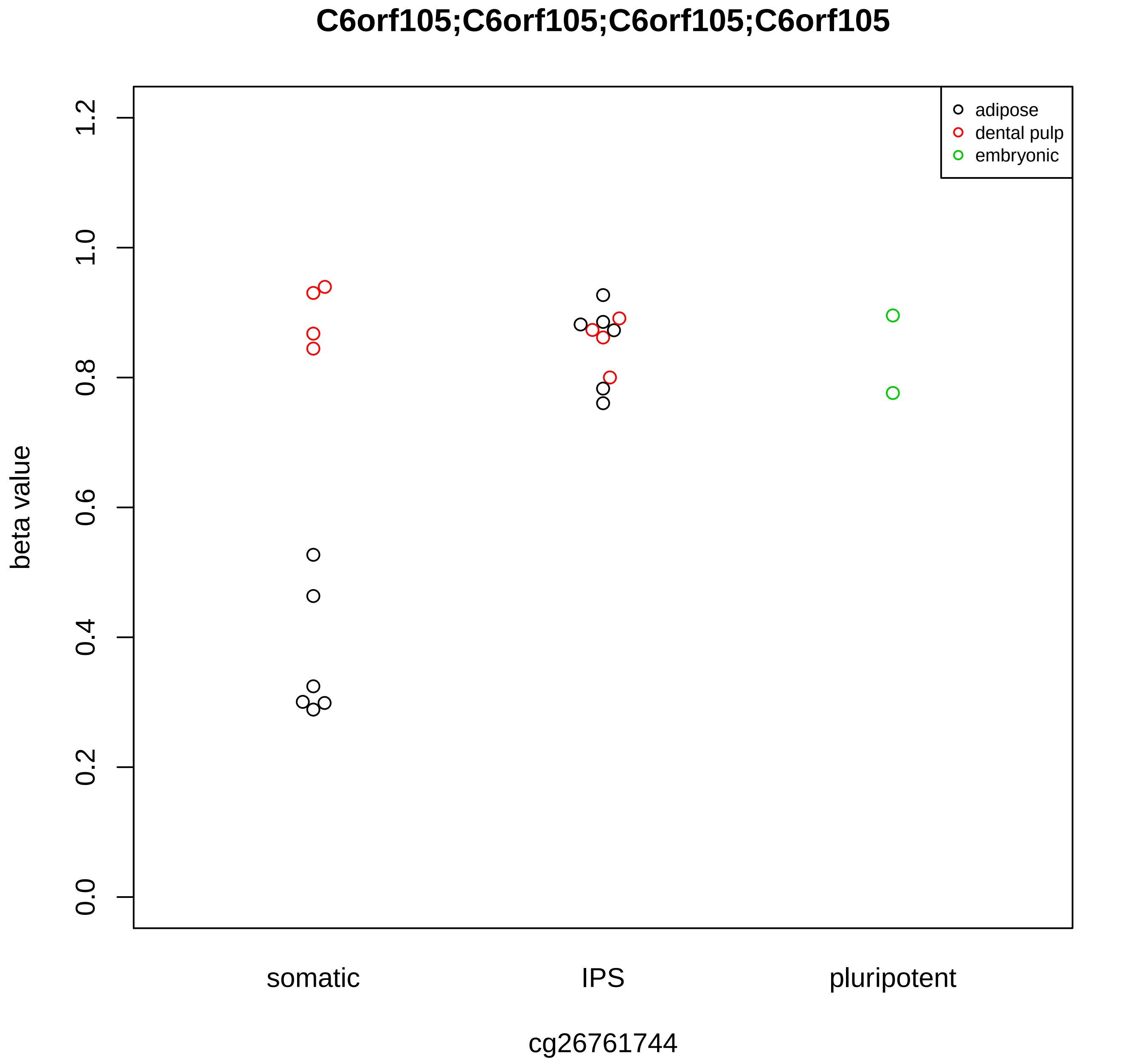
C6orf105;C6orf105;C6orf105;C6orf105
adipose
dental pulp
embryonic
beta value
0.0
0.2
0.4
0.6
0.8
1.0
1.2
somatic
IPS
pluripotent
cg26761744

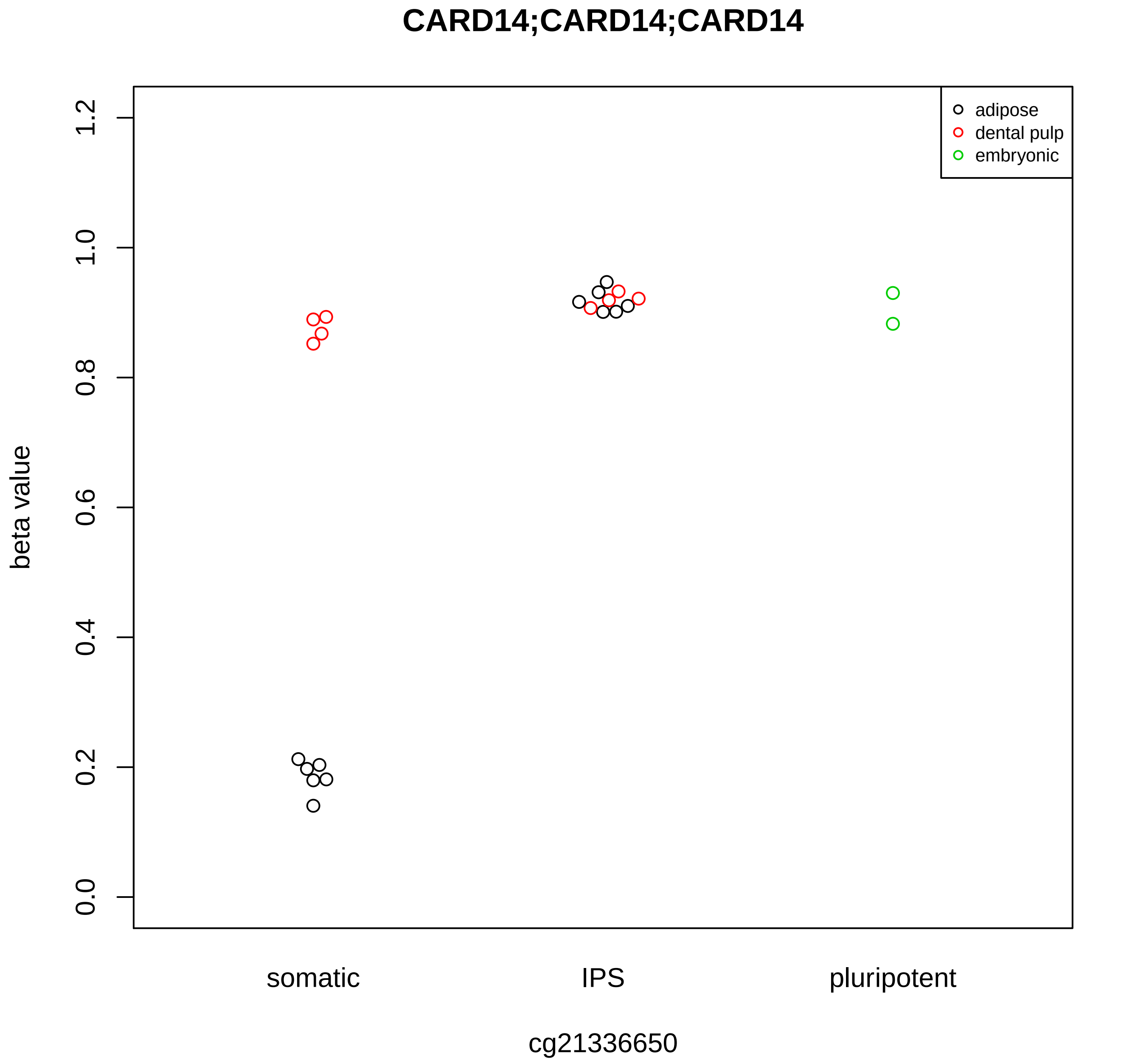

CARD14;CARD14;CARD14
beta value
0.0
0.2
0.4
0.6
0.8
1.0
1.2
adipose
dental pulp
embryonic
somatic
IPS
pluripotent
cg21336650

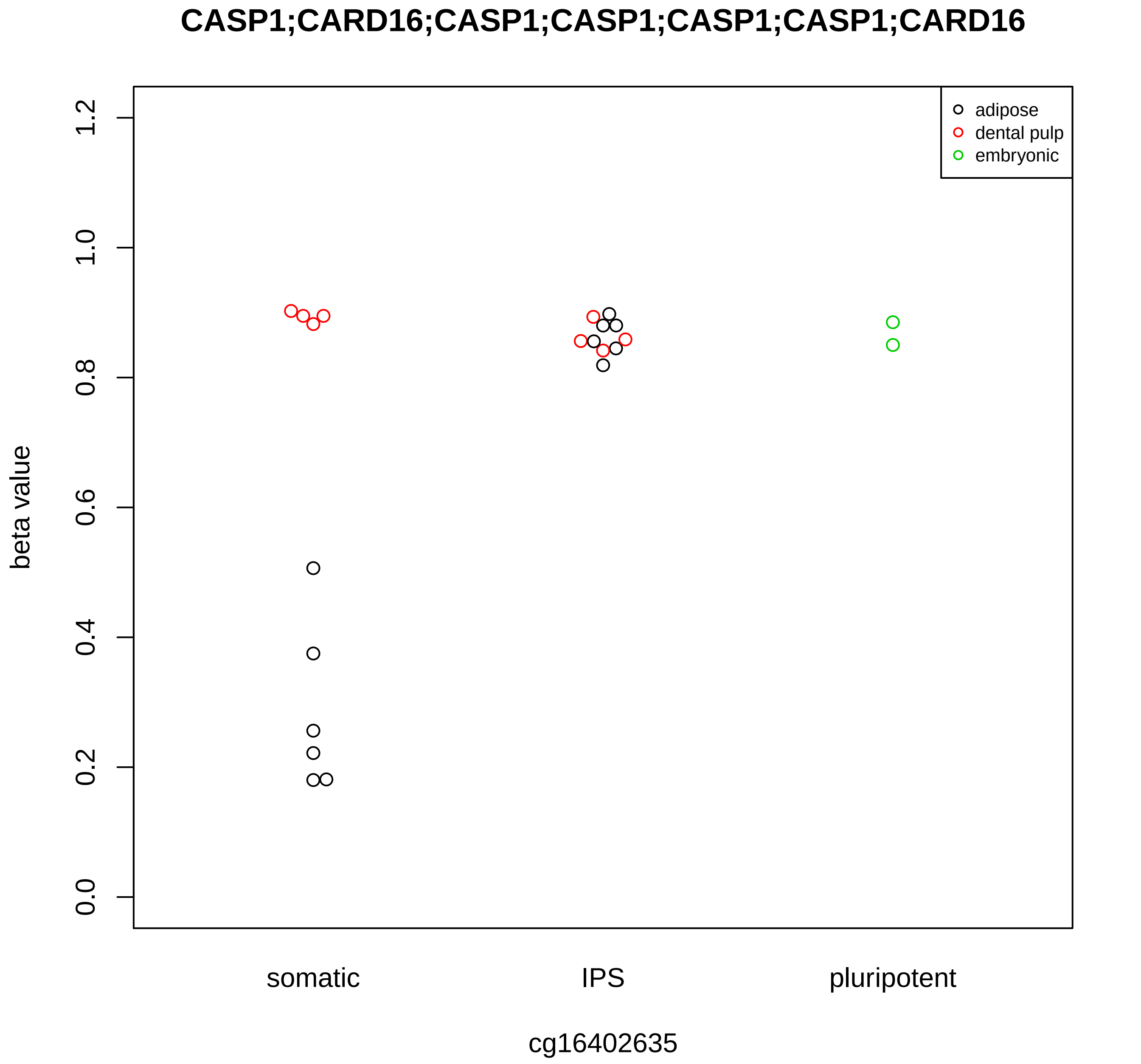

CASP1;CARD16;CASP1;CASP1;CASP1;CASP1;CARD16
beta value
0.0
0.2
0.4
0.6
0.8
1.0
1.2
adipose
dental pulp
embryonic
somatic
IPS
pluripotent
cg16402635

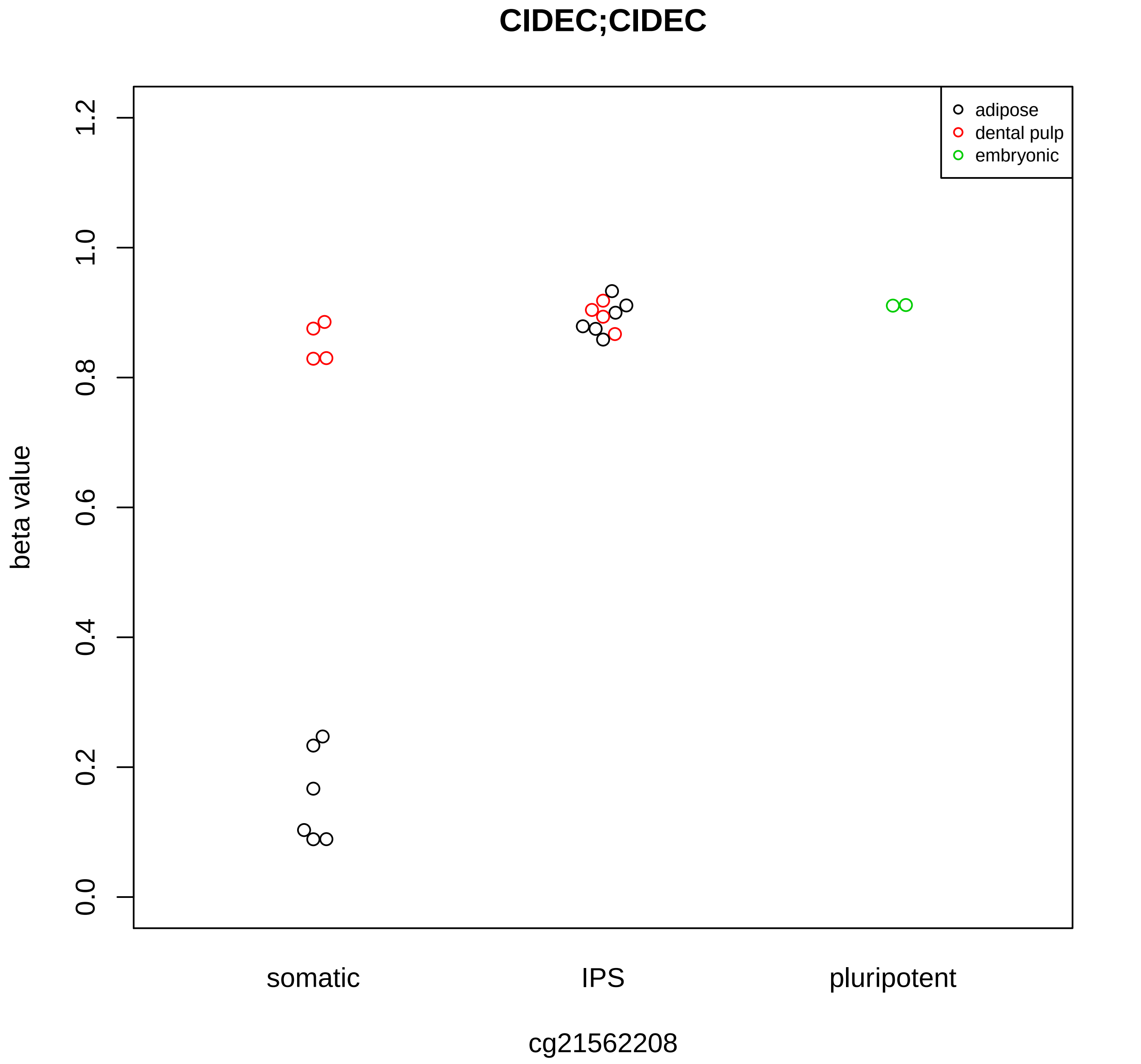
CIDEC;CIDEC
beta value
0.0
0.2
0.4
0.6
0.8
1.0
1.2
adipose
dental pulp
embryonic
somatic
IPS
pluripotent
cg21562208

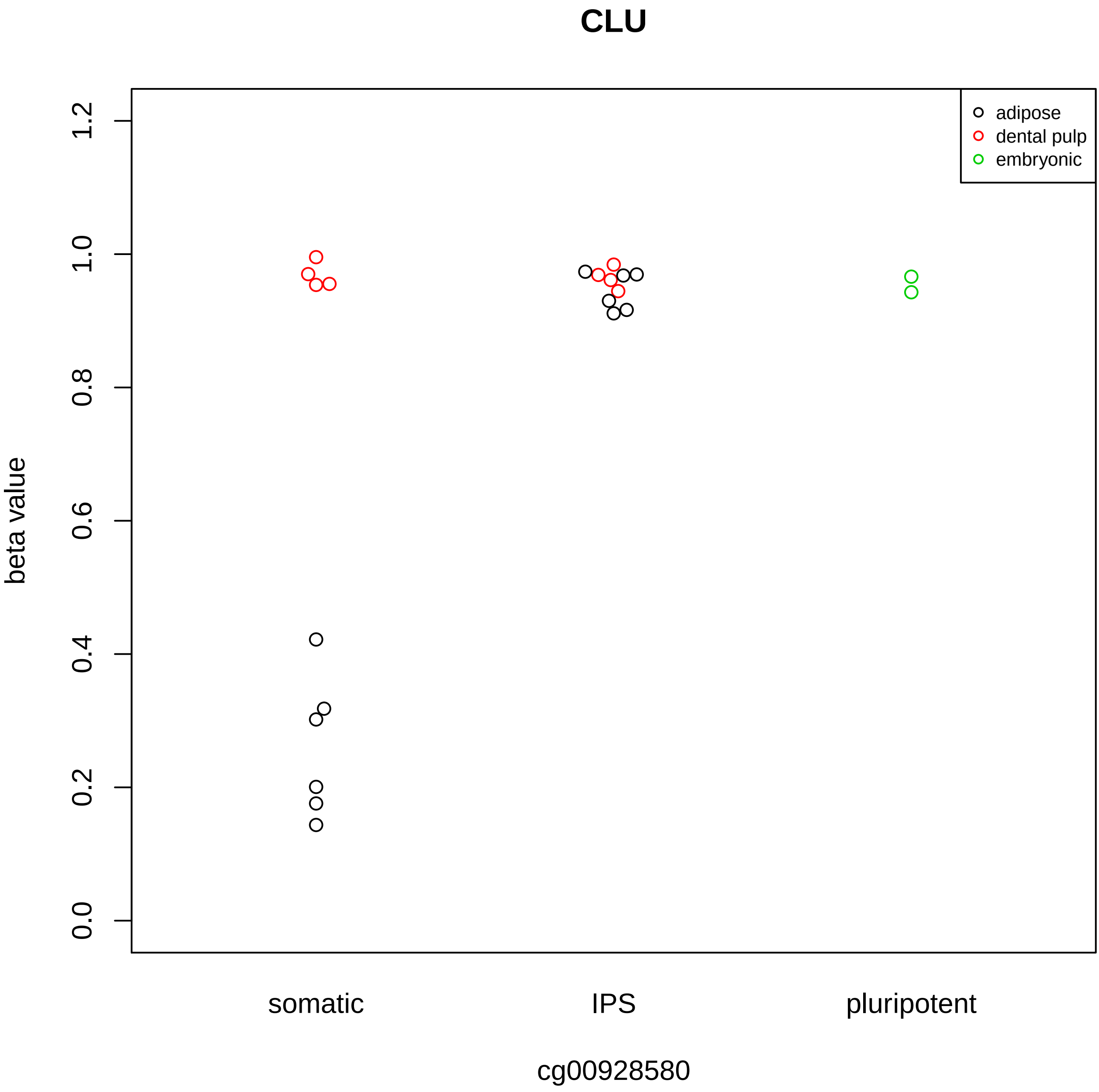
CLU
adipose
dental pulp
embryonic
beta value
0.0
0.2
0.4
0.6
0.8
1.0
1.2
somatic
IPS
pluripotent
cg00928580

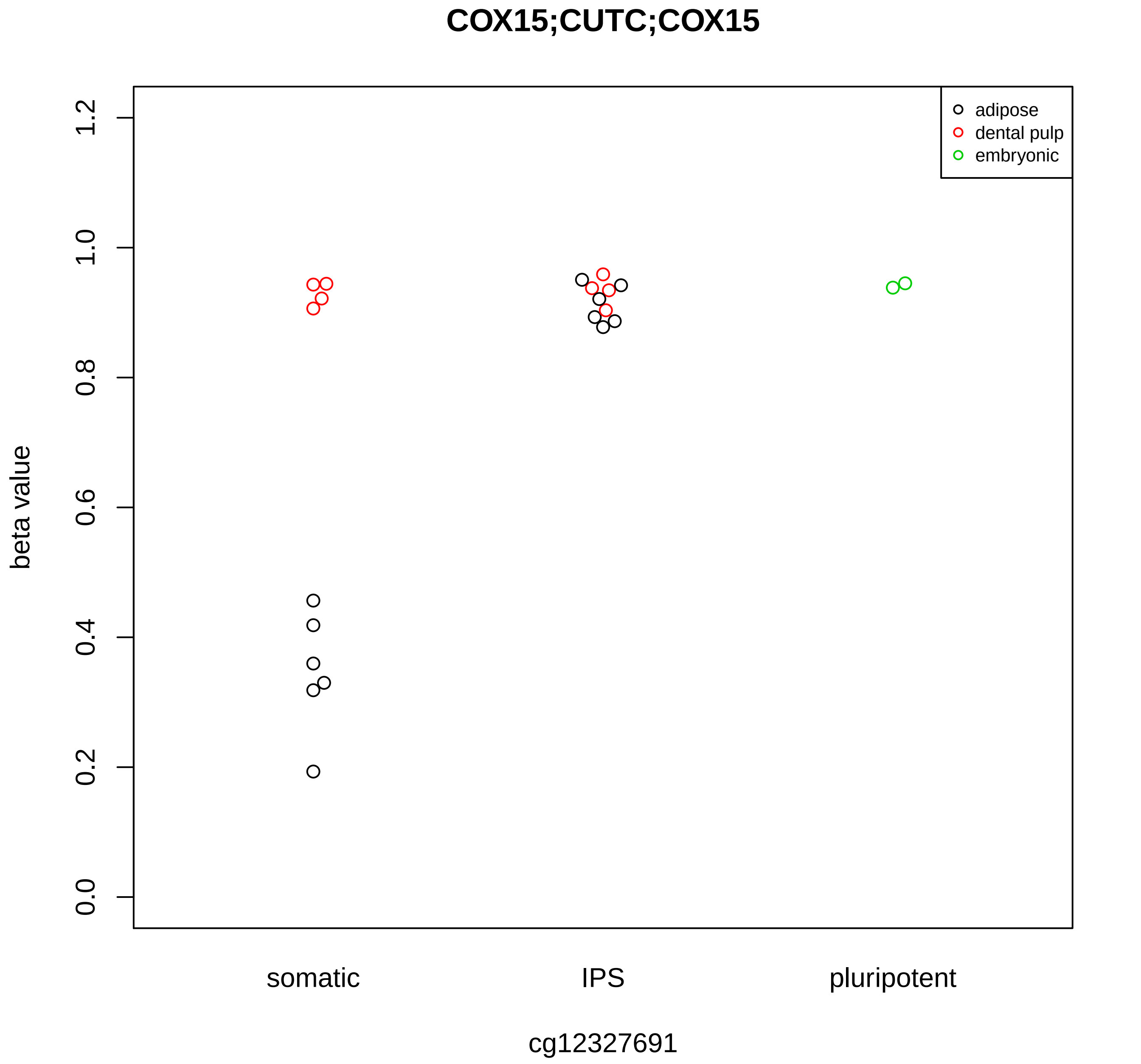
COX15;CUTC;COX15
beta value
0.0
0.2
0.4
0.6
0.8
1.0
1.2
adipose
dental pulp
embryonic
somatic
IPS
pluripotent
cg12327691

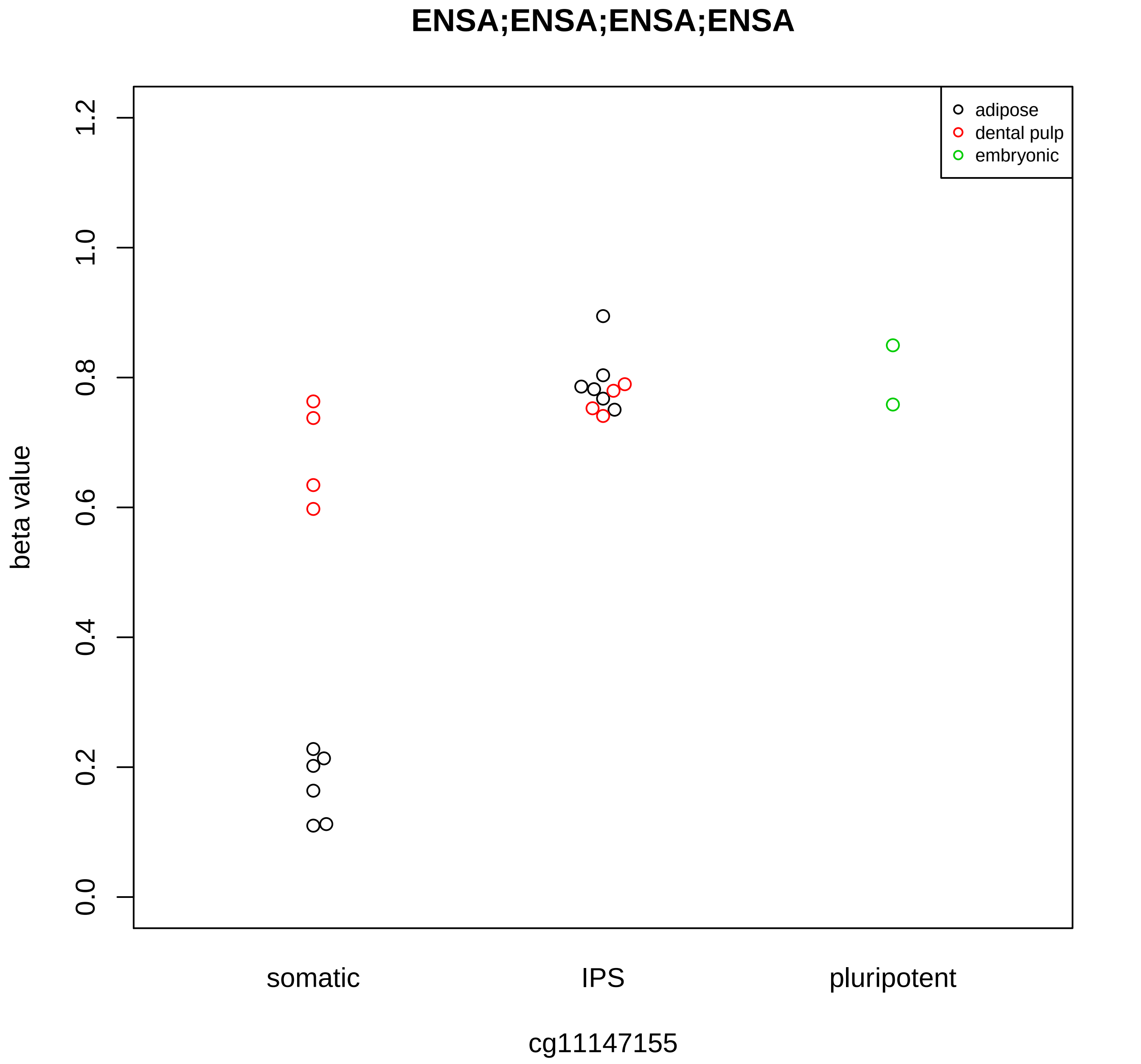

ENSA;ENSA;ENSA;ENSA
beta value
0.0
0.2
0.4
0.6
0.8
1.0
1.2
adipose
dental pulp
embryonic
somatic
IPS
pluripotent
cg11147155

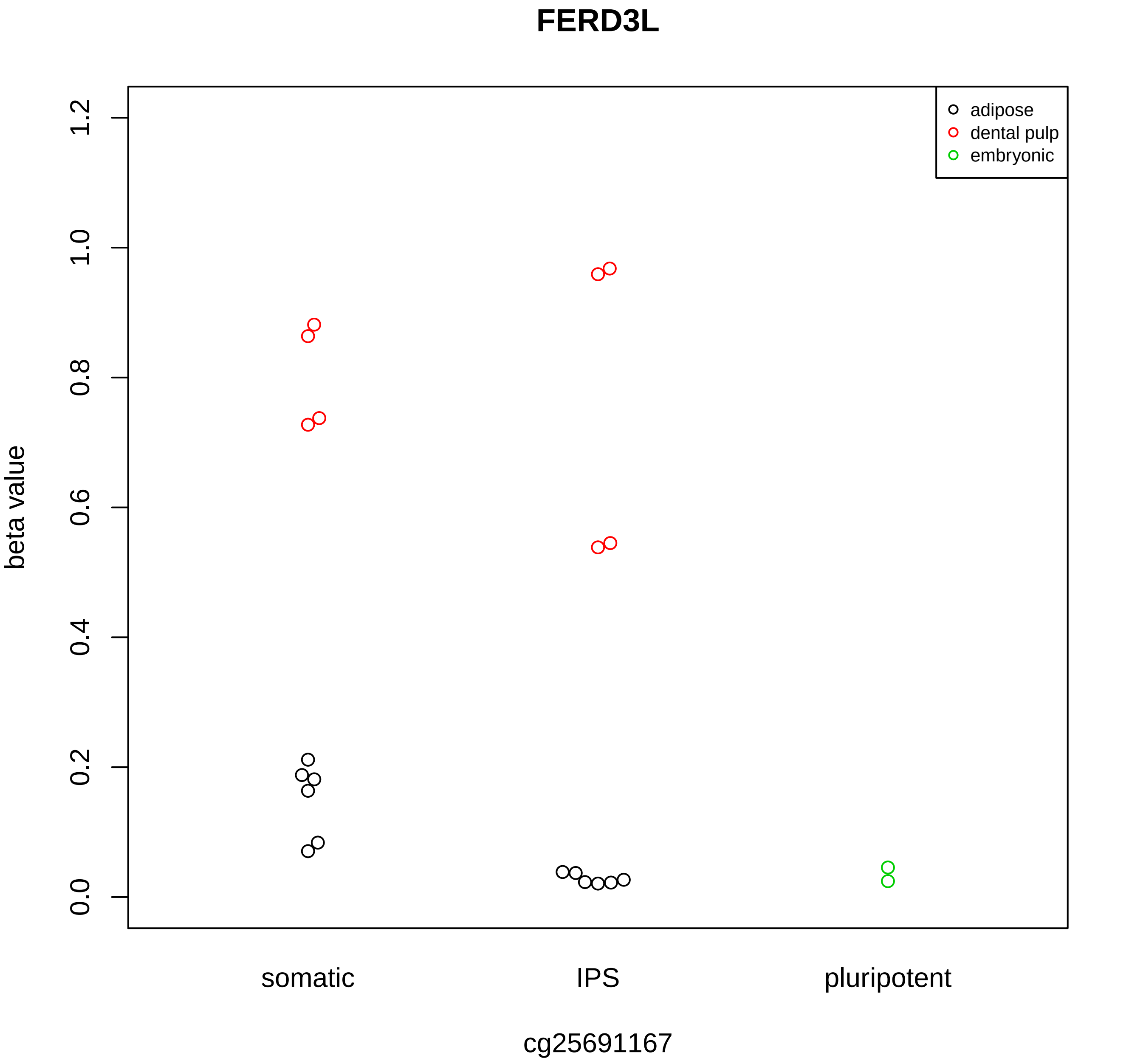
FERD3L
adipose
dental pulp
embryonic
beta value
0.0
0.2
0.4
0.6
0.8
1.0
1.2
somatic
IPS
pluripotent
cg25691167

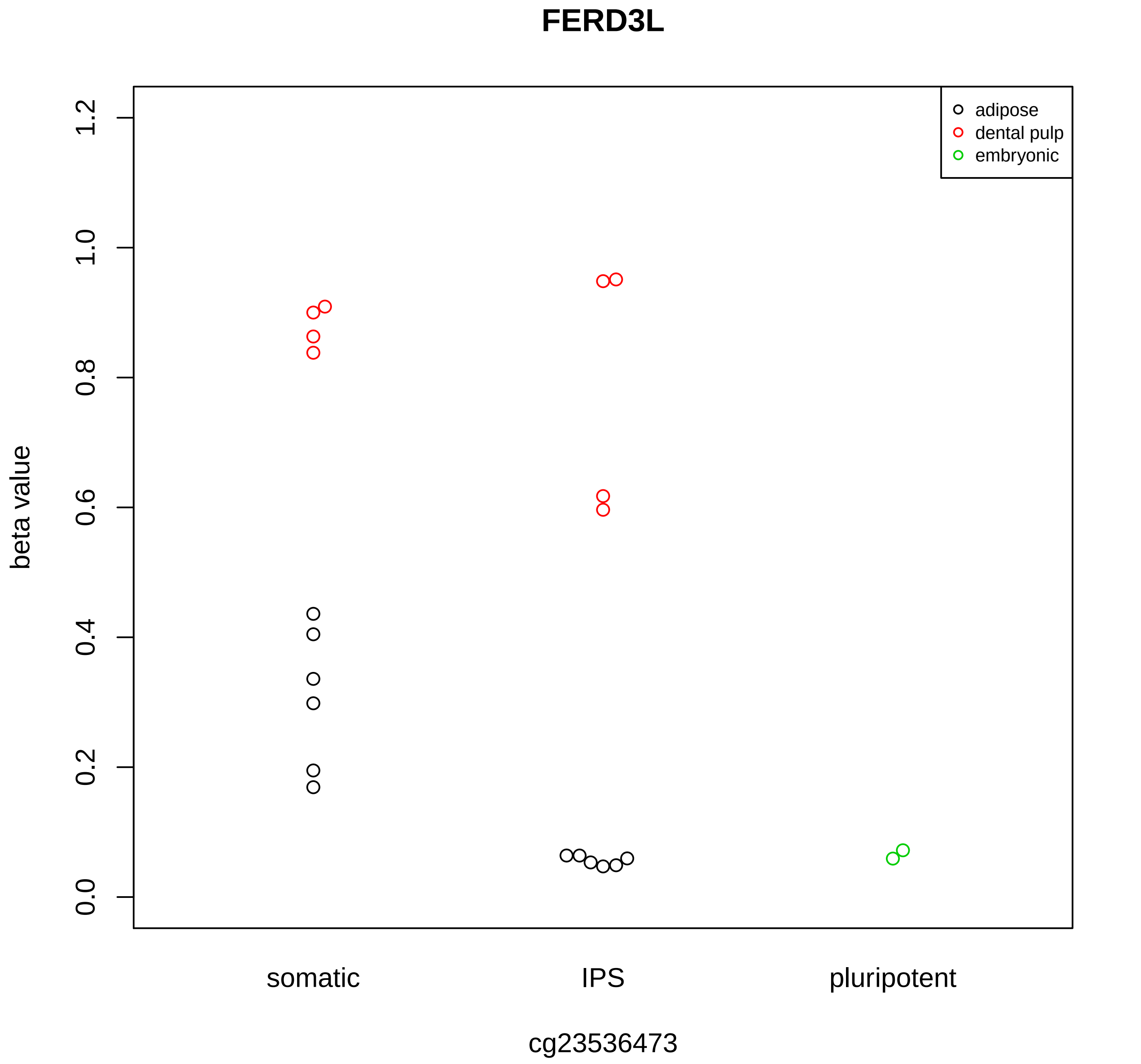
FERD3L
beta value
0.0
0.2
0.4
0.6
0.8
1.0
1.2
adipose
dental pulp
embryonic
somatic
IPS
pluripotent
cg23536473

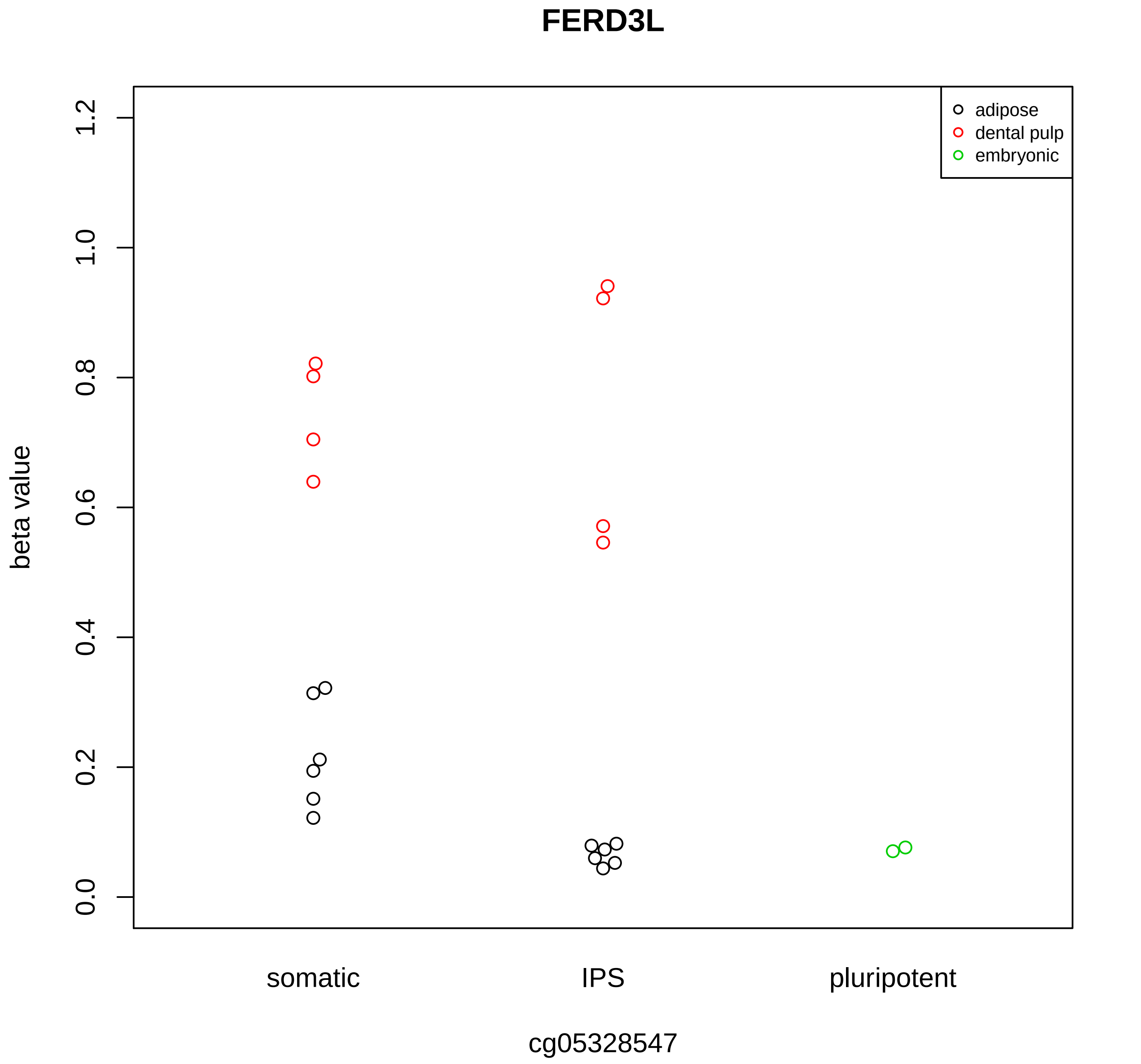
FERD3L
beta value
0.0
0.2
0.4
0.6
0.8
1.0
1.2
adipose
dental pulp
embryonic
somatic
IPS
pluripotent
cg05328547

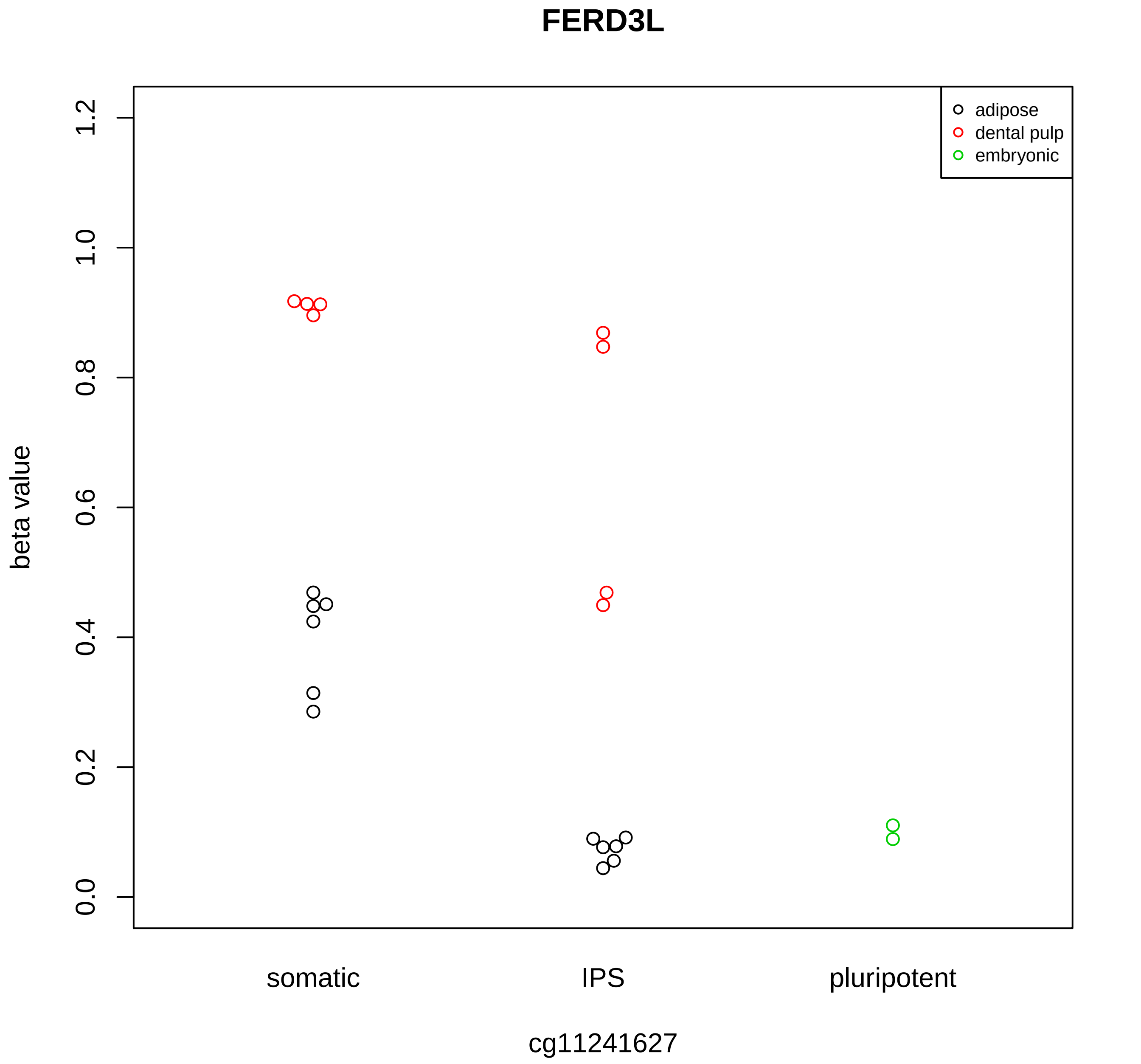
FERD3L
adipose
dental pulp
embryonic
beta value
0.0
0.2
0.4
0.6
0.8
1.0
1.2
somatic
IPS
pluripotent
cg11241627

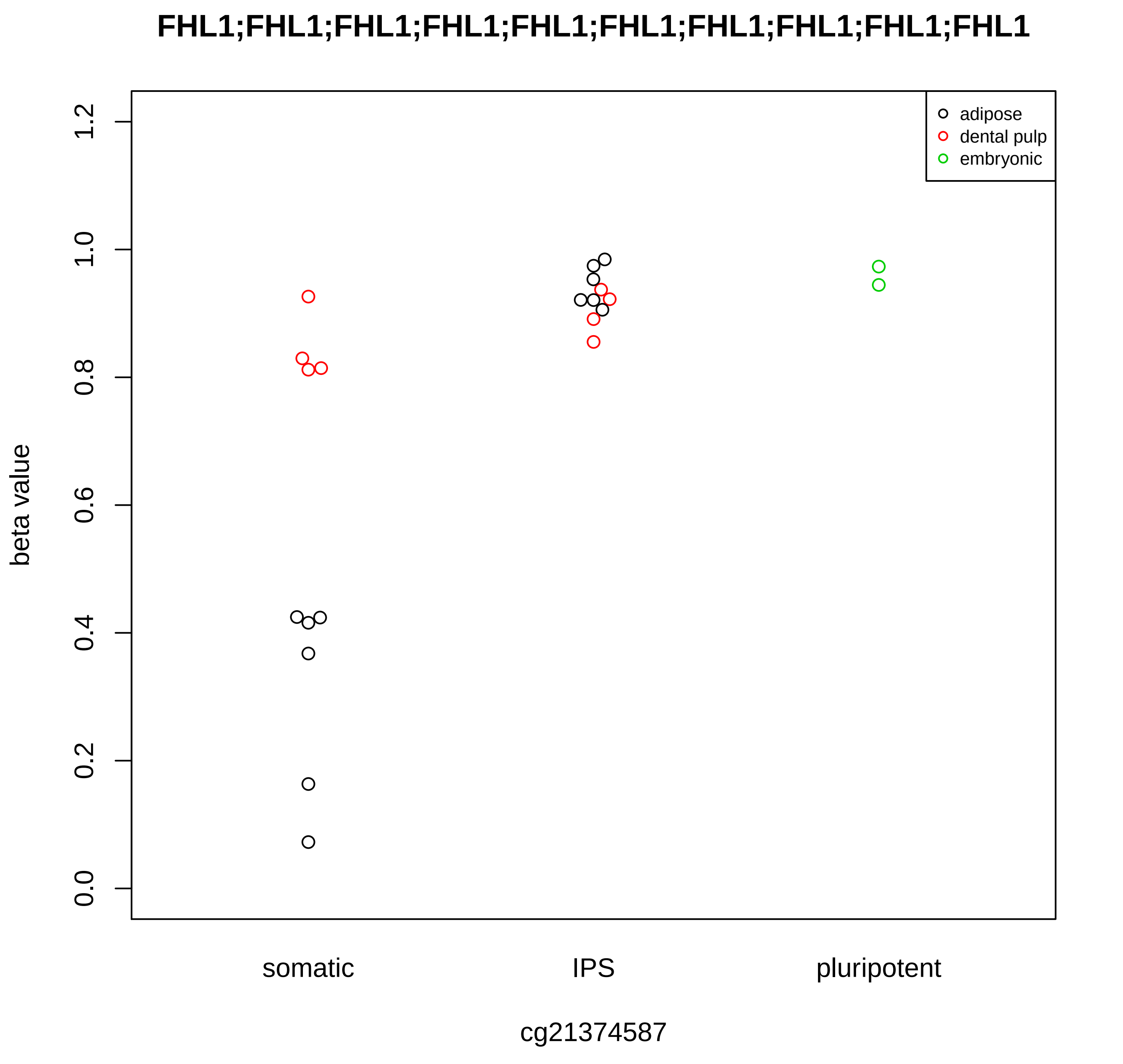

FHL1;FHL1;FHL1;FHL1;FHL1;FHL1;FHL1;FHL1;FHL1;FHL1
beta value
0.0
0.2
0.4
0.6
0.8
1.0
1.2
adipose
dental pulp
embryonic
somatic
IPS
pluripotent
cg21374587

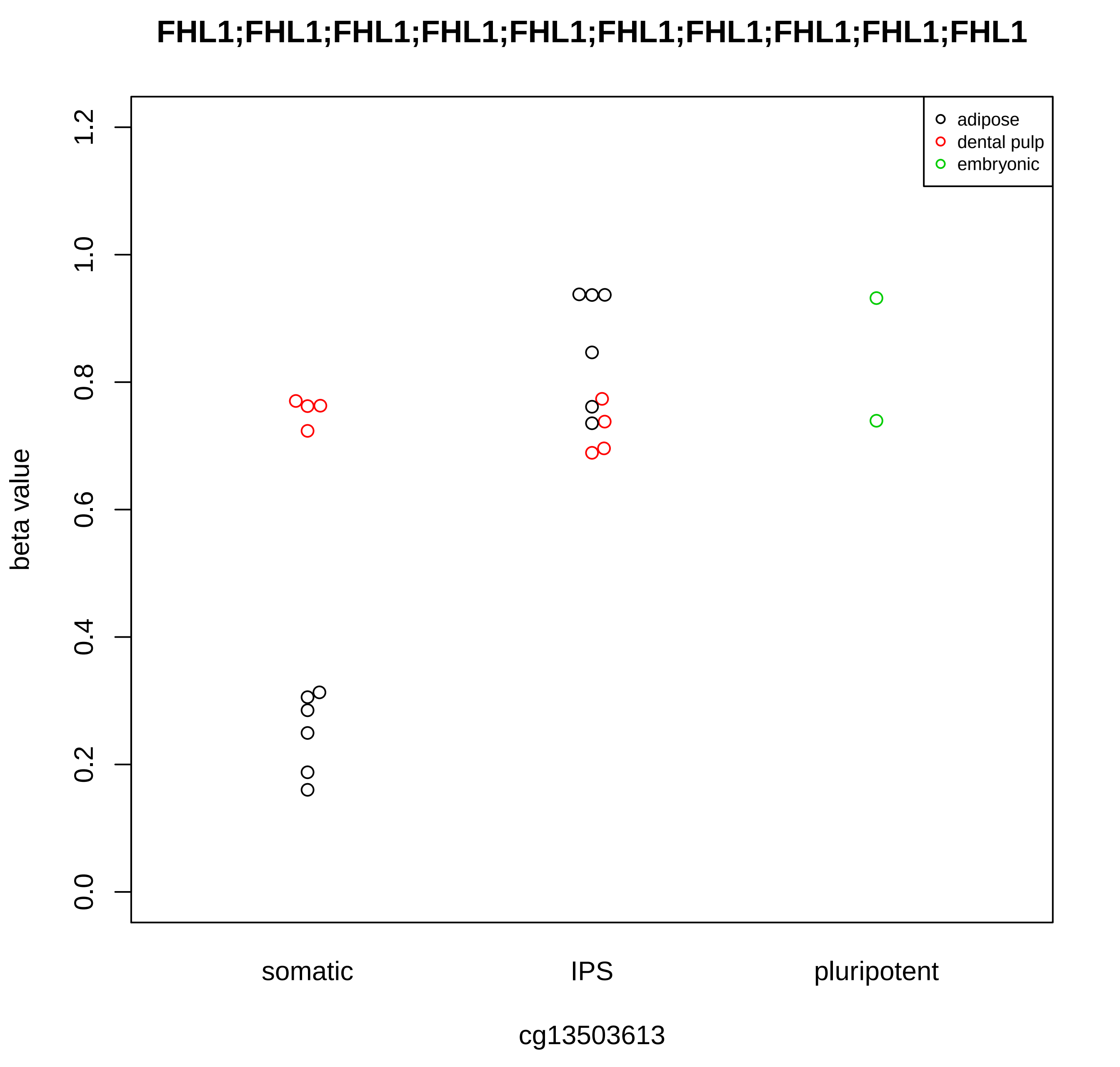

FHL1;FHL1;FHL1;FHL1;FHL1;FHL1;FHL1;FHL1;FHL1;FHL1
adipose
dental pulp
embryonic
beta value
0.0
0.2
0.4
0.6
0.8
1.0
1.2
somatic
IPS
pluripotent
cg13503613

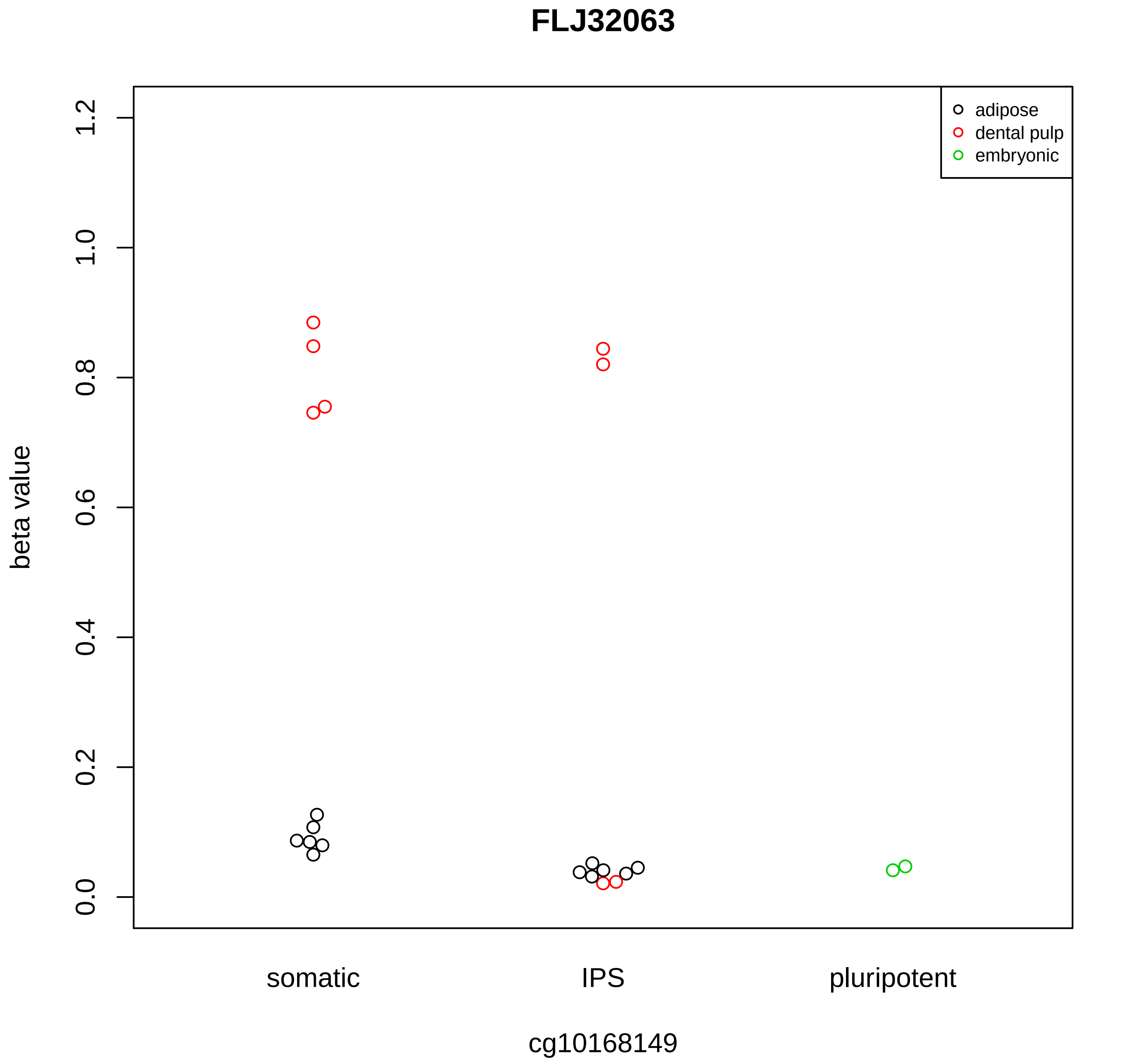

FLJ32063
beta value
0.0
0.2
0.4
0.6
0.8
1.0
1.2
adipose
dental pulp
embryonic
somatic
IPS
pluripotent
cg10168149

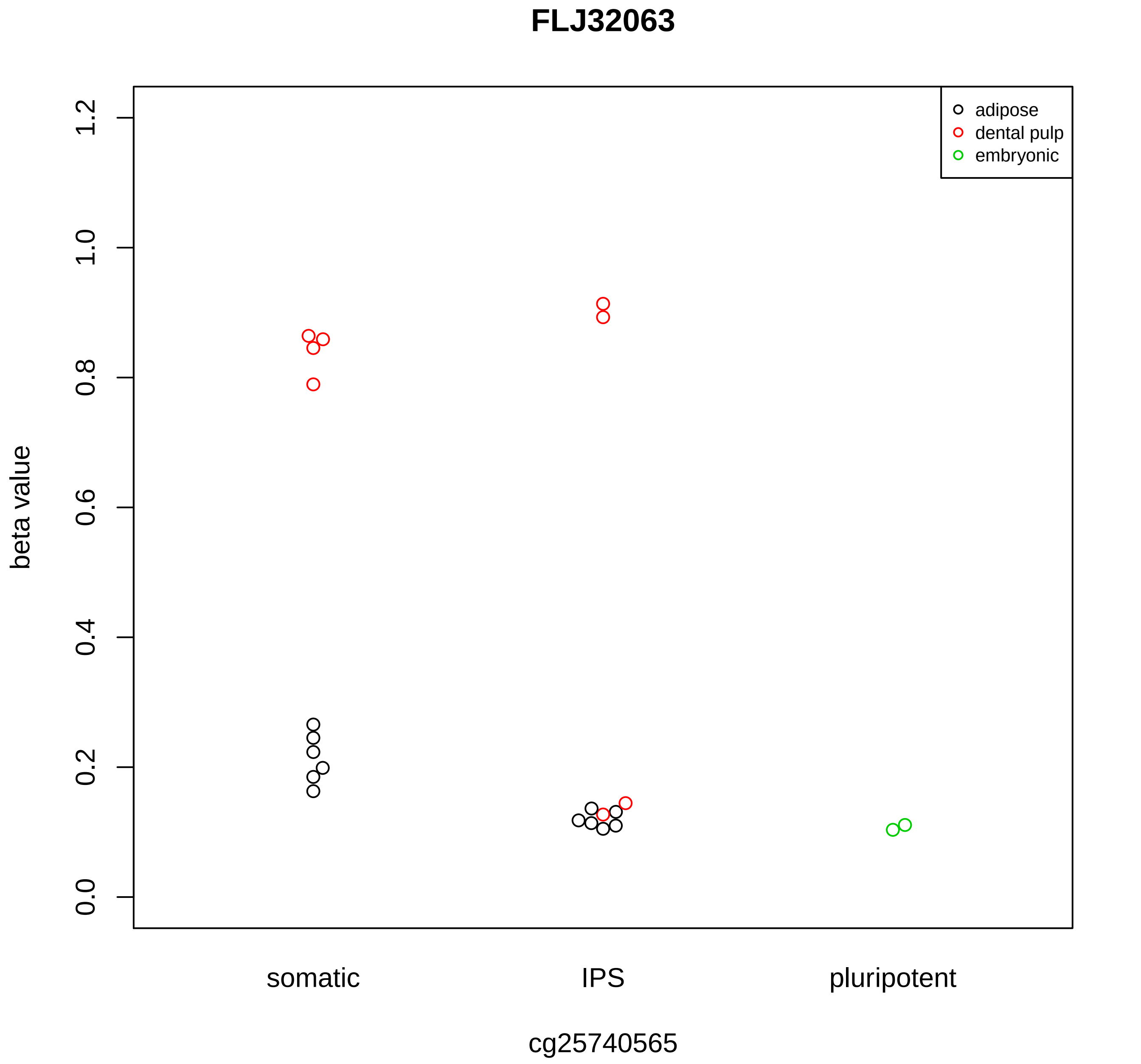
FLJ32063
adipose
dental pulp
embryonic
beta value
1.2
1.0
0.8
0.6
0.4
0.2
0.0
somatic
IPS
pluripotent
cg25740565

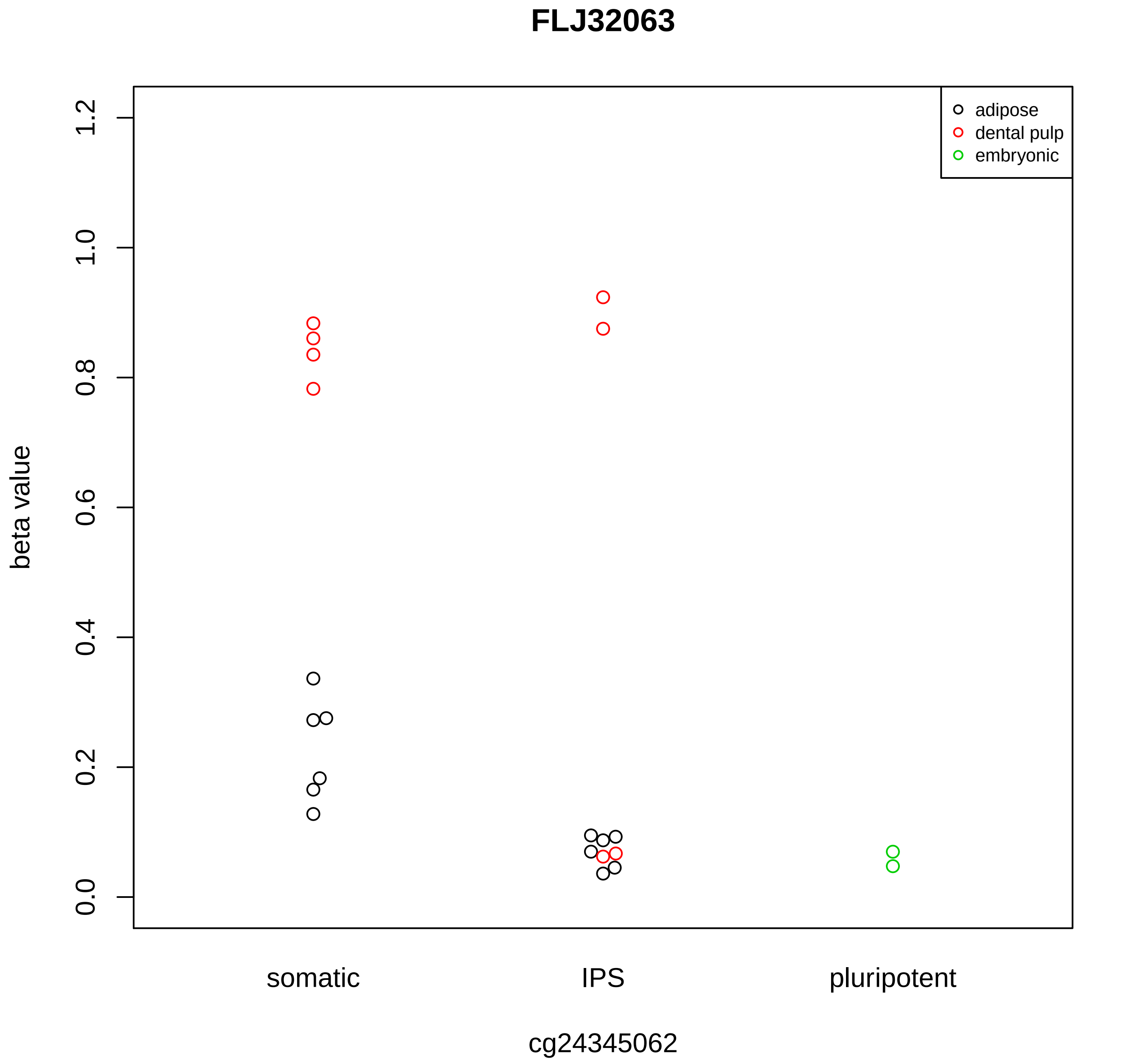
FLJ32063
beta value
0.0
0.2
0.4
0.6
0.8
1.0
1.2
adipose
dental pulp
embryonic
somatic
IPS
pluripotent
cg24345062

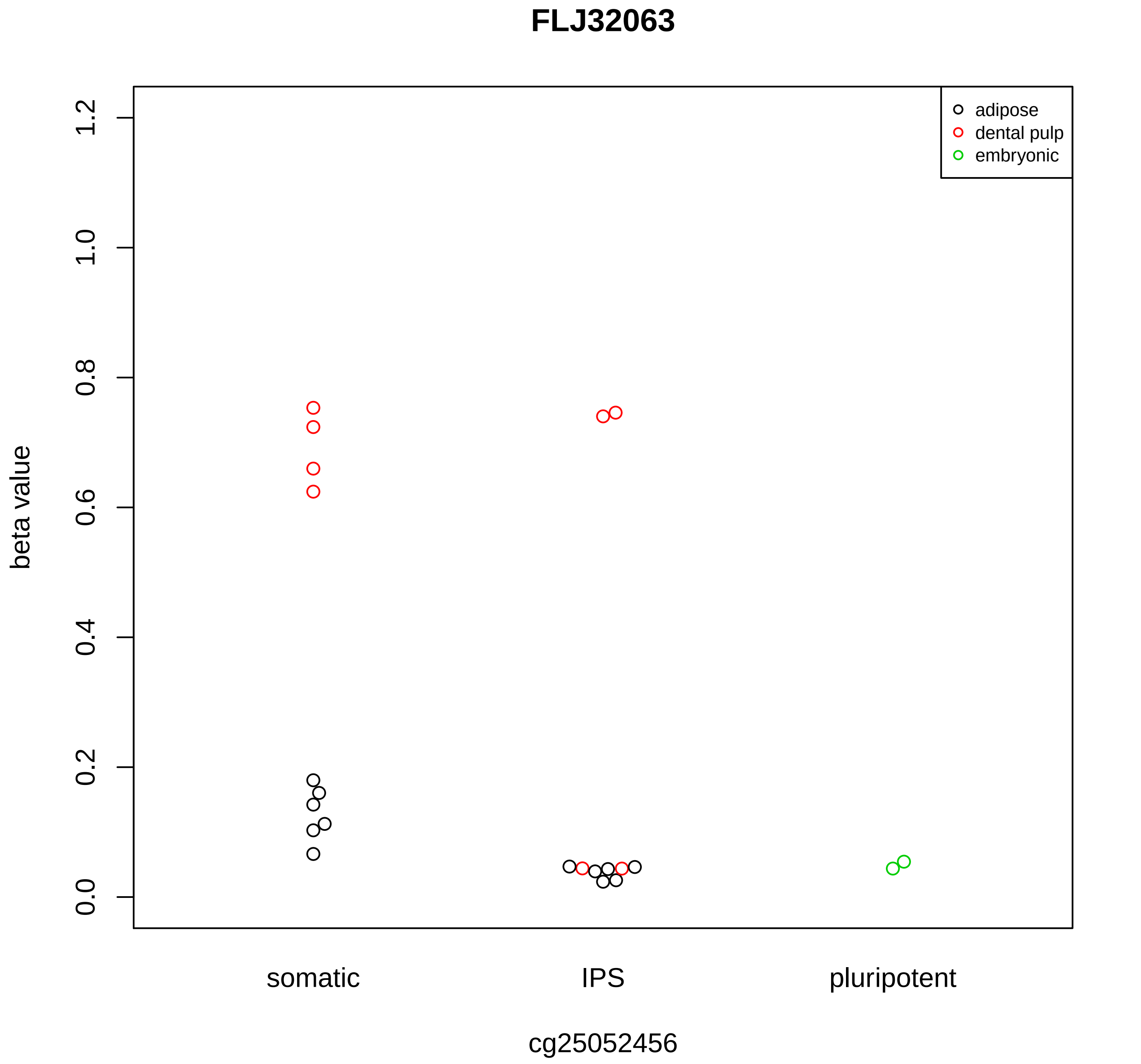

FLJ32063
adipose
dental pulp
embryonic
beta value
0.0
0.2
0.4
0.6
0.8
1.0
1.2
somatic
IPS
pluripotent
cg25052456

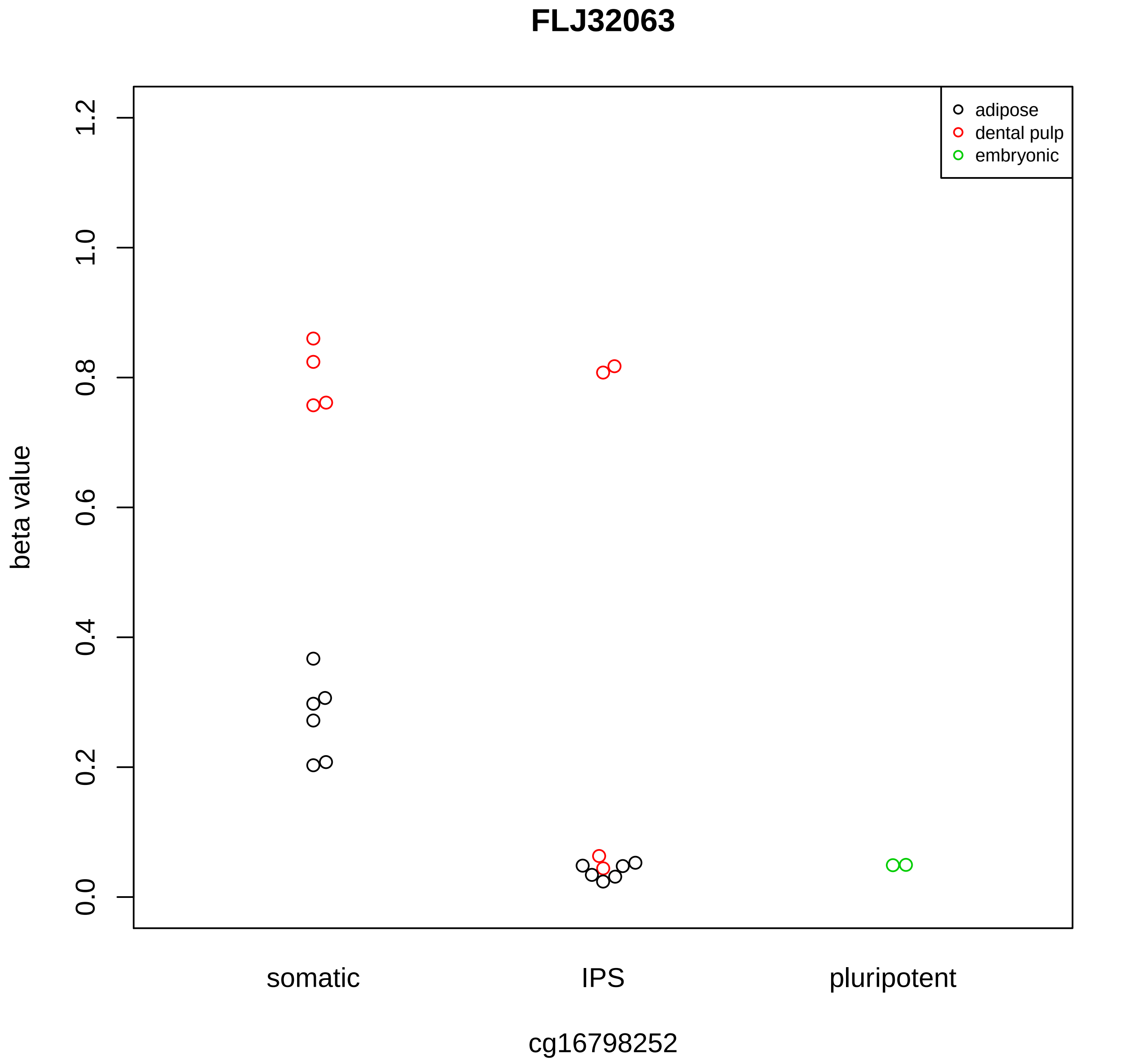
FLJ32063
beta value
1.2
1.0
0.8
0.6
0.4
0.2
0.0
adipose
dental pulp
embryonic
somatic
IPS
pluripotent
cg16798252

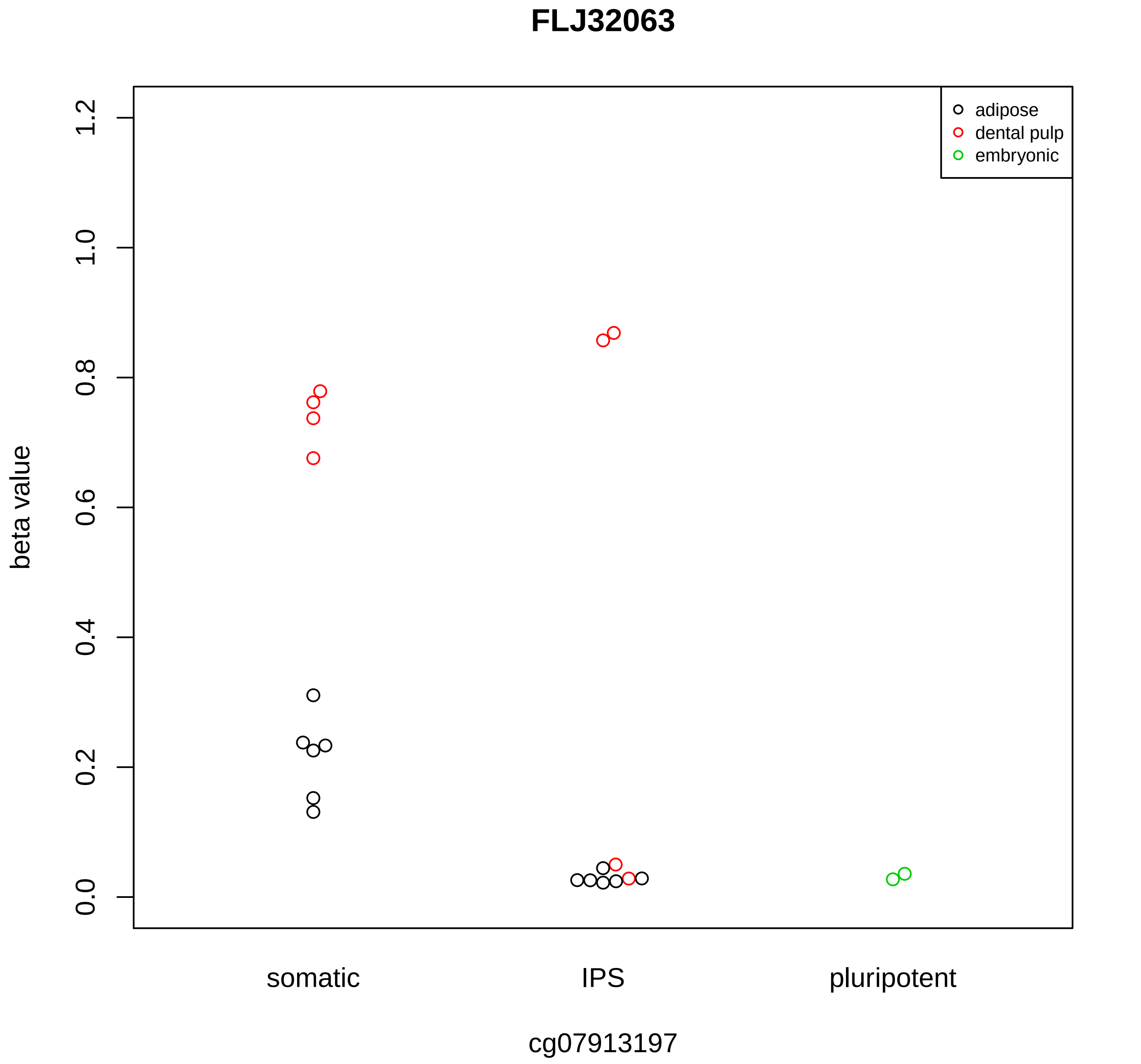
FLJ32063
beta value
0.0
0.2
0.4
0.6
0.8
1.0
1.2
adipose
dental pulp
embryonic
somatic
IPS
pluripotent
cg07913197

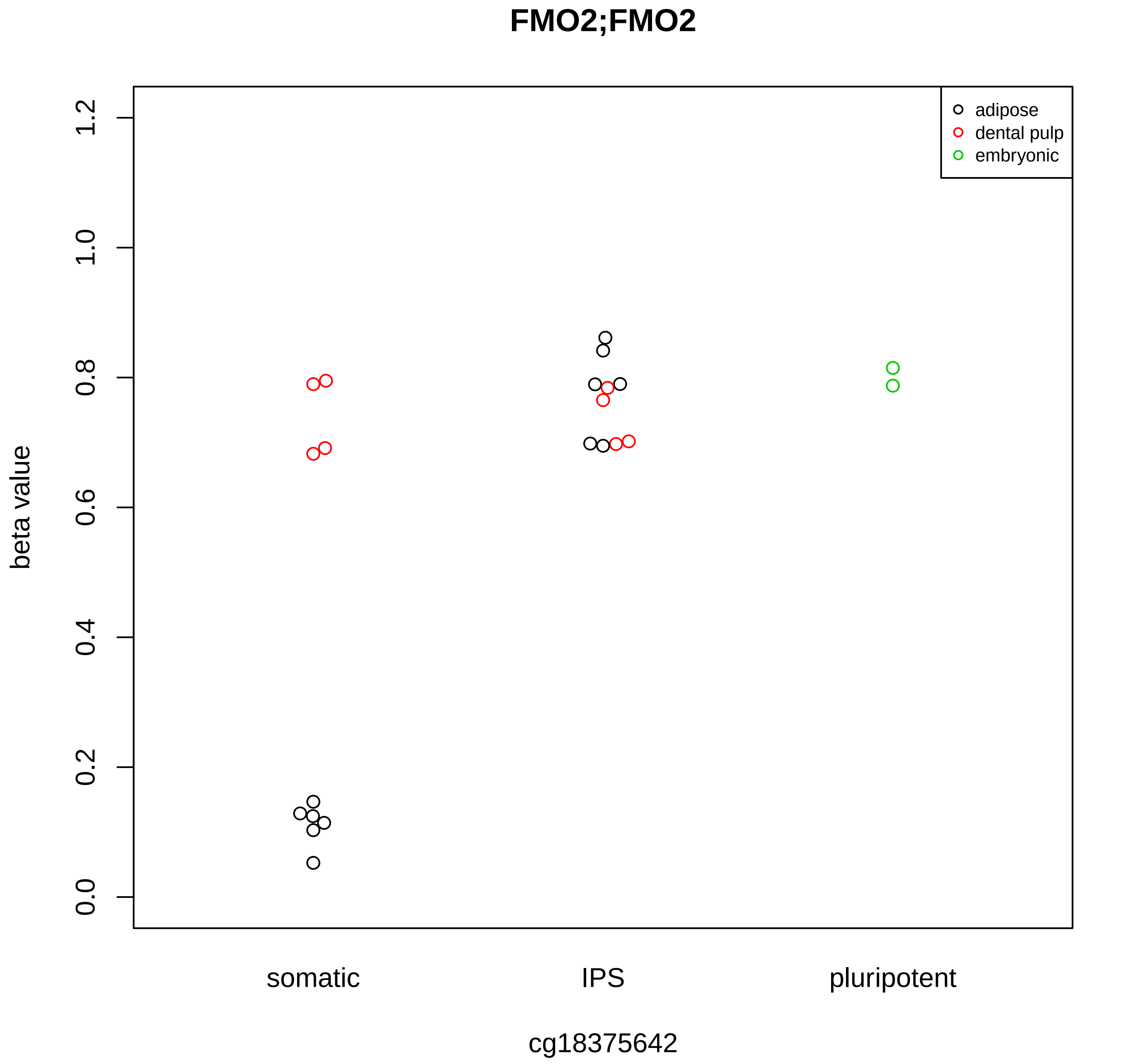

FMO2;FMO2
adipose
dental pulp
embryonic
beta value
0.0
0.2
0.4
0.6
0.8
1.0
1.2
somatic
IPS
pluripotent
cg18375642

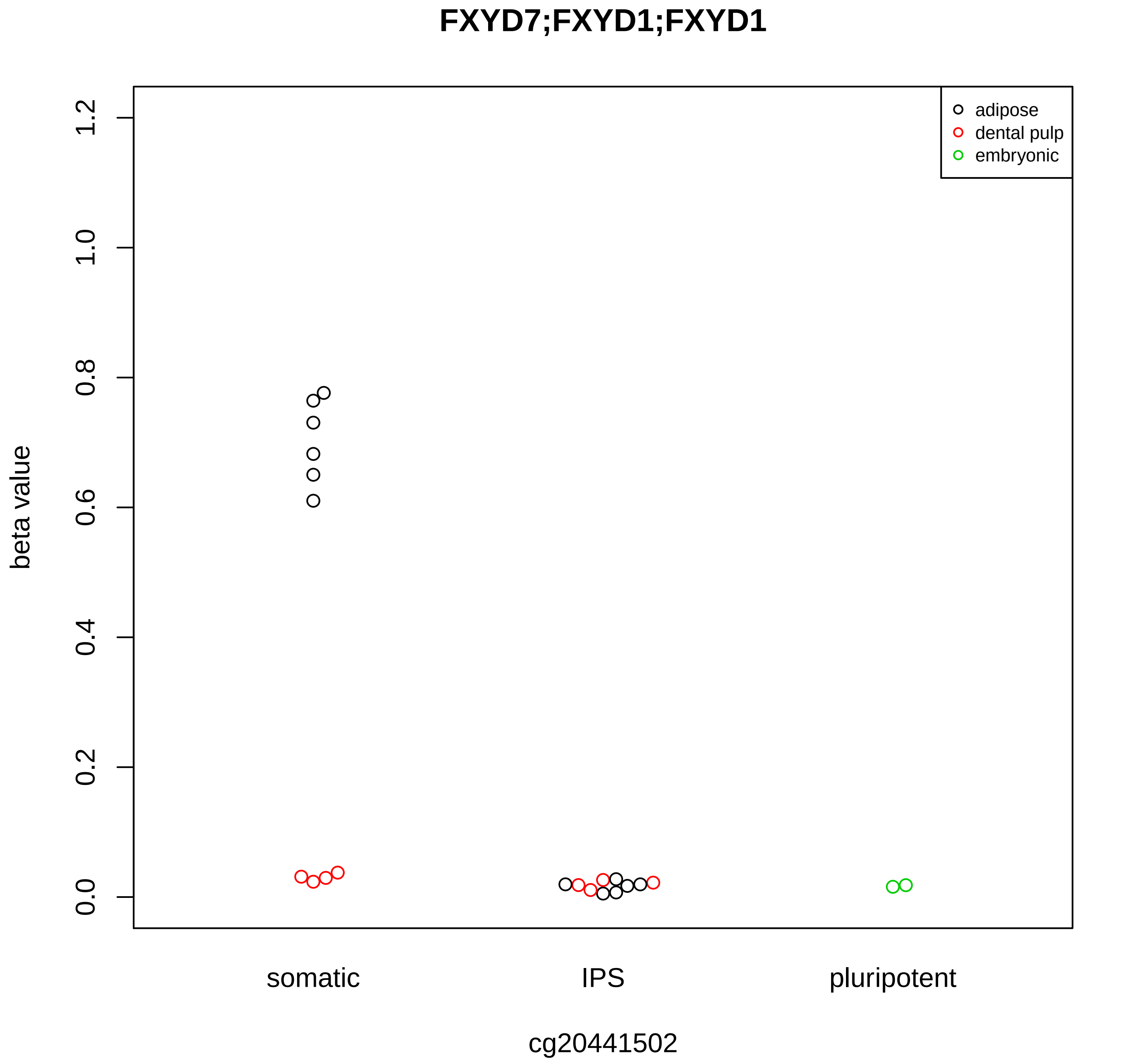
FXYD7;FXYD1;FXYD1
beta value
1.2
1.0
0.8
0.6
0.4
0.2
0.0
adipose
dental pulp
embryonic
somatic
IPS
pluripotent
cg20441502

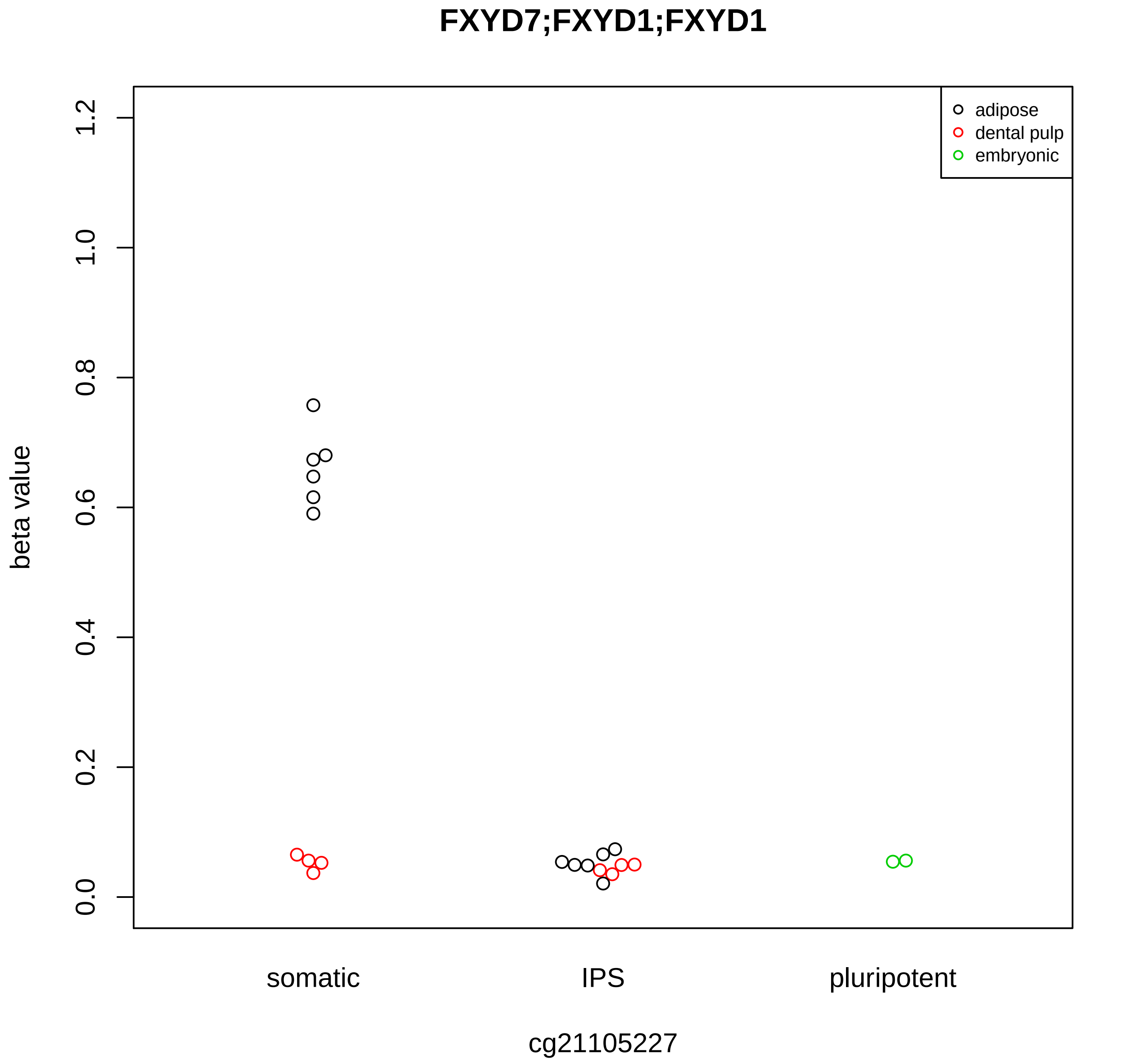
FXYD7;FXYD1;FXYD1
beta value
0.0
0.2
0.4
0.6
0.8
1.0
1.2
adipose
dental pulp
embryonic
somatic
IPS
pluripotent
cg21105227

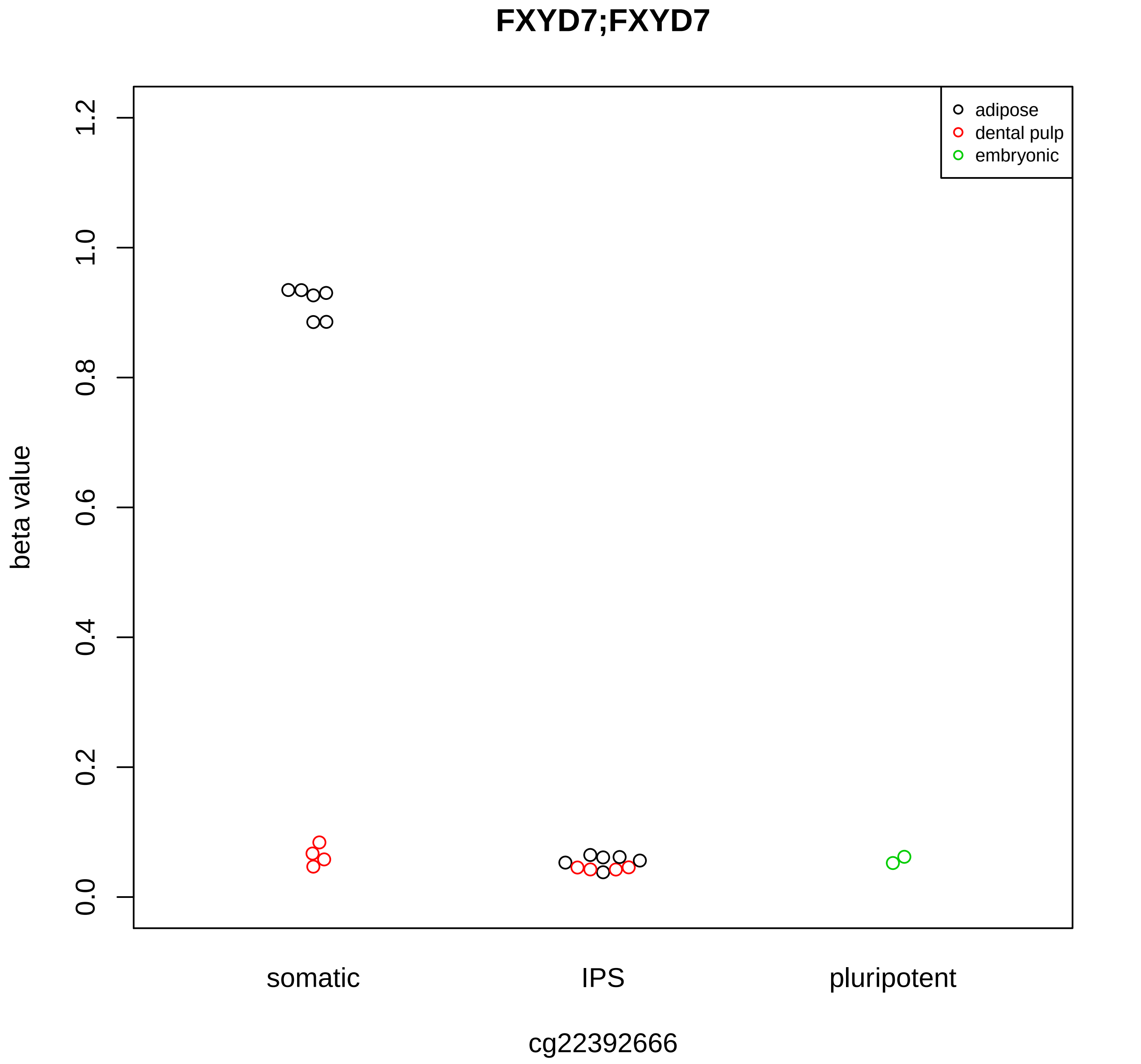
FXYD7;FXYD7
adipose
dental pulp
embryonic
beta value
1.2
1.0
0.8
0.6
0.4
0.2
0.0
somatic
IPS
pluripotent
cg22392666

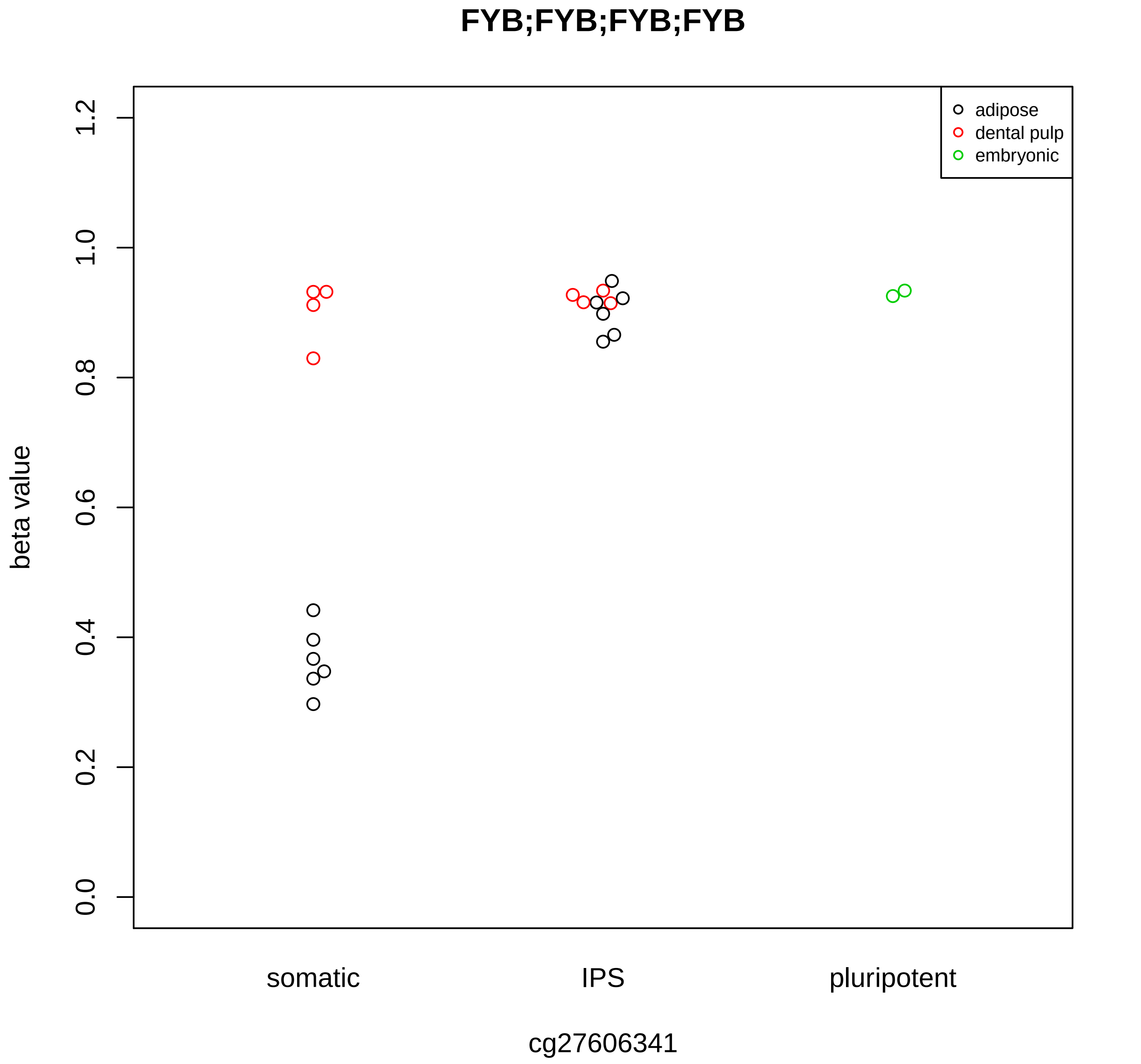

FYB;FYB;FYB;FYB
beta value
0.0
0.2
0.4
0.6
0.8
1.0
1.2
adipose
dental pulp
embryonic
somatic
IPS
pluripotent
cg27606341

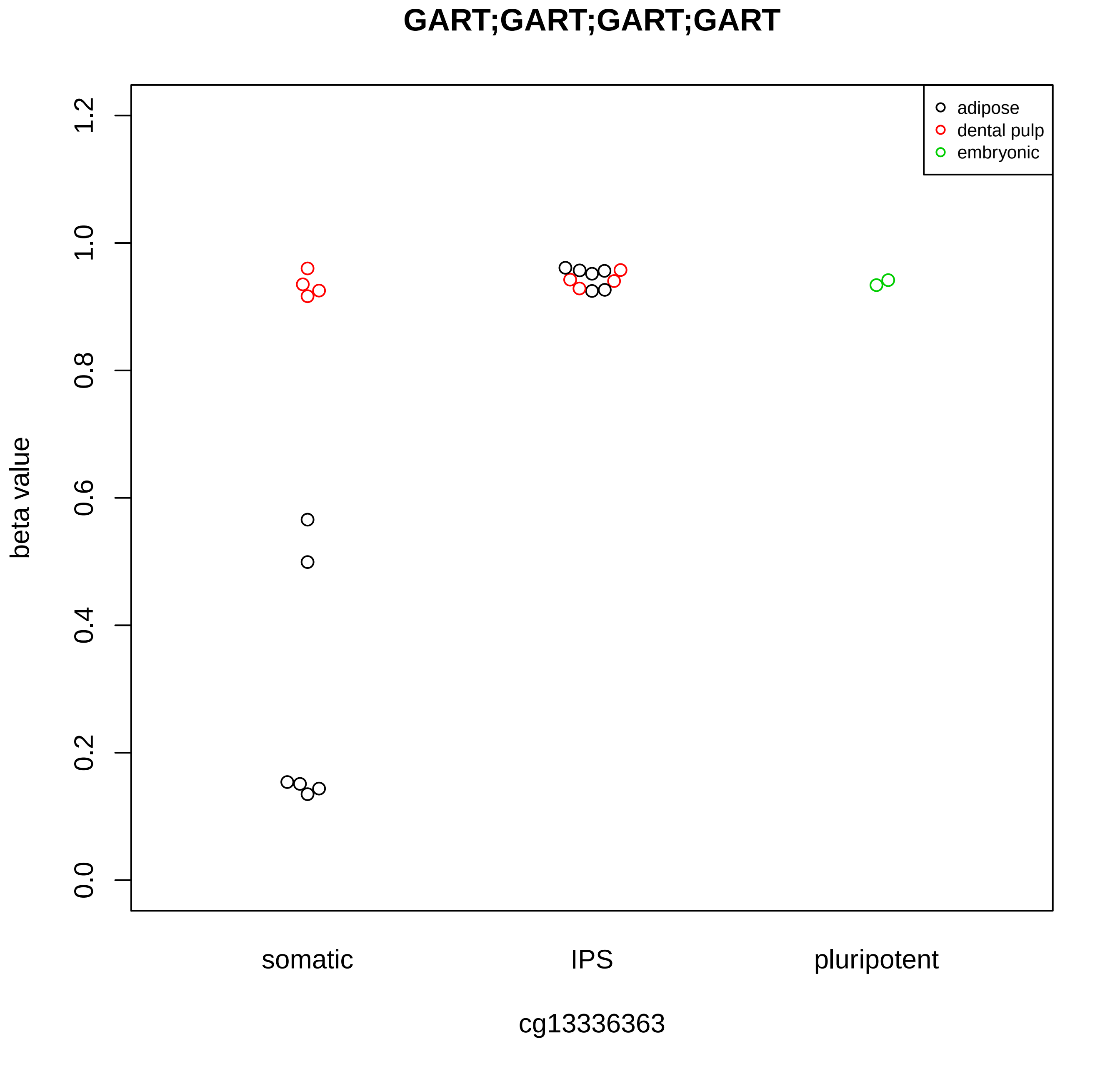
GART;GART;GART;GART
adipose
dental pulp
embryonic
beta value
0.0
0.2
0.4
0.6
0.8
1.0
1.2
somatic
IPS
pluripotent
cg13336363

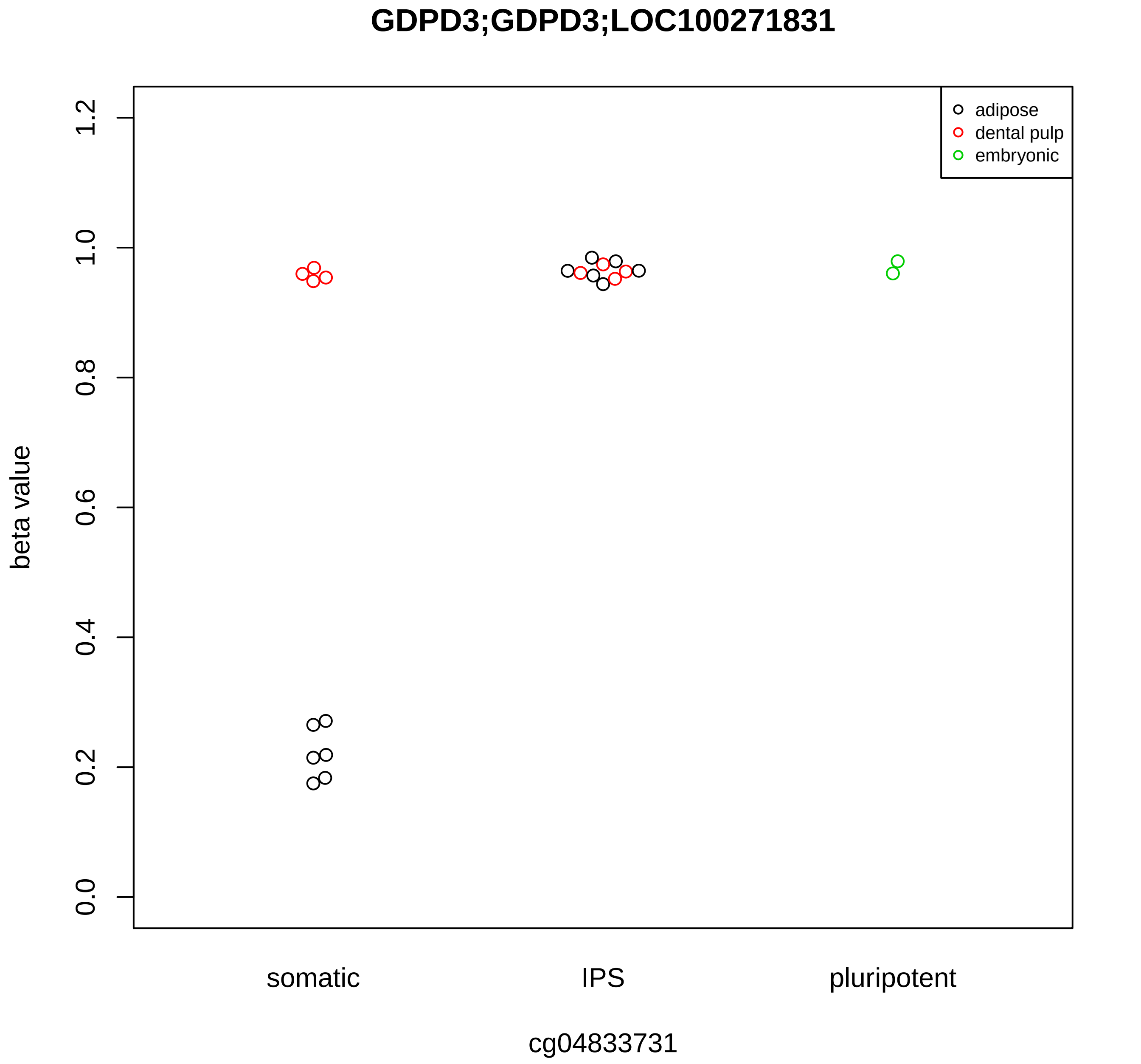

GDPD3;GDPD3;LOC100271831
adipose
dental pulp
embryonic
beta value
0.0
0.2
0.4
0.6
0.8
1.0
1.2
somatic
IPS
pluripotent
cg04833731

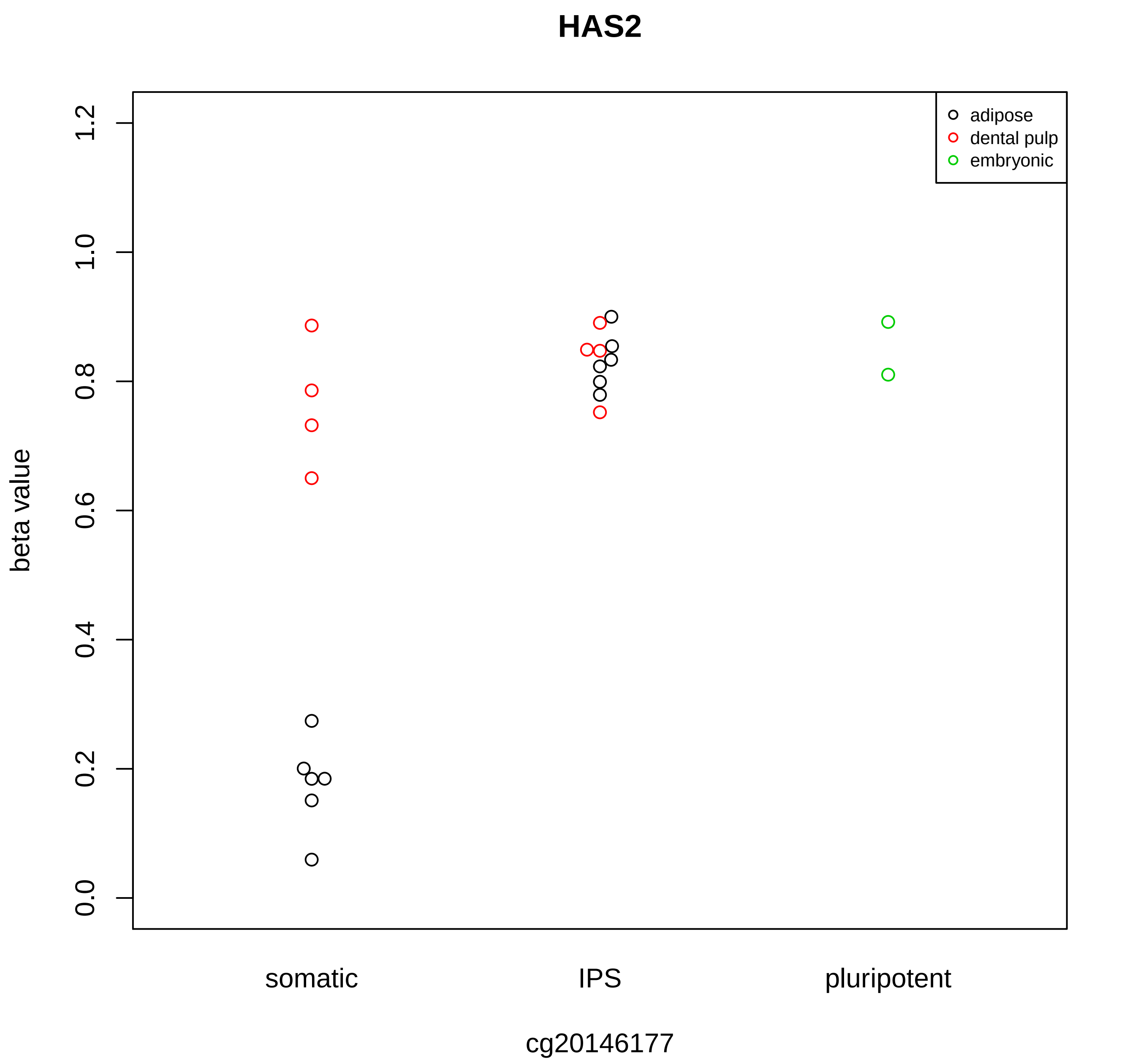
HAS2
adipose
dental pulp
embryonic
beta value
0.0
0.2
0.4
0.6
0.8
1.0
1.2
somatic
IPS
pluripotent
cg20146177

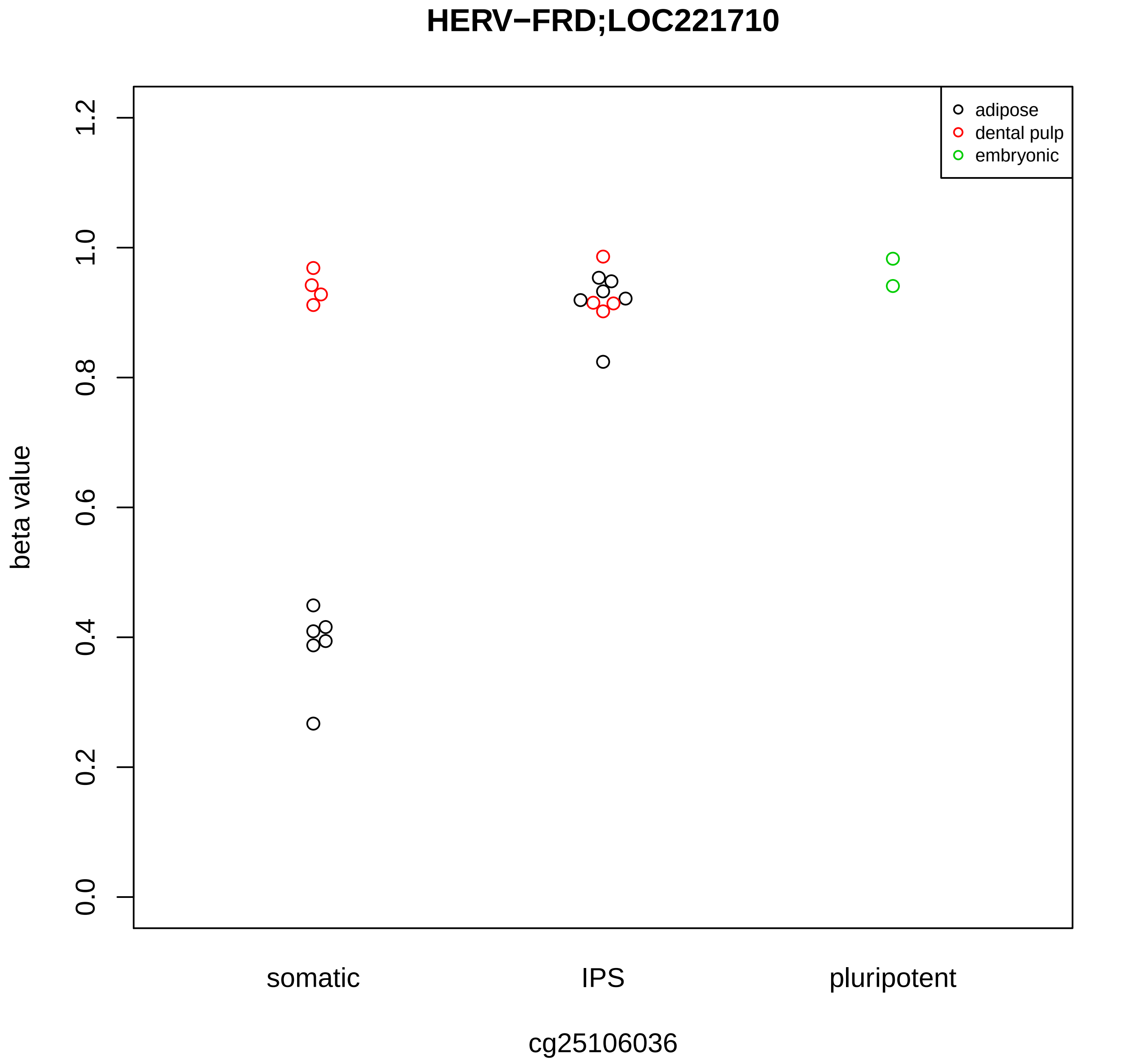

HERV−FRD;LOC221710
adipose
dental pulp
embryonic
beta value
0.0
0.2
0.4
0.6
0.8
1.0
1.2
somatic
IPS
pluripotent
cg25106036

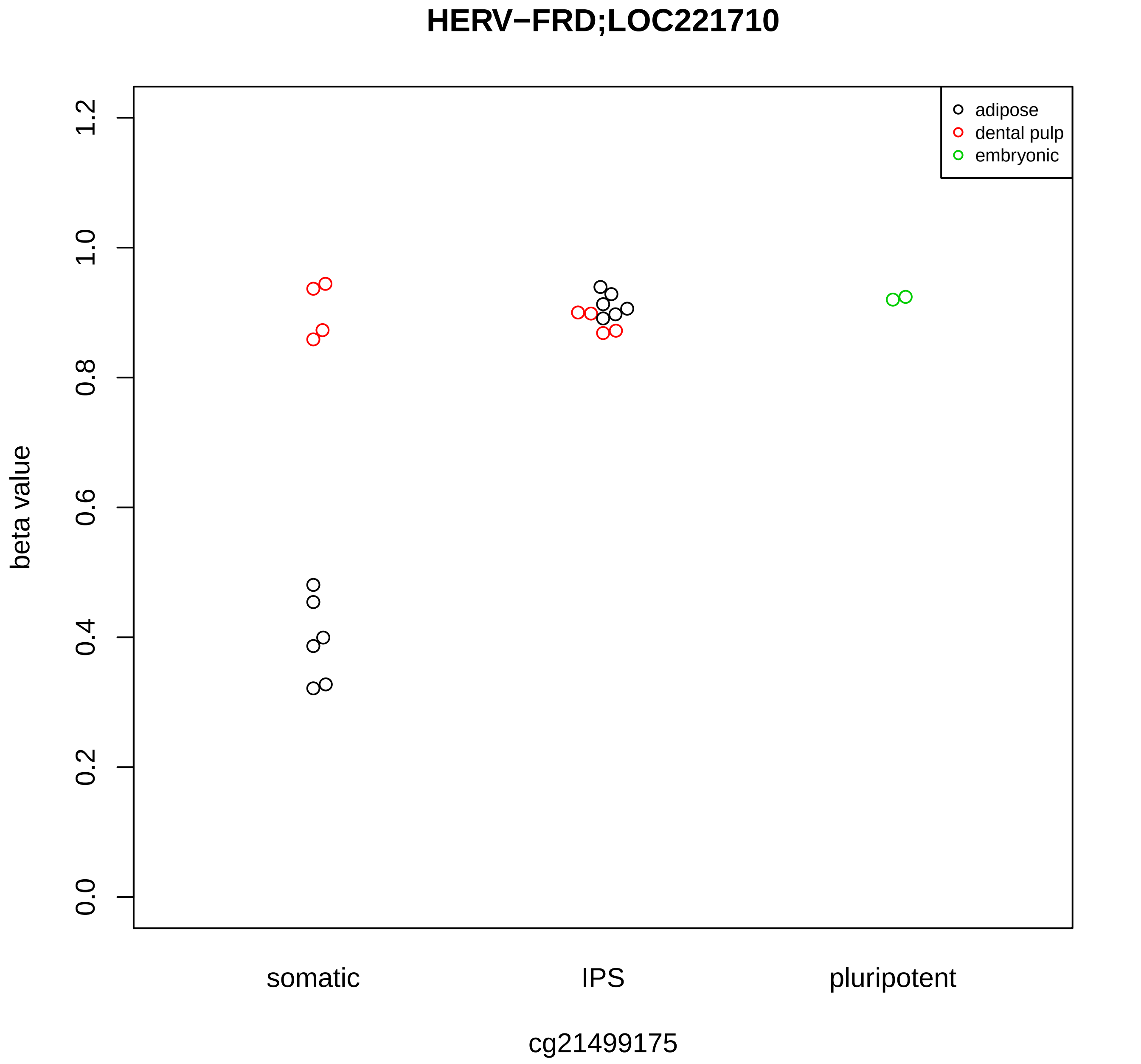
HERV−FRD;LOC221710
adipose
dental pulp
embryonic
1.2
1.0
0.8
0.6
0.4
0.2
0.0
beta value
somatic
IPS
pluripotent
cg21499175

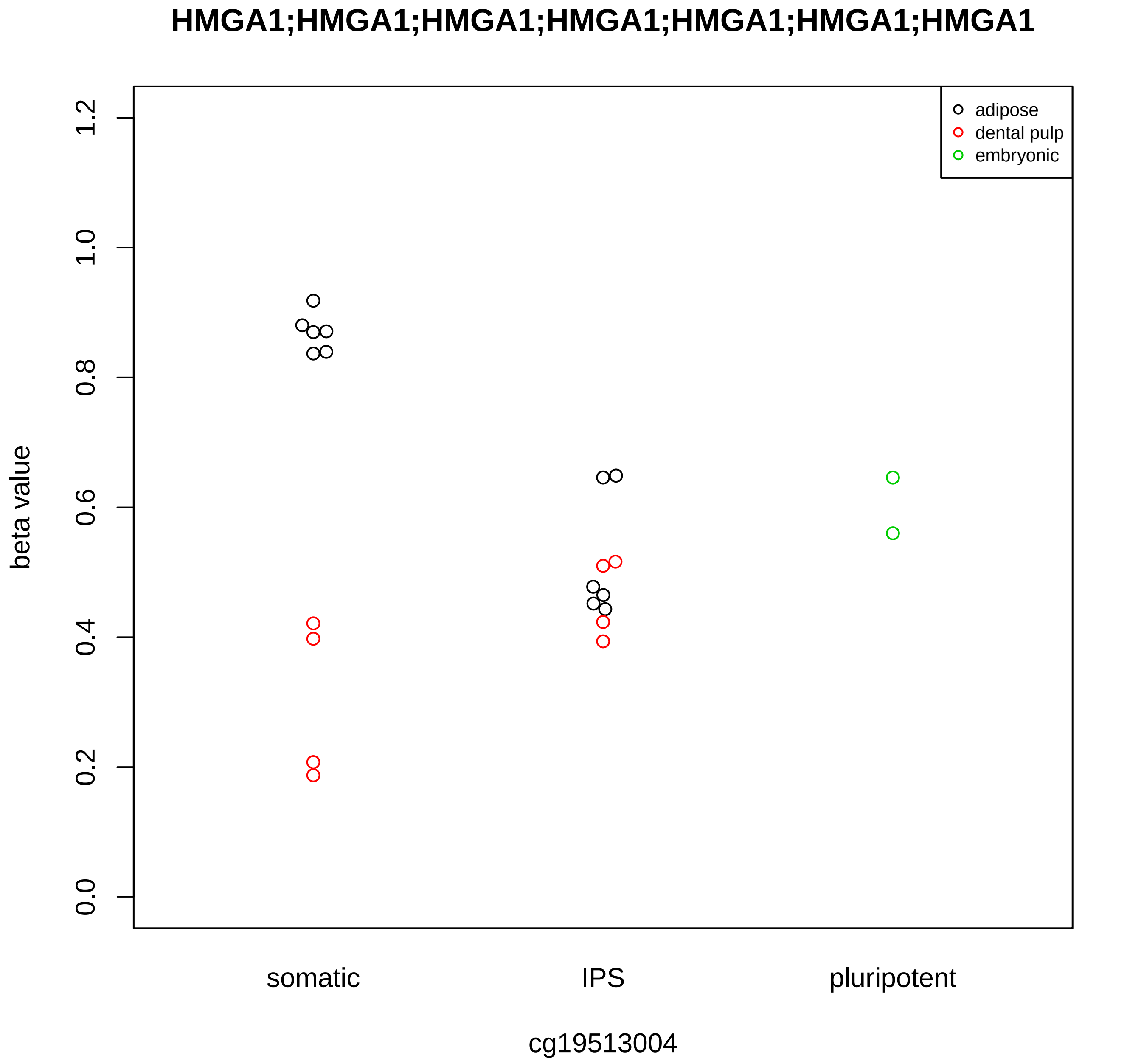
HMGA1;HMGA1;HMGA1;HMGA1;HMGA1;HMGA1;HMGA1
adipose
dental pulp
embryonic
beta value
0.0
0.2
0.4
0.6
0.8
1.0
1.2
somatic
IPS
pluripotent
cg19513004

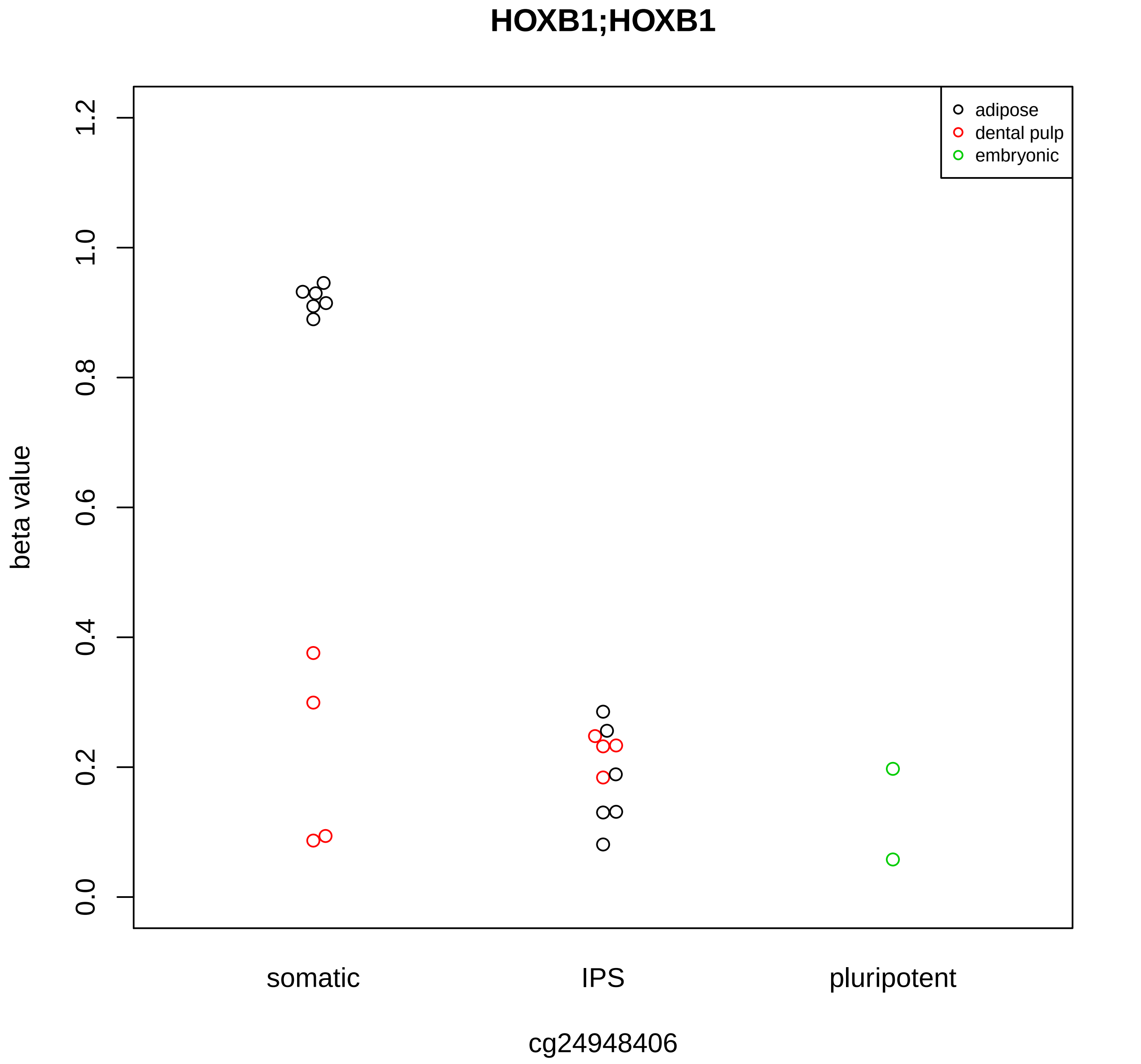
HOXB1;HOXB1
adipose
dental pulp
embryonic
beta value
0.0
0.2
0.4
0.6
0.8
1.0
1.2
somatic
IPS
pluripotent
cg24948406

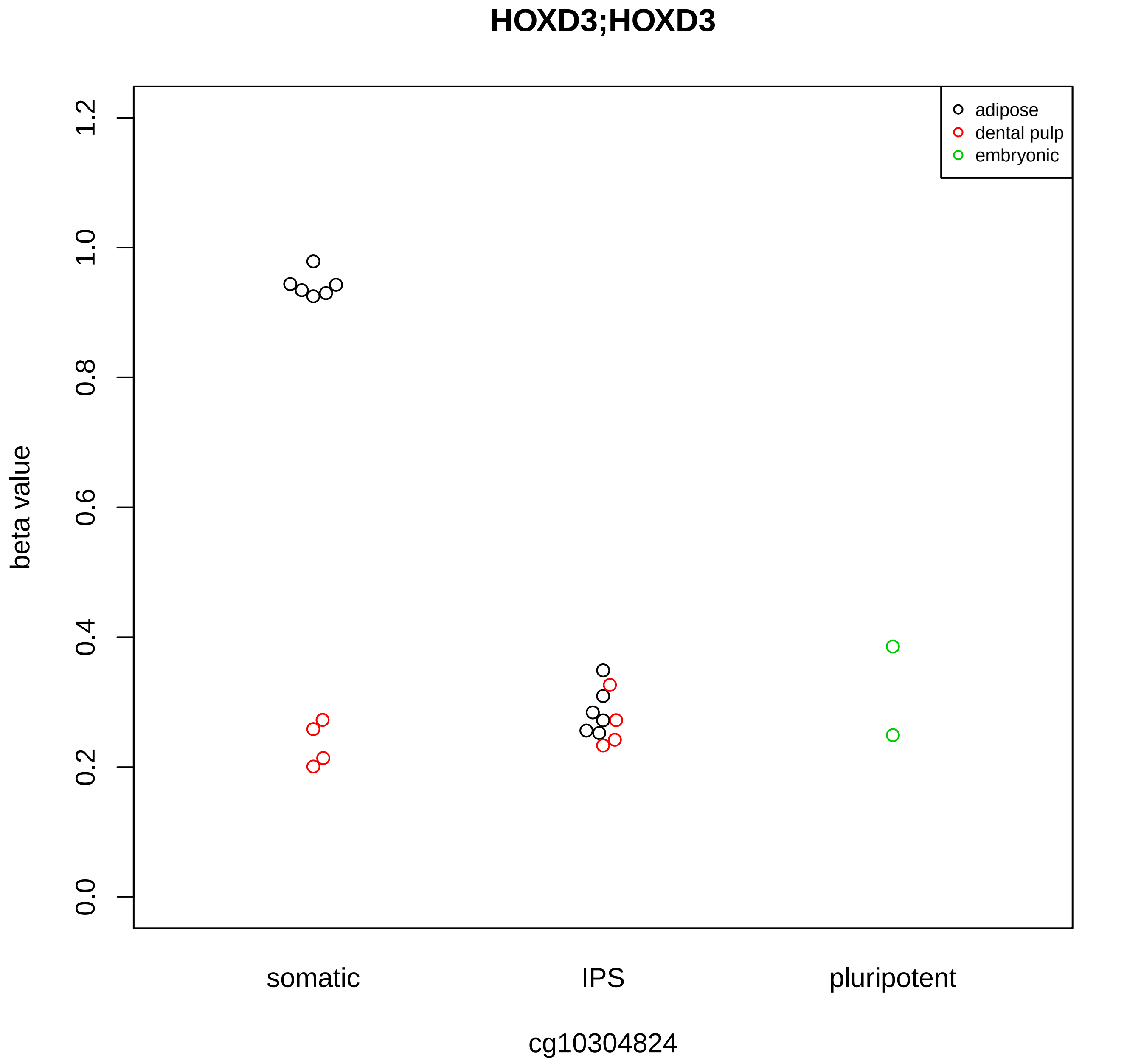

HOXD3;HOXD3
beta value
0.0
0.2
0.4
0.6
0.8
1.0
1.2
adipose
dental pulp
embryonic
somatic
IPS
pluripotent
cg10304824

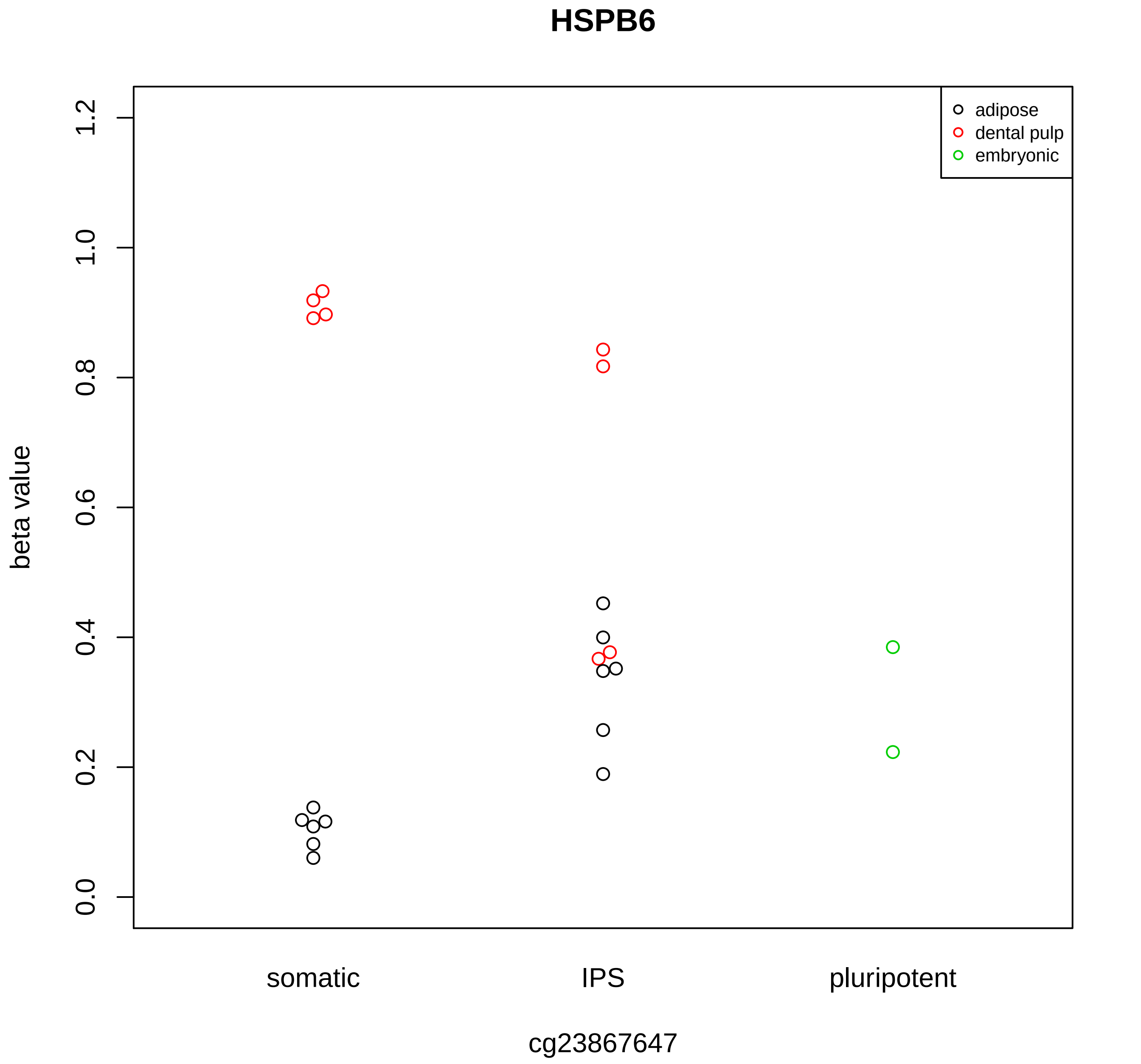

HSPB6
adipose
dental pulp
embryonic
beta value
0.0
0.2
0.4
0.6
0.8
1.0
1.2
somatic
IPS
pluripotent
cg23867647

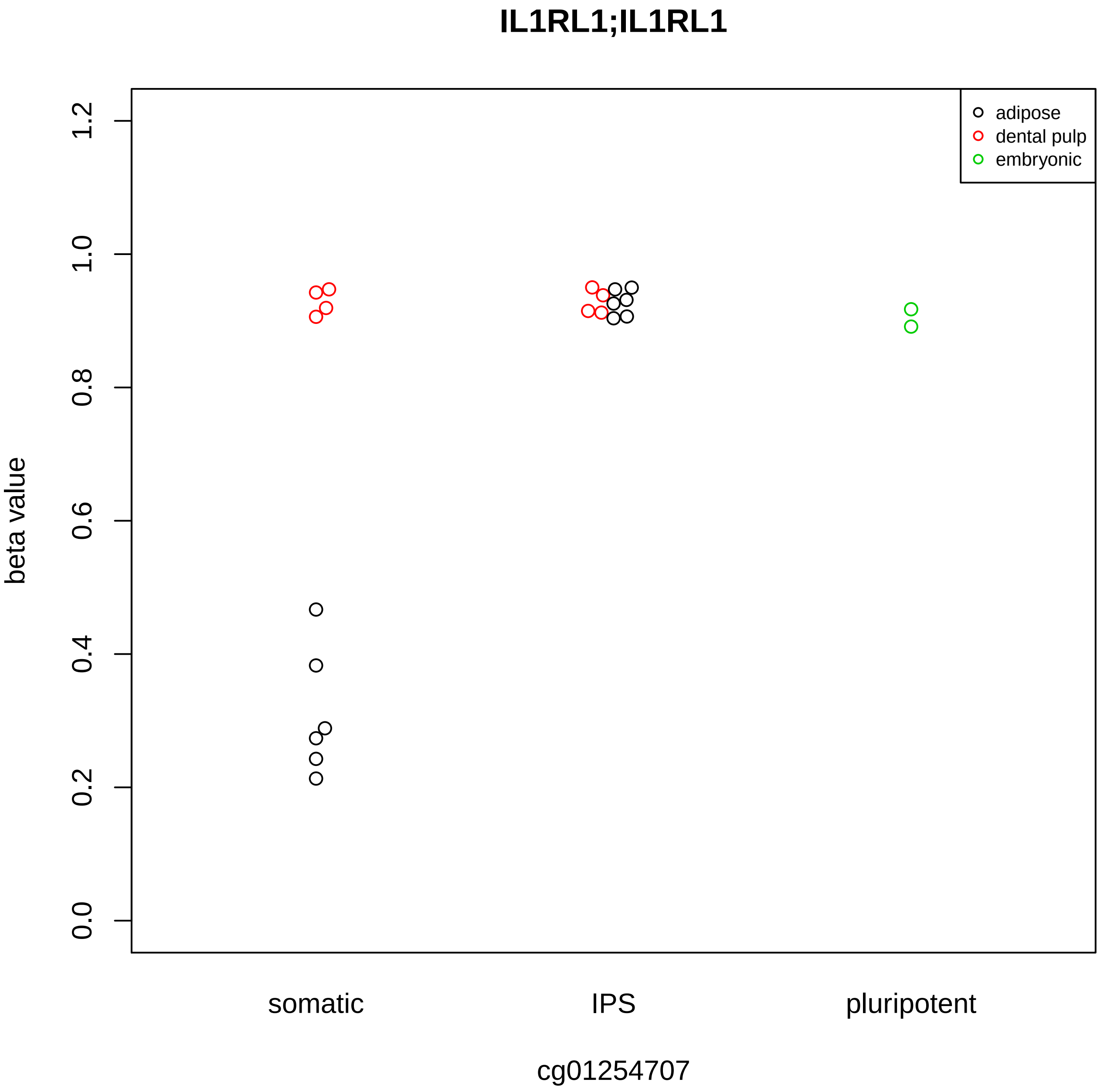

IL1RL1;IL1RL1
beta value
0.0
0.2
0.4
0.6
0.8
1.0
1.2
adipose
dental pulp
embryonic
somatic
IPS
pluripotent
cg01254707

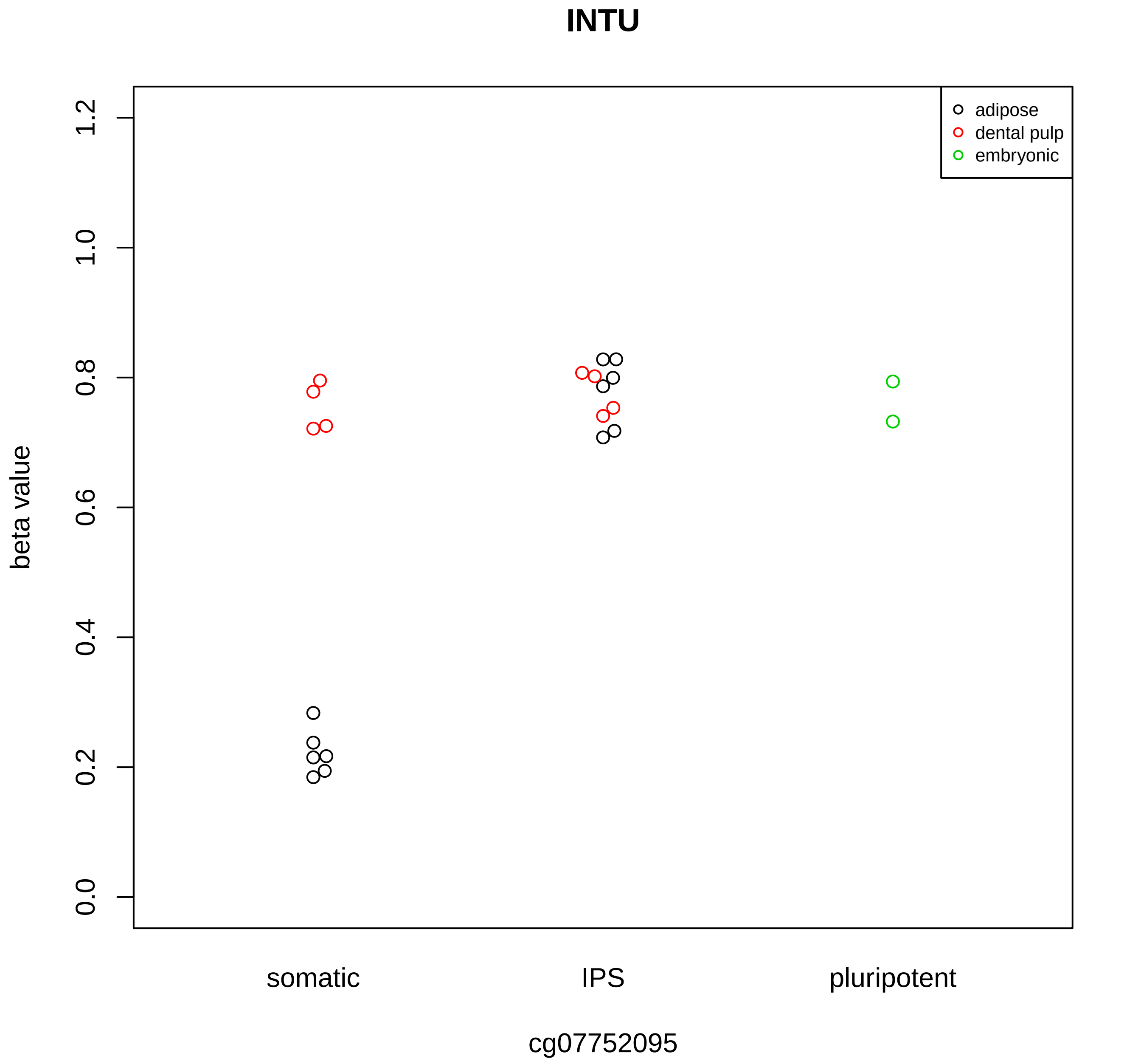

INTU
beta value
0.0
0.2
0.4
0.6
0.8
1.0
1.2
adipose
dental pulp
embryonic
somatic
IPS
pluripotent
cg07752095

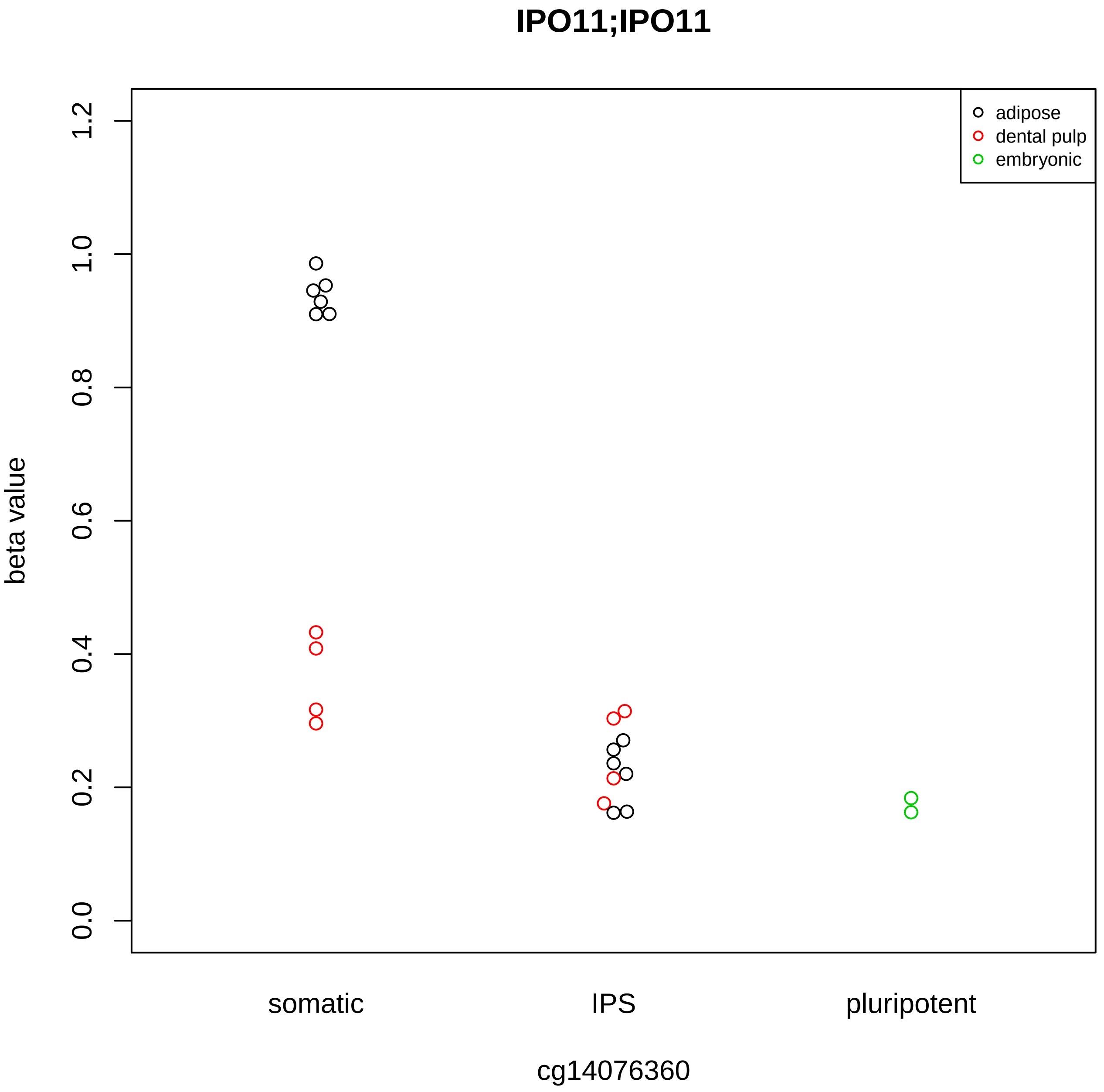
IPO11;IPO11
adipose
dental pulp
embryonic
beta value
0.0
0.2
0.4
0.6
0.8
1.0
1.2
somatic
IPS
pluripotent
cg14076360

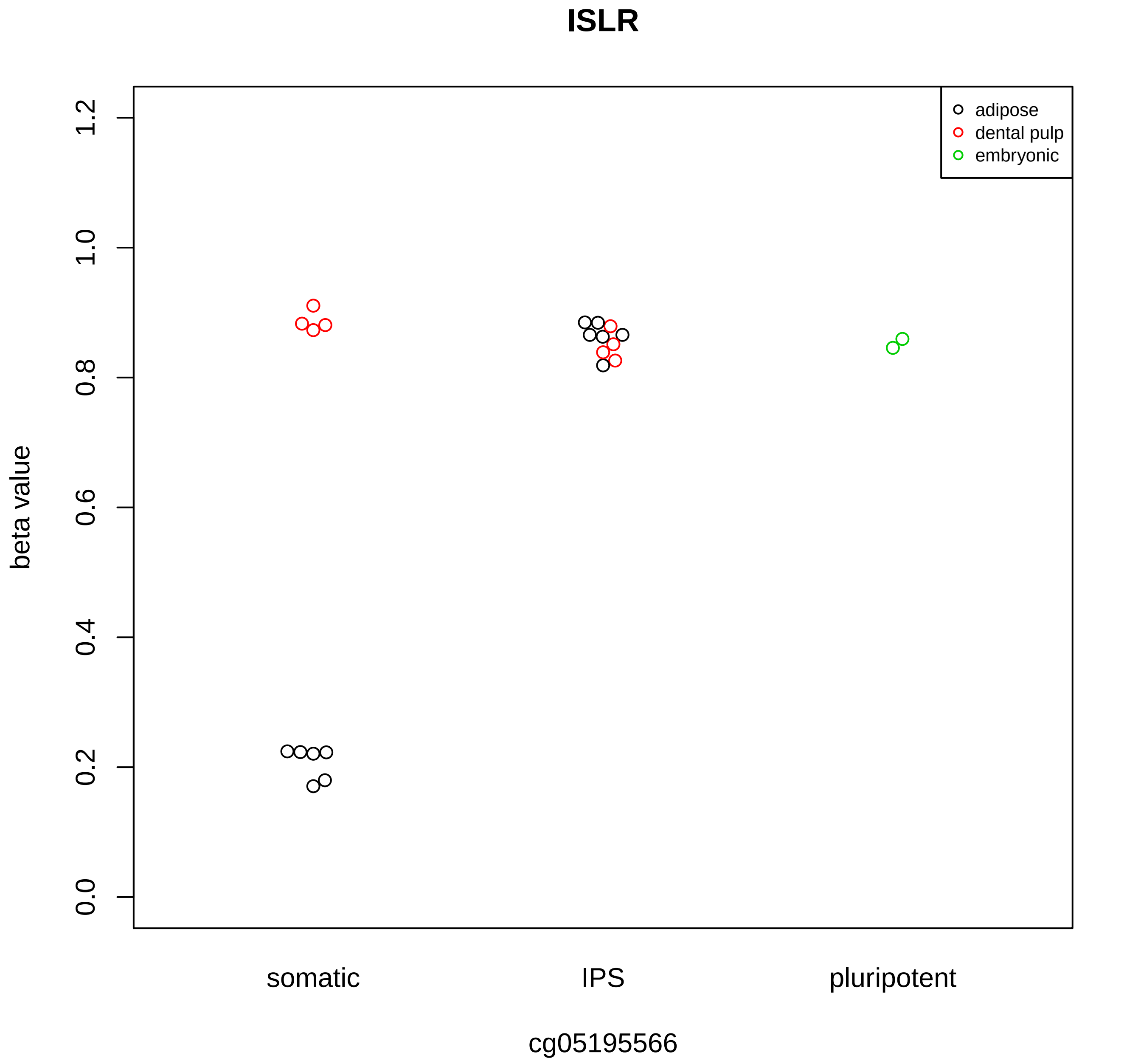
ISLR
adipose
dental pulp
embryonic
beta value
1.2
1.0
0.8
0.6
0.4
0.2
0.0
somatic
IPS
pluripotent
cg05195566

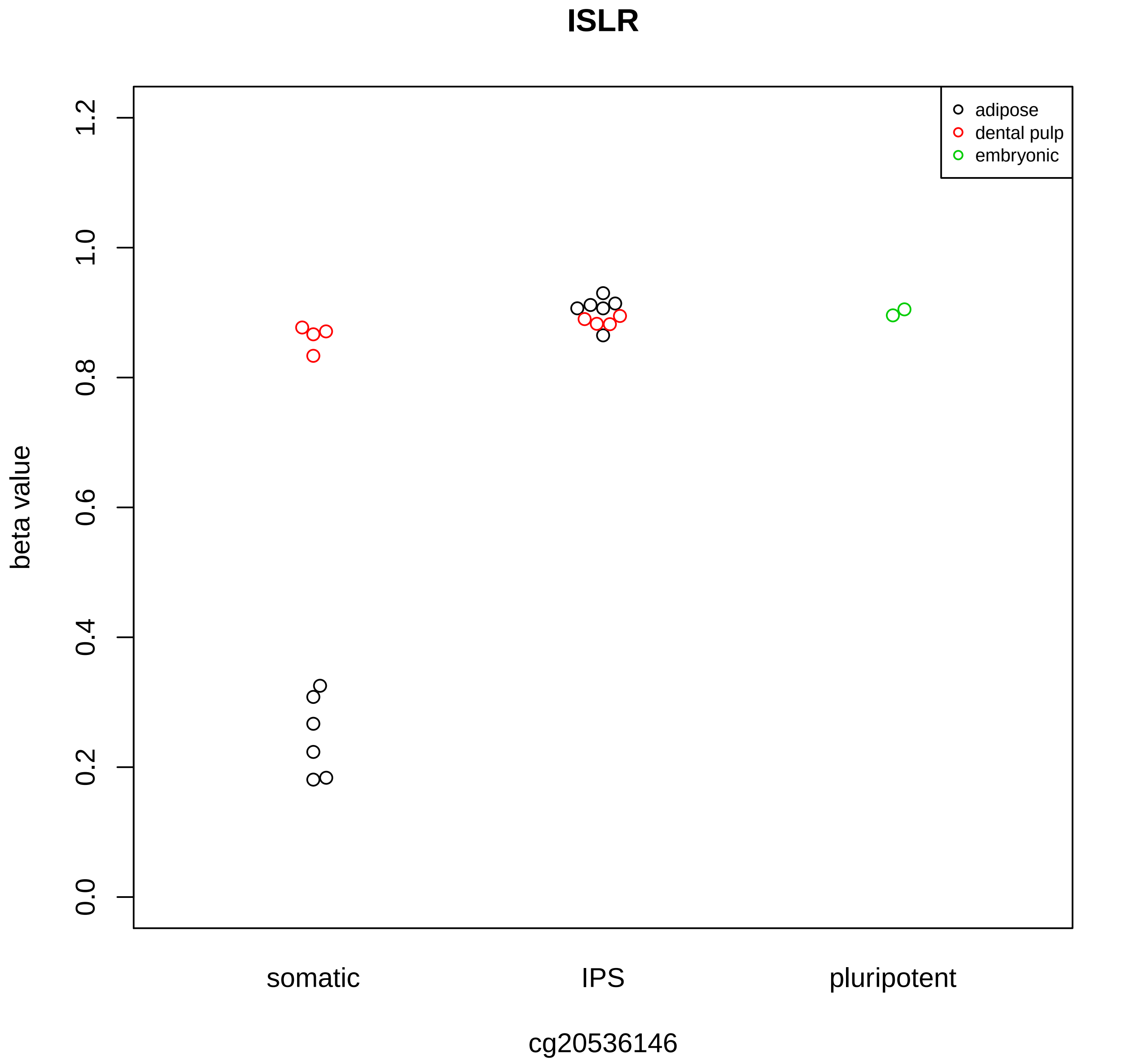

ISLR
adipose
dental pulp
embryonic
beta value
0.0
0.2
0.4
0.6
0.8
1.0
1.2
somatic
IPS
pluripotent
cg20536146

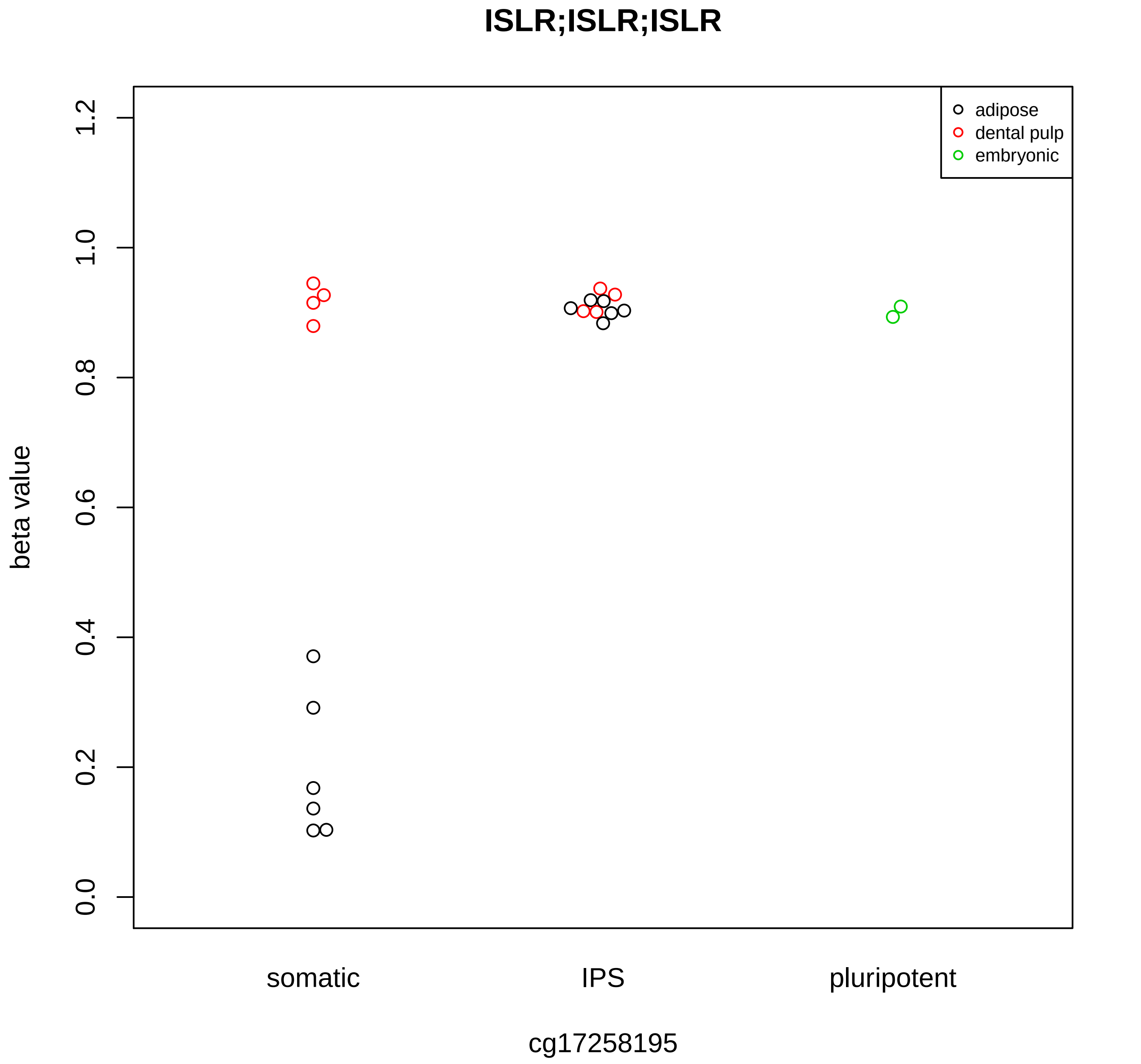
ISLR;ISLR;ISLR
beta value
0.0
0.2
0.4
0.6
0.8
1.0
1.2
adipose
dental pulp
embryonic
somatic
IPS
pluripotent
cg17258195

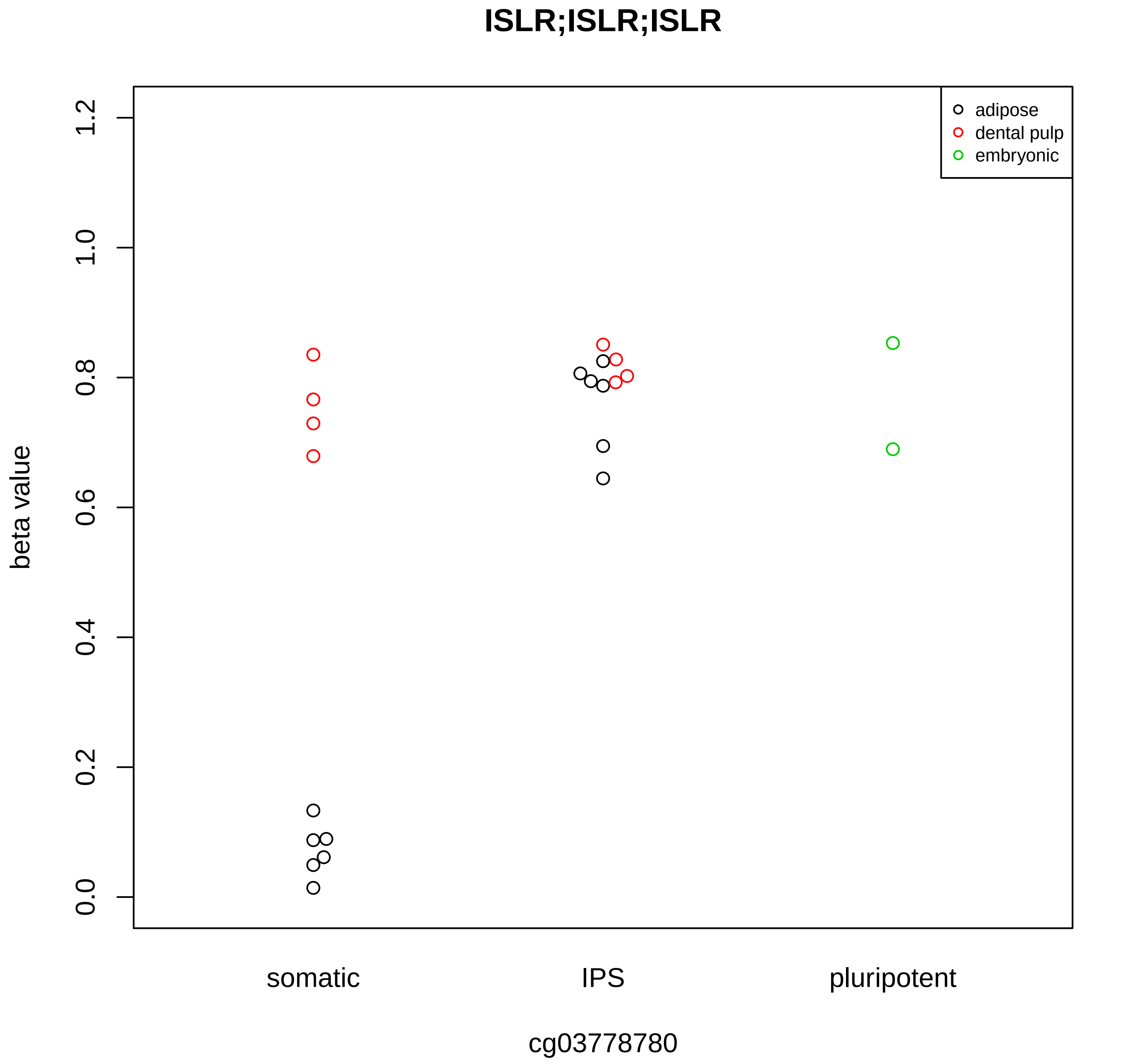
ISLR;ISLR;ISLR
beta value
0.0
0.2
0.4
0.6
0.8
1.0
1.2
adipose
dental pulp
embryonic
somatic
IPS
pluripotent
cg03778780

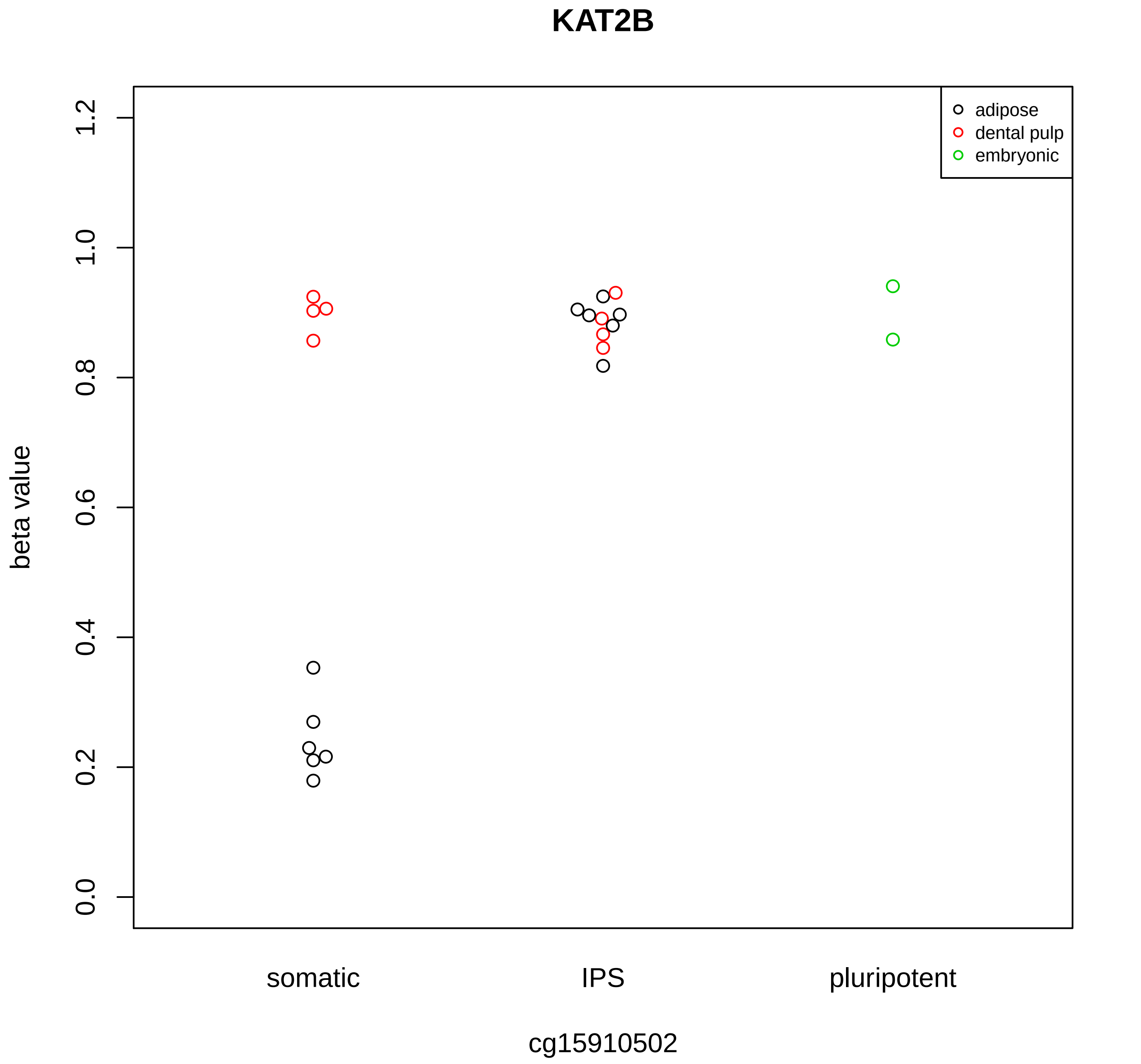
KAT2B
beta value
1.2
1.0
0.8
0.6
0.4
0.2
0.0
adipose
dental pulp
embryonic
somatic
IPS
pluripotent
cg15910502

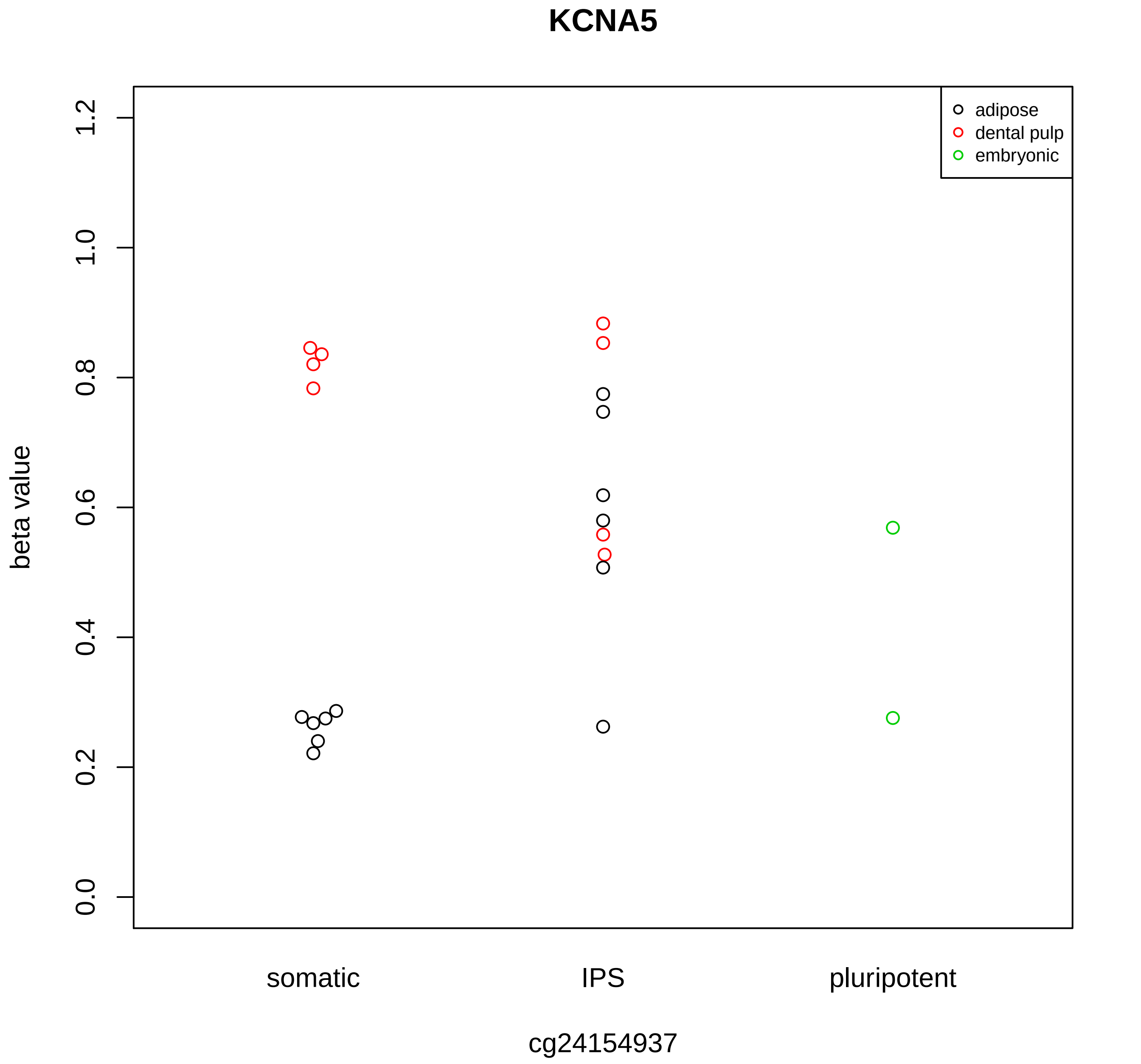
KCNA5
adipose
dental pulp
embryonic
beta value
0.0
0.2
0.4
0.6
0.8
1.0
1.2
somatic
IPS
pluripotent
cg24154937

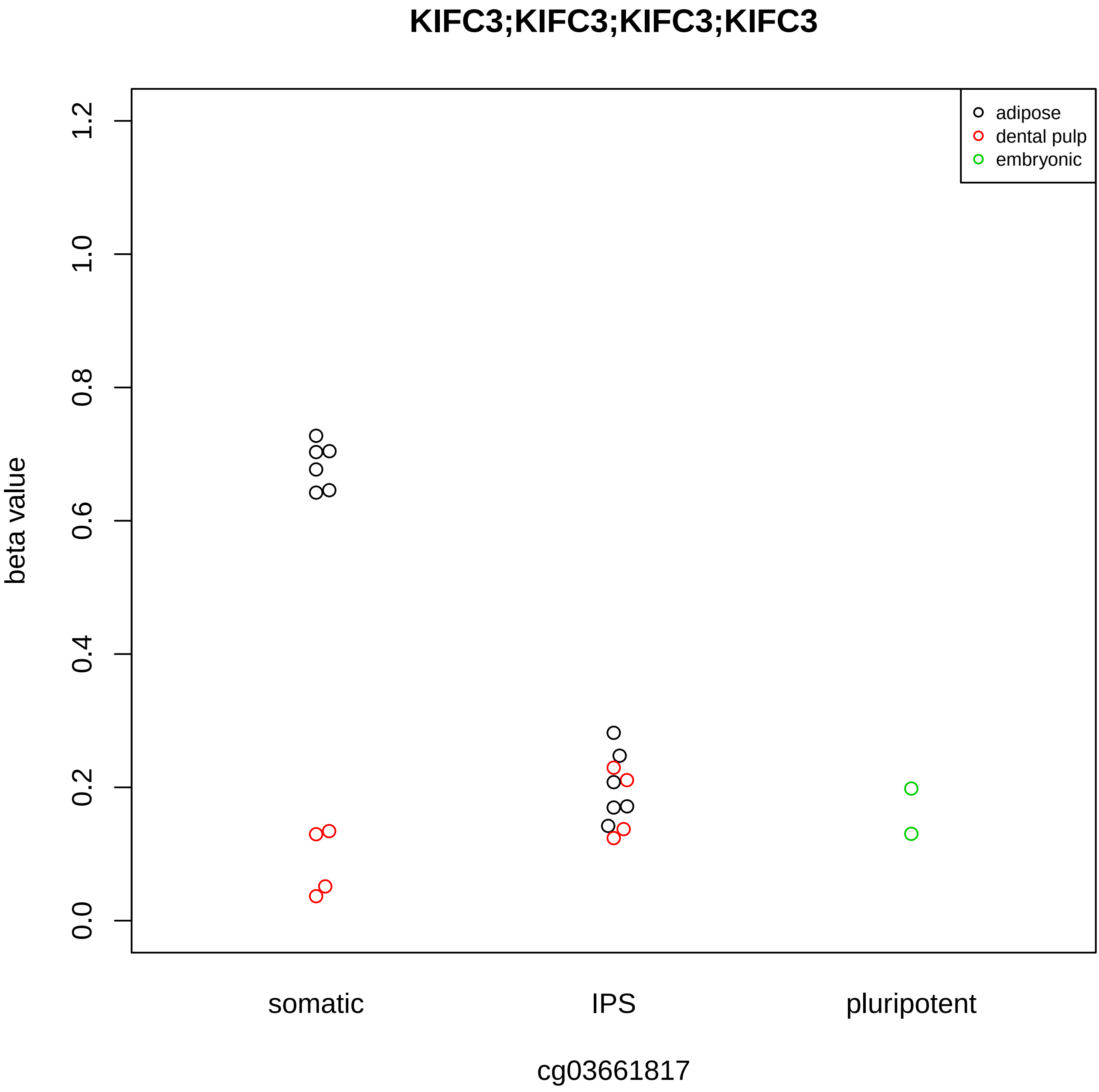
KIFC3;KIFC3;KIFC3;KIFC3
adipose
dental pulp
embryonic
beta value
0.0
0.2
0.4
0.6
0.8
1.0
1.2
somatic
IPS
pluripotent
cg03661817

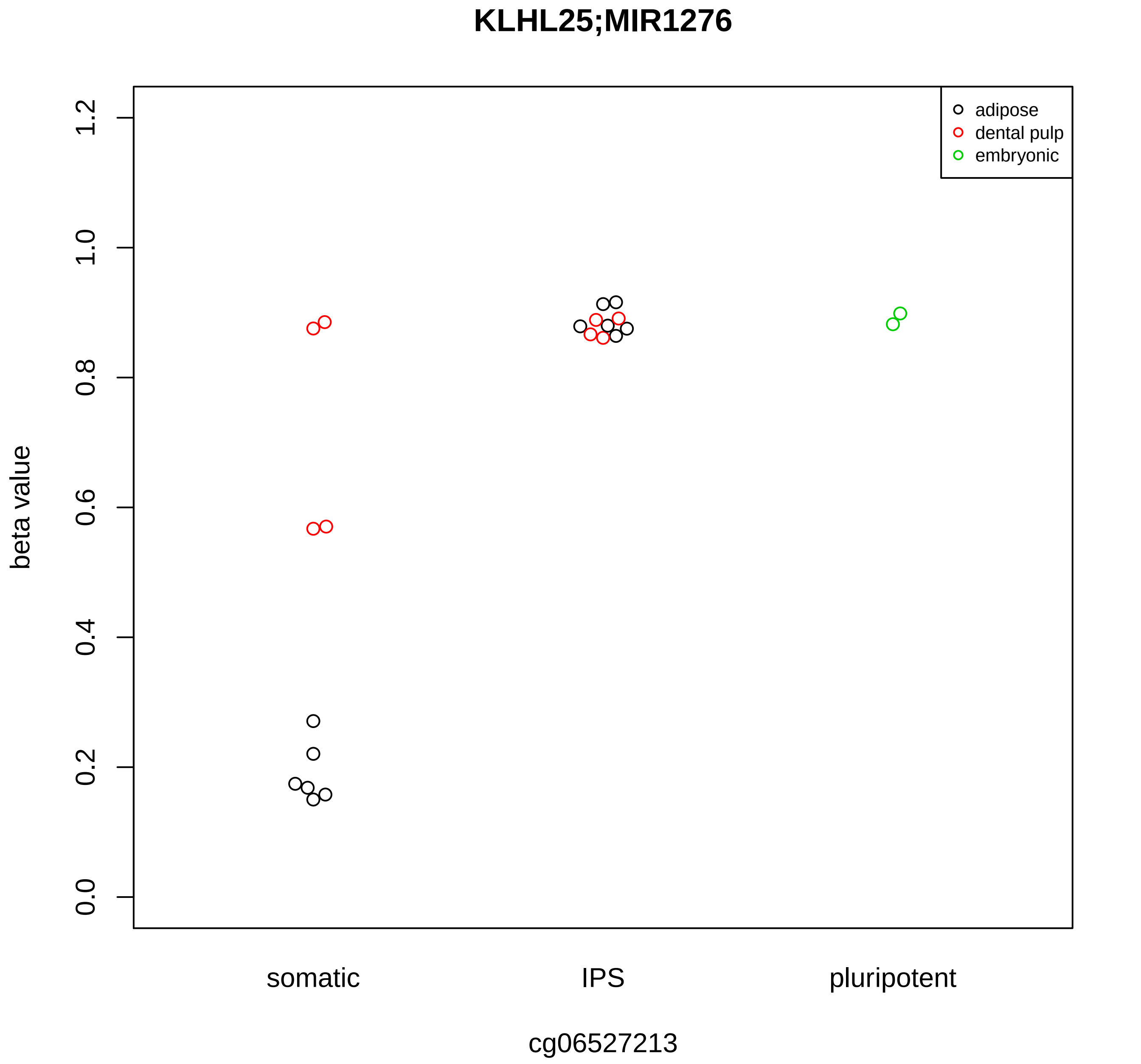

KLHL25;MIR1276
beta value
0.0
0.2
0.4
0.6
0.8
1.0
1.2
adipose
dental pulp
embryonic
somatic
IPS
pluripotent
cg06527213

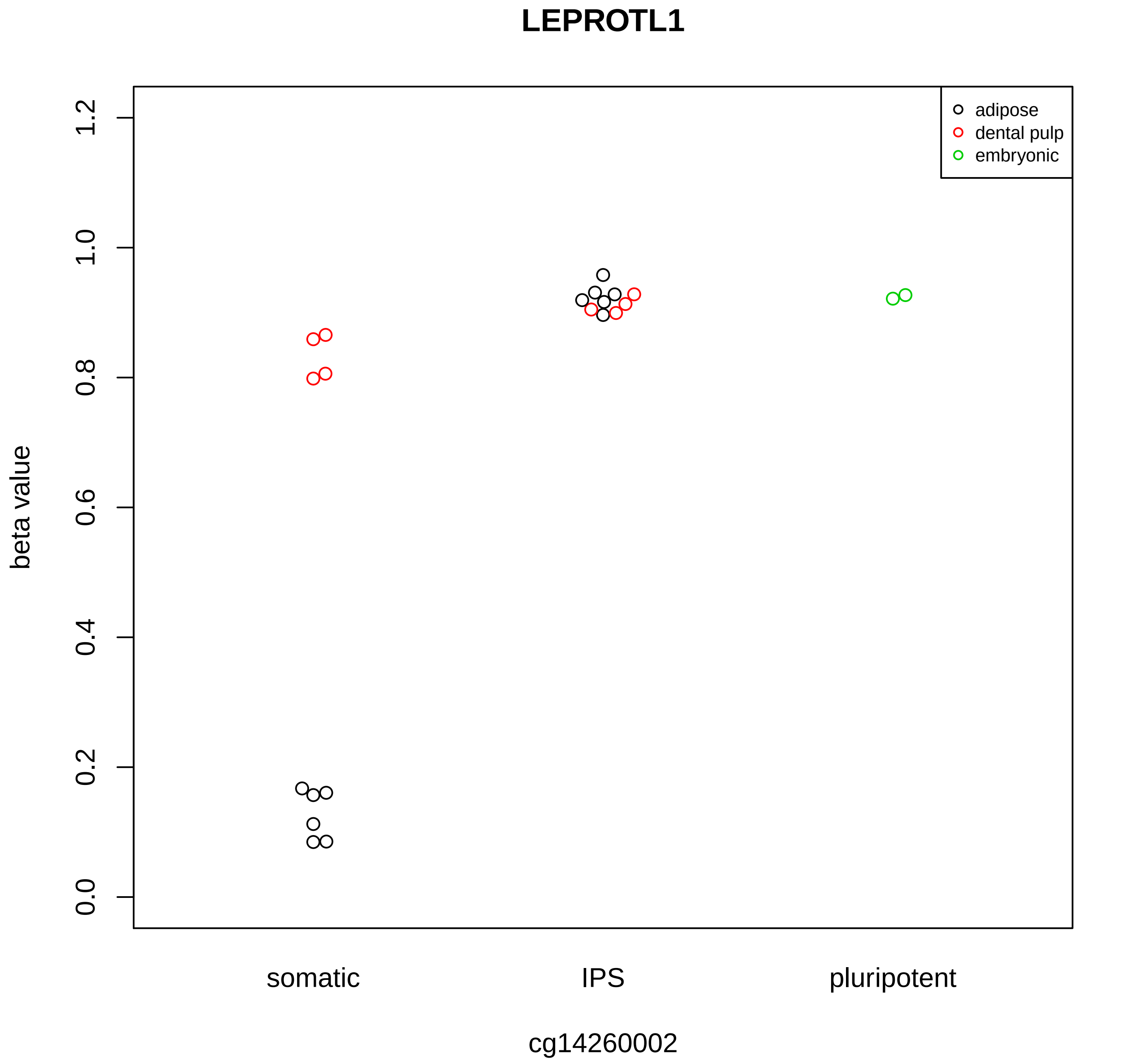

LEPROTL1
adipose
dental pulp
embryonic
0.0
0.2
0.4
0.6
0.8
1.0
1.2
beta value
somatic
IPS
pluripotent
cg14260002

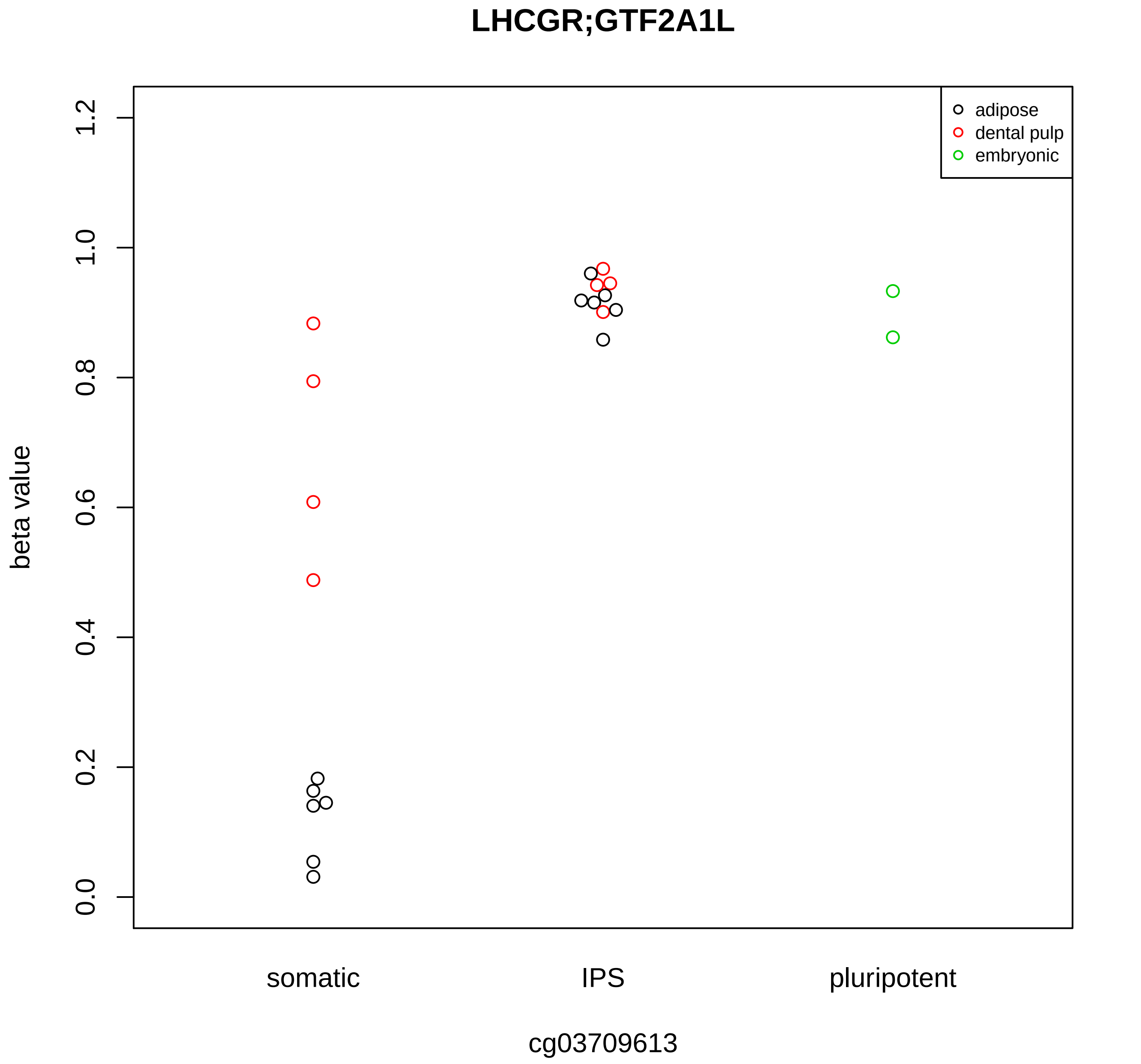
LHCGR;GTF2A1L
adipose
dental pulp
embryonic
beta value
0.0
0.2
0.4
0.6
0.8
1.0
1.2
somatic
IPS
pluripotent
cg03709613

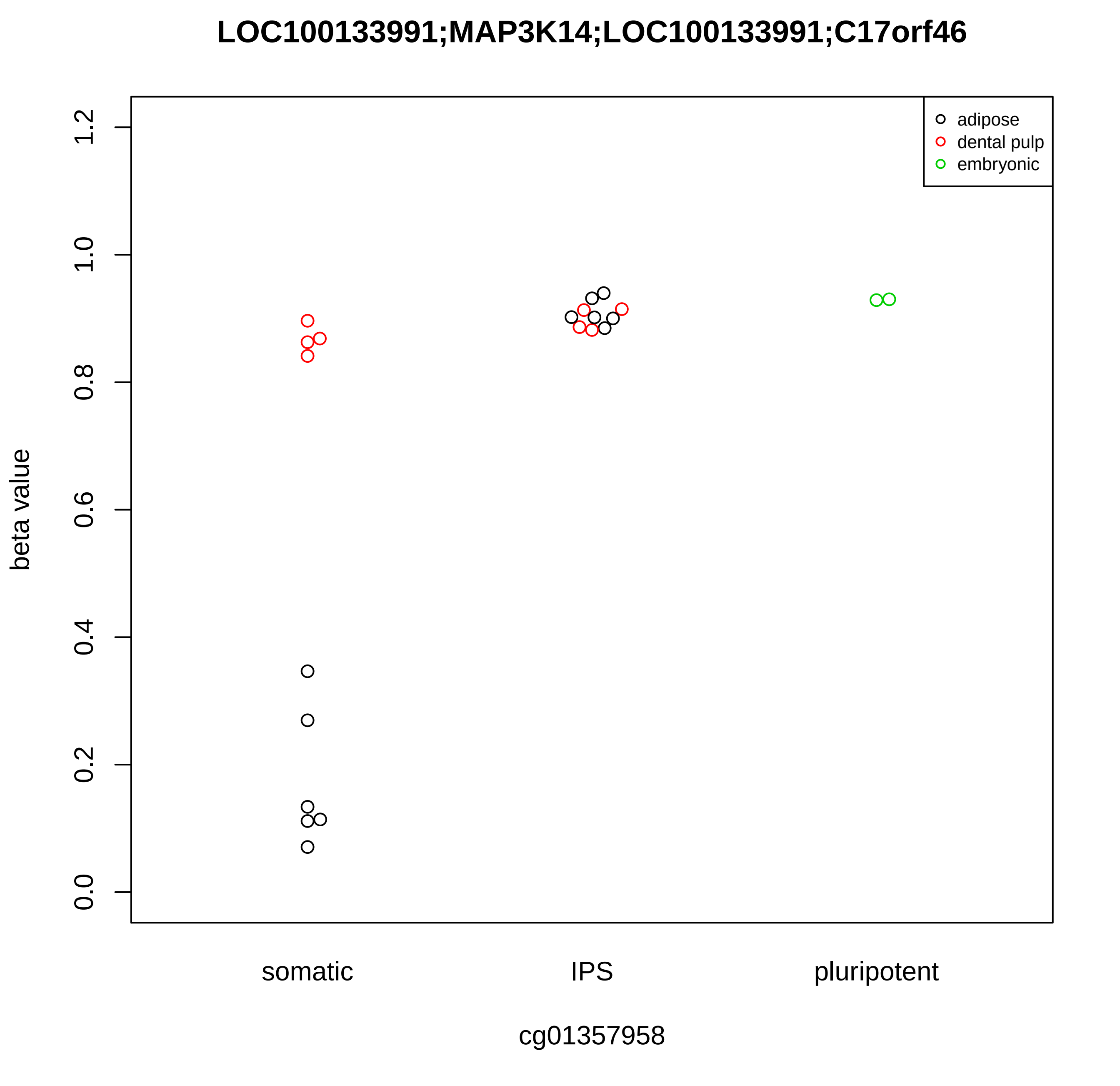

LOC100133991;MAP3K14;LOC100133991;C17orf46
beta value
0.0
0.2
0.4
0.6
0.8
1.0
1.2
adipose
dental pulp
embryonic
somatic
IPS
pluripotent
cg01357958

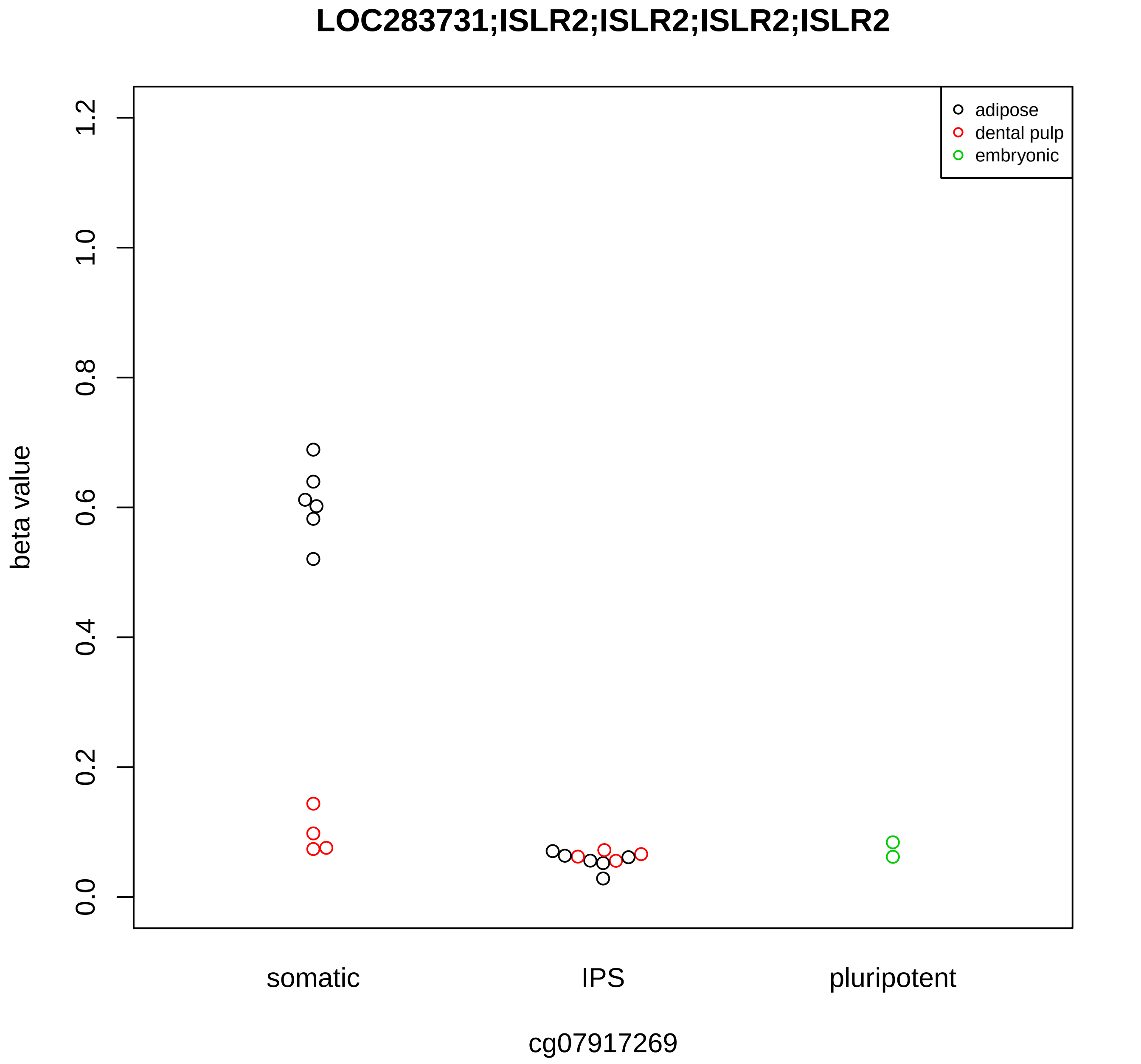
LOC283731;ISLR2;ISLR2;ISLR2;ISLR2
beta value
0.0
0.2
0.4
0.6
0.8
1.0
1.2
adipose
dental pulp
embryonic
somatic
IPS
pluripotent
cg07917269

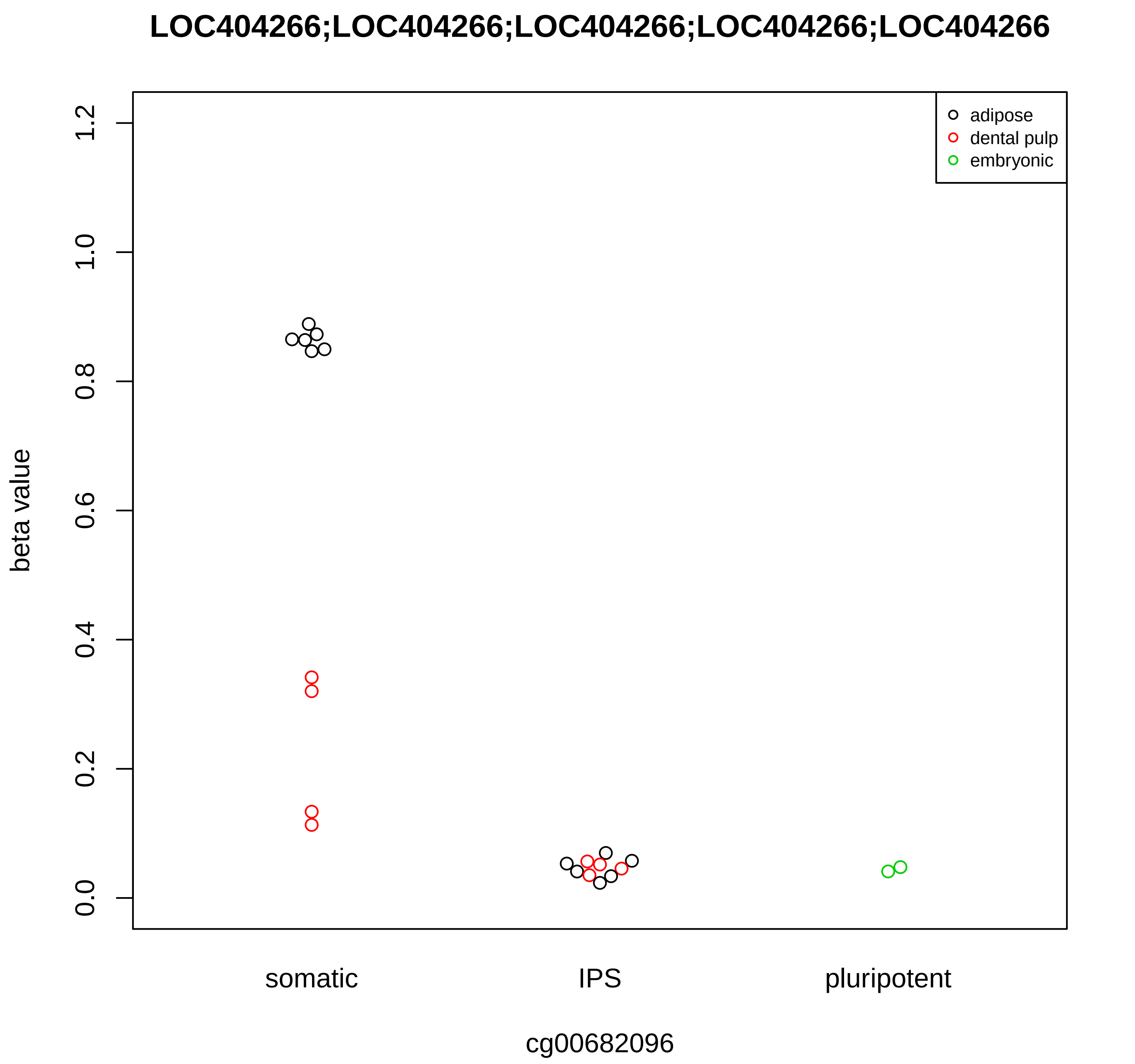

LOC404266;LOC404266;LOC404266;LOC404266;LOC404266
adipose
dental pulp
embryonic
beta value
1.2
1.0
0.8
0.6
0.4
0.2
0.0
somatic
IPS
pluripotent
cg00682096

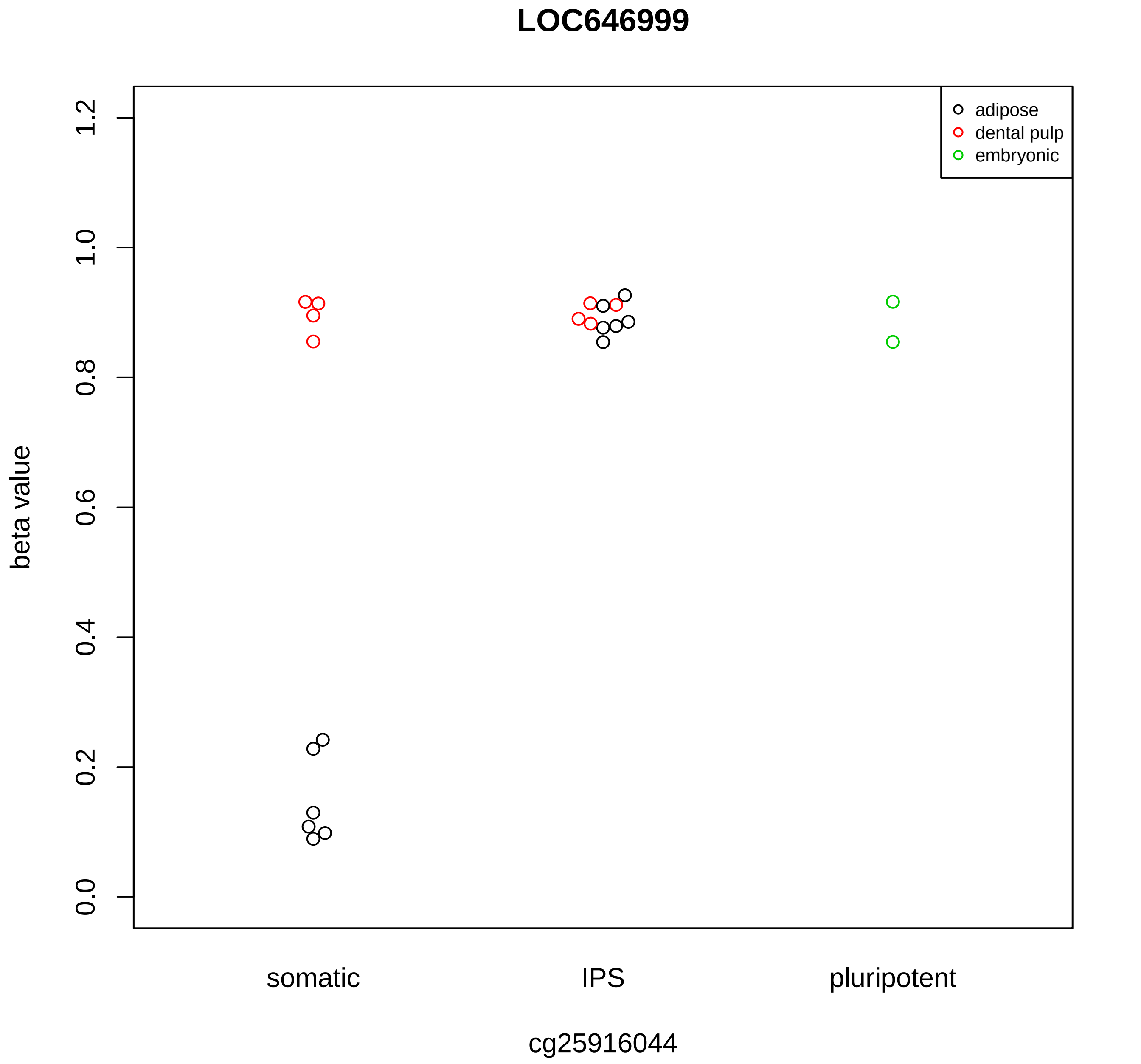

LOC646999
beta value
0.0
0.2
0.4
0.6
0.8
1.0
1.2
adipose
dental pulp
embryonic
somatic
IPS
pluripotent
cg25916044

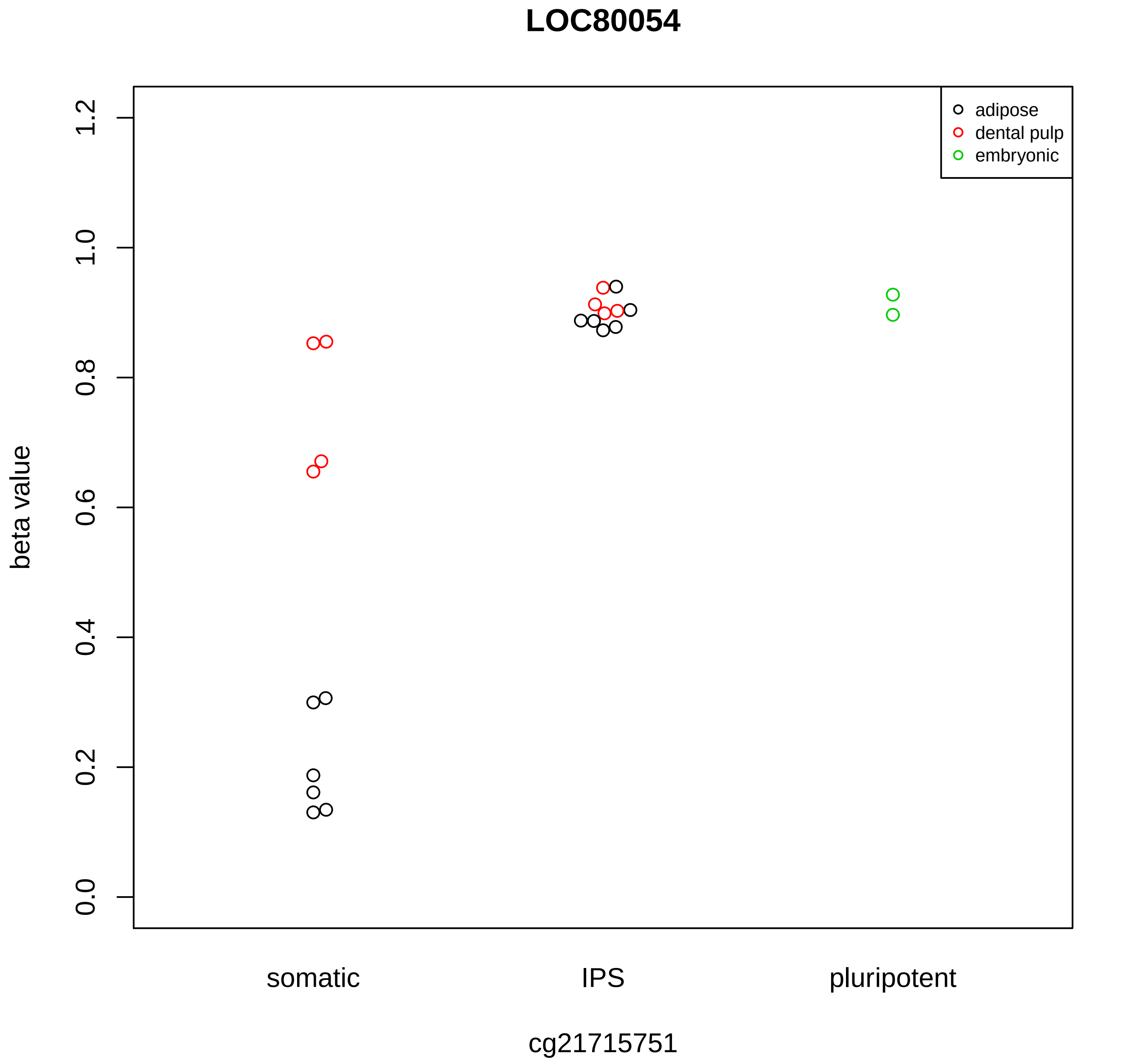
LOC80054
adipose
dental pulp
embryonic
beta value
0.0
0.2
0.4
0.6
0.8
1.0
1.2
somatic
IPS
pluripotent
cg21715751

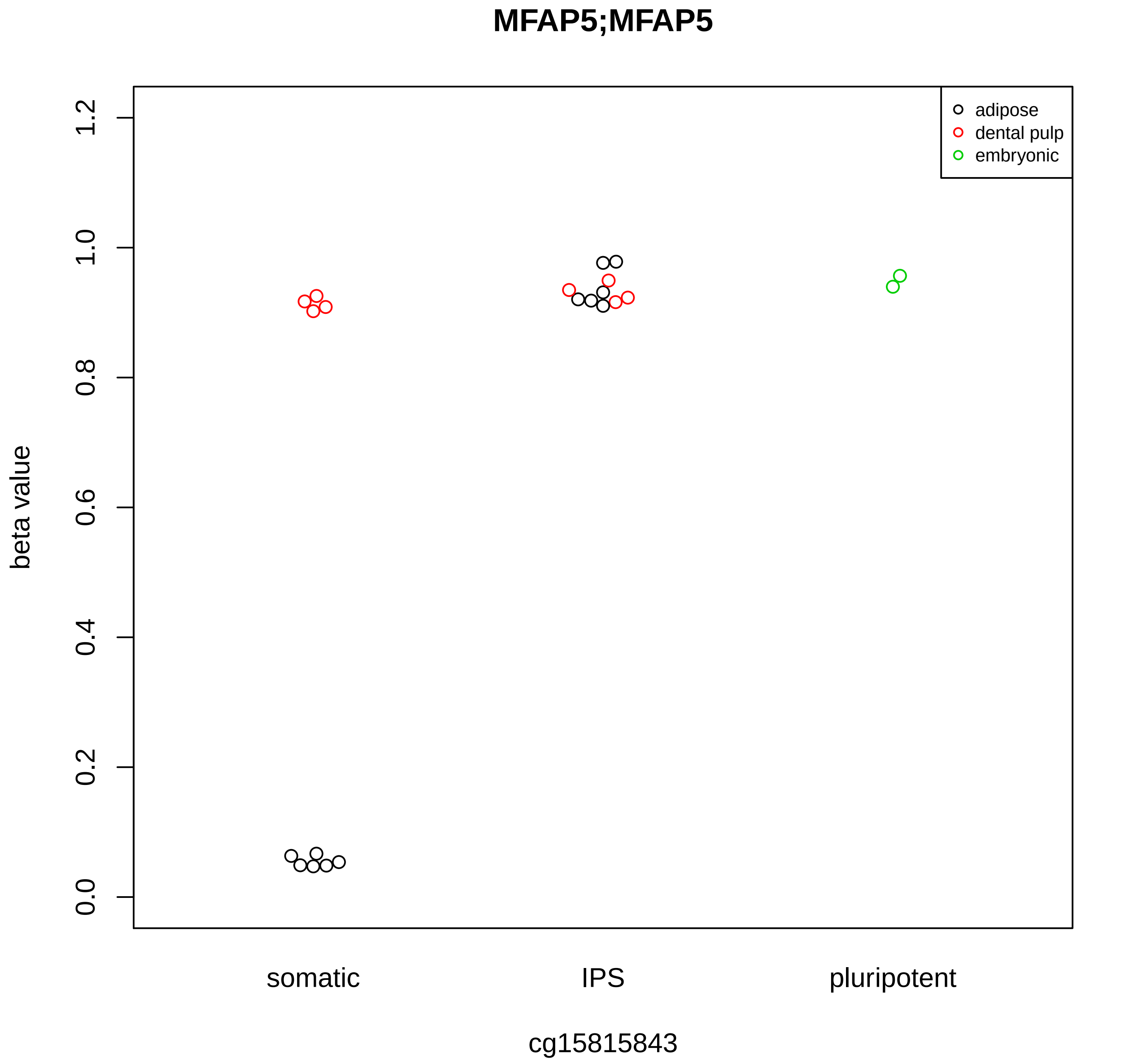
MFAP5;MFAP5
beta value
0.0
0.2
0.4
0.6
0.8
1.0
1.2
adipose
dental pulp
embryonic
somatic
IPS
pluripotent
cg15815843

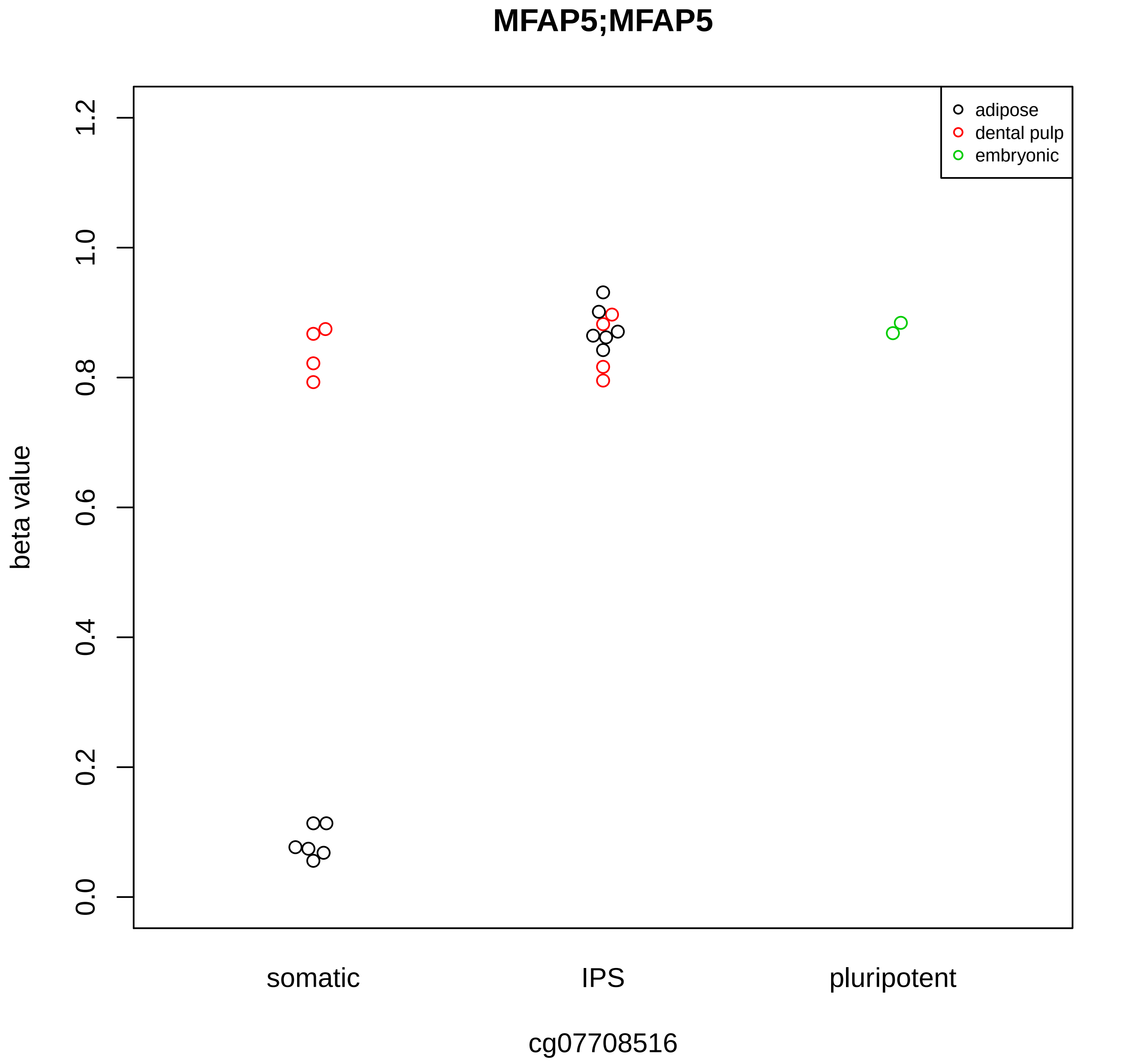
MFAP5;MFAP5
adipose
dental pulp
embryonic
beta value
0.0
0.2
0.4
0.6
0.8
1.0
1.2
somatic
IPS
pluripotent
cg07708516

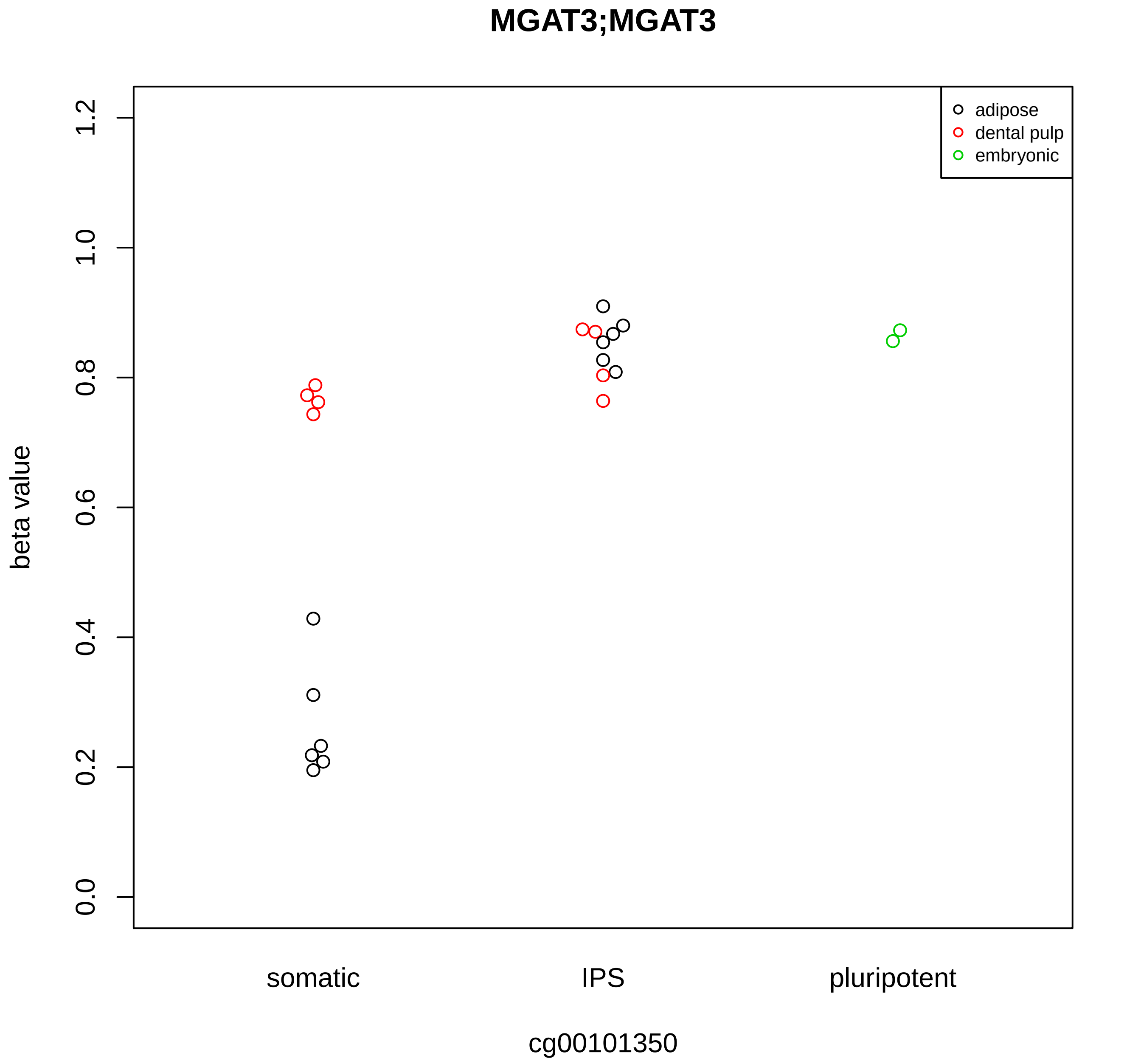

MGAT3;MGAT3
adipose
dental pulp
embryonic
beta value
0.0
0.2
0.4
0.6
0.8
1.0
1.2
somatic
IPS
pluripotent
cg00101350

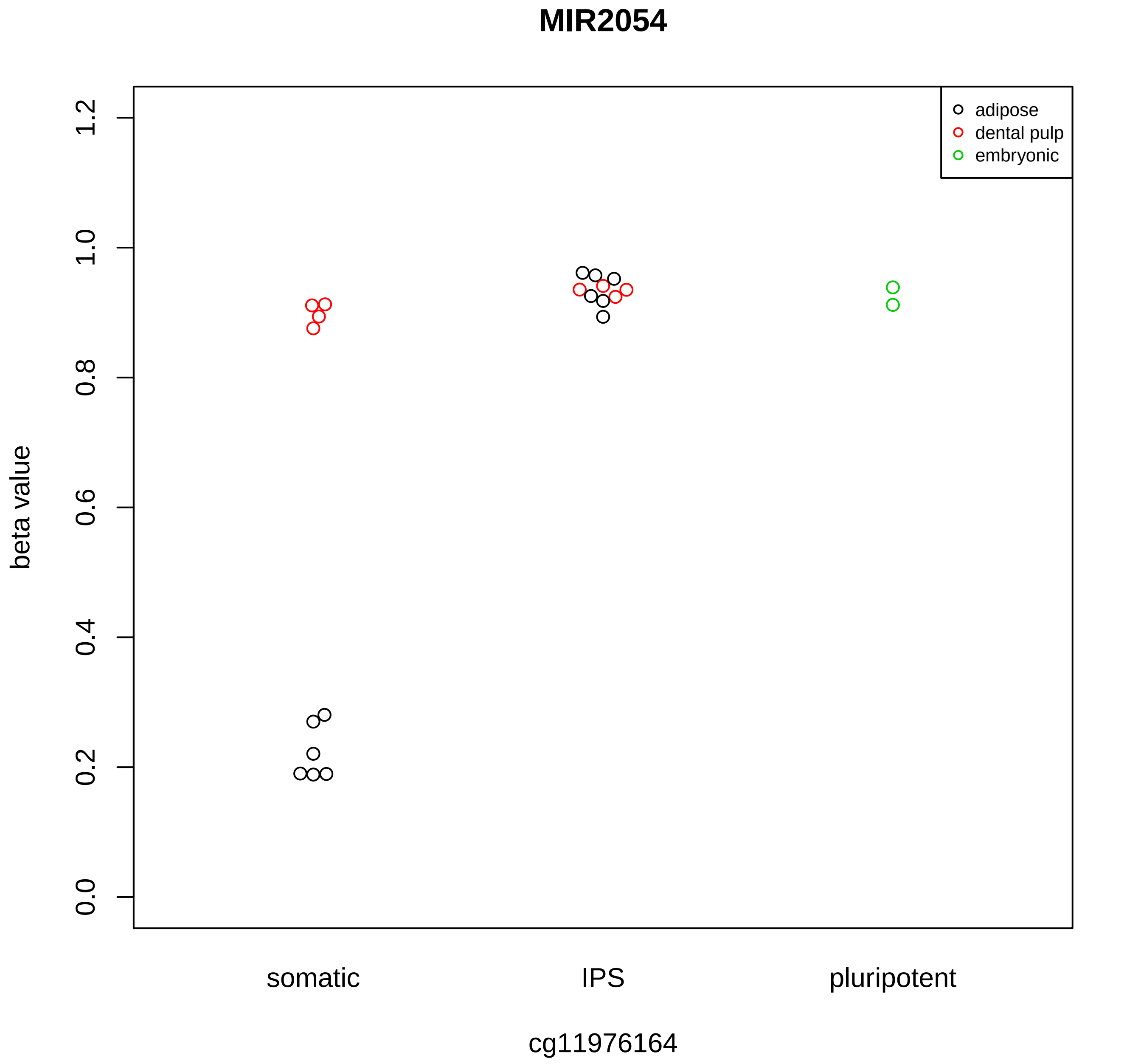
MIR2054
beta value
0.0
0.2
0.4
0.6
0.8
1.0
1.2
adipose
dental pulp
embryonic
somatic
IPS
pluripotent
cg11976164

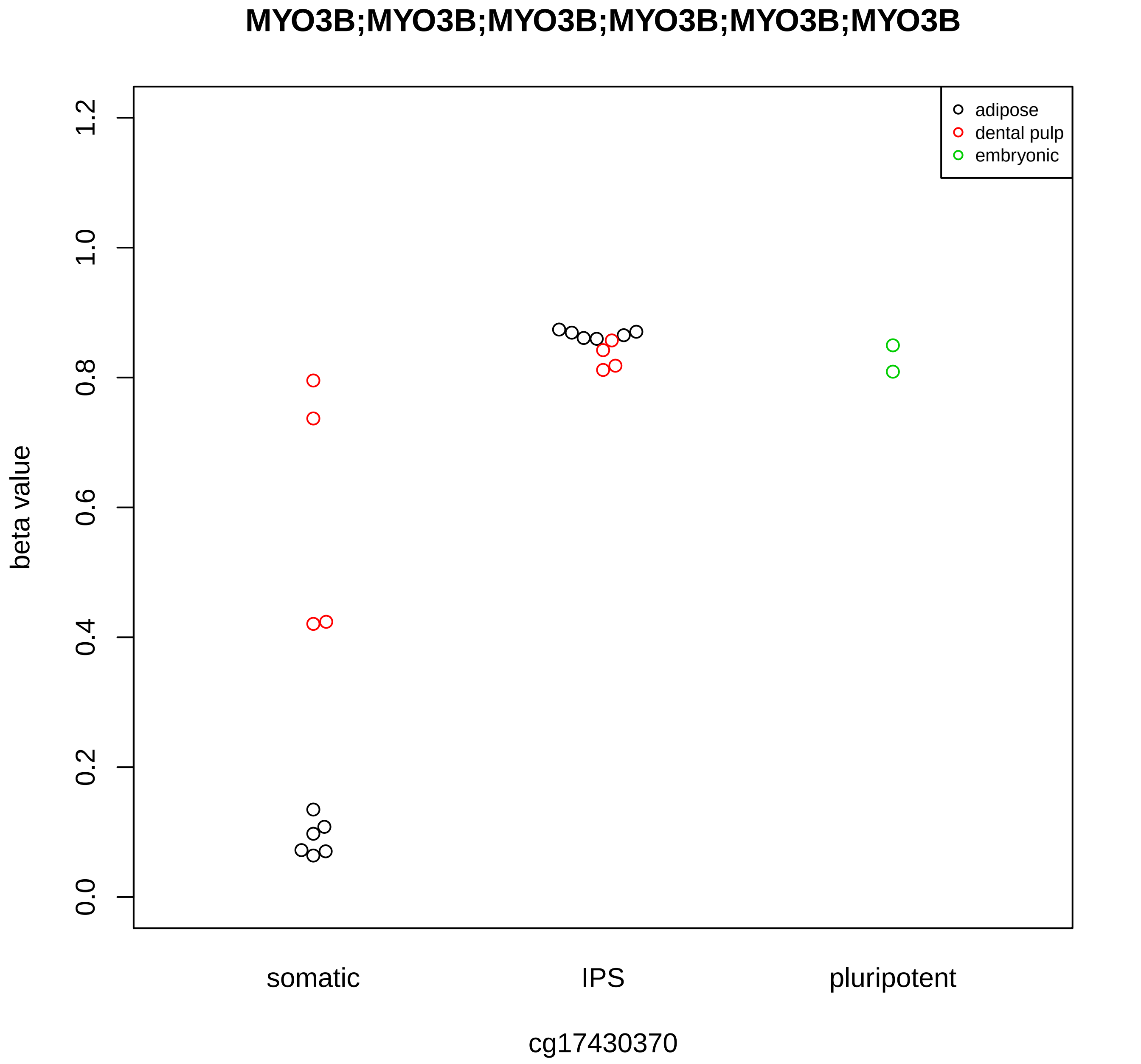
MYO3B;MYO3B;MYO3B;MYO3B;MYO3B;MYO3B
beta value
0.0
0.2
0.4
0.6
0.8
1.0
1.2
adipose
dental pulp
embryonic
somatic
IPS
pluripotent
cg17430370

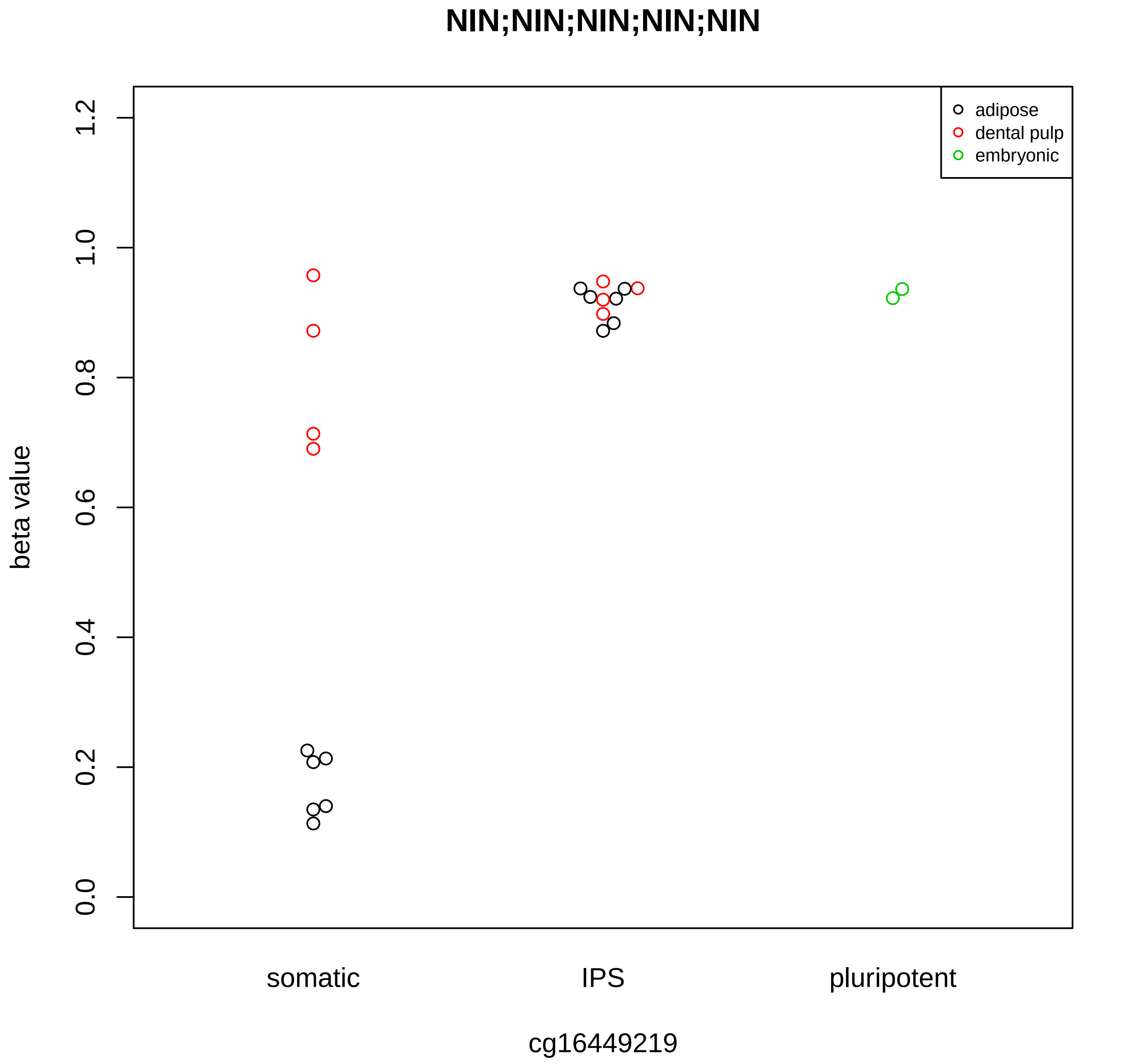

NIN;NIN;NIN;NIN;NIN
beta value
0.0
0.2
0.4
0.6
0.8
1.0
1.2
adipose
dental pulp
embryonic
somatic
IPS
pluripotent
cg16449219

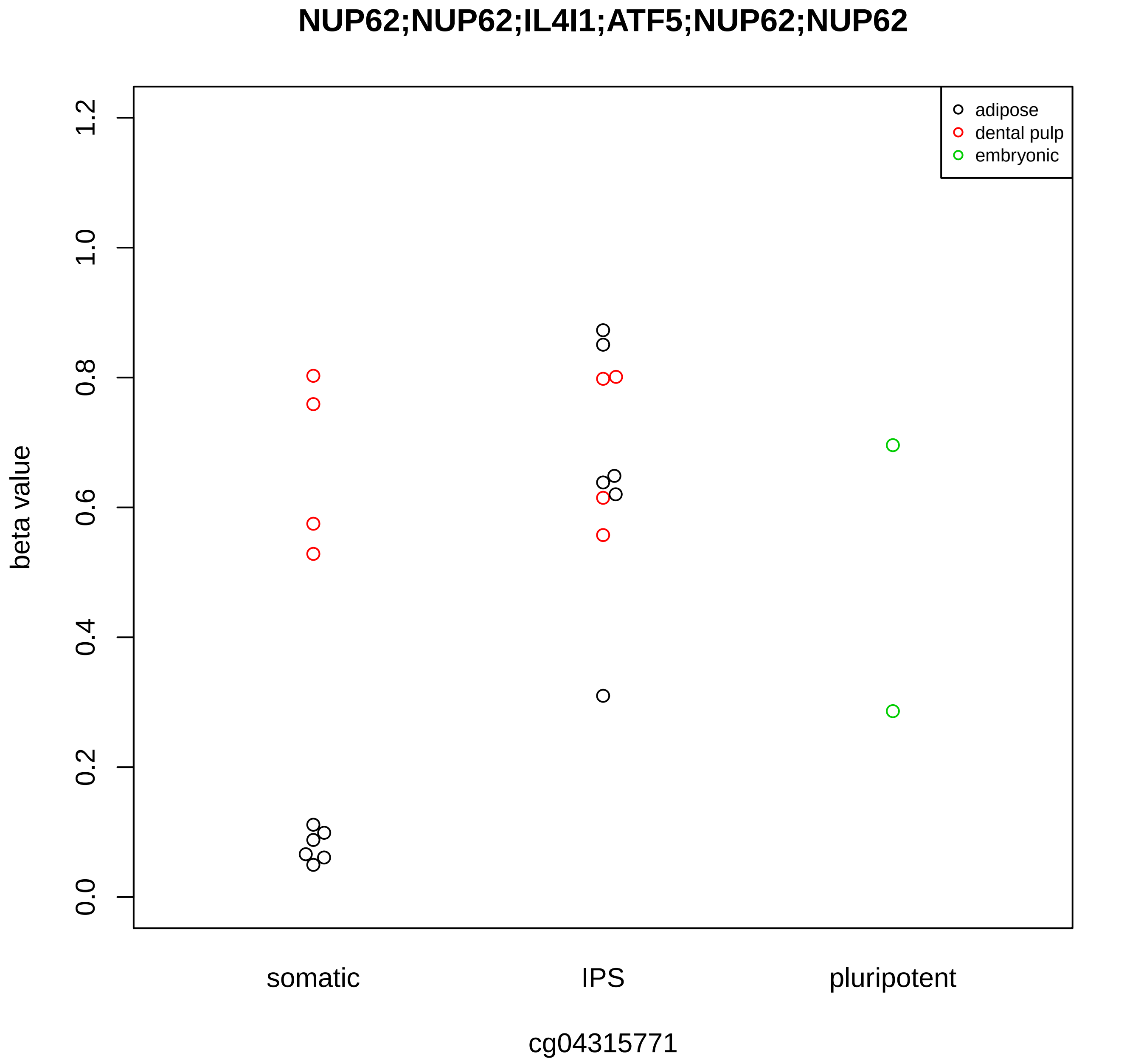

NUP62;NUP62;IL4I1;ATF5;NUP62;NUP62
beta value
0.0
0.2
0.4
0.6
0.8
1.0
1.2
adipose
dental pulp
embryonic
somatic
IPS
pluripotent
cg04315771

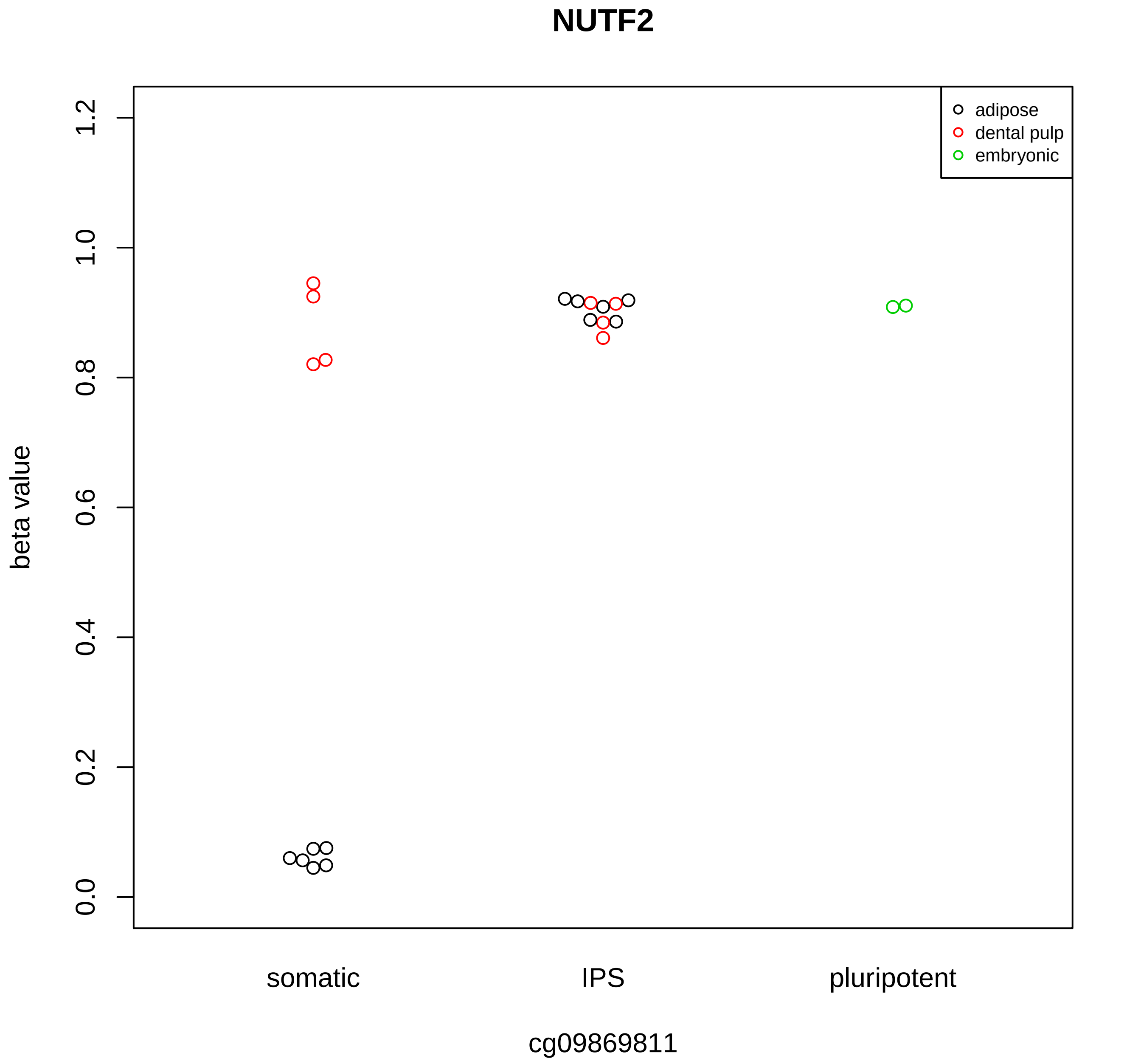
NUTF2
adipose
dental pulp
embryonic
beta value
0.0
0.2
0.4
0.6
0.8
1.0
1.2
somatic
IPS
pluripotent
cg09869811

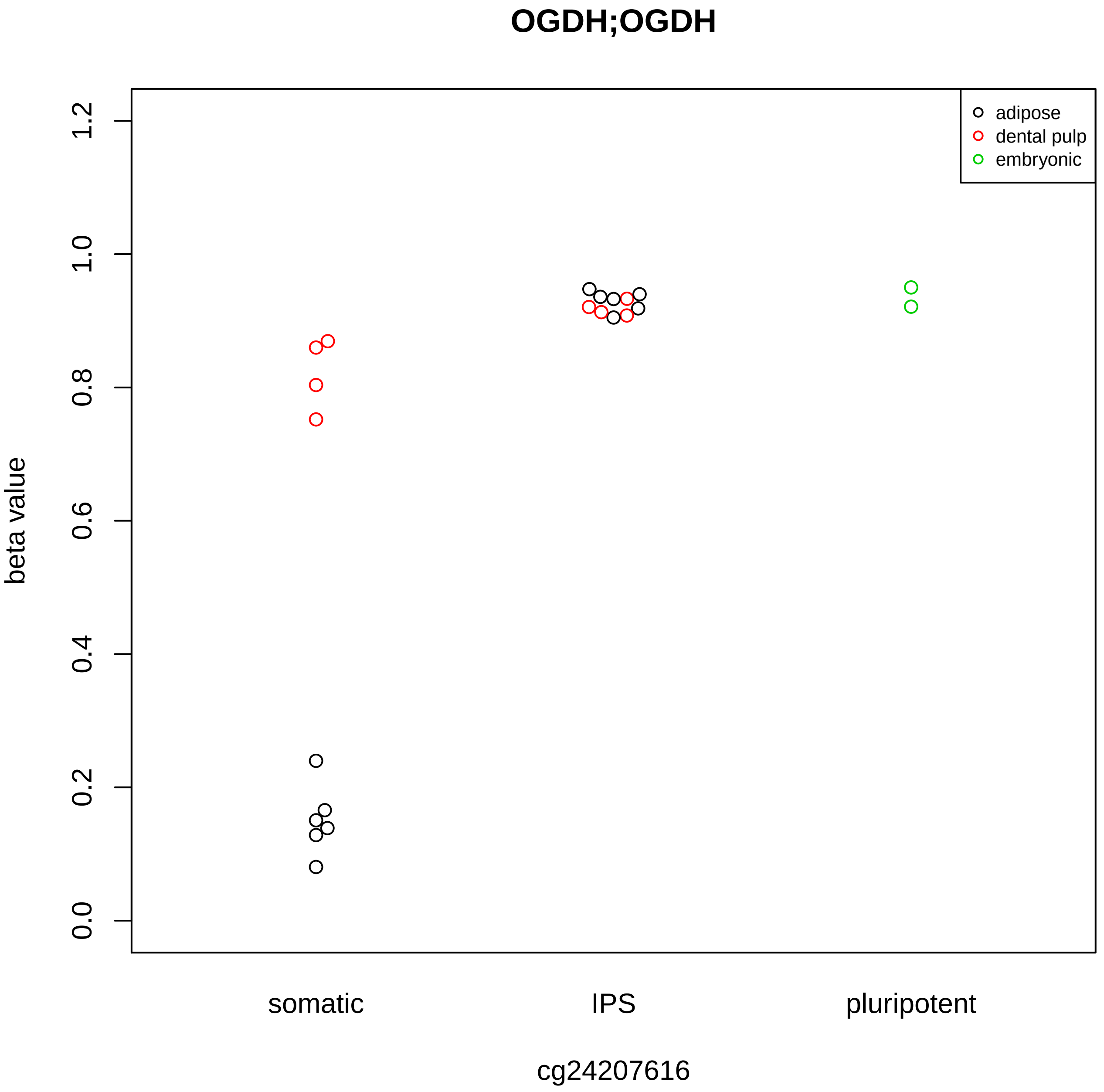
OGDH;OGDH
beta value
0.0
0.2
0.4
0.6
0.8
1.0
1.2
adipose
dental pulp
embryonic
somatic
IPS
pluripotent
cg24207616

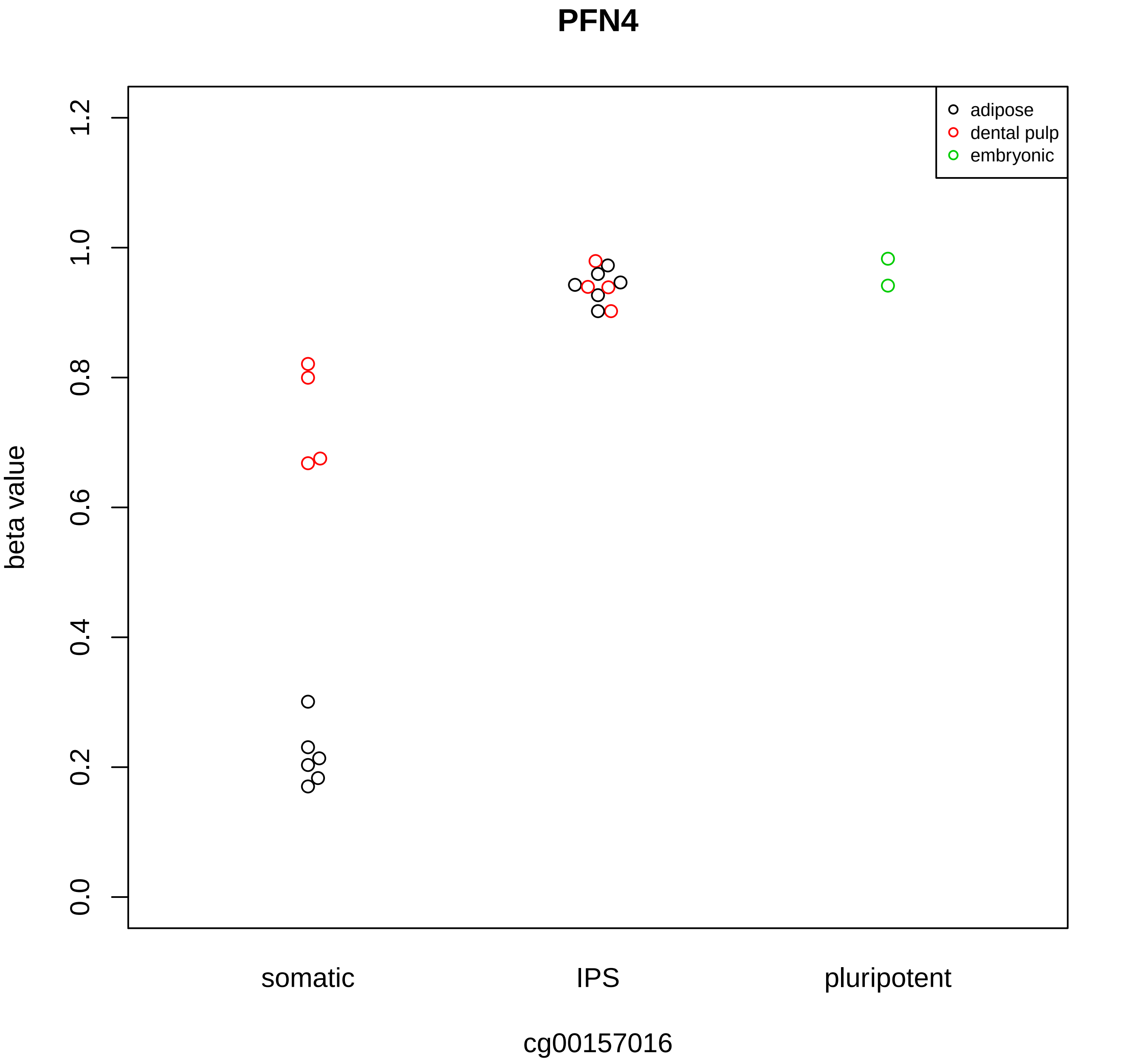
PFN4
adipose
dental pulp
embryonic
beta value
0.0
0.2
0.4
0.6
0.8
1.0
1.2
somatic
IPS
pluripotent
cg00157016

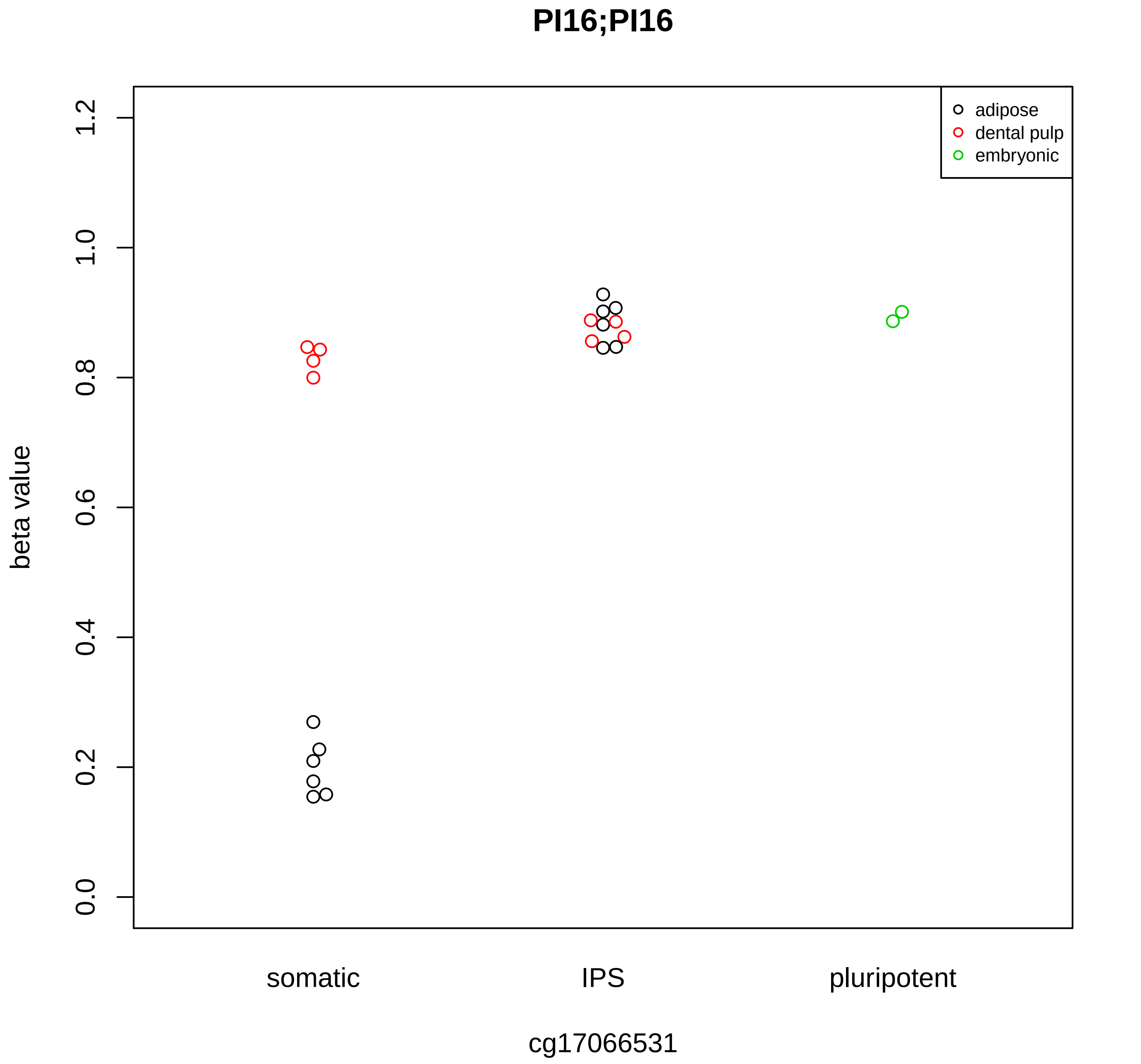
PI16;PI16
beta value
0.0
0.2
0.4
0.6
0.8
1.0
1.2
adipose
dental pulp
embryonic
somatic
IPS
pluripotent
cg17066531

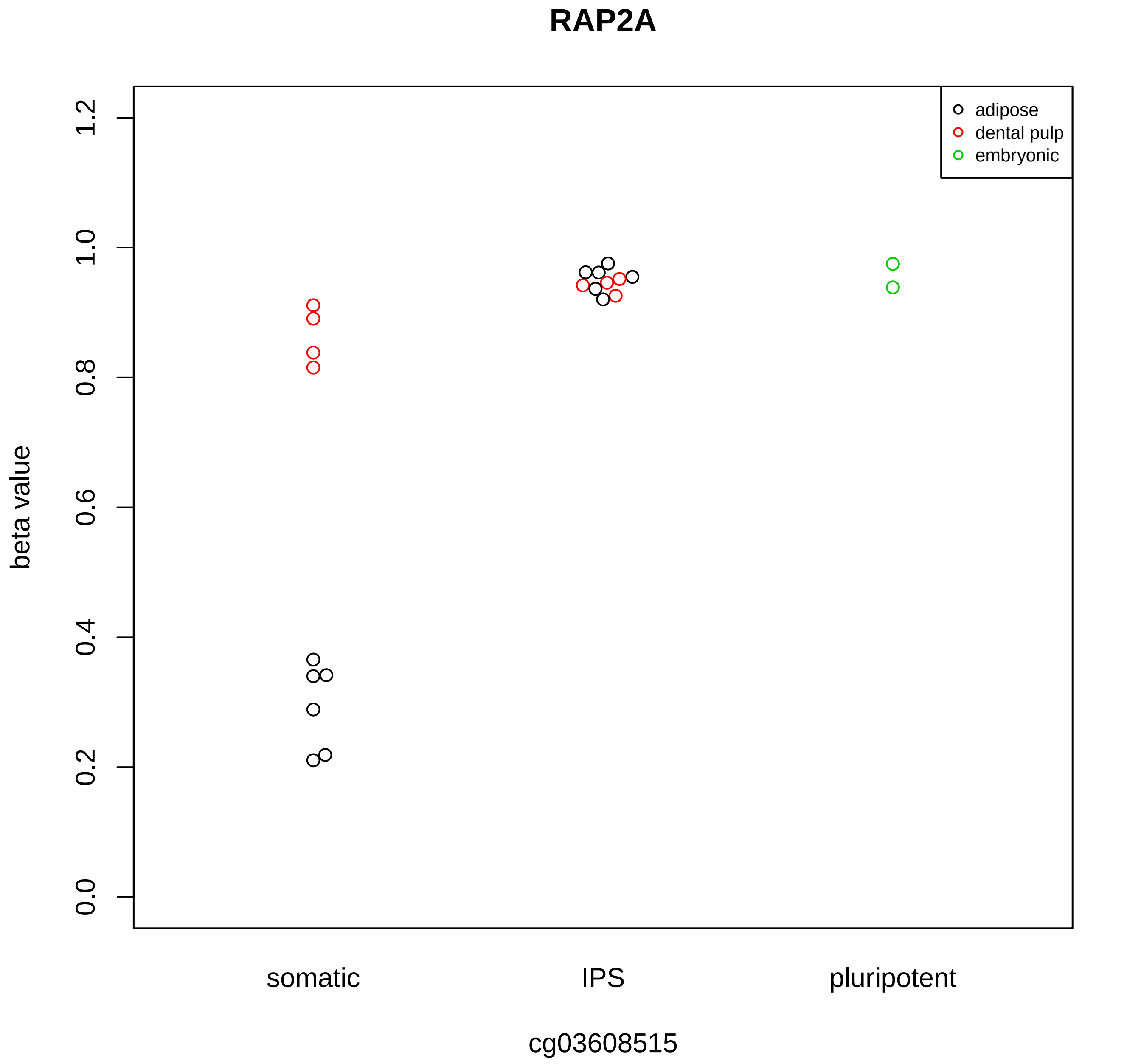

RAP2A
adipose
dental pulp
embryonic
beta value
0.0
0.2
0.4
0.6
0.8
1.0
1.2
somatic
IPS
pluripotent
cg03608515

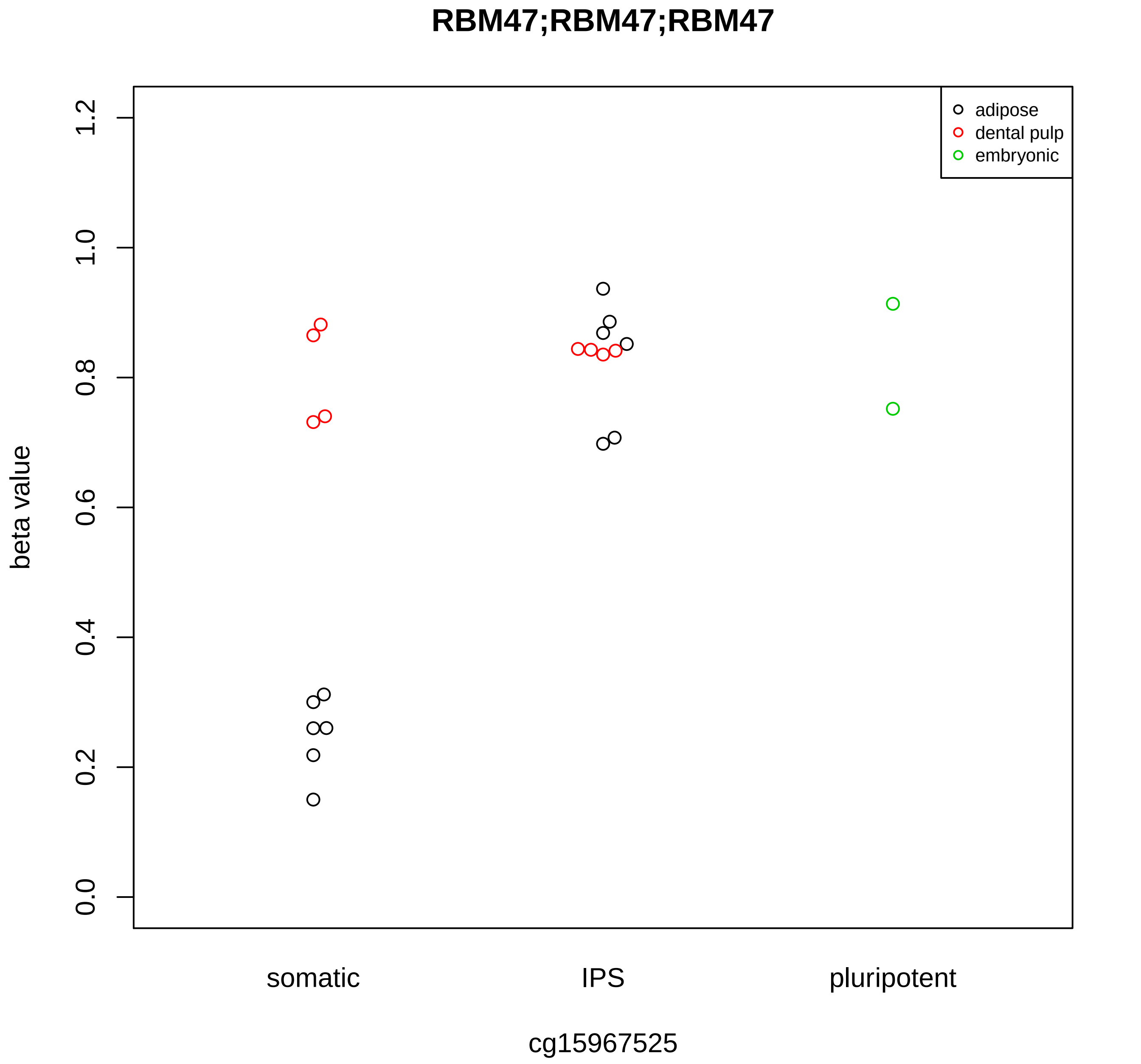
RBM47;RBM47;RBM47
beta value
0.0
0.2
0.4
0.6
0.8
1.0
1.2
adipose
dental pulp
embryonic
somatic
IPS
pluripotent
cg15967525

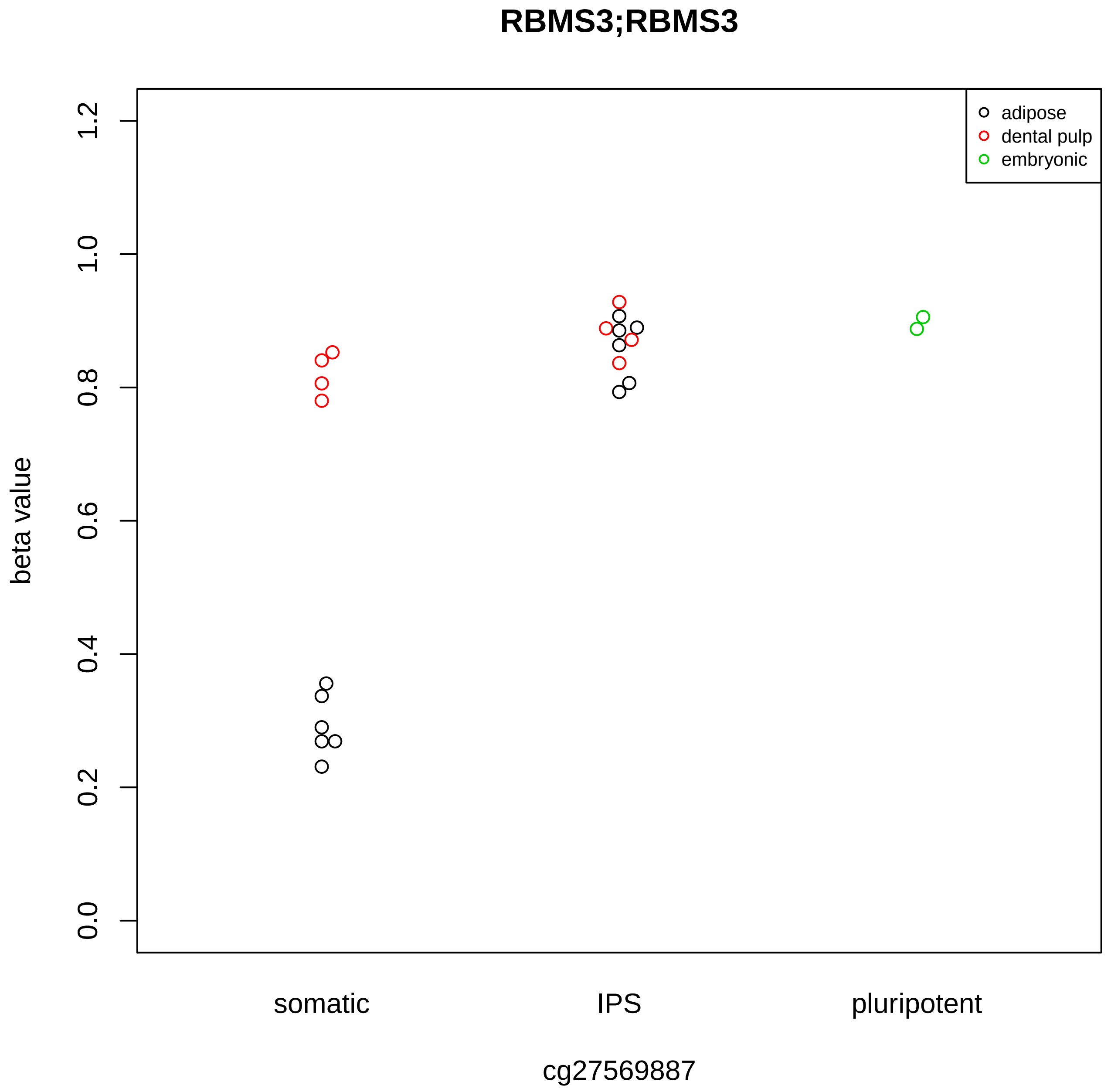

RBMS3;RBMS3
adipose
dental pulp
embryonic
beta value
0.0
0.2
0.4
0.6
0.8
1.0
1.2
somatic
IPS
pluripotent
cg27569887

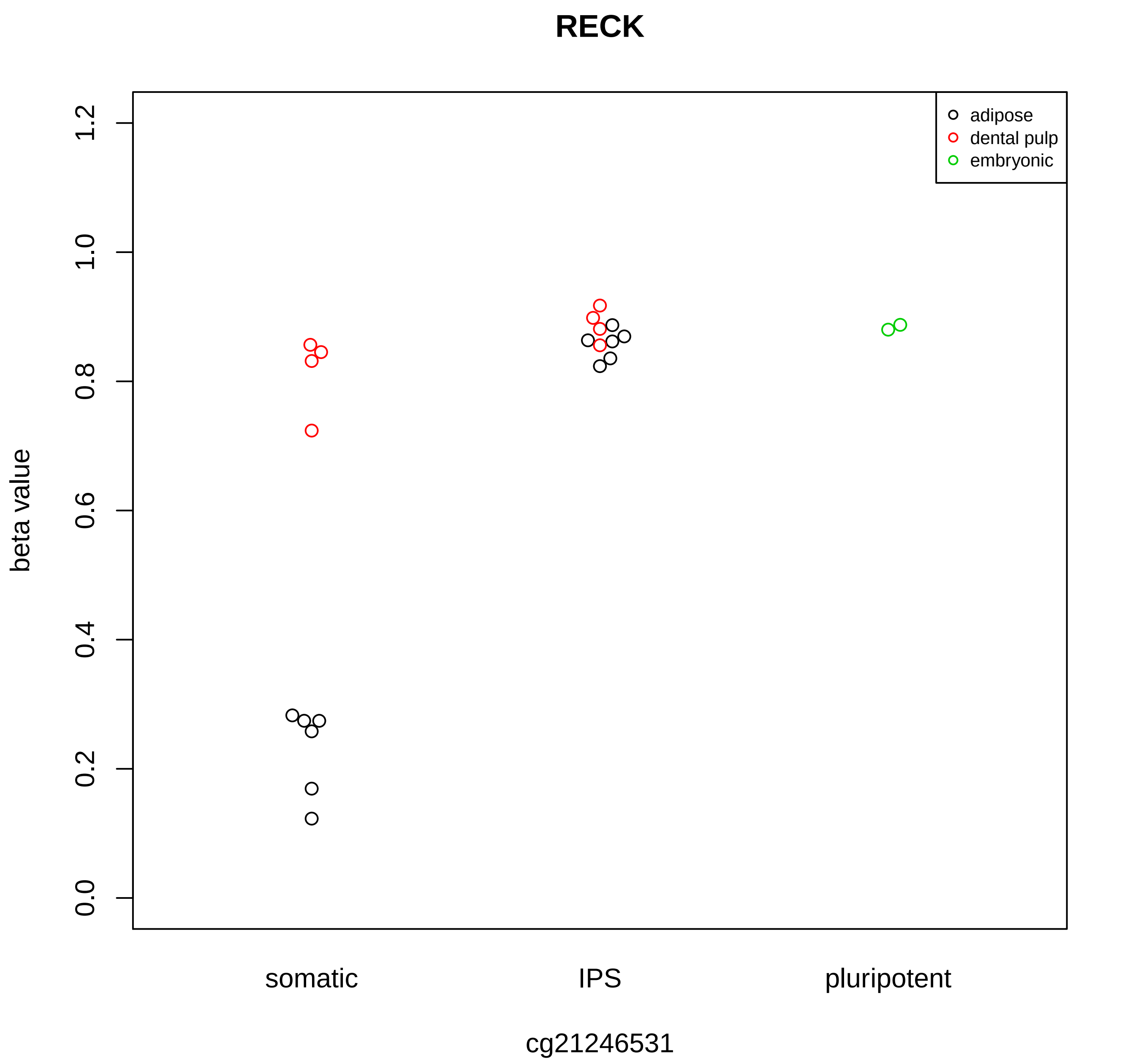
RECK
adipose
dental pulp
embryonic
beta value
0.0
0.2
0.4
0.6
0.8
1.0
1.2
somatic
IPS
pluripotent
cg21246531

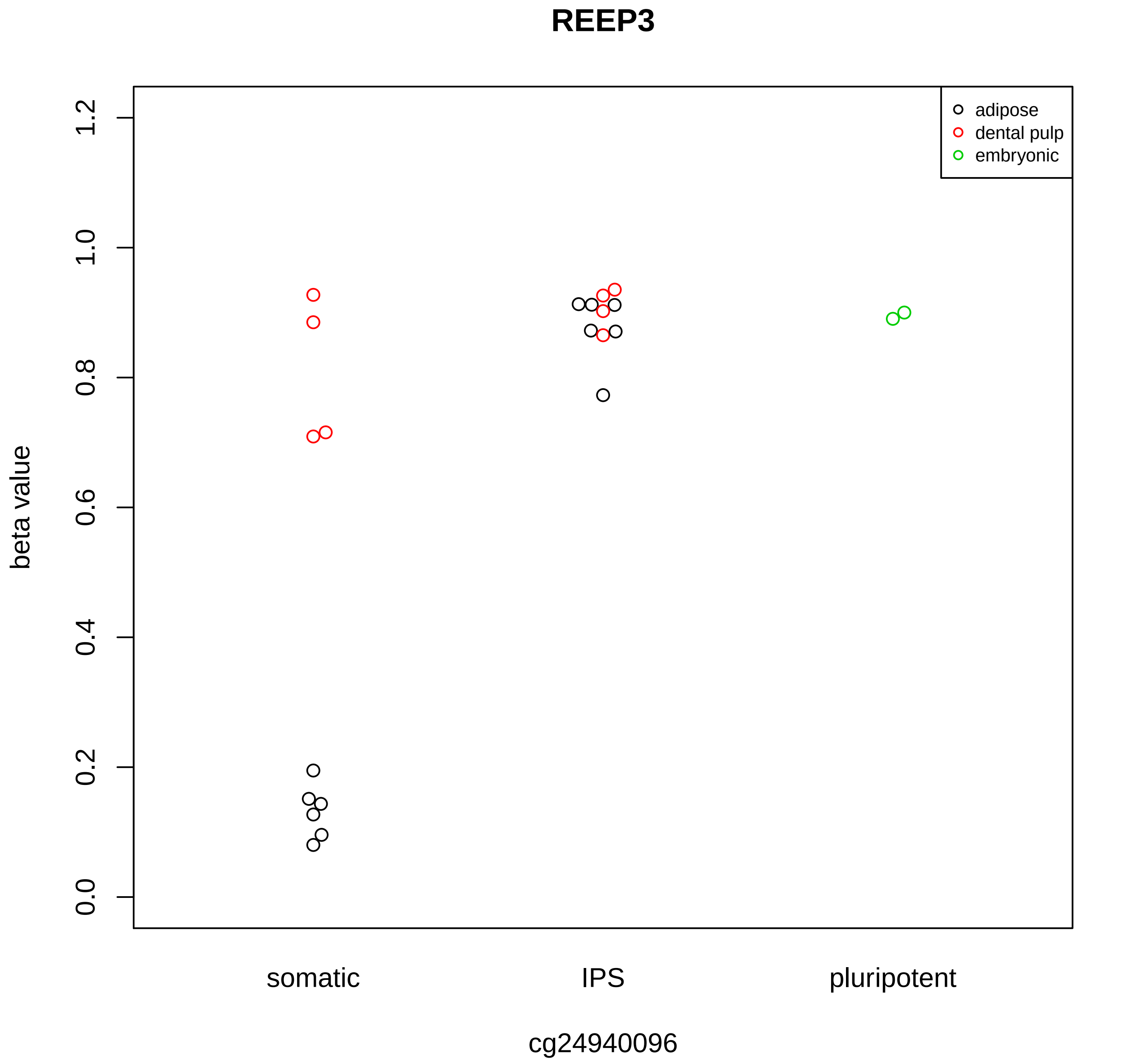

REEP3
adipose
dental pulp
embryonic
beta value
0.0
0.2
0.4
0.6
0.8
1.0
1.2
somatic
IPS
pluripotent
cg24940096

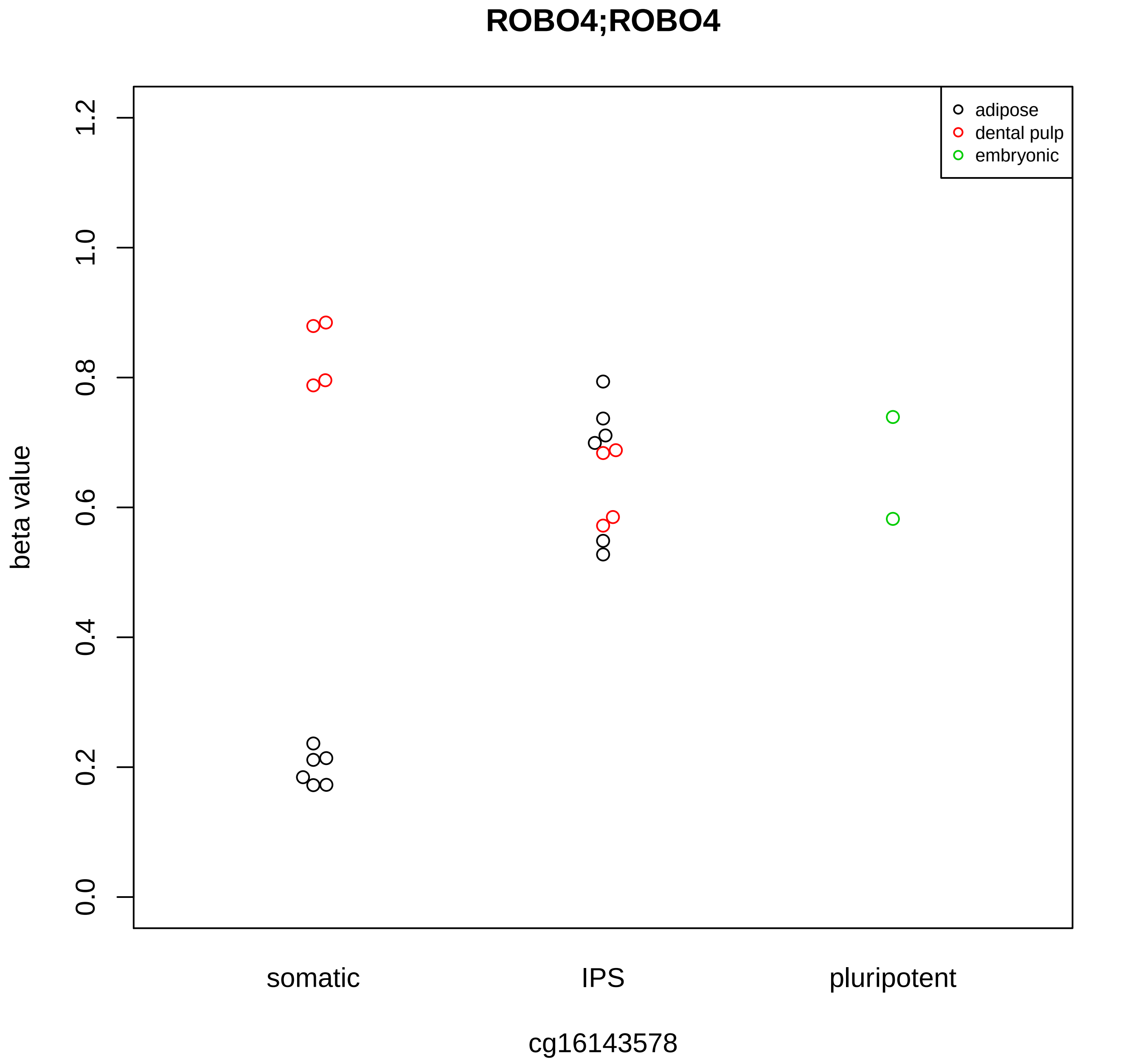
ROBO4;ROBO4
adipose
dental pulp
embryonic
beta value
0.0
0.2
0.4
0.6
0.8
1.0
1.2
somatic
IPS
pluripotent
cg16143578

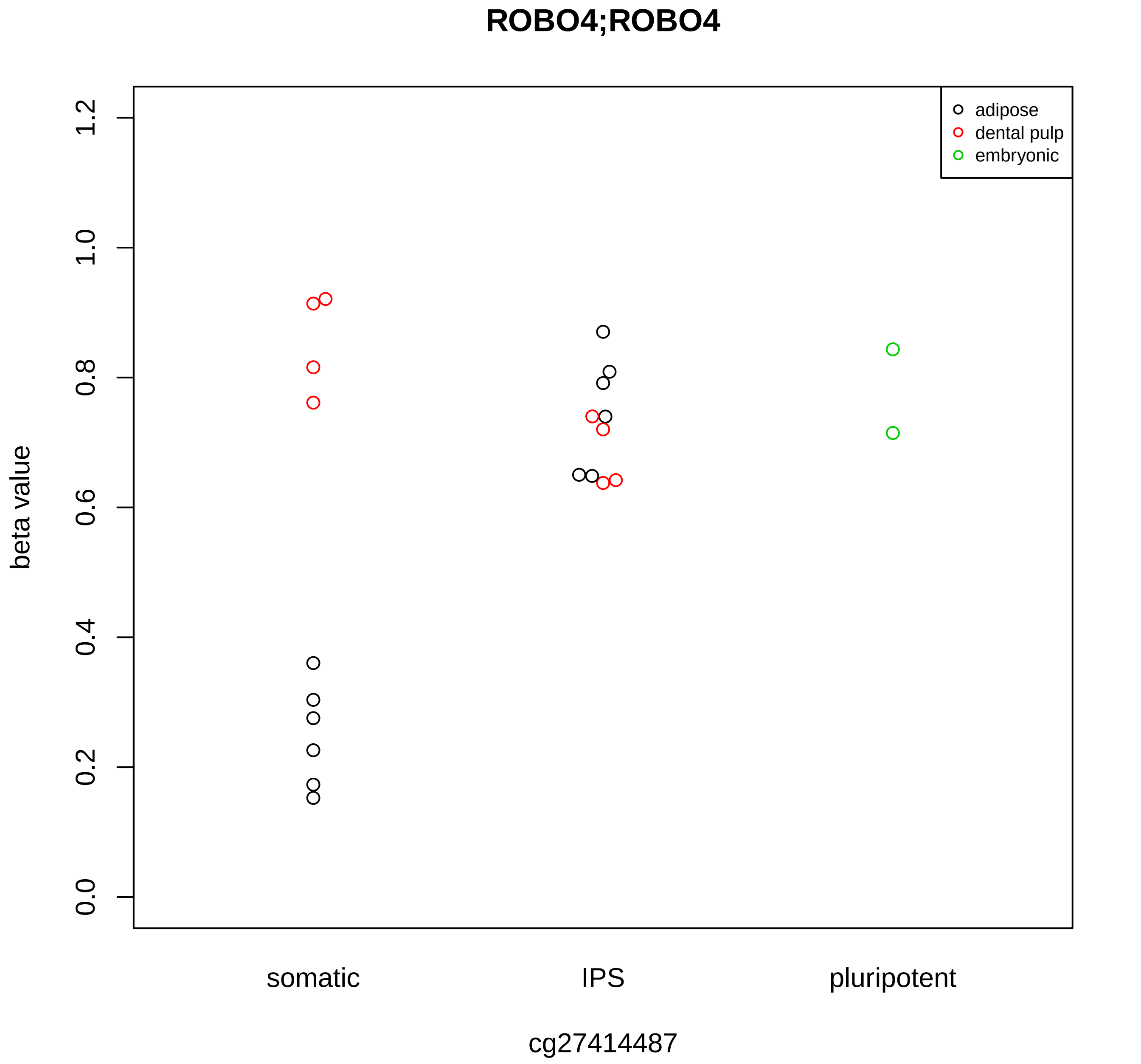
ROBO4;ROBO4
adipose
dental pulp
embryonic
beta value
0.0
0.2
0.4
0.6
0.8
1.0
1.2
somatic
IPS
pluripotent
cg27414487

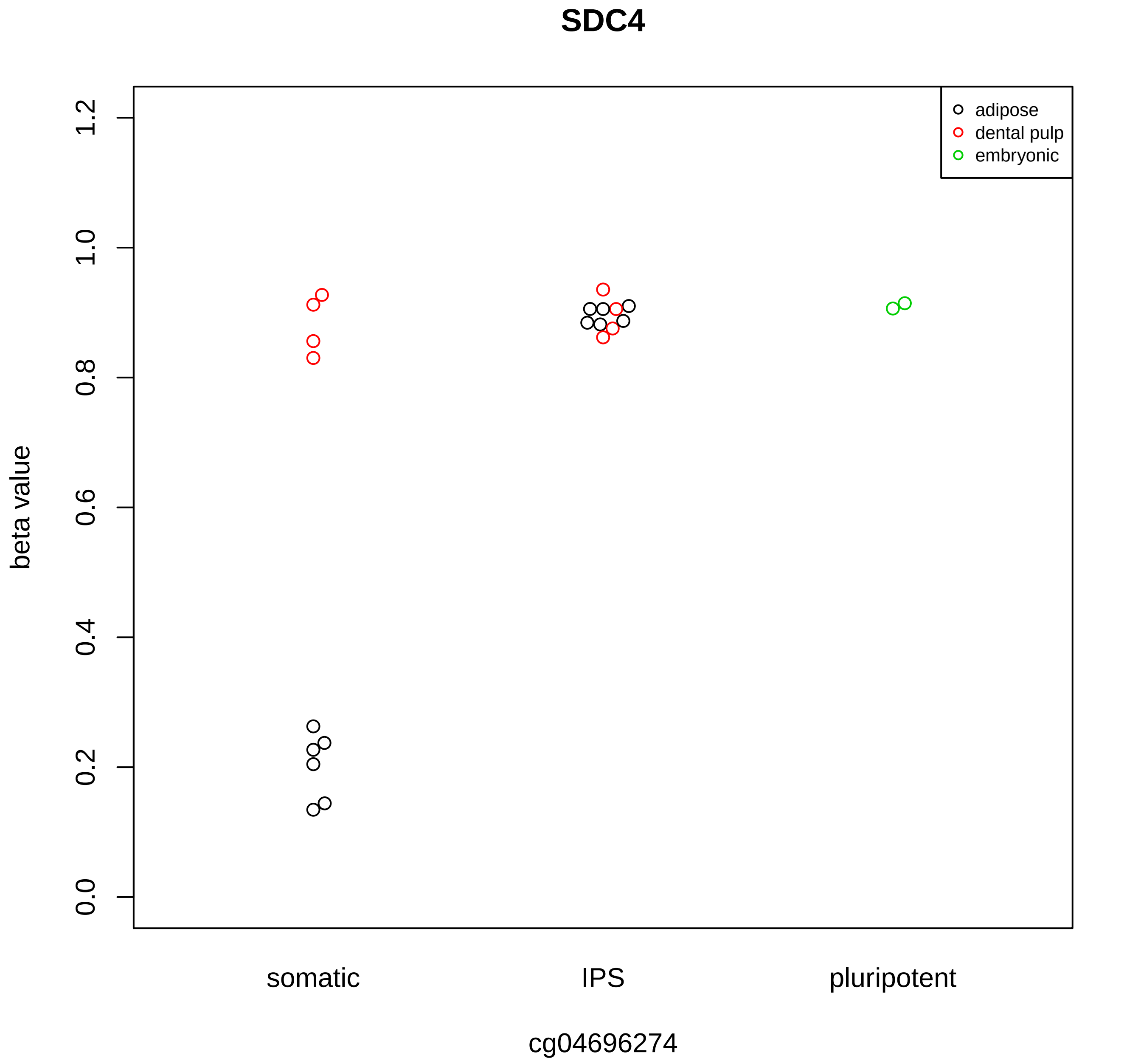
SDC4
beta value
0.0
0.2
0.4
0.6
0.8
1.0
1.2
adipose
dental pulp
embryonic
somatic
IPS
pluripotent
cg04696274

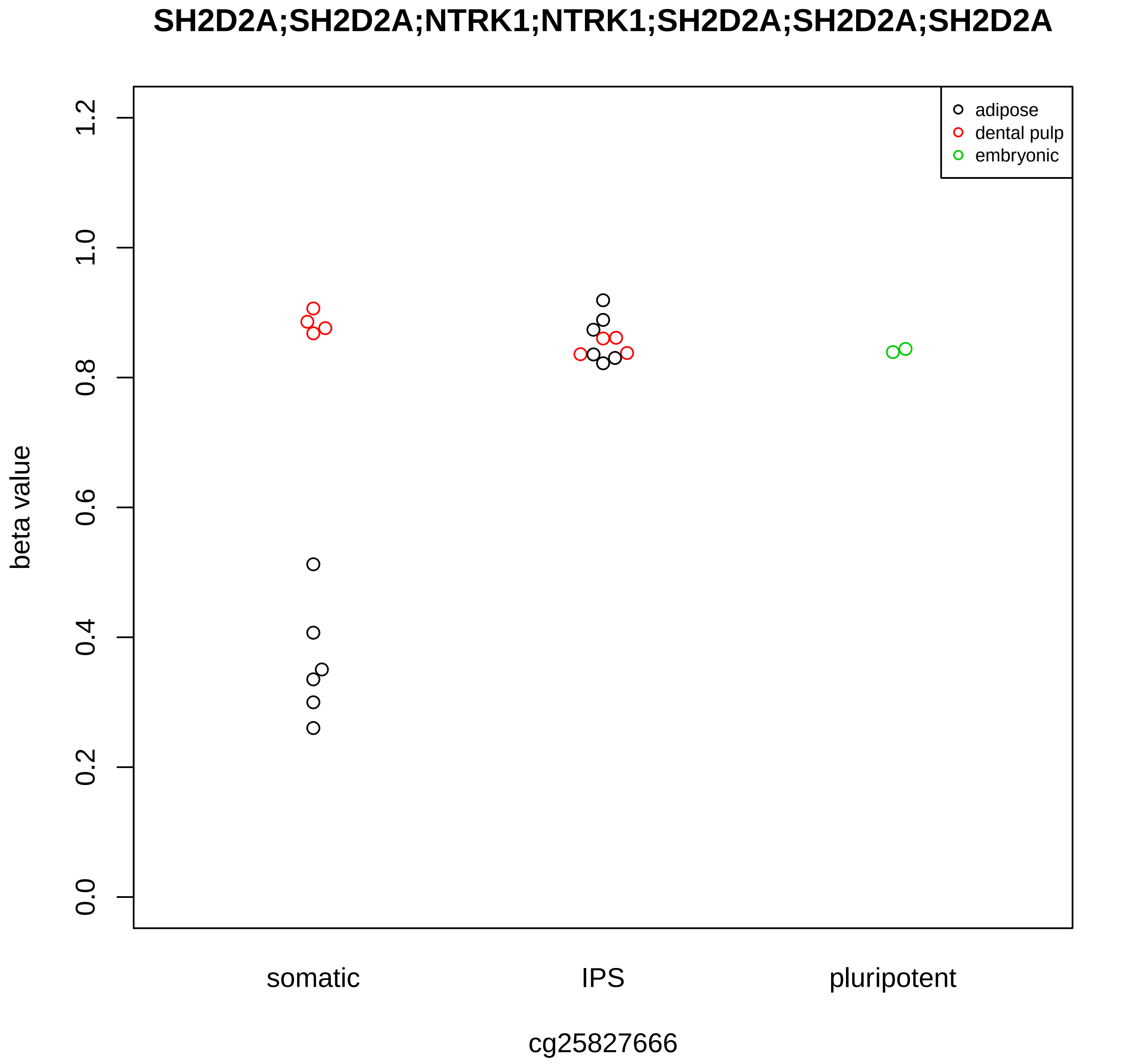

SH2D2A;SH2D2A;NTRK1;NTRK1;SH2D2A;SH2D2A;SH2D2A
beta value
0.0
0.2
0.4
0.6
0.8
1.0
1.2
adipose
dental pulp
embryonic
somatic
IPS
pluripotent
cg25827666

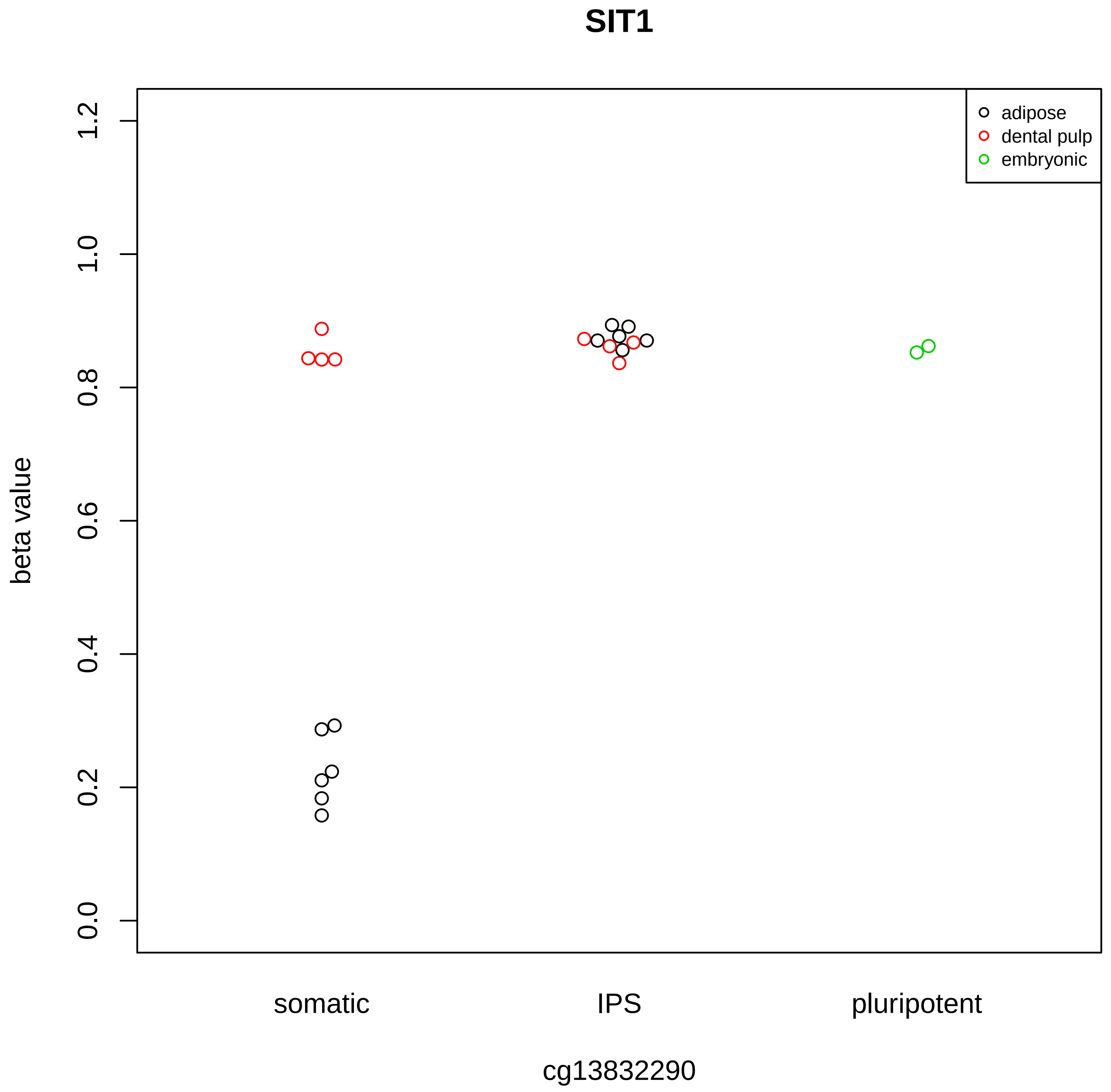
SIT1
adipose
dental pulp
embryonic
beta value
0.0
0.2
0.4
0.6
0.8
1.0
1.2
somatic
IPS
pluripotent
cg13832290

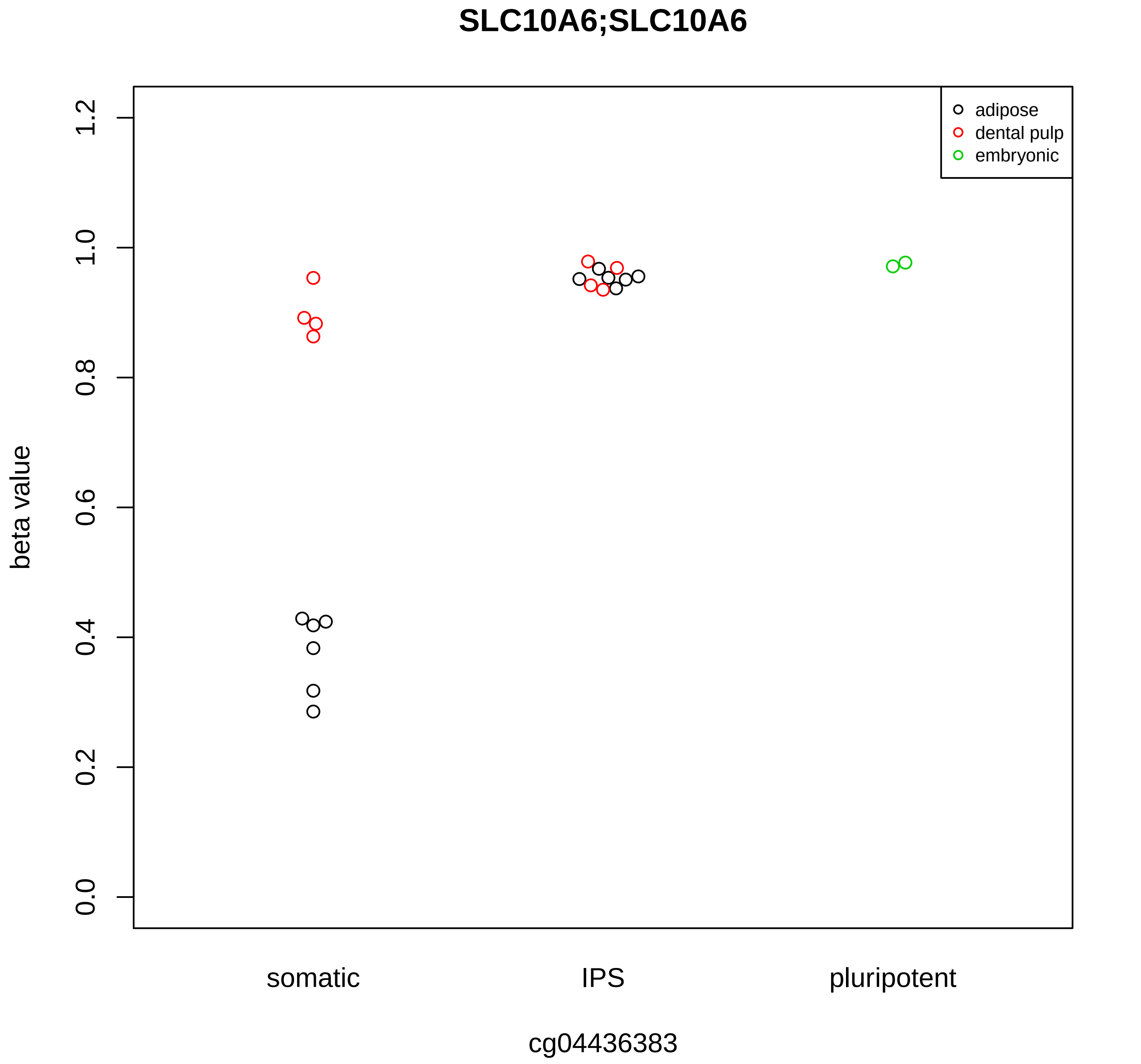

SLC10A6;SLC10A6
beta value
0.0
0.2
0.4
0.6
0.8
1.0
1.2
adipose
dental pulp
embryonic
somatic
IPS
pluripotent
cg04436383

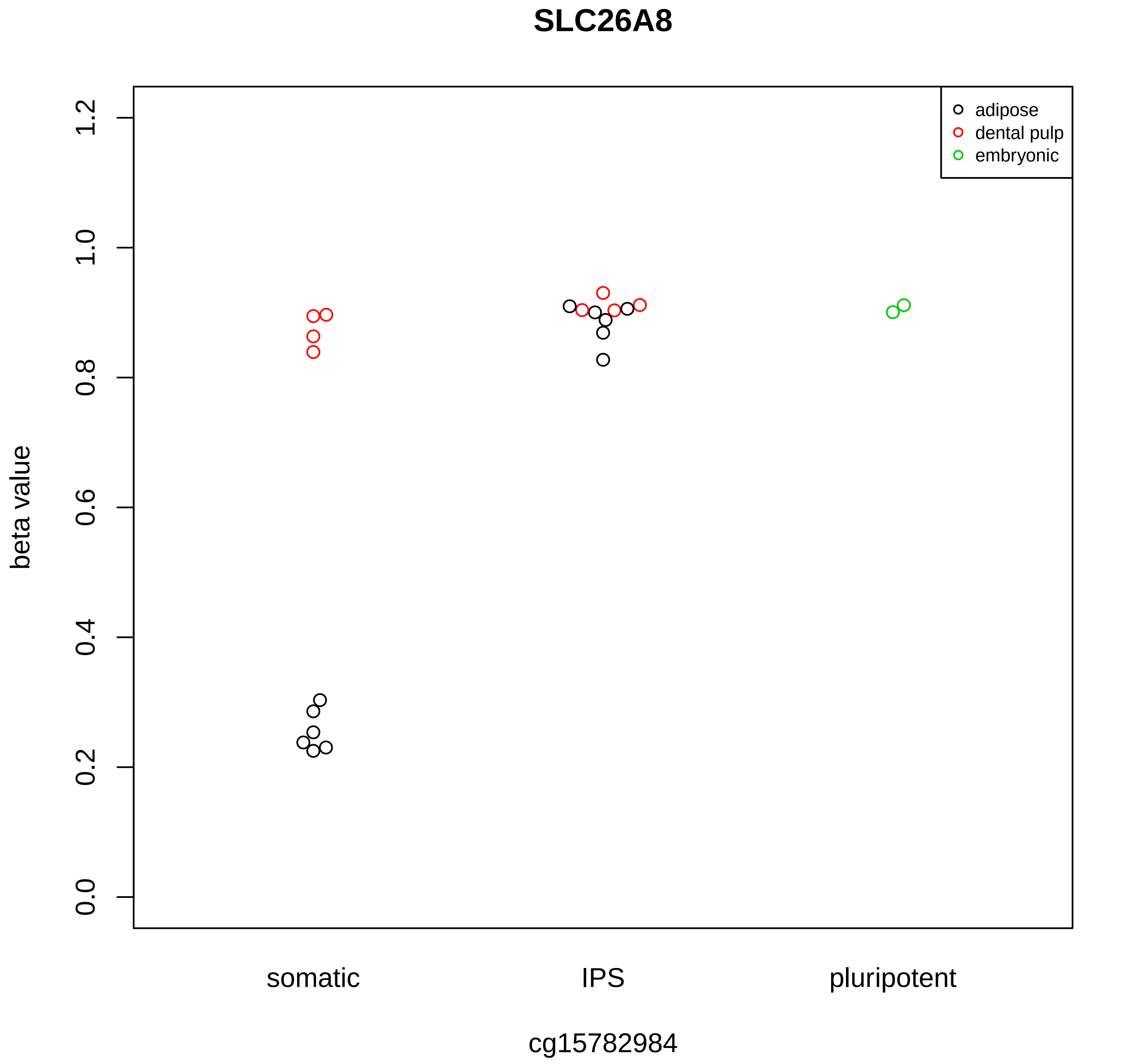

SLC26A8
adipose
dental pulp
embryonic
beta value
0.0
0.2
0.4
0.6
0.8
1.0
1.2
somatic
IPS
pluripotent
cg15782984

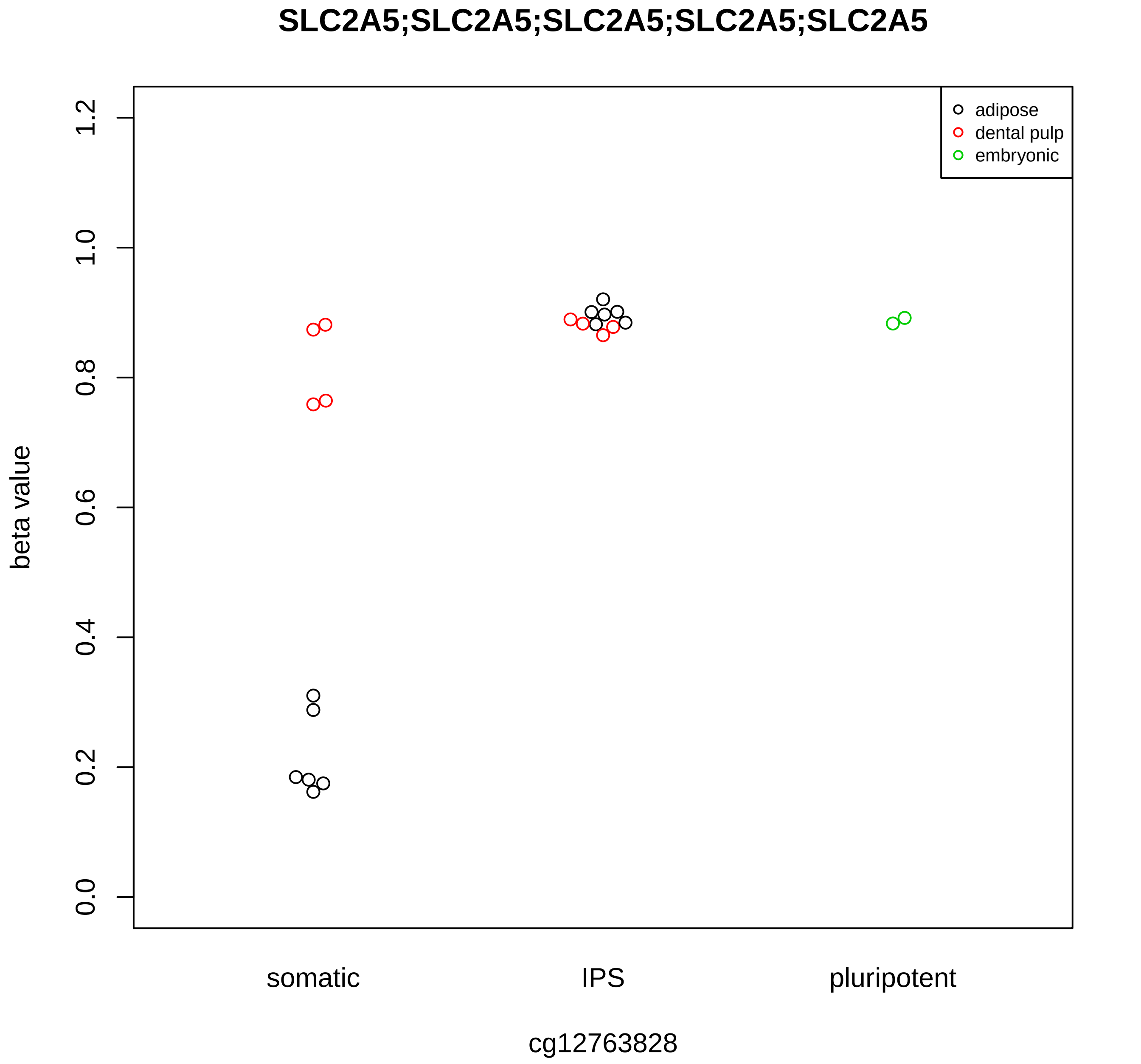

SLC2A5;SLC2A5;SLC2A5;SLC2A5;SLC2A5
adipose
dental pulp
embryonic
beta value
0.0
0.2
0.4
0.6
0.8
1.0
1.2
somatic
IPS
pluripotent
cg12763828

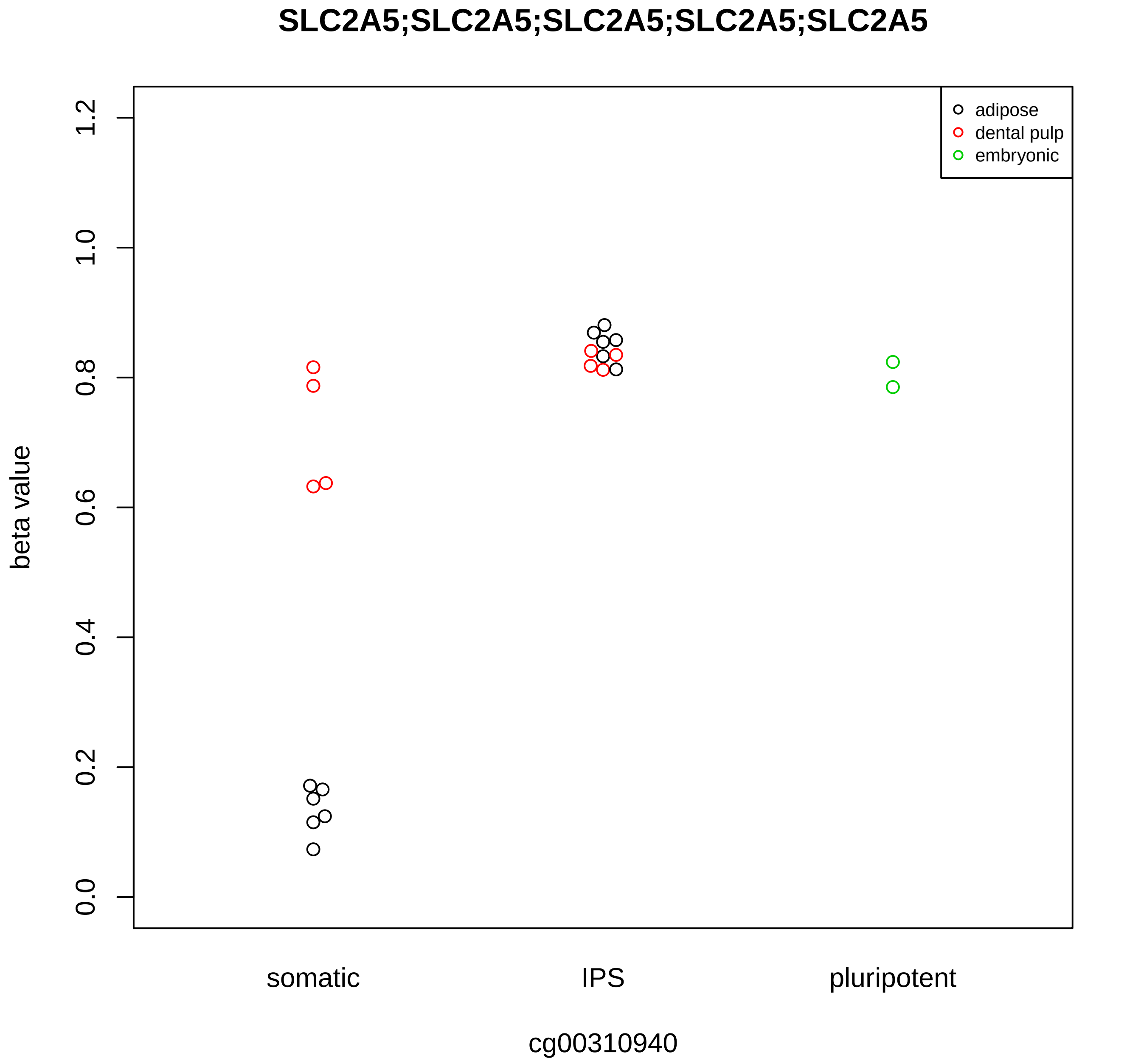

SLC2A5;SLC2A5;SLC2A5;SLC2A5;SLC2A5
beta value
0.0
0.2
0.4
0.6
0.8
1.0
1.2
adipose
dental pulp
embryonic
somatic
IPS
pluripotent
cg00310940

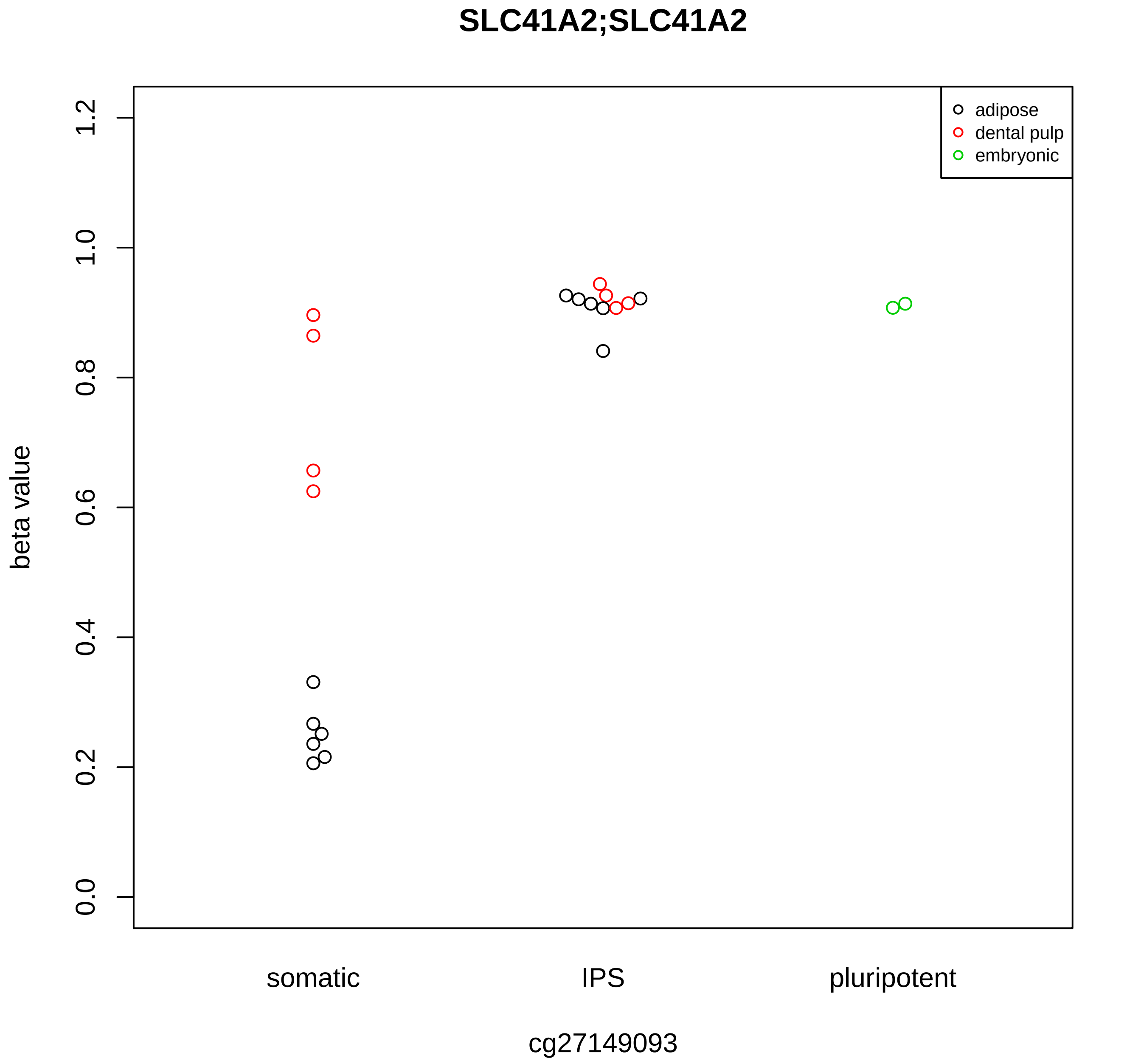

SLC41A2;SLC41A2
beta value
0.0
0.2
0.4
0.6
0.8
1.0
1.2
adipose
dental pulp
embryonic
somatic
IPS
pluripotent
cg27149093

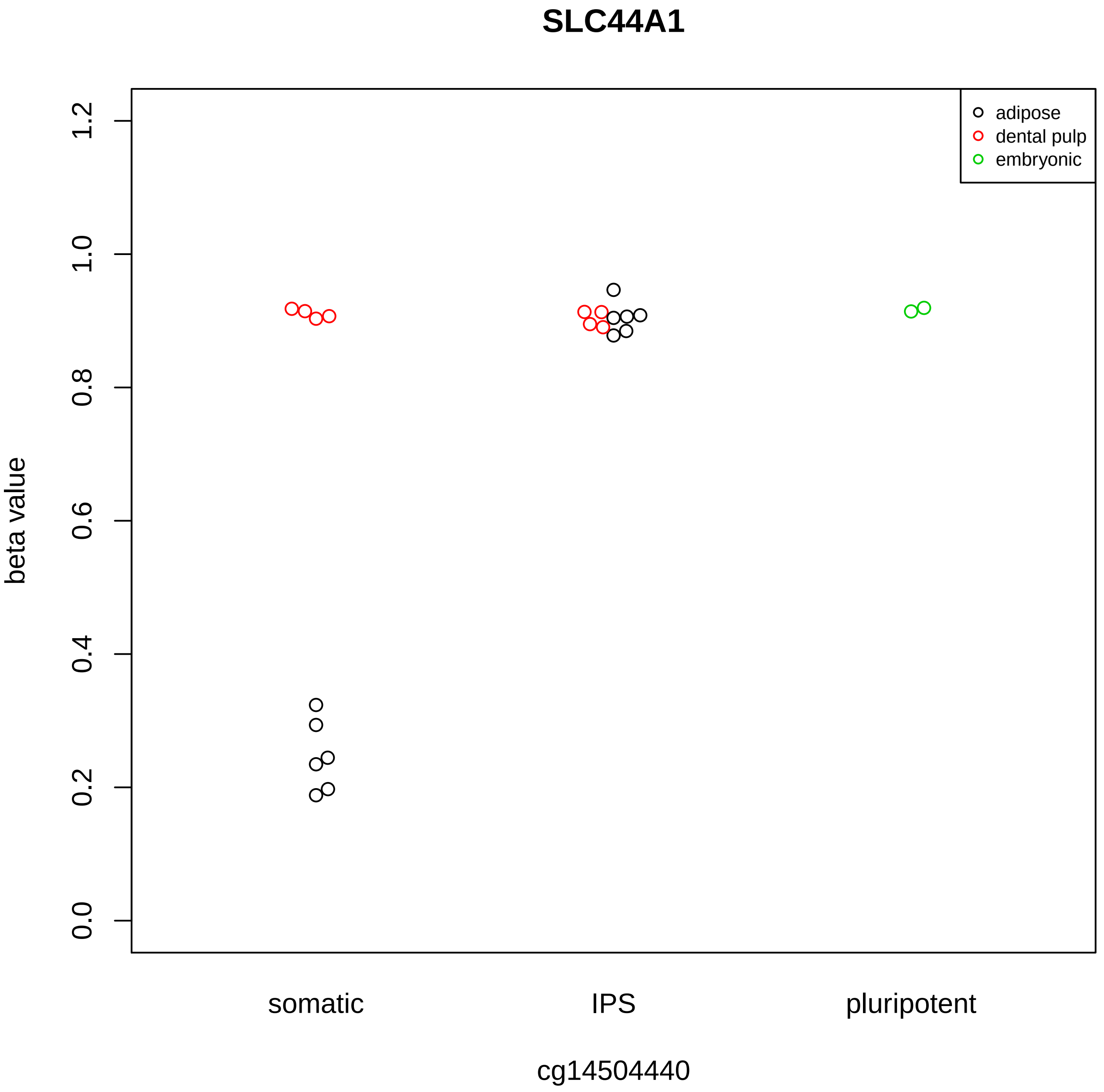

SLC44A1
beta value
1.2
1.0
0.8
0.6
0.4
0.2
0.0
adipose
dental pulp
embryonic
somatic
IPS
pluripotent
cg14504440

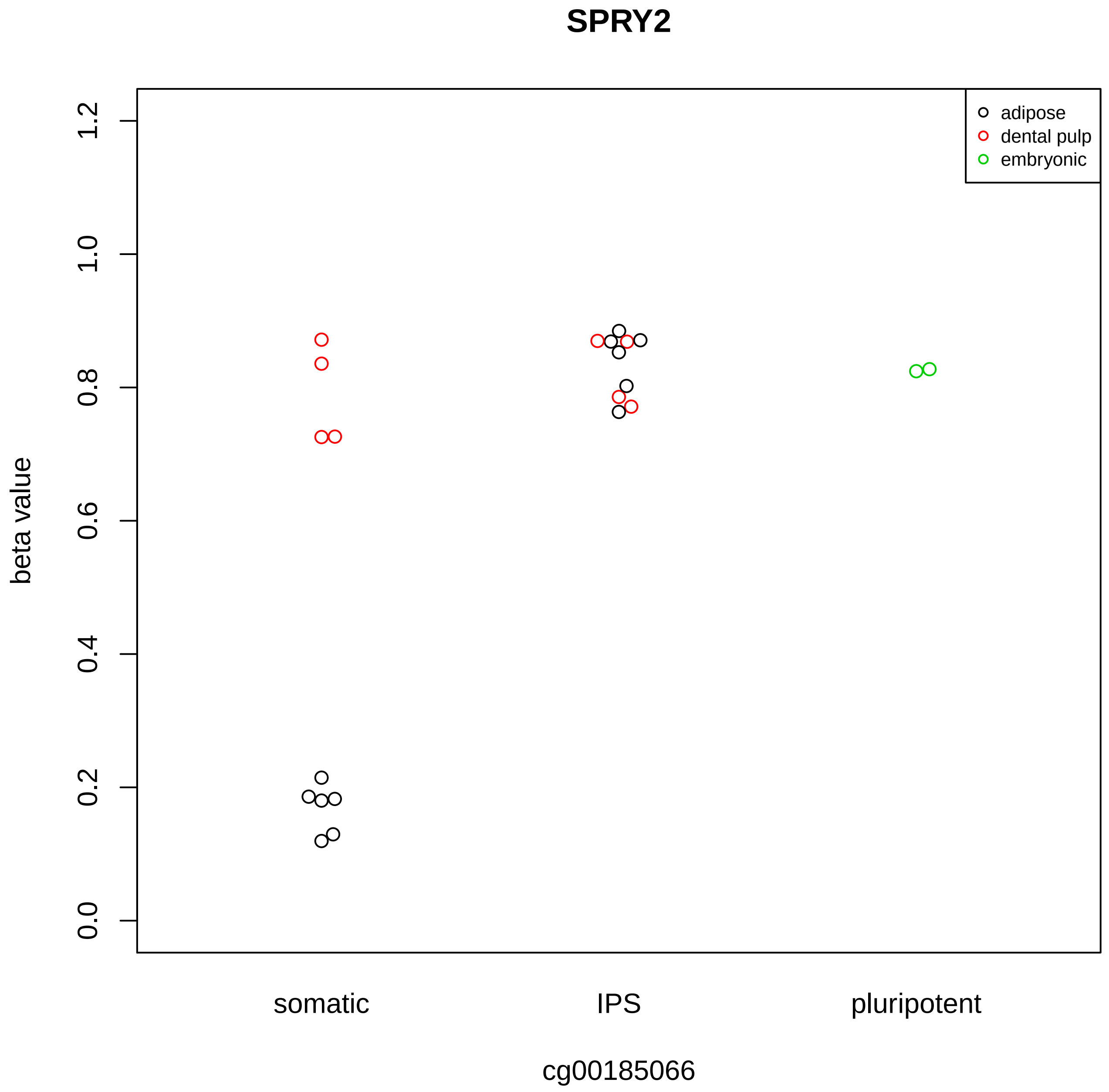
SPRY2
beta value
0.0
0.2
0.4
0.6
0.8
1.0
1.2
adipose
dental pulp
embryonic
somatic
IPS
pluripotent
cg00185066

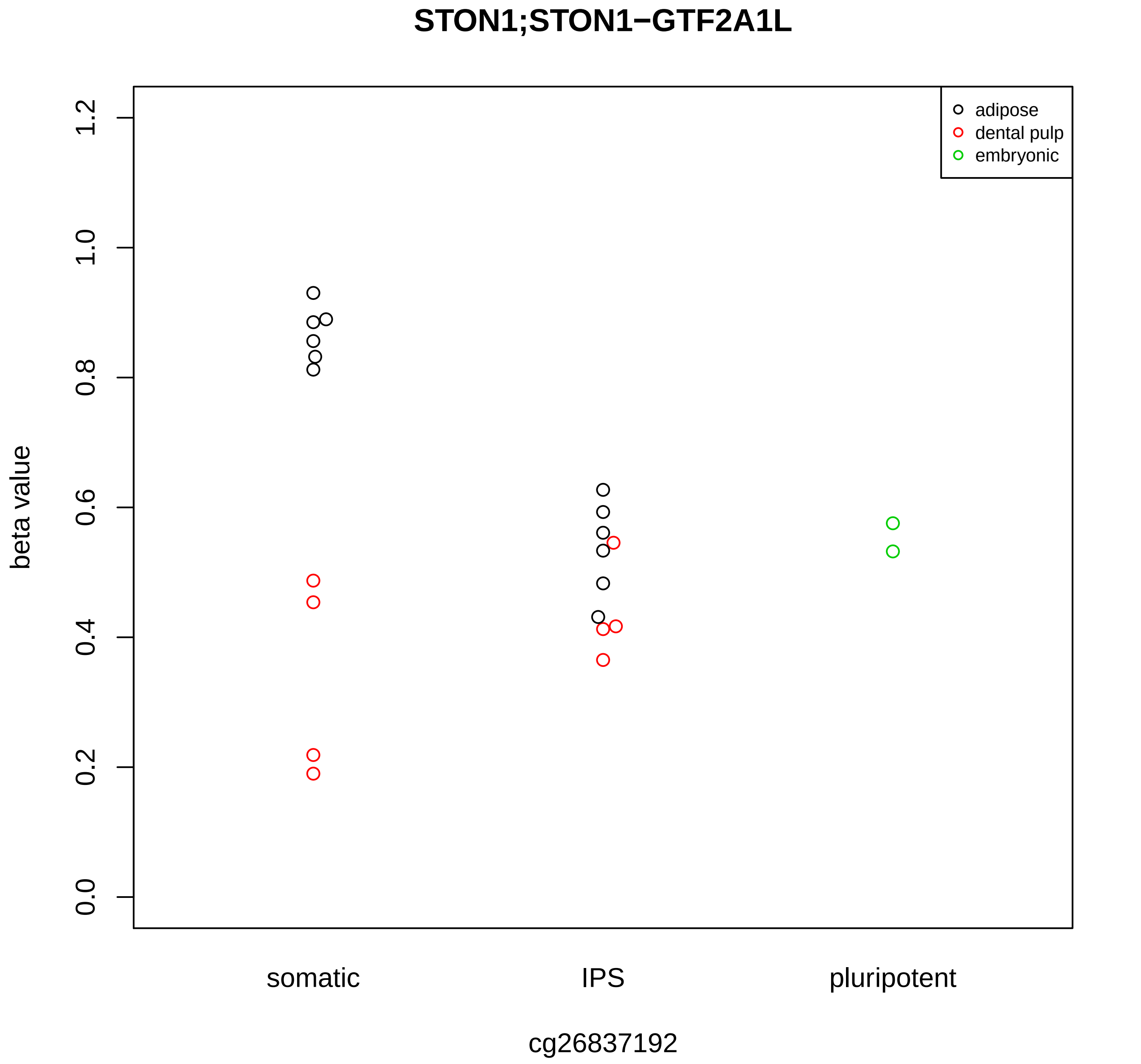
STON1;STON1−GTF2A1L
beta value
0.0
0.2
0.4
0.6
0.8
1.0
1.2
adipose
dental pulp
embryonic
somatic
IPS
pluripotent
cg26837192

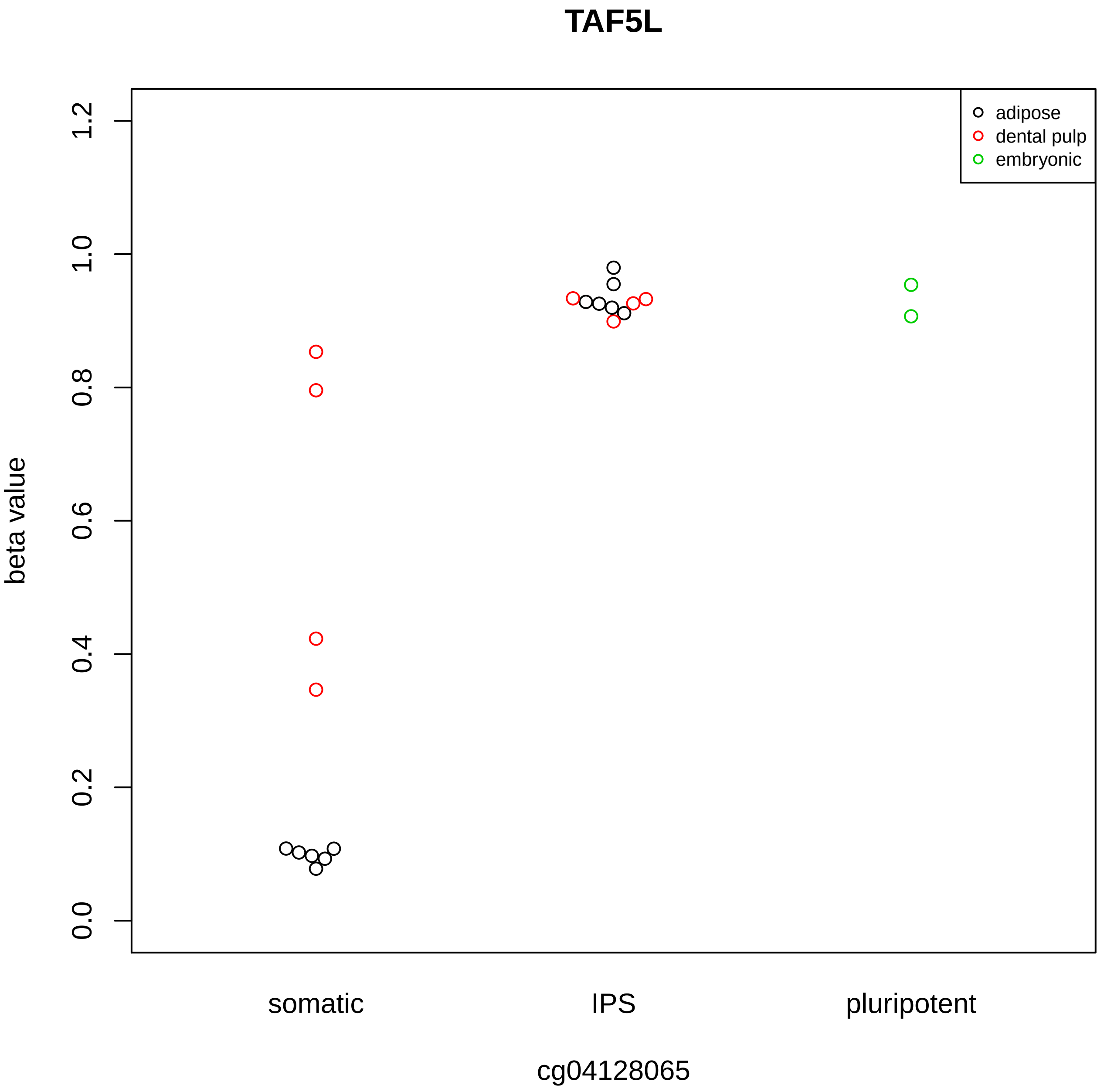
TAF5L
beta value
0.0
0.2
0.4
0.6
0.8
1.0
1.2
adipose
dental pulp
embryonic
somatic
IPS
pluripotent
cg04128065

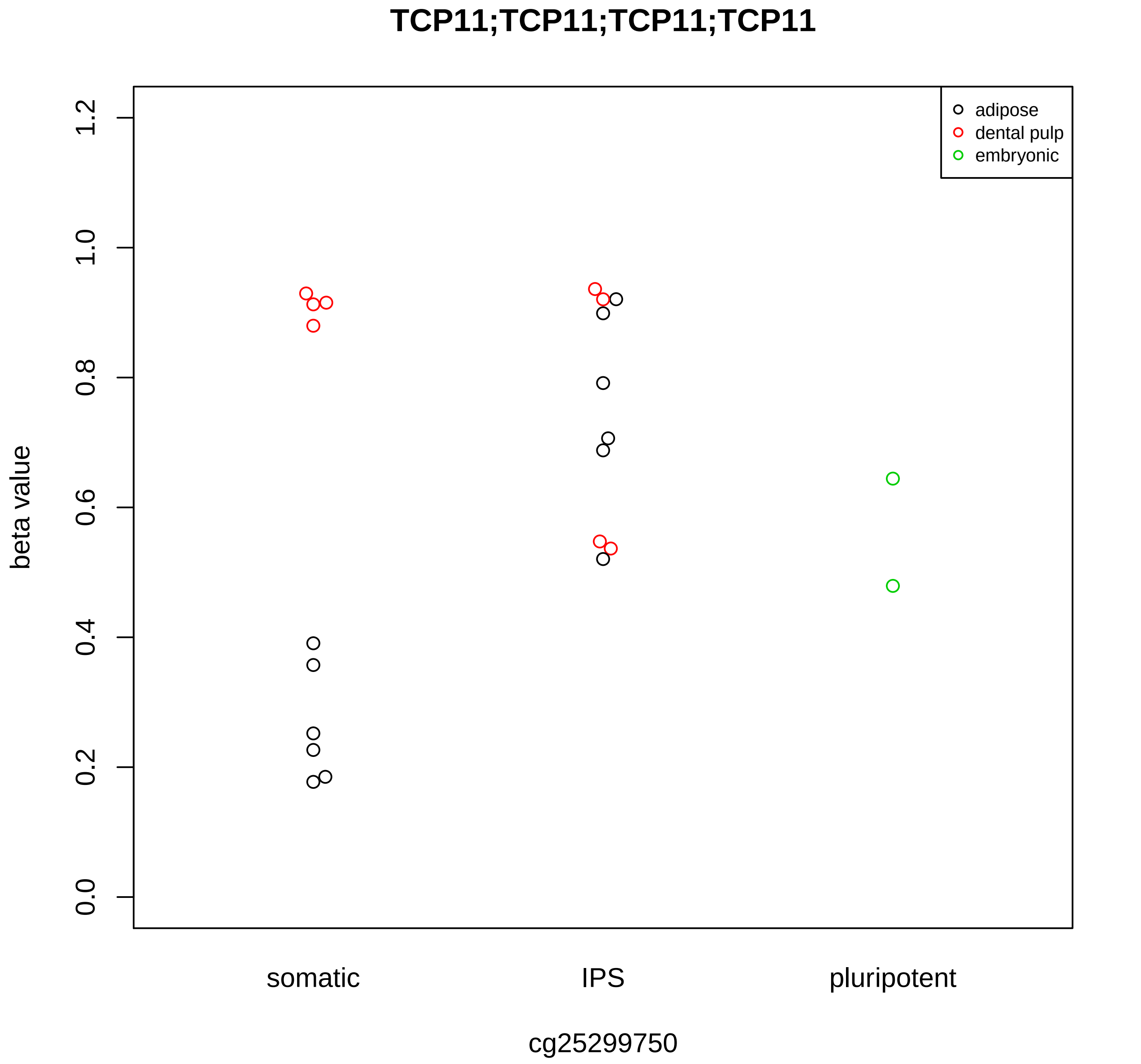
TCP11;TCP11;TCP11;TCP11
beta value
0.0
0.2
0.4
0.6
0.8
1.0
1.2
adipose
dental pulp
embryonic
somatic
IPS
pluripotent
cg25299750

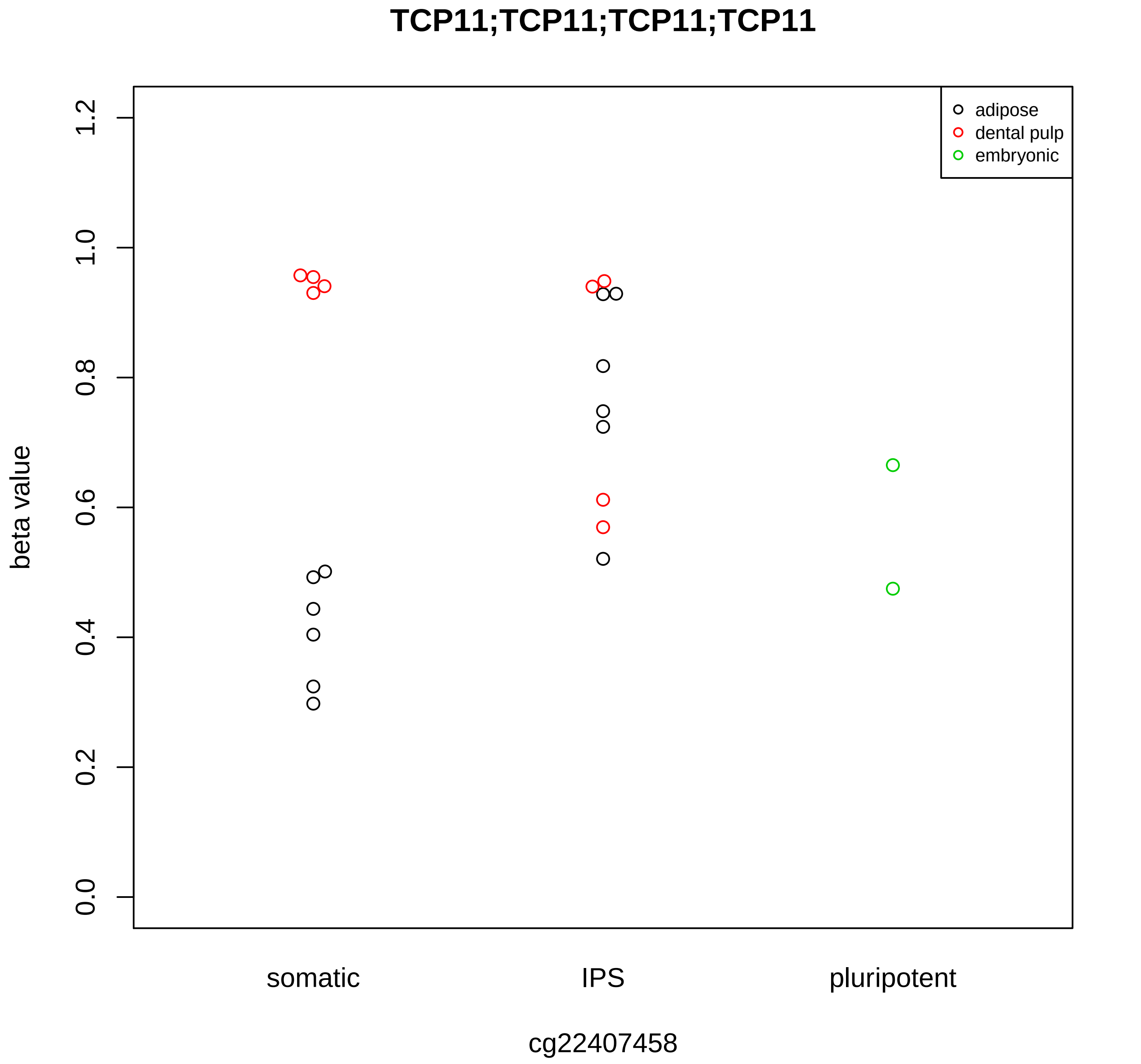
TCP11;TCP11;TCP11;TCP11
beta value
0.0
0.2
0.4
0.6
0.8
1.0
1.2
adipose
dental pulp
embryonic
somatic
IPS
pluripotent
cg22407458

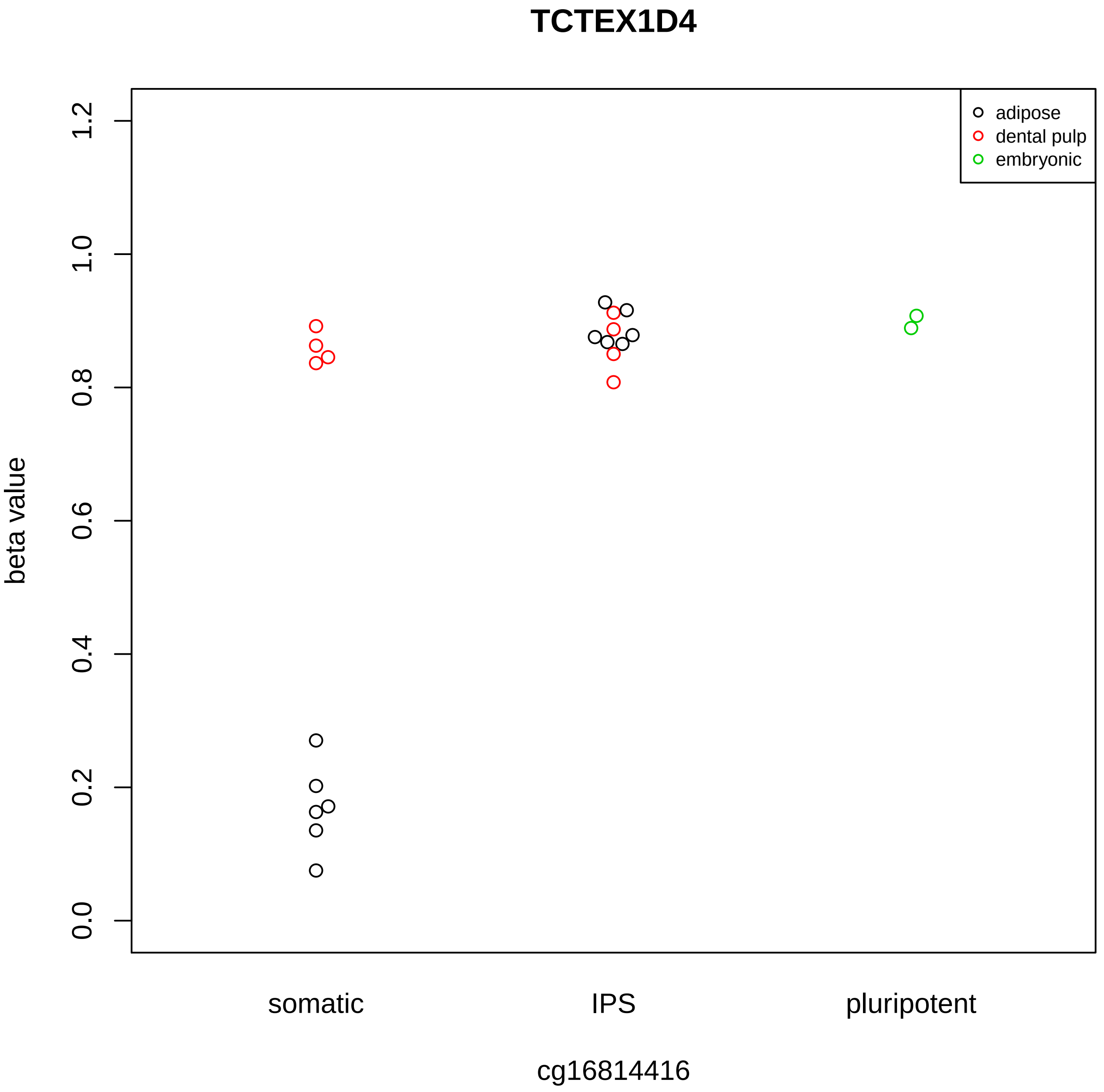
TCTEX1D4
beta value
0.0
0.2
0.4
0.6
0.8
1.0
1.2
adipose
dental pulp
embryonic
somatic
IPS
pluripotent
cg16814416

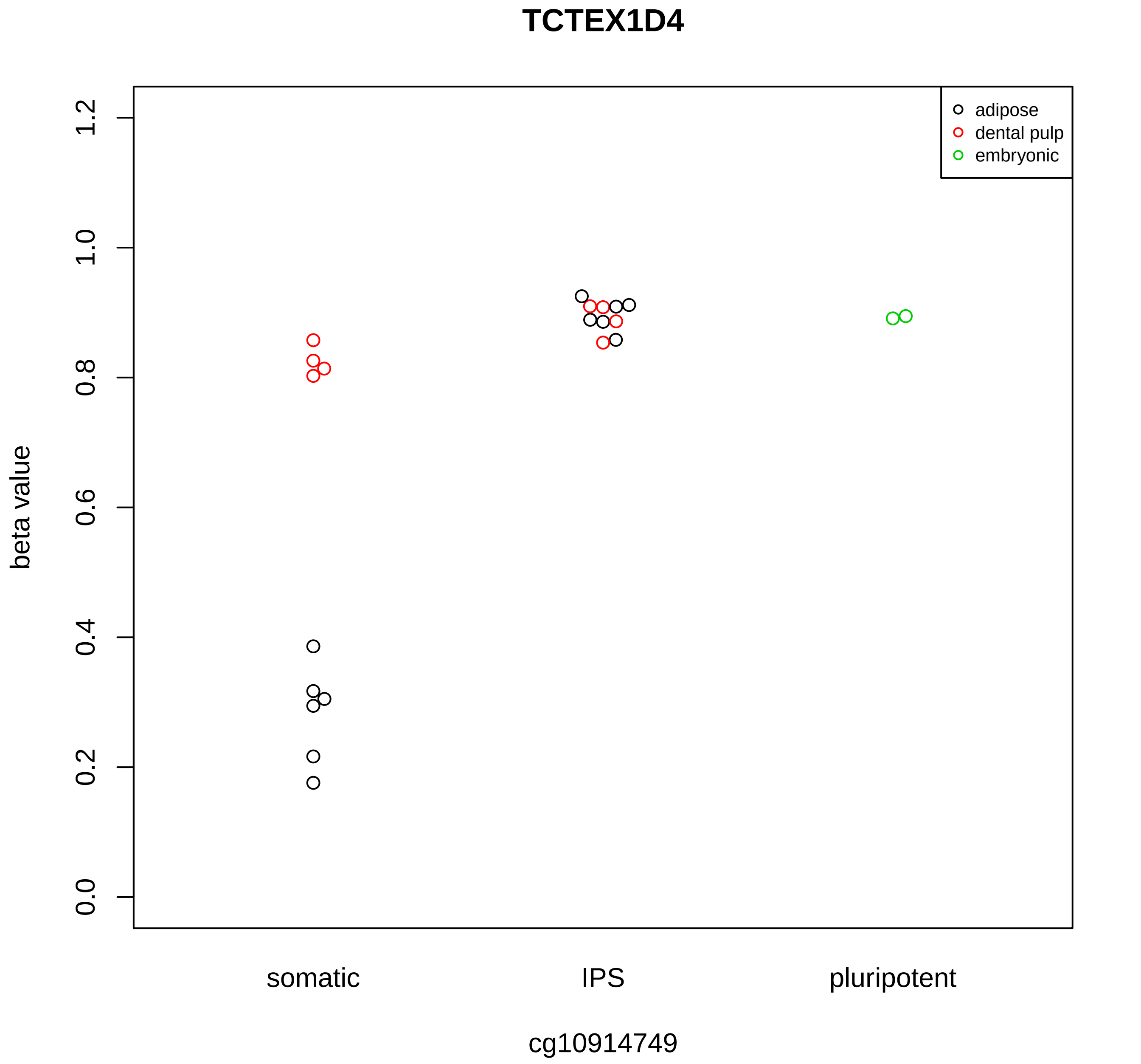
TCTEX1D4
adipose
dental pulp
embryonic
beta value
0.0
0.2
0.4
0.6
0.8
1.0
1.2
somatic
IPS
pluripotent
cg10914749

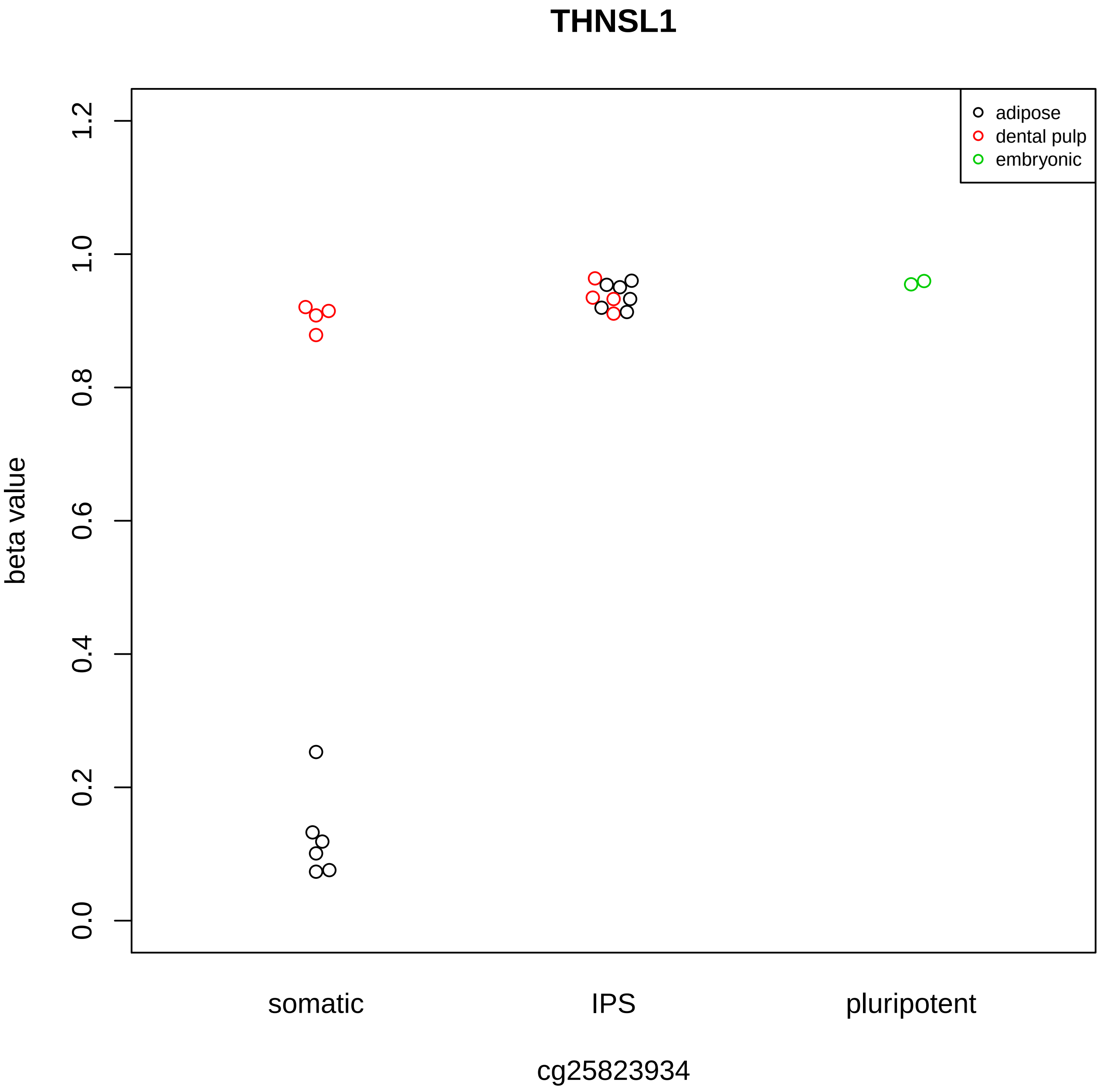
THNSL1
beta value
0.0
0.2
0.4
0.6
0.8
1.0
1.2
adipose
dental pulp
embryonic
somatic
IPS
pluripotent
cg25823934

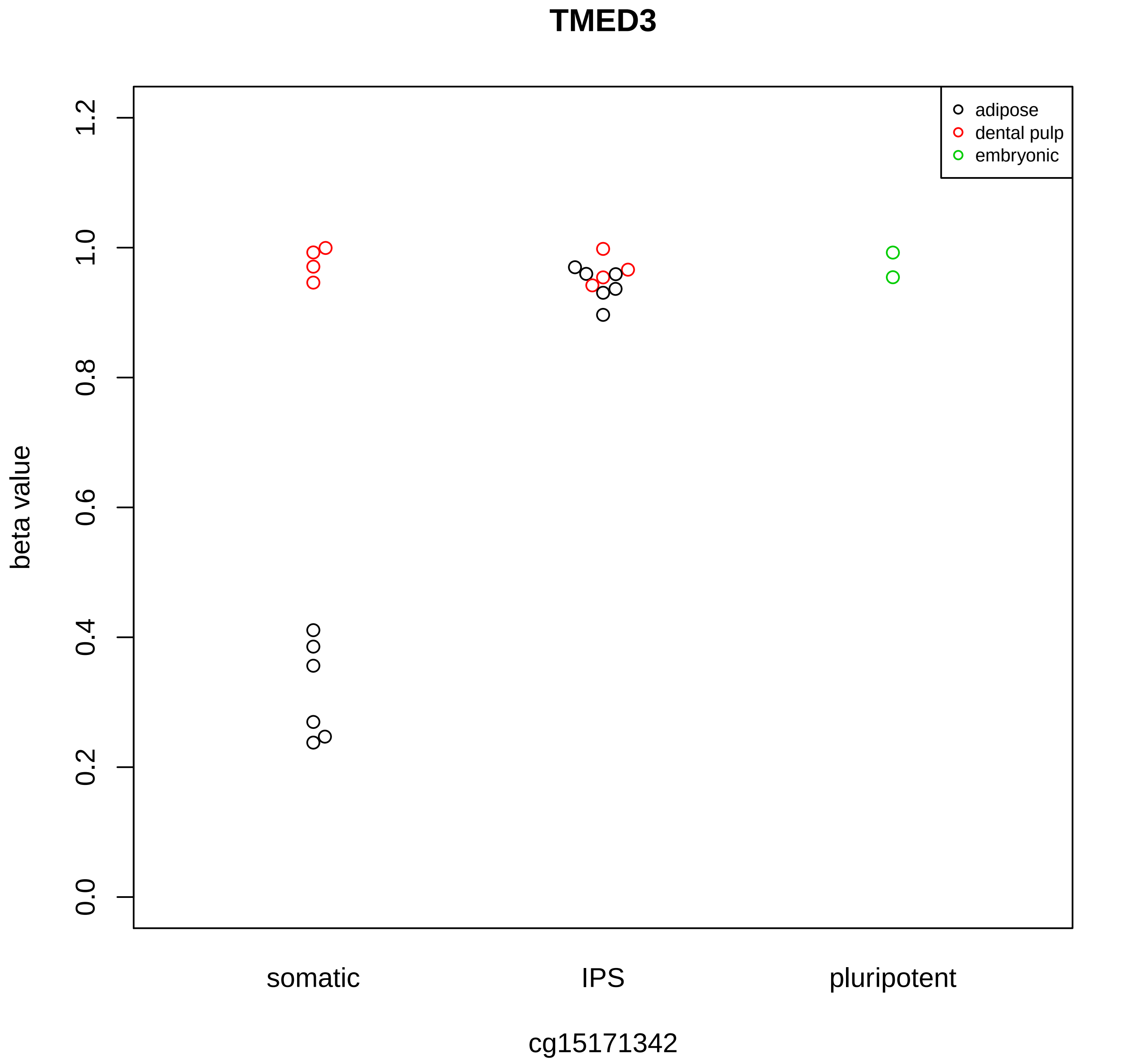
TMED3
adipose
dental pulp
embryonic
beta value
0.0
0.2
0.4
0.6
0.8
1.0
1.2
somatic
IPS
pluripotent
cg15171342

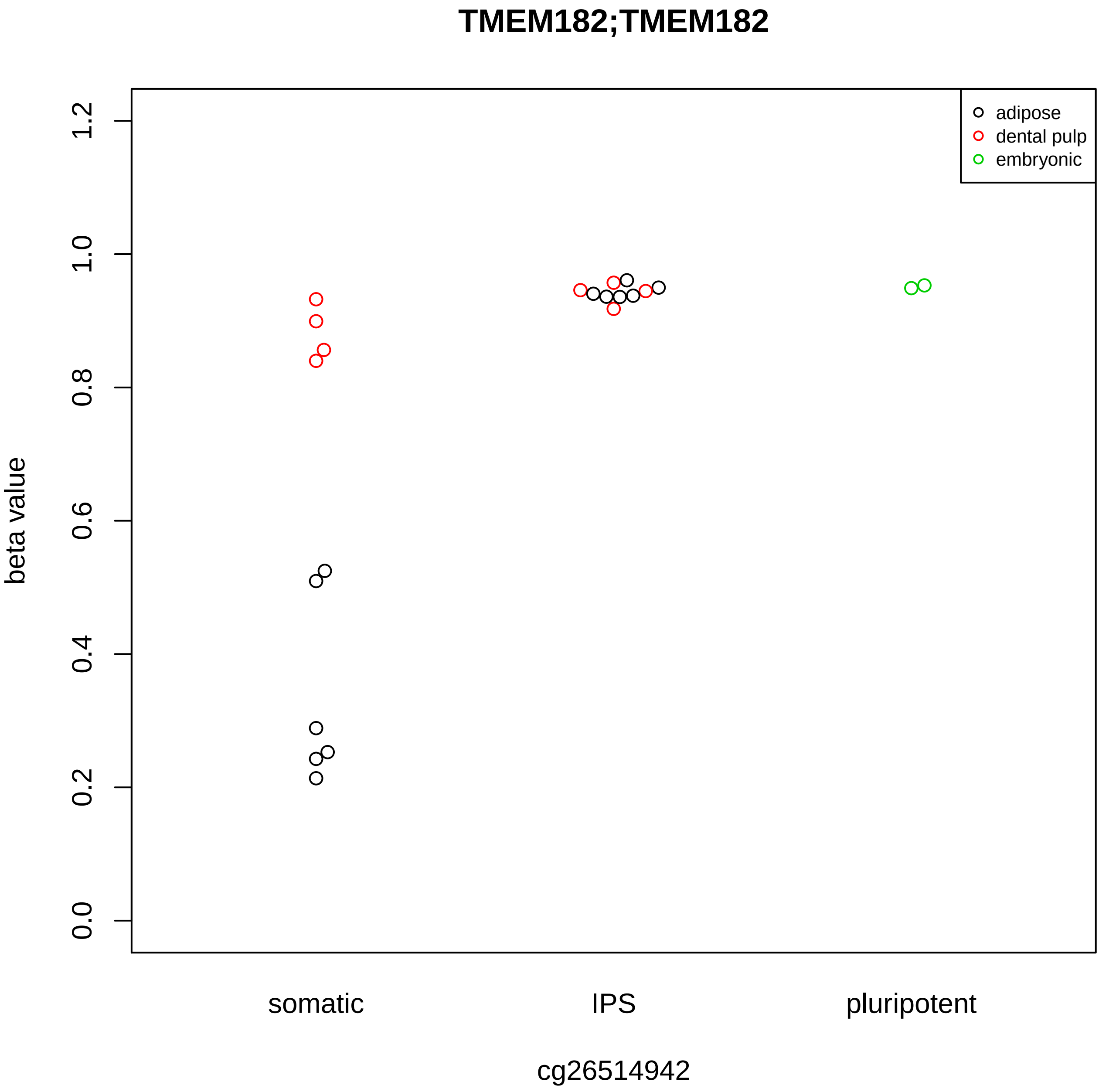
TMEM182;TMEM182
beta value
1.2
1.0
0.8
0.6
0.4
0.2
0.0
adipose
dental pulp
embryonic
somatic
IPS
pluripotent
cg26514942

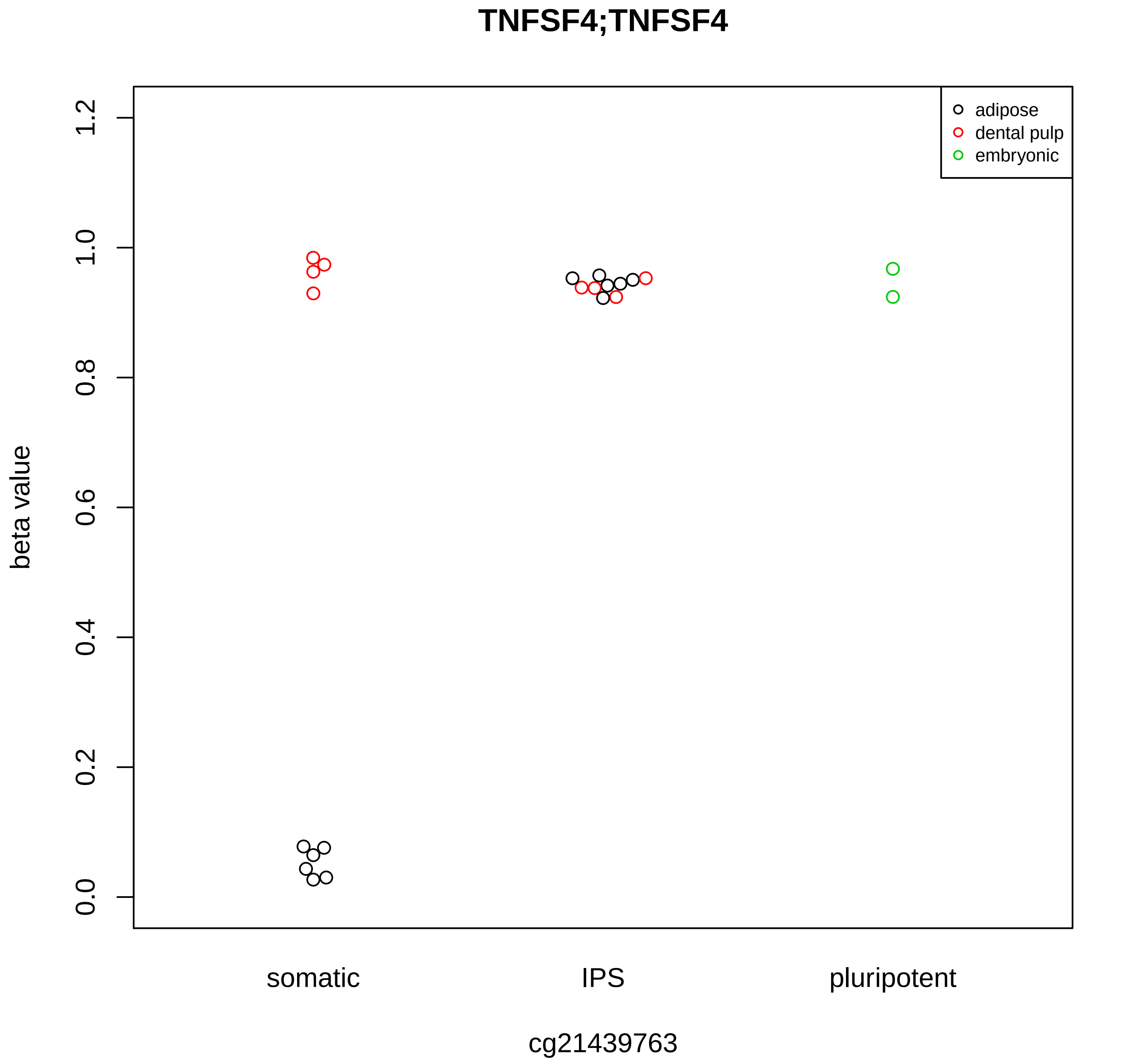

TNFSF4;TNFSF4
adipose
dental pulp
embryonic
beta value
0.0
0.2
0.4
0.6
0.8
1.0
1.2
somatic
IPS
pluripotent
cg21439763

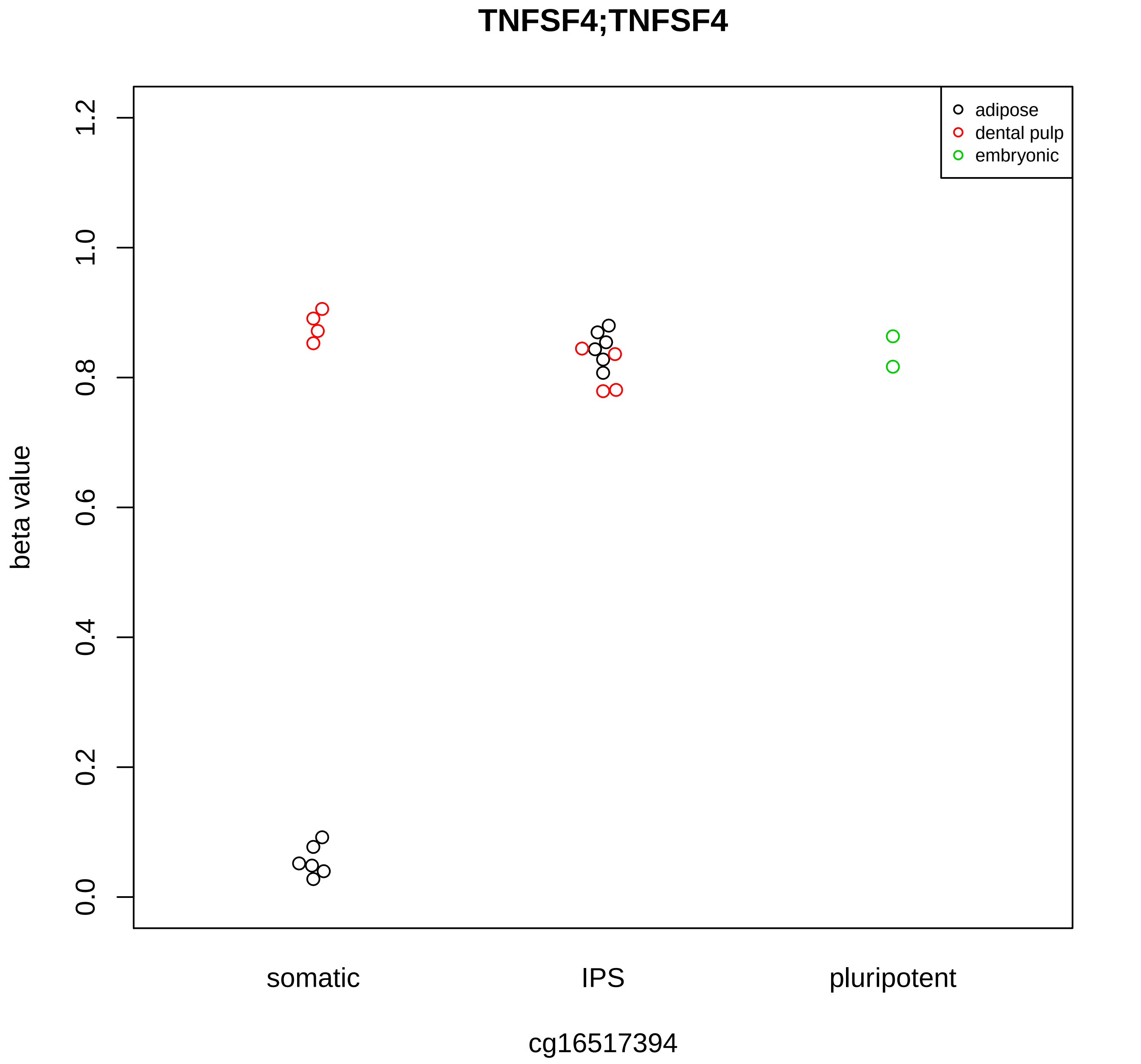
TNFSF4;TNFSF4
adipose
dental pulp
embryonic
beta value
0.0
0.2
0.4
0.6
0.8
1.0
1.2
somatic
IPS
pluripotent
cg16517394

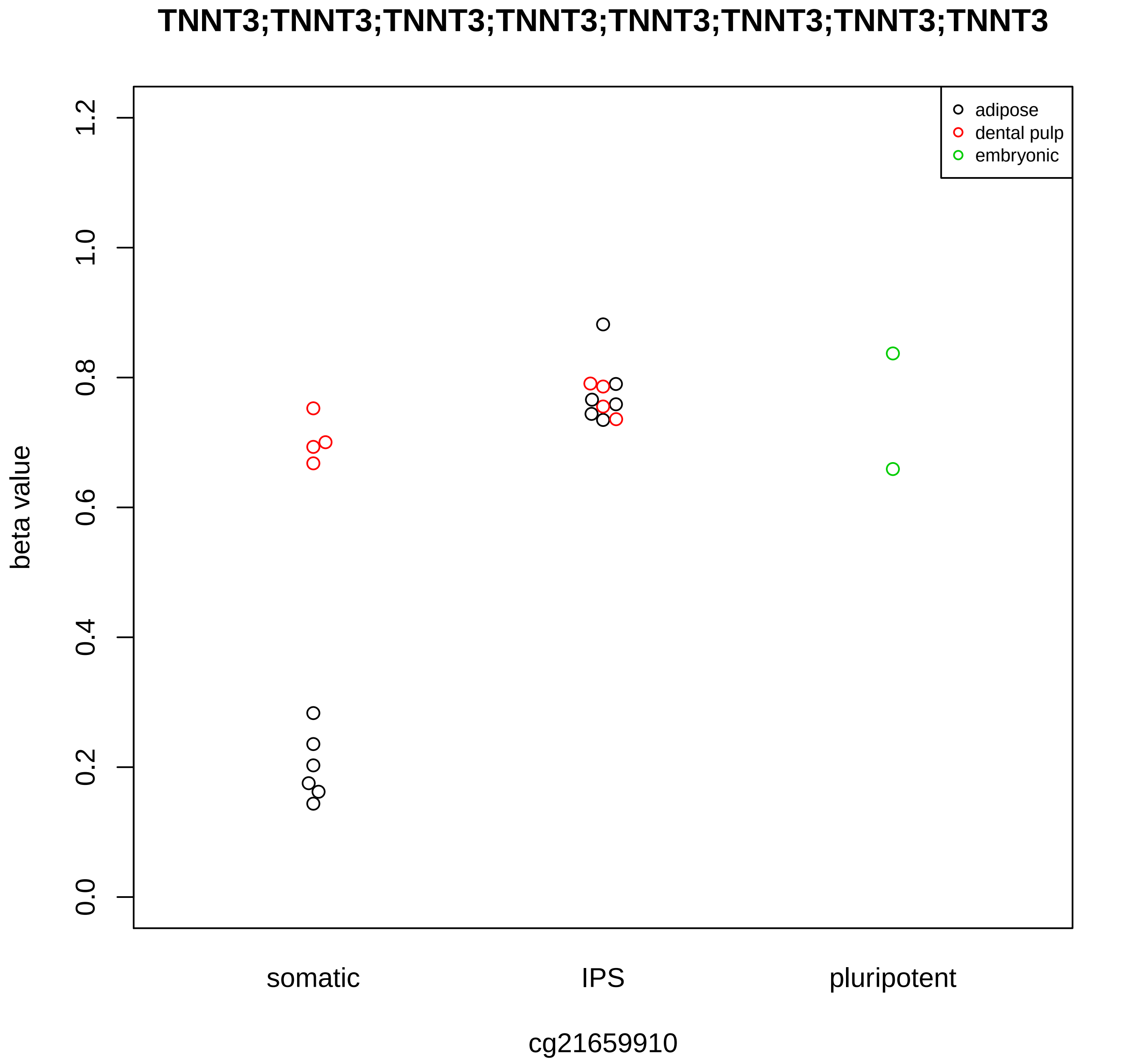

TNNT3;TNNT3;TNNT3;TNNT3;TNNT3;TNNT3;TNNT3;TNNT3
beta value
0.0
0.2
0.4
0.6
0.8
1.0
1.2
adipose
dental pulp
embryonic
somatic
IPS
pluripotent
cg21659910

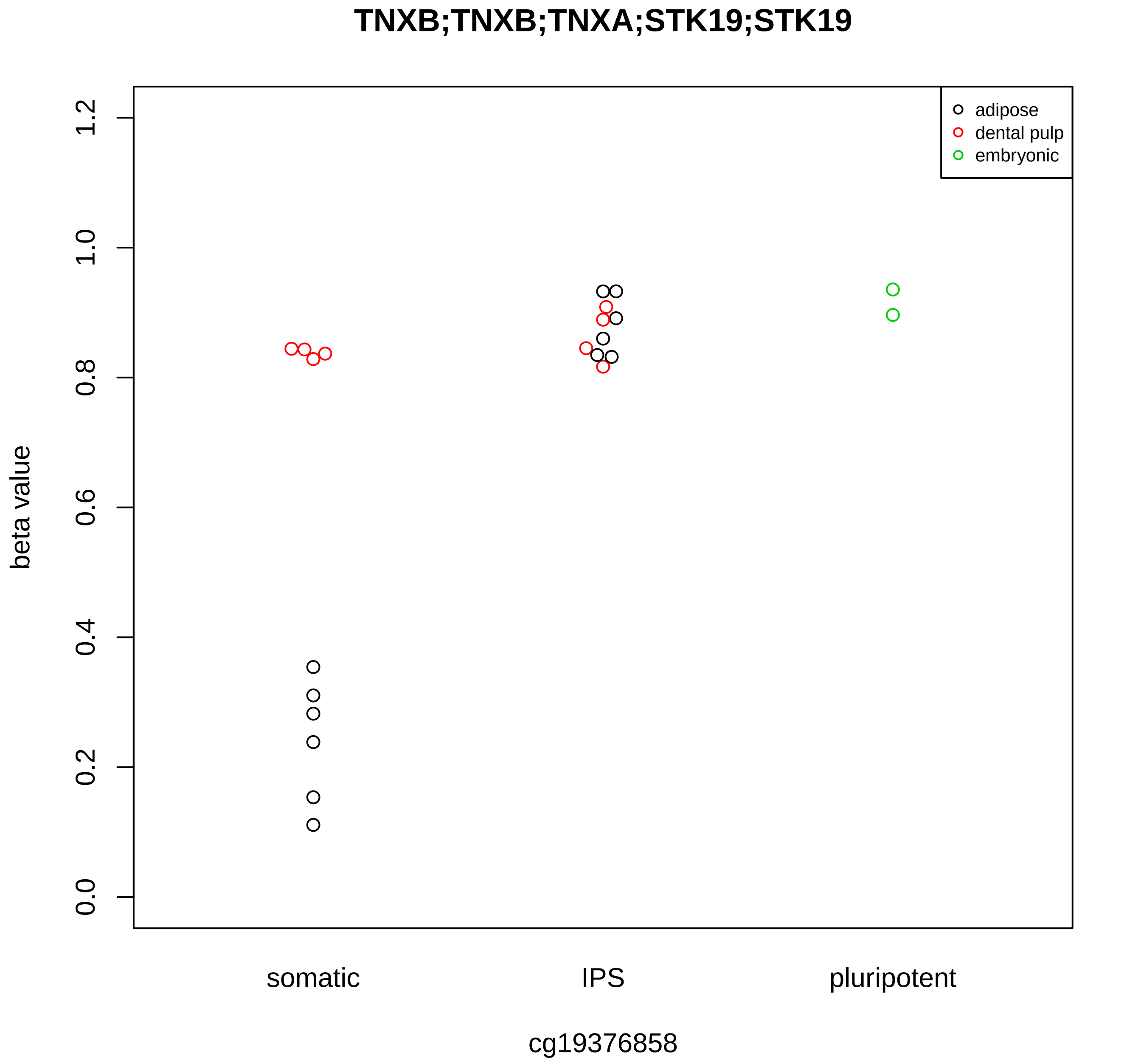
TNXB;TNXB;TNXA;STK19;STK19
beta value
0.0
0.2
0.4
0.6
0.8
1.0
1.2
adipose
dental pulp
embryonic
somatic
IPS
pluripotent
cg19376858

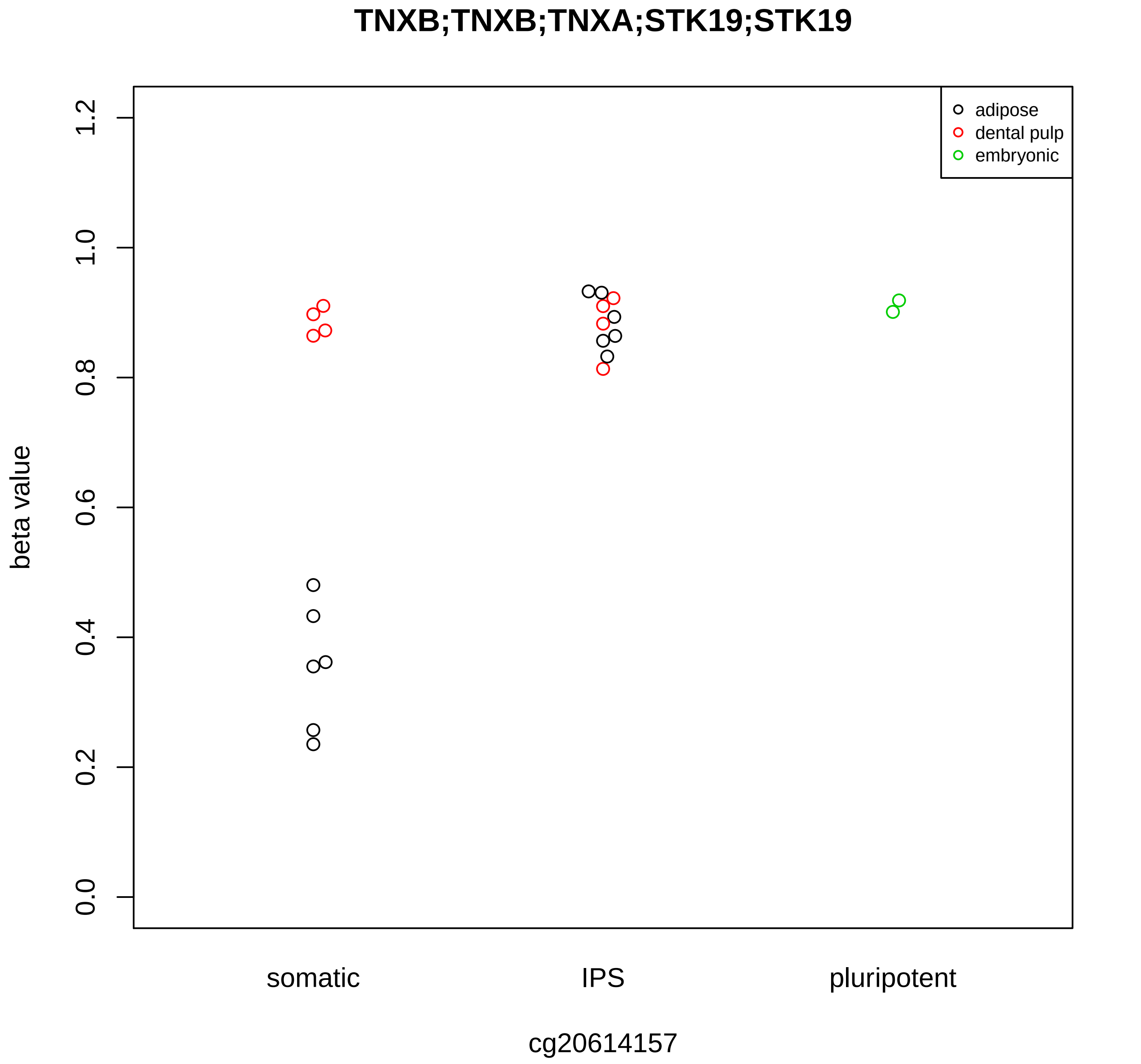
TNXB;TNXB;TNXA;STK19;STK19
adipose
dental pulp
embryonic
beta value
0.0
0.2
0.4
0.6
0.8
1.0
1.2
somatic
IPS
pluripotent
cg20614157

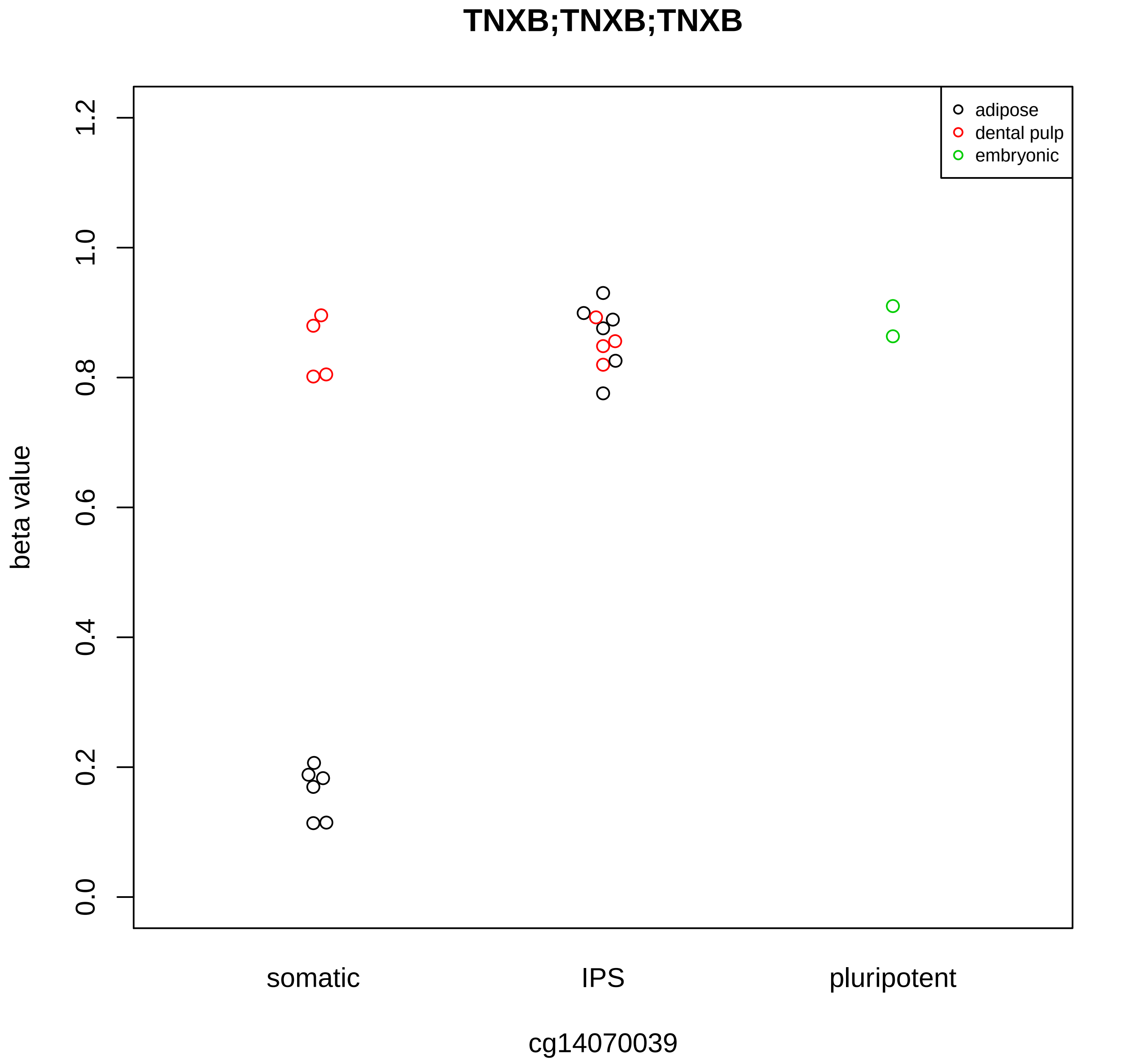
TNXB;TNXB;TNXB
adipose
dental pulp
embryonic
beta value
0.0
0.2
0.4
0.6
0.8
1.0
1.2
somatic
IPS
pluripotent
cg14070039

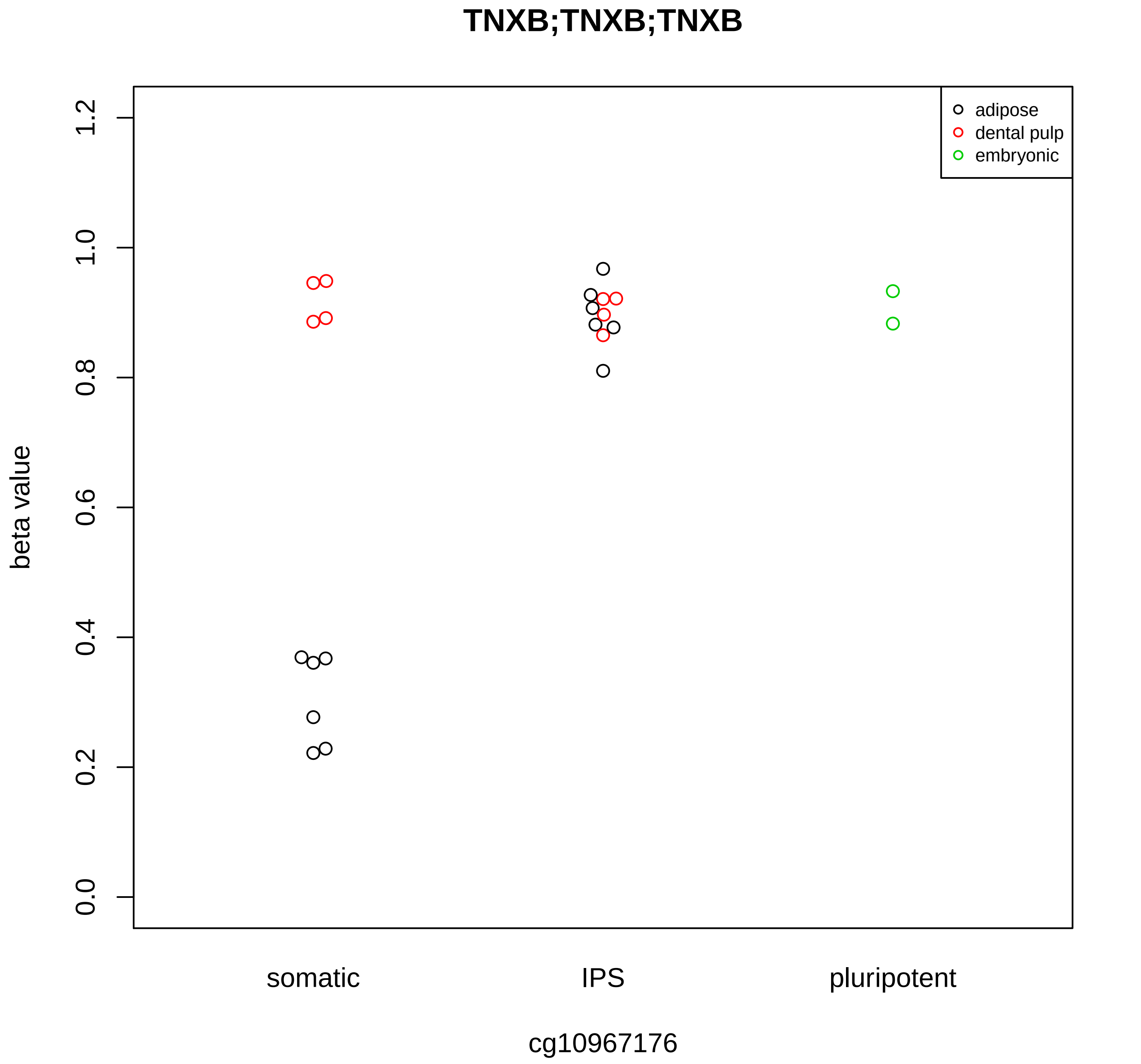
TNXB;TNXB;TNXB
beta value
0.0
0.2
0.4
0.6
0.8
1.0
1.2
adipose
dental pulp
embryonic
somatic
IPS
pluripotent
cg10967176

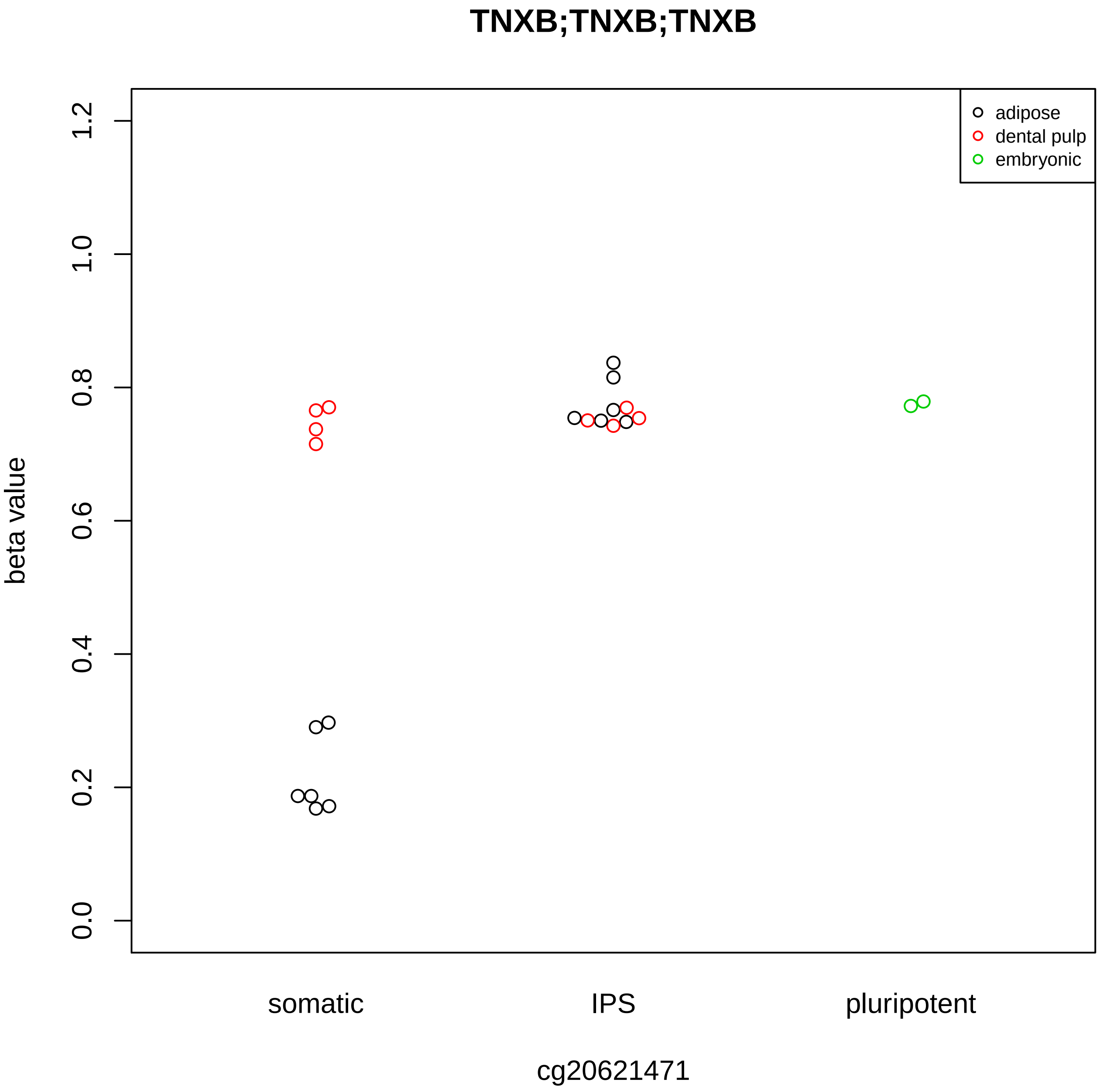

TNXB;TNXB;TNXB
beta value
0.0
0.2
0.4
0.6
0.8
1.0
1.2
adipose
dental pulp
embryonic
somatic
IPS
pluripotent
cg20621471

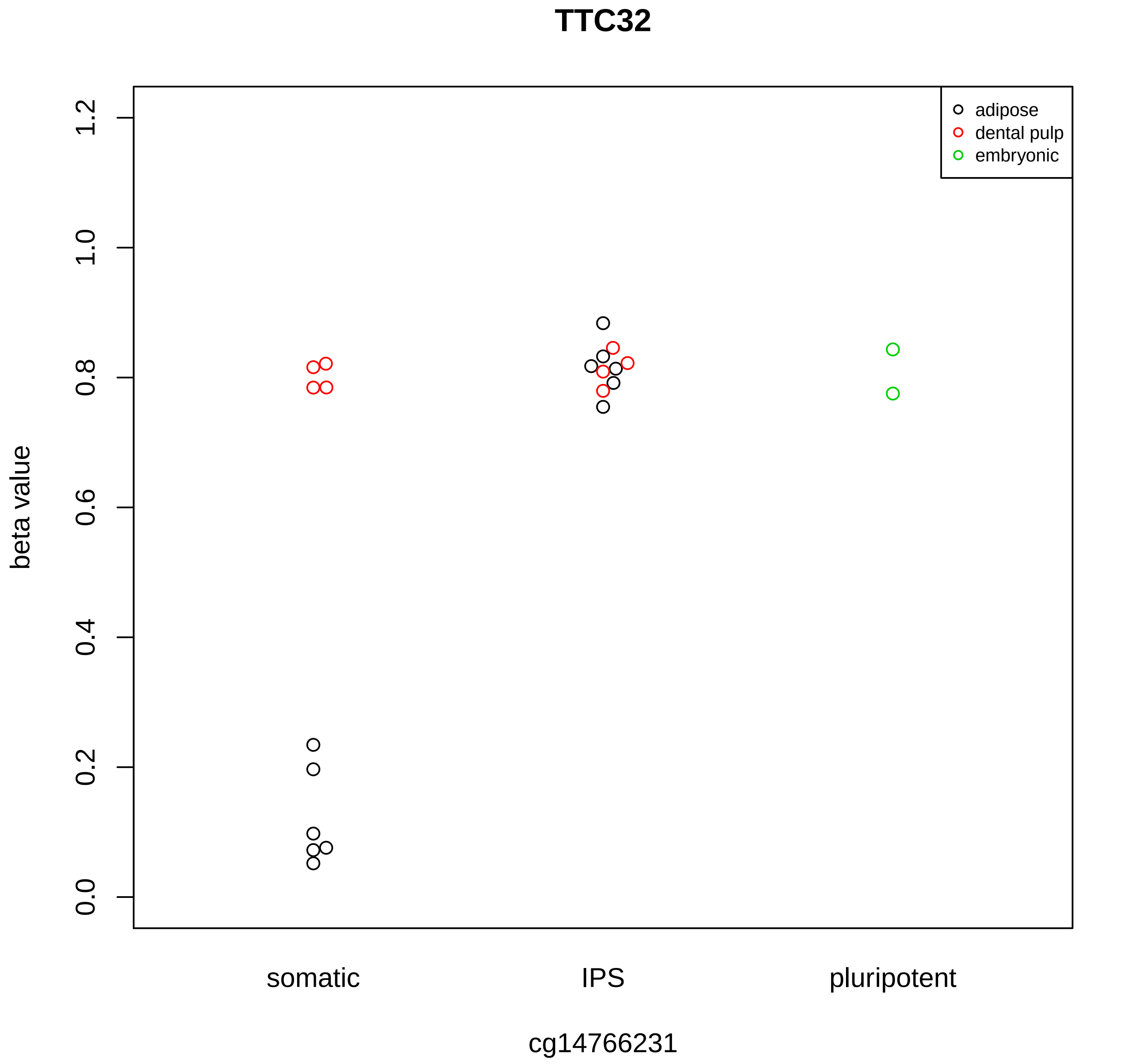
TTC32
beta value
1.2
1.0
0.8
0.6
0.4
0.2
0.0
adipose
dental pulp
embryonic
somatic
IPS
pluripotent
cg14766231

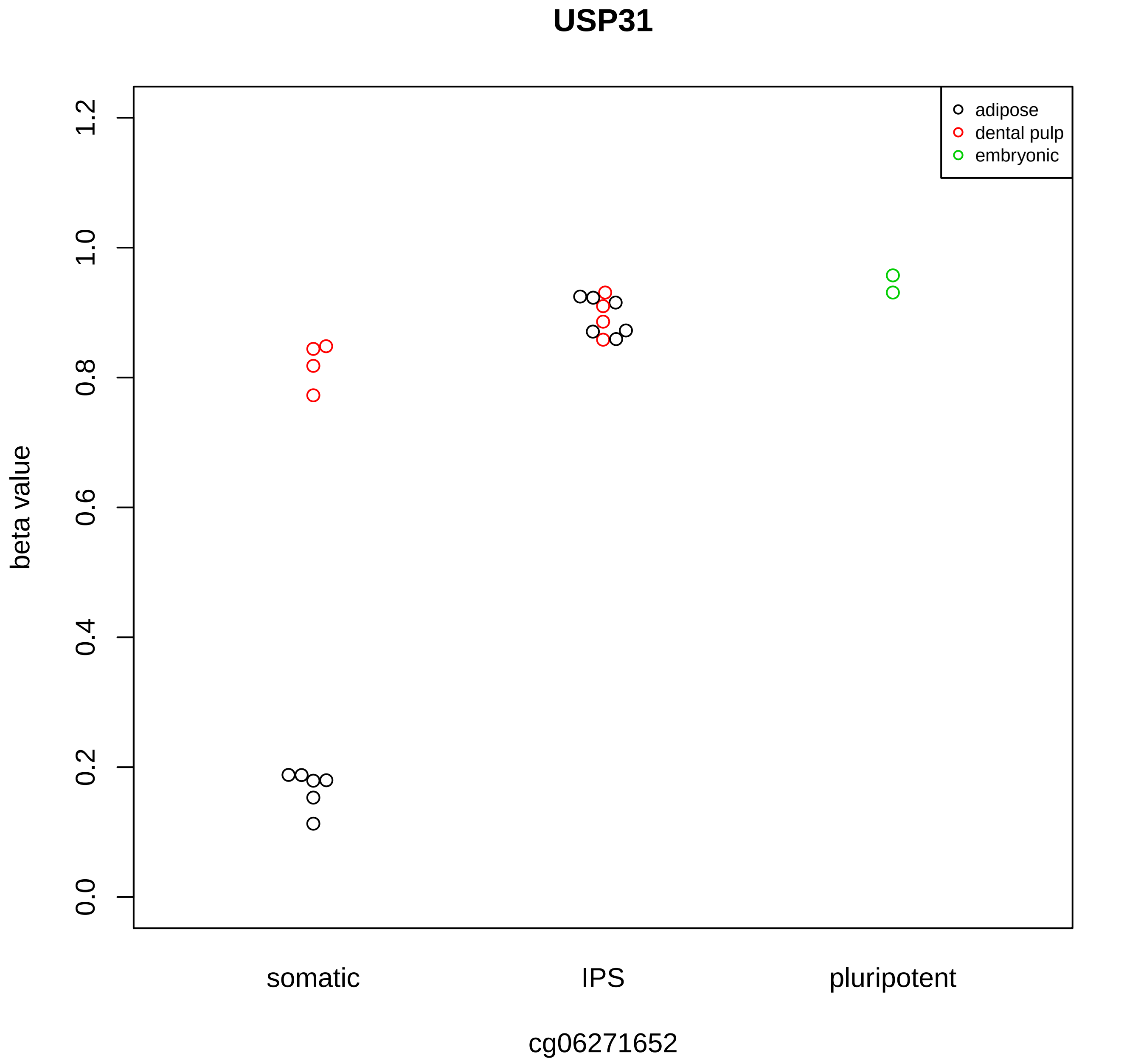
USP31
beta value
0.0
0.2
0.4
0.6
0.8
1.0
1.2
adipose
dental pulp
embryonic
somatic
IPS
pluripotent
cg06271652

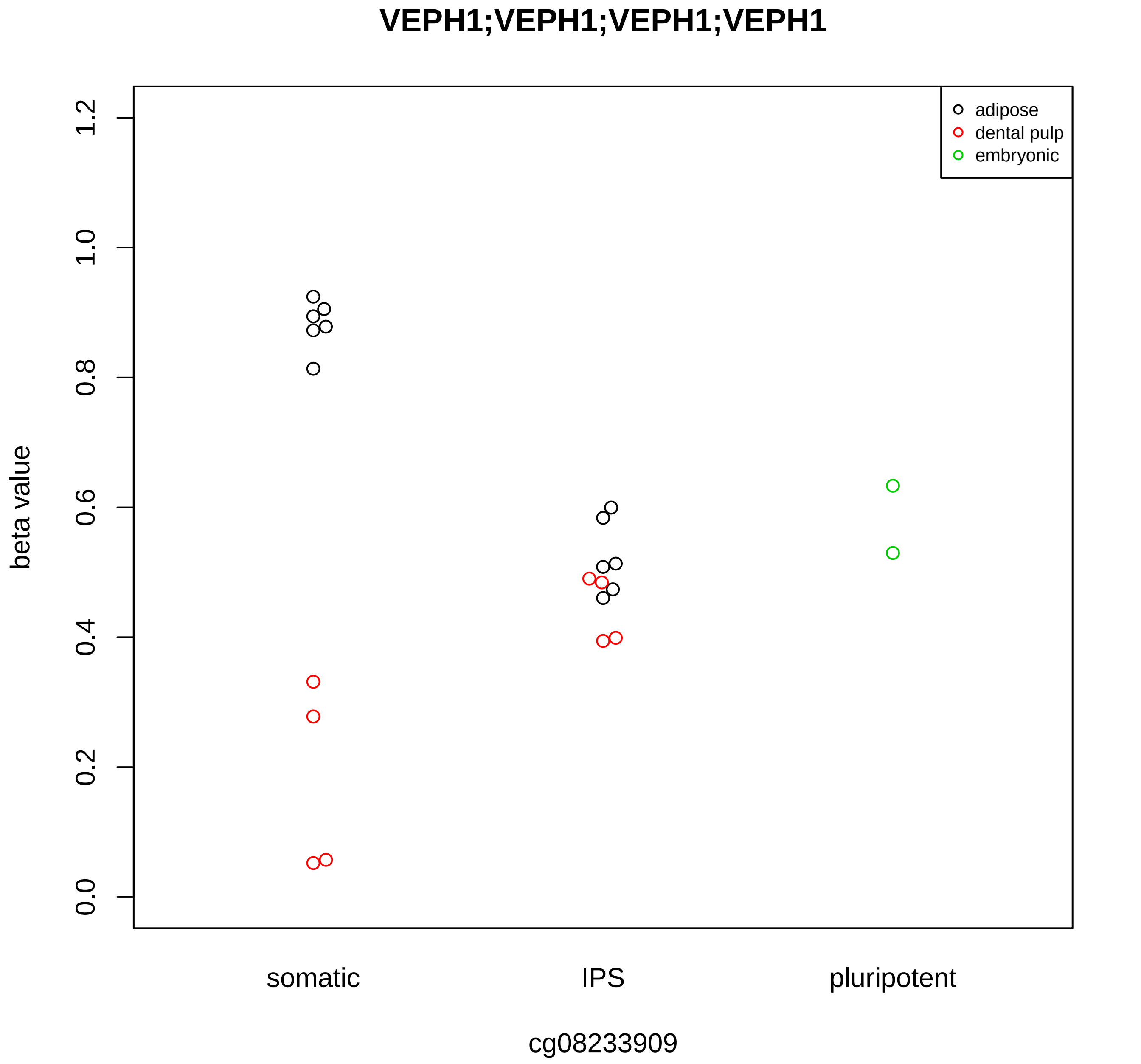

VEPH1;VEPH1;VEPH1;VEPH1
adipose
dental pulp
embryonic
beta value
0.0
0.2
0.4
0.6
0.8
1.0
1.2
somatic
IPS
pluripotent
cg08233909

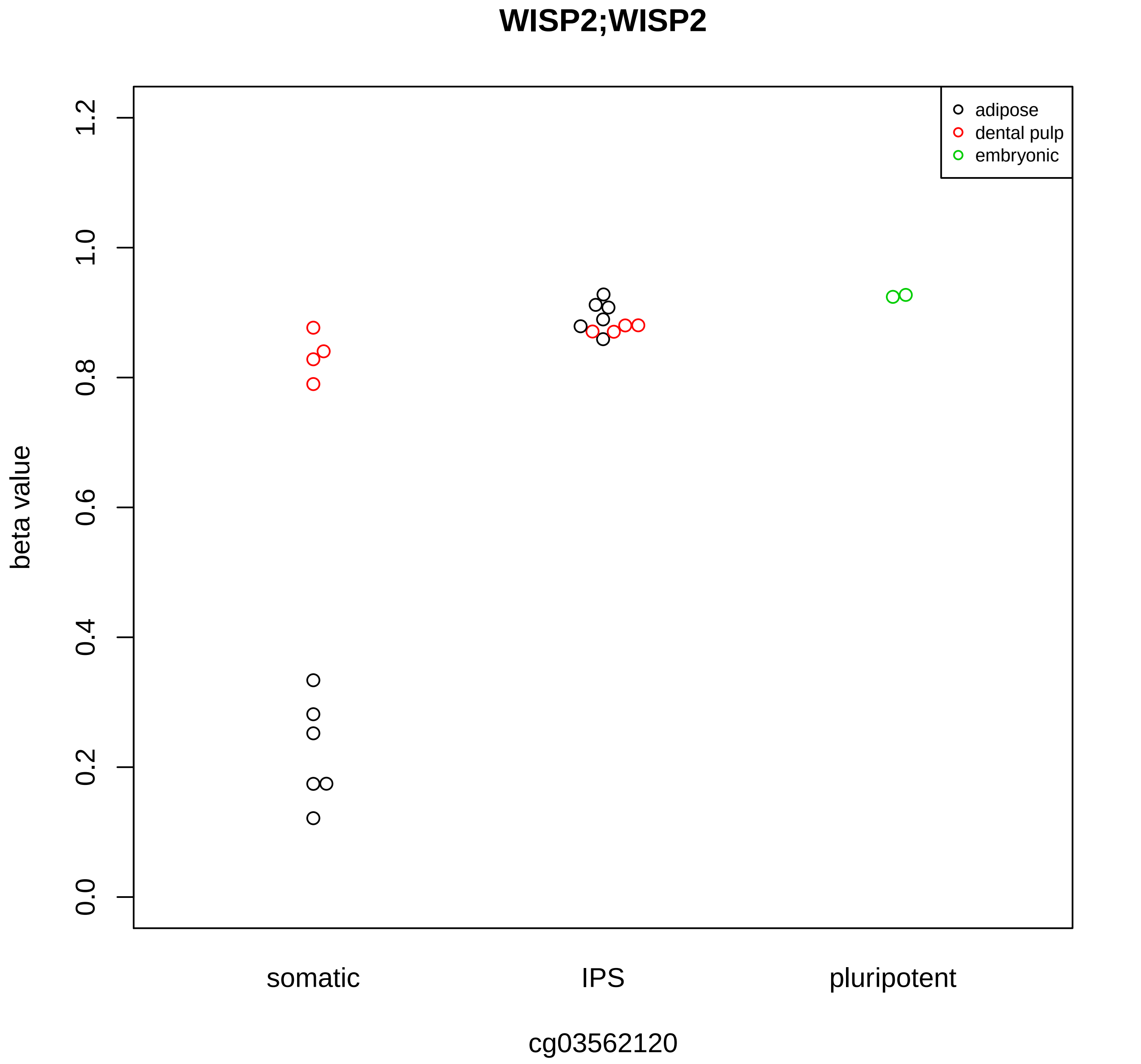
WISP2;WISP2
beta value
1.2
1.0
0.8
0.6
0.4
0.2
0.0
adipose
dental pulp
embryonic
somatic
IPS
pluripotent
cg03562120

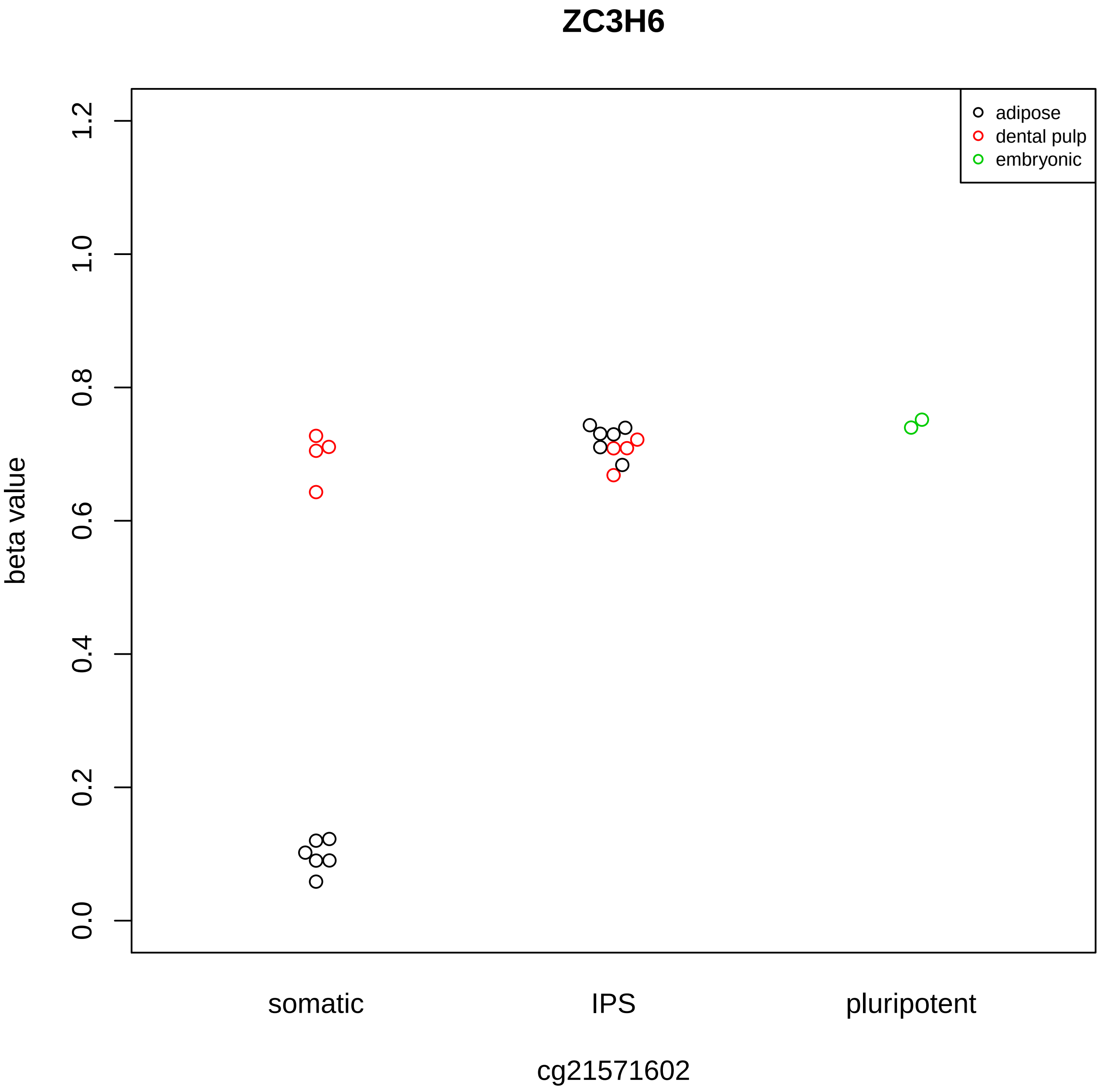
ZC3H6
beta value
0.0
0.2
0.4
0.6
0.8
1.0
1.2
adipose
dental pulp
embryonic
somatic
IPS
pluripotent
cg21571602

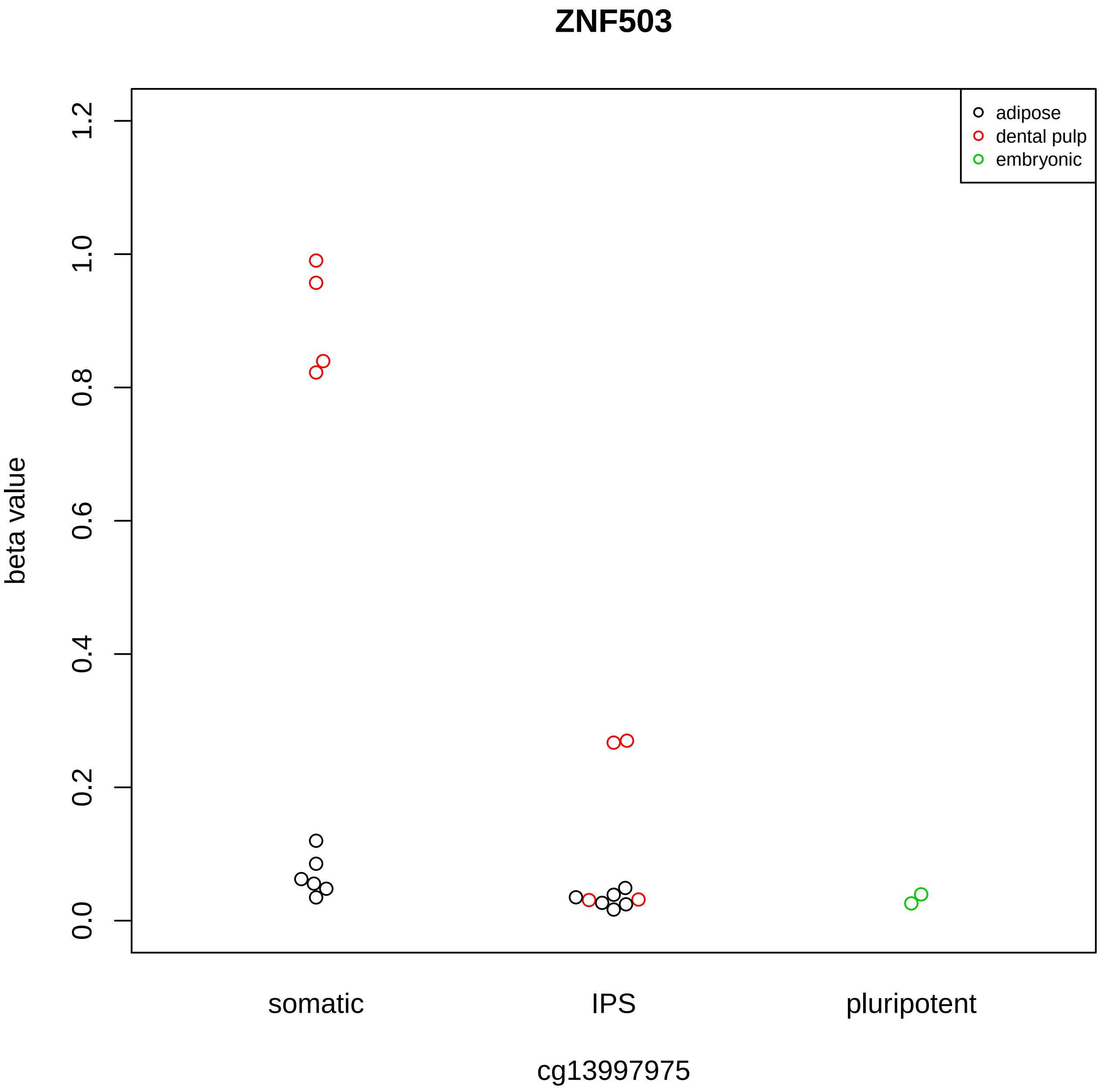
ZNF503
beta value
1.2
1.0
0.8
0.6
0.4
0.2
0.0
adipose
dental pulp
embryonic
somatic
IPS
pluripotent
cg13997975
