# Supplementary figures and images for: Amenable epigenetic traits of dental pulp stem cells underlie high capability of xeno-free episomal reprogramming
Source: Stem Cell Res Ther. 2018 Mar 20;9:68. doi: 10.1186/s13287-018-0796-2 (PMC5859503; doi:10.1186/s13287-018-0796-2)

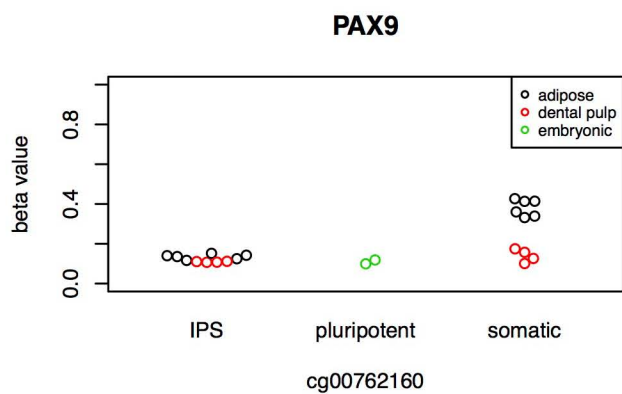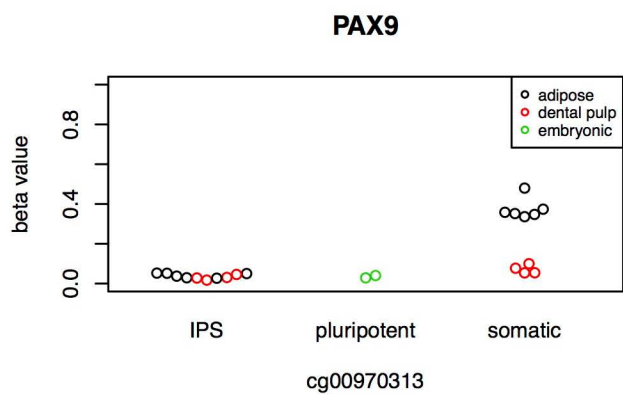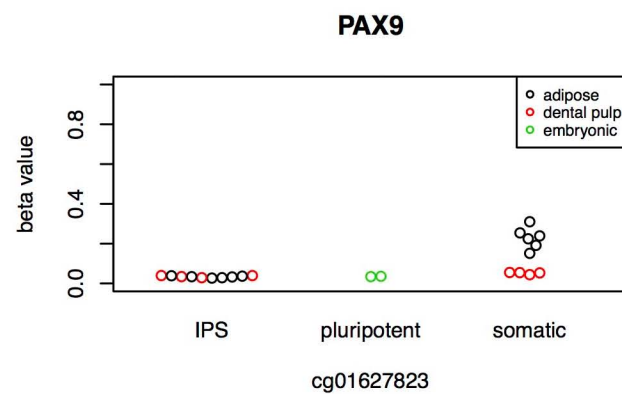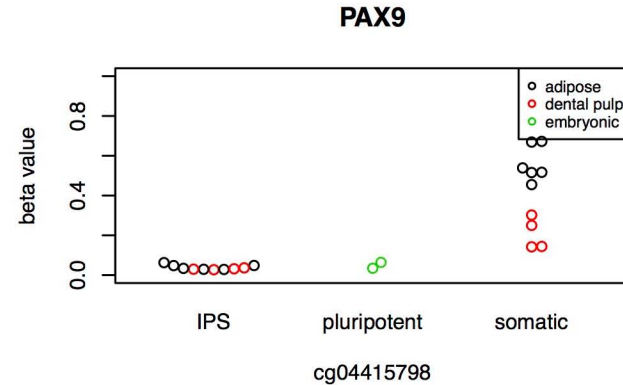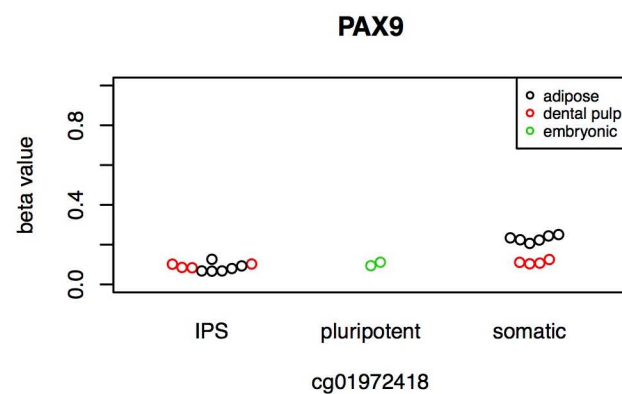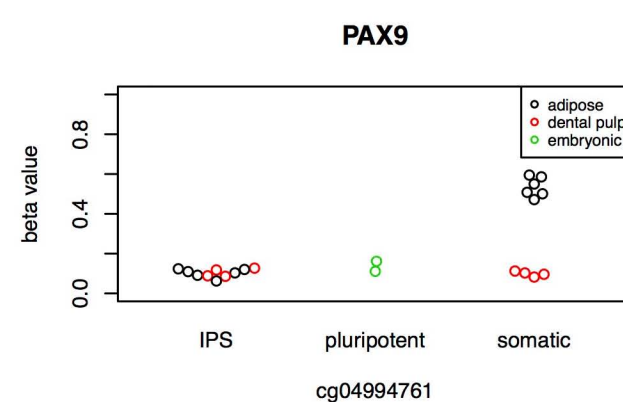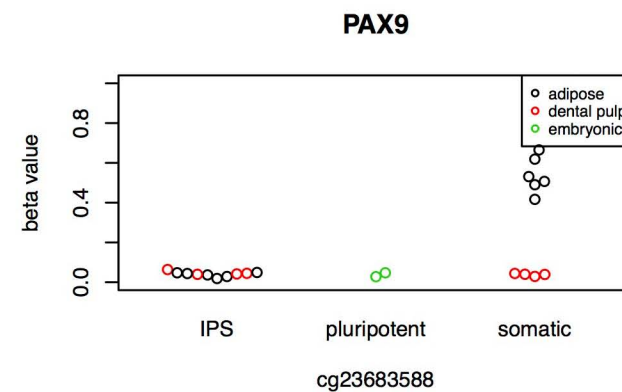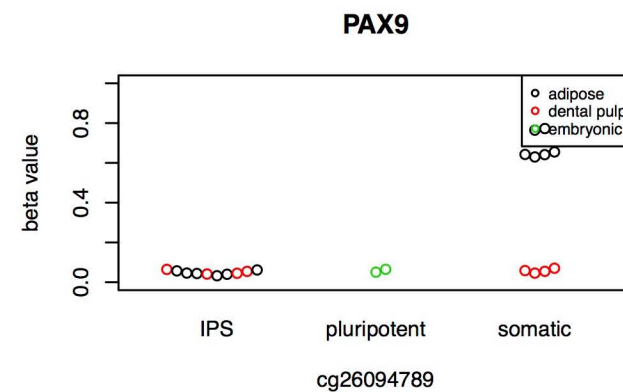

Supplement: Supplementary file 1 — Table S1. List of commercial and patient-derived dental stem cell samples used. Table S2. DiPS colony counts of dental pulp stem cell samples used for reprogramming under different culture conditions. Figure S1. Transduction efficiencies for reprogramming DPSCs. Figure S2. Generation of iPS cells from DPSCs in the presence of enhancer compounds. Figure S3. Time course during episomal-based reprogramming of DPSCs. Figure S4. Characterisation of DPSCs cultured in xeno-free media. Figure S5. Cytogenetics and in vivo characterisation of DiPS lines generated under xeno-free culture conditions. Table S3. List of differentially methylated regions of selected genes in DPSCs versus ASCs with respect to iPS (DiPS and AiPS) and H1 hES cell lines. Table S4. DNA methylation raw data analysed with multiple probe sets of PAX9 gene that exhibit significant differences between DPSCs and ASCs. Table S5. Top networks by ingenuity pathway analysis (IPA) for differentially methylated genes in ASCs versus AiPS cells that do not exhibit such differences in DPSCs versus DiPS cells. Figure S6. Pluripotent and self-renewal supporting characteristics of DPSCs. (ZIP 8876 kb) [file 13287_2018_796_MOESM1_ESM.zip › Table S4.pdf]
